# Enantioselective lactonization catalyzed by chiral N-heterocyclic carbenes enables access to inherently chiral eight-membered lactones

**DOI:** 10.1039/d5sc05037e

**Published:** 2025-08-25

**Authors:** Vojtěch Dočekal, Adam Kurčina, Ivana Císařová, Jan Veselý

**Affiliations:** a Department of Organic Chemisty, Faculty of Science, Charles University Hlavova 2030/8 128 00 Prague 2 Czech Republic vojtech.docekal@natur.cuni.czJan jan.vesely@natur.cuni.cz; b Department of Inorganic Chemistry, Faculty of Science, Charles University Hlavova 2030/8 128 00 Prague 2 Czech Republic

## Abstract

Chiral saddle-shaped molecules are an emerging class of compounds with significant potential in both materials science and medicinal chemistry. However, their broader application has been hindered by limited synthetic accessibility. Herein, we report a metal-free, organocatalytic protocol for the oxidative lactonization of readily available aldehydic derivatives, enabling the efficient synthesis of chiral saddle-shaped lactones. The method exhibits excellent enantiocontrol, high yields (nearly quantitative), and broad functional group tolerance, as demonstrated by the synthesis of a small library of structurally diverse products. The scalability of the reaction further underscores its practical utility. Moreover, computational studies provide mechanistic insight into the origin of enantioinduction in N-heterocyclic carbene-catalyzed lactonization.

## Abstract 2

Chiral saddle-shaped molecules have emerged as a promising class of compounds with significant potential. In this work, we describe a metal-free, enantioselective lactonization protocol that provides access to inherently chiral lactones.

## Graphical abstract

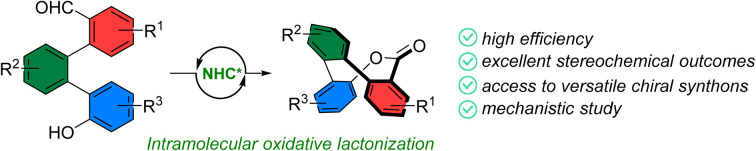

## Introduction

**Fig. 1:**
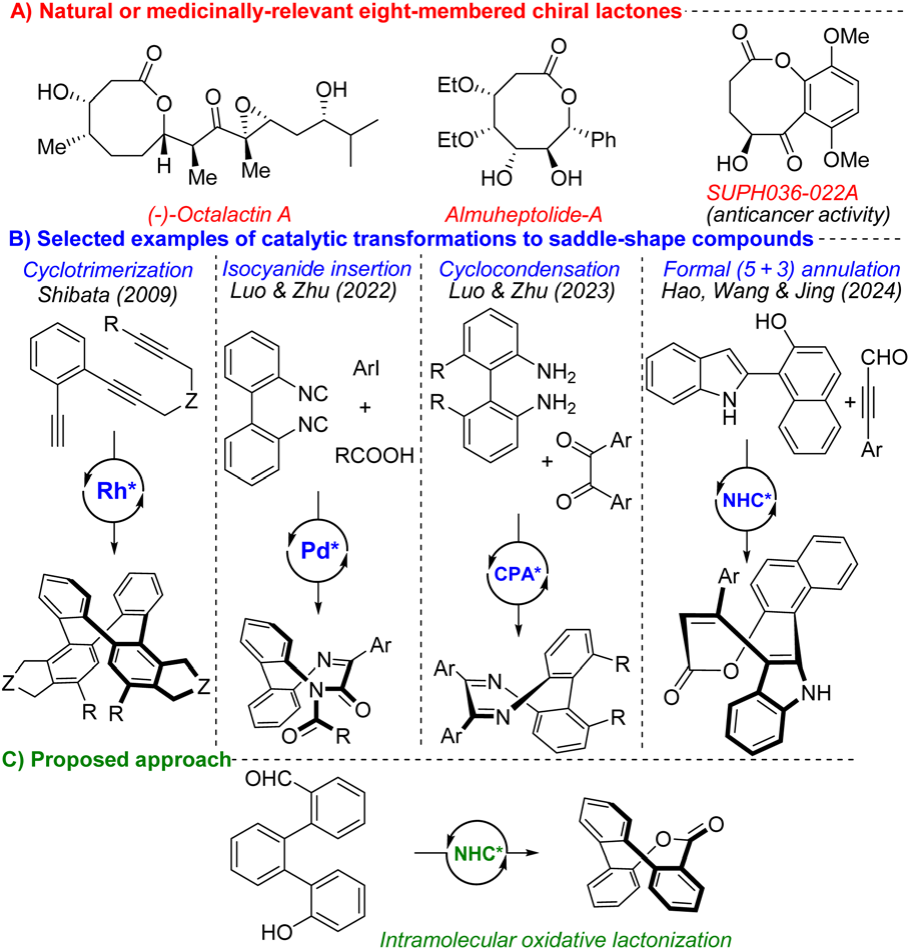
(A) Selected examples of natural or medicinally-relevant chiral eight-membered lactones. (B) Selected accesses to enantioenriched saddle-shape molecules. (C) Our proposed approach.

Chiral medium-sized rings, particularly eight-membered carbocycles and heterocycles, occupy a unique structural position in modern organic chemistry.^1^ Notably, chiral eight-membered ring systems, often incorporating one or more stereogenic centers, are prevalent structural motifs in natural products, therapeutic agents, and functional molecular materials (Fig. 1A).^2^ Unlike their smaller and larger analogs, these ring systems are inclined to adopt three-dimensional, twisted geometries that can be both rigid and asymmetric, even in the absence of classical chiral elements, such as central, axial, planar, or helical chirality. Such molecules are now referred to as saddle-shaped molecules, belonging to a family of inherently chiral compounds.^3^ Saddle-shaped molecules in their conformations can exhibit stable chirality when the energy barrier for racemization is sufficiently high. The resulting inherent chirality is commonly present not only when conformational stability is enhanced by the presence of bulky substituents, but also in sp^2^-rich structures, such as tetraphenylenes and related molecules.^4^

Tetraphenylenes, known also as tetrabenzocyclooctatetraenes, are sp^2^-rich eight-membered cycles characterized by their rigid, saddle-shaped structures with remarkable conformational stability.^5^ Owing to their three-dimensional architecture and directionally fixed substituents, tetraphenylenes and related molecules have emerged as promising scaffolds for the design of self-assembling systems, chiral catalysts, and supramolecular materials.^6^ However, synthesizing inherently chiral saddle-shaped architectures remains a significant challenge due to the enthalpic and entropic penalties associated with medium-ring closure and conformational locking.^7^

Recent advances in both transition-metal catalysis and organocatalysis have led to the development of several enantioselective routes to conformationally stable saddle-shaped molecules.^8^ The first catalytic and highly enantioselective synthesis of chiral eight-membered tetraphenylenes was described by Shibata,^9^ using Rh-catalyzed consecutive inter- and intramolecular cycloaddition (Fig. 1B). More than a decade later, Luo and Zhu developed an enantioselective synthesis of new saddle-shaped analogs of tetraphenylenes, diazocines, using Pd-catalyzed double isocyanide insertion (Fig. 1B).^10^ Soon after, the same authors reported additional approaches to inherently chiral benzodiazocines based on chiral phosphoric acid (CPA)-catalyzed condensation^11^ (Fig. 1B) and Yang described the dimerization of 2-acylbenzoisocyanates.^12^ Additionally, Yang utilized CPAs in the asymmetric synthesis of dihydrotribenzoazocines *via* (dynamic) kinetic resolution,^13^ and Miller disclosed enantioselective iminophosphorane-catalyzed cyclization affording inherently chiral benzodiazocinones.^14^ Besides tetraphenylenes and N-containing saddle-shaped molecules, the corresponding O-heterocycles have recently gained considerable attention.^8^ In 2024, Hao and Jiang developed an oxidative NHC catalytic formal (5 + 3) annulation strategy to produce inherently chiral eight-membered lactones (Fig. 1B).^15^ As already mentioned, NHC catalysis represents a powerful methodology for the construction of inherently chiral molecules, as well as related planar chiral macrocycles containing cyclophanes and ferrocenes.^16^ Very recently, Liu and Yan reported the construction of another inherently chiral eight-membered O-heterocycles through a cross-[4 + 4] cycloaddition reaction in the presence of quinine-derived catalyst and DBU.^17^

Inspired by recent advances in the preparation of inherently chiral molecules containing oxygen and our previous experience with NHC-mediated oxidative esterification,^18^ we aimed to develop a method for synthesizing inherently chiral eight-membered lactones through an oxidative NHC-catalyzed process (Fig. 1C). During the manuscript preparation, Chi, Zhang and Yang disclosed synthesis of proposed lactones, utilizing different catalytic system.^19^

## Results and discussion

### Optimization of reaction conditions

At the outset of the study, we investigated the configurational stability (racemization barrier) of ester 2a using density functional theory (DFT) calculations. To our satisfaction, calculations (at B3LYP/TZVP level of theory, for more information, refer to the SI) revealed a racemization barrier of approximately 28 kcal mol^−1^. We subsequently developed a versatile two-step synthesis of aldehyde 1a from commercially available 2-phenylphenol *via* borylation followed by Suzuki coupling, affording 1a in high yield (on up to a 5 g scale). Notably, mixing 1a with an achiral NHC precursor (*pre*-C1), an oxidant (Kharasch reagent, 3,3′,5,5′-tetra-*tert*-butyldiphenoquinone, DQ), and a base (cesium carbonate) resulted in the formation of the expected product 2a in high yield (73%) as a racemic mixture (Table 1, entry 1) of chiral HPLC-separable enantiomers (for more details, please refer to the SI). Replacing the achiral precursor with chiral Bode catalyst 1 (*pre*-C2) led to the formation of an enantioenriched product (32 : 68 er, entry 2) in an excellent yield (94%). Building on these proof-of-concept experiments, we aimed to improve the yield and enantioselectivity of the model reaction by varying of chiral precursors, solvents, oxidants, bases, and other reaction parameters (for a full optimization survey, please refer to the SI). A significant improvement in enantiopurity (13 : 87 er) was observed in the model reaction catalyzed by nitro-substituted Bode catalyst (*pre*-C3).^20^ In contrast, no improvement in enantioselectivity was achieved using more sterically hindered morpholine-backbone-based precursors. For instance, the camphor-derived precursor (*pre*-C4) afforded a product with a low optical purity (32 : 68 er). Encouragingly, significant improvement of enantioselectivity was achieved with an NHC precursor derived from l-valine (*pre*-C5, entry 5). The corresponding ester 2a was isolated in high yield (68%) and high enantiomeric excess (80 : 20 er). To our delight, the model reaction catalyzed with l-phenylalanine-derived precursor (*pre*-C6) proved even more efficient in terms of enantioselectivity, yielding product 2a with a high enantiomeric ratio (92 : 8 er). Further optimization of the model reaction catalyzed by *pre*-C6 revealed that the reaction is tolerant to some changes of bases or solvents. For example, substituting cesium carbonate with rubidium carbonate led to the formation of product 2a in nearly quantitative yield with minimal drop of optical purity (entry 7). The model reaction was also performed in the presence of tertiary amines such as DIPEA or DBU (entries 8 and 9), though both cases resulted in significantly lower yields and enantioselectivity. Upon further optimisation, we identified chlorinated solvents as suitable for this transformation. For instance, the reaction conducted in 1,2-dichloroethane (1,2-DCE, entry 10) provided the expected product 2a in nearly quantitative yield (98%) and high enantiopurity (93 : 7 er). Using other solvents such as toluene or ethyl acetate (entries 11 and 12) did not provide better outcomes. Similarly, alternative oxidants to DQ (TEMPO, MnO_2_) tested proved unsuitable (entries 13, 14). Notably, the use of these alternative oxidants resulted in a significant decrease in enantiopurity. A similar decrease in optical purity was observed when the catalyst loading was reduced to 10 mol% (entry 15). However, lowering reaction temperature to 0 °C (entry 17) improved the optical purity to 96 : 4 er, with high isolated yield (89%). Finally, performing the model reaction at 10 °C (entry 16) furnished the desired product in nearly quantitative yield (98%) with a high enantiomeric excess (97 : 3 er).

**Table 1:** Optimization of conditions for the model reaction

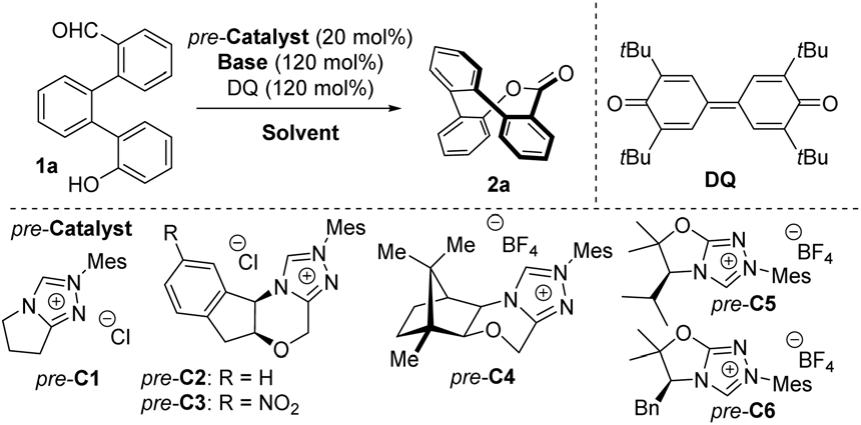						
Entry^a^	*pre*-C	Base	Solvent	Time	Yield^b^ (2a, %)	er^c^ (2a)
1	*pre*-C1	Cs_2_CO_3_	DCM	20 min	73	50 : 50
2	*pre*-C2	Cs_2_CO_3_	DCM	7 h	94	32 : 68
3	*pre*-C3	Cs_2_CO_3_	DCM	1 h	70	13 : 87
4	*pre*-C4	Cs_2_CO_3_	DCM	72 h	85	32 : 68
5	*pre*-C5	Cs_2_CO_3_	DCM	7 h	68	80 : 20
6	*pre*-C6	Cs_2_CO_3_	DCM	1 h	71	92 : 8
7	*pre*-C6	Rb_2_CO_3_	DCM	2 h	98	91 : 9
8	*pre*-C6	DIPEA	DCM	24 h	75	85 : 15
9	*pre*-C6	DBU	DCM	1 h	49	90 : 10
10	*pre*-C6	Cs_2_CO_3_	1,2-DCE	30 min	98	93 : 7
11	*pre*-C6	Cs_2_CO_3_	Toluene	15 min	67	85 : 15
12	*pre*-C6	Cs_2_CO_3_	EtOAc	5 min	93	86 : 14
13^d^	*pre*-C6	Cs_2_CO_3_	1,2-DCE	48 h	19	62 : 38
14^e^	*pre*-C6	Cs_2_CO_3_	1,2-DCE	72 h	11	49 : 51
15^f^	*pre*-C6	Cs_2_CO_3_	1,2-DCE	45 min	97	72 : 28
16^g^	*pre*-C6	Cs_2_CO_3_	1,2-DCE	30 h	98	97 : 3
17^h^	*pre*-C6	Cs_2_CO_3_	1,2-DCE	48 h	89	96 : 4

a Reactions were conducted with 1a (0.10 mmol), selected base (0.12 mmol), DQ (0.12 mmol) and selected pre catalyst (20 mol%) in selected solvent (2.0 ml) at room temperature (∼21 °C).

b Isolated yield after column chromatography.

c Determined by chiral HPLC analysis.

d TEMPO was used as an oxidant.

e MnO_2_ was used as an oxidant.

f 10 mol% of *pre*-C6 was used.

g Reaction was conducted at 10 °C.

h Reaction was conducted at 0 °C.

### Reaction scope

**Scheme 1:**
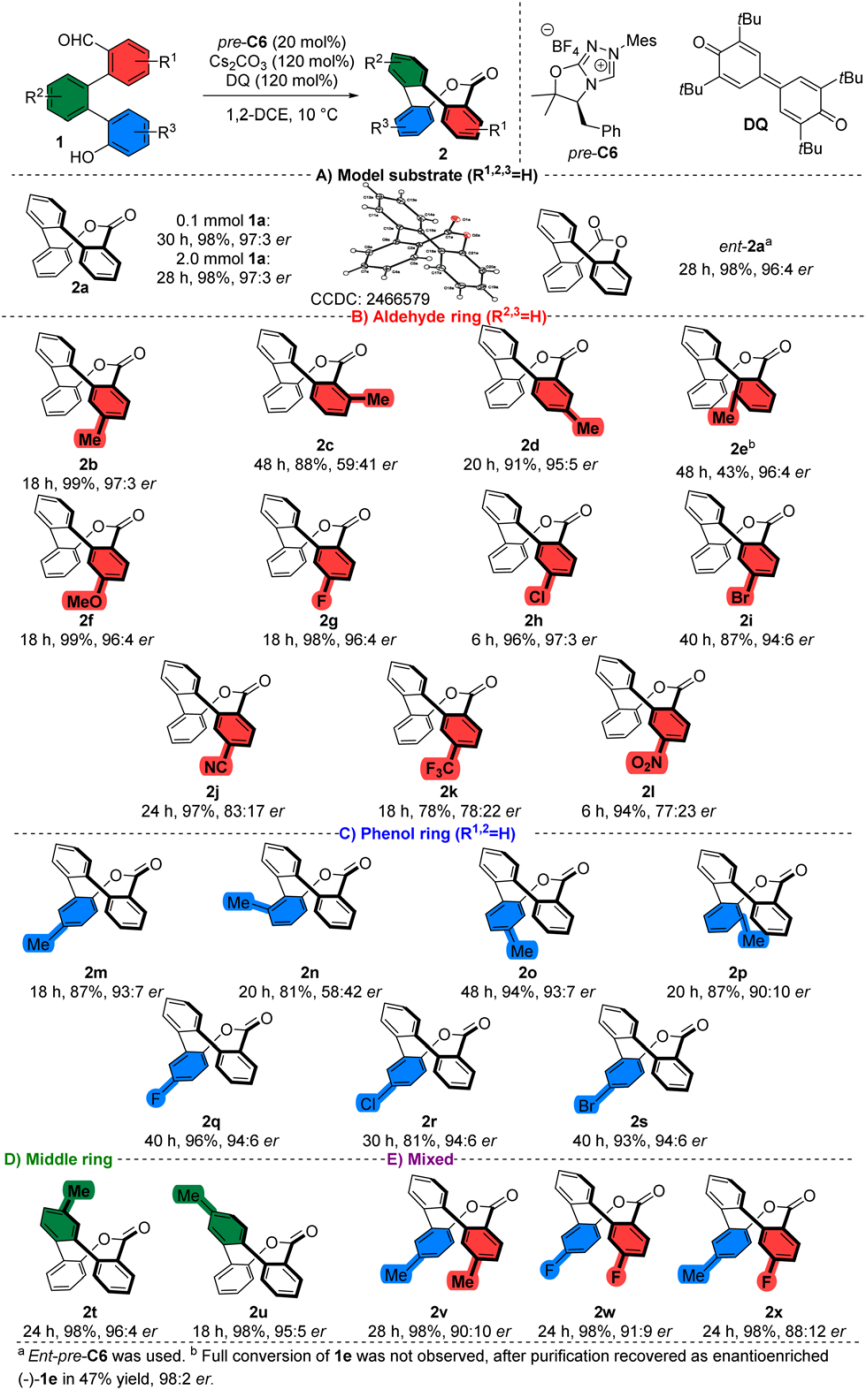
Substrate scope.

After optimizing the reaction conditions, we successfully scaled up the model reaction to a 2.0 mmol scale of 1a, affording 2a in nearly quantitative yield without any deviation in stereochemical outcome (Scheme 1A). Notably, the highly enantioenriched compound was suitable for X-ray crystallographic analysis, enabling us to confirm its structure and absolute configuration. The model reaction performed with the opposite enantiomer of the chiral catalyst (*ent-pre*-C6) yielded the enantiomeric product *ent*-2a in nearly quantitative yield (98%) and with high enantiopurity (96 : 4 er).

Subsequently, we examined the influence of substitution on the aldehydic aromatic ring of 1 (Scheme 1B). No significant changes in yield or stereochemical outcome were observed for methyl-derived products 2b and 2d bearing substituents in the *para* or *meta* positions relative to the ester group. Furthermore, shifting of the methyl substituent to the *ortho* position resulted in significantly diminished enantiocontrol (59 : 41 er) for product 2c, which we attribute to steric hindrance. Interestingly, we did not observe complete consumption of the starting material in the organocatalytic esterification of the starting material 1e, bearing a methyl substituent in *ortho*-position relative to the middle phenyl ring. Together with the expected product 2e, which was isolated in moderate yield (43%) with a high level of enantiopurity (96 : 4 er), we recovered the starting aldehyde 1e in 47% yield. Recovered aldehyde revealed high enantiopurity (98 : 2 er) by chiral HPLC, which can be attributed to the kinetic resolution of atropoisomeric starting aldehyde 1e. Continuing with other starting materials substituted with an electron-donating group (EDG) in the *para* position relative to the aldehyde group did not show any inconsistency in yield or enantioselectivity. For instance, the methoxy-substituted product 2f was obtained in nearly quantitative yield (99%) with high enantiomeric excess (96 : 4 er). Similarly, halogen-containing substrates, representing weak electron-withdrawing groups (EWGs), afforded the expected products without significantly affecting enantioselectivity or yield. In contrast, using a strongly EWG-substituted starting material resulted in a slight decrease in enantiomeric excess, albeit without significantly affecting the yield. For example, the nitrile-derived product 2j was obtained in nearly quantitative yield with moderate-to-high enantioselectivity (83 : 17 er).

To further assess our method (Scheme 1C), we introduced derivatives bearing a substitution on the phenol aromatic ring. Reaction of methyl-substituted starting material 1m, which bears substituent in the *para* position relative to the hydroxy group, resulted in the formation of the expected product 2m in high yield (87%) with high enantiopurity (93 : 7 er). *Ortho*-methyl substituted (with respect to the middle phenyl ring) product 2n was isolated with significantly diminished enantiopurity (58 : 42 er). Notably, we isolated starting material 1n as a mixture of rotamers (for more details, refer to the SI). We propose that non-restricted bond rotation between aromatic rings is crucial for achieving a high level of enantiodiscrimination. Other expected products 2q–s were isolated with high enantiomeric purities (91 : 9–96 : 4 er) and high-to-excellent isolated yields (over 81%), including examples substituted with both EDGs and EWGs.

Moreover, products 2t, 2u bearing methyl substitution on the central ring (Scheme 1D) in both relevant positions were obtained in nearly quantitative yield with high enantiomeric purities (over 95 : 5 er).

Additionally, we tested the scope of the method using starting materials substituted on both the aldehydic and phenolic rings (Scheme 1E). As a result, we observed the formation of expected products 2v–x with slightly decreased enantiocontrol compared to the monosubstituted derivatives. Despite this, the corresponding products 2v–x were isolated in nearly quantitative yield with high enantioselectivity. For instance, difluoro derivative 2w was obtained in a quantitative amount with the high enantiomeric ratio (91 : 9 er).

### Reaction mechanism

**Scheme 2:**
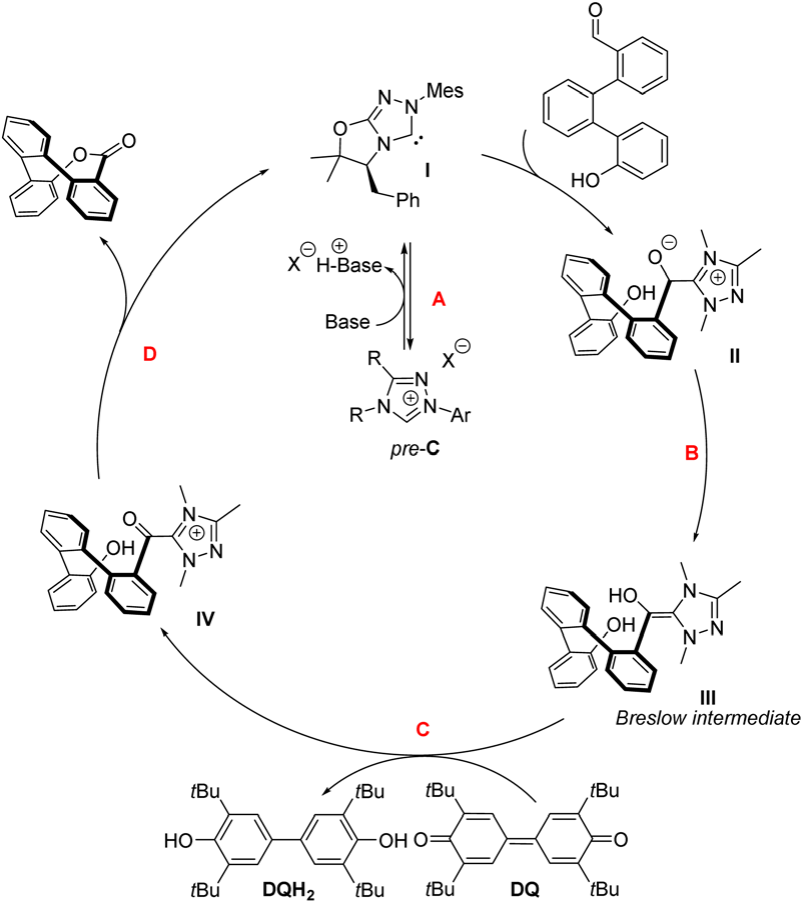
Proposed reaction mechanism.

Moreover, we investigate the origin of stereocontrol using DFT calculations. In line with several literature reports, we propose a possible catalytic cycle (Scheme 2). Briefly, chiral carbene I is generated by base-mediated deprotonation of the corresponding azolium salt (*pre*-C6). Then, the nucleophilic carbene attacks the carbonyl carbon of the aldehyde (step A), yielding a tetrahedral intermediate II. This intermediate undergoes a 1,2-C-to-O proton shift (step B), generating a Breslow intermediate (III). In the presence of an external oxidant (DQ), the Breslow intermediate is irreversibly oxidized (step C) to form an acyl azolium intermediate (IV). The resulting acyl azolium is electrophilic at the carbonyl carbon and thus undergoes intermolecular acyl substitution (step D) with phenol, regenerating the carbene and furnishing product 2.

**Fig. 2:**
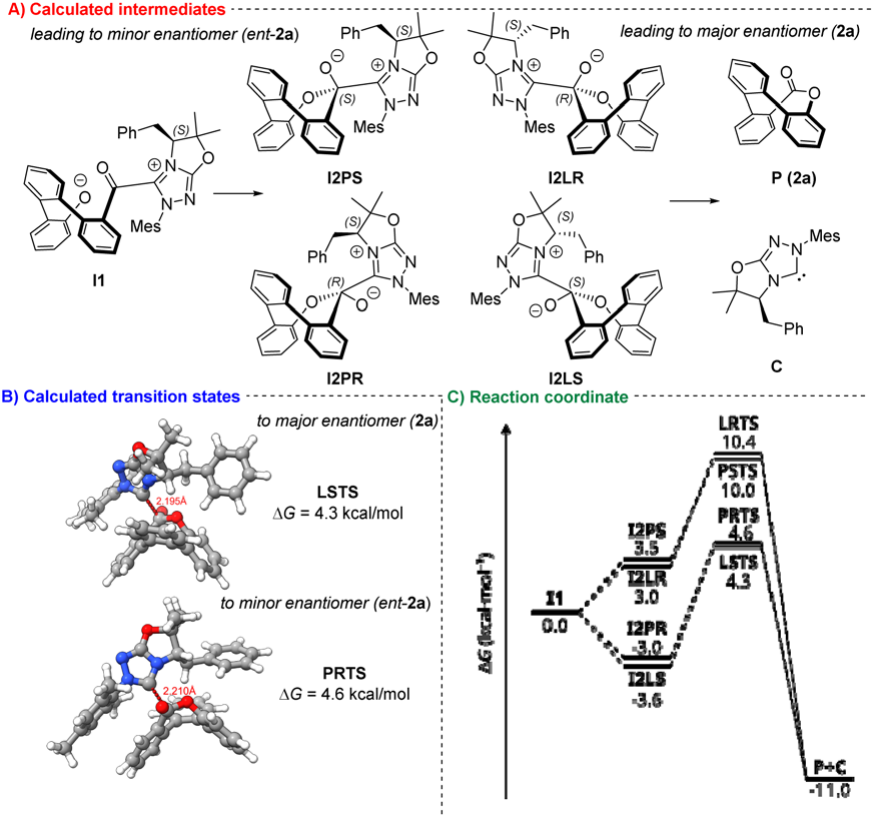
DFT calculations. (A) Calculated intermediates. (B) Calculated transition states. (C) Reaction coordinate.

Based on the proposed reaction mechanism, we postulate intermolecular acyl substitution (step D) as the key step for enantiodiscrimination (Fig. 2). Our calculations (at B3LYP(D3BJ)/def2-TVZP level of theory, for more information, refer to the SI) suggest that intramolecular attack of bare phenolate anion (formed by deprotonation of IV) to the acyl azolium is barrierless (we found no stationary point in a relaxed surface scan of the bond connecting phenolate oxygen to the acyl azolium carbon). This results in I2P and I2L series of intermediates, which can undergo elimination of the NHC catalyst C producing the saddle-shaped ester. The reaction scheme shows little preference for reaction path, as the energetic differences between intermediates I2PR and I2LS (0.6 kcal mol^−1^) and transition states PRTS and LSTS (0.3 kcal mol^−1^) fall below the typical accuracy of DFT methods.

Additionally, we reveal significant differences in enantiocontrol in comparison to Chi, Zhang, and Yang methodology.^19^ With our optimized catalytic system, we did not observe the inversion of the enantiocontrol by changing the nature of the base (Table 1, entries 6 *vs.* 9). We hypothesize that the role of *N*-aryl substituent of the triazol ring of the carbene is an important factor, which influences the basicity of the carbene.^21^ We believe electron-donating carbenes (such as *N*-Mes-substituted catalysts) represent a weaker leaving group in the carbene elimination reaction as the last step of the esterification reaction, which allows for control of enantioselectivity by catalyst choice in a predictable way.

## Conclusions

In summary, we have developed a highly efficient and versatile methodology for enantioselective lactonization, providing straightforward and adaptable access to unique chiral saddle-shaped lactones. This operationally simple and highly enantioselective strategy highlights the utility of amino acid-derived chiral carbenes as organocatalysts. Furthermore, the feasibility of the developed method was demonstrated by its good functional group tolerance and scalability. In addition, we elucidated the origin of enantioinduction during the intermolecular acyl substitution step using DFT calculations. Moving forward, our ongoing research will focus on the synthesis of inherently chiral molecules *via* organocatalytic reactions and the exploration of their diverse applications.

## Author contributions

V. D. conceived the concept, designed project and performed the synthesis. A. K. performed DFT calculations. I. C. performed X-ray analysis. J. V. directed the project. V. D., and J. V. wrote the manuscript. All authors have given approval to the final version of the manuscript.

## Conflicts of interest

There are no conflicts to declare.

## Supplementary Material

SC-016-D5SC05037E-s001

SC-016-D5SC05037E-s002

SC-016-D5SC05037E-s003

## Data Availability

FAIR data for this article, including the NMR dataset, is available at the figshare repository at https://doi.org/10.6084/m9.figshare.29487803. CCDC 2466579 (2a) contains the supplementary crystallographic data for this paper.^22^ Supplementary information: The data supporting this article have been included as part of the SI. See DOI: https://doi.org/10.1039/d5sc05037e.
