# Supplementary material for: Enantioselective lactonization catalyzed by chiral N-heterocyclic carbenes enables access to inherently chiral eight-membered lactones
Source: Chem Sci. 2025 Aug 25;16(37):17369–73. doi: 10.1039/d5sc05037e (PMC12400956; doi:10.1039/d5sc05037e)
Supplement: SC-016-D5SC05037E-s001 [file SC-016-D5SC05037E-s001.pdf]

Supplementary Information (SI) for Chemical Science.
This journal is © The Royal Society of Chemistry 2025

Enantioselective Lactonization Catalyzed by Chiral *N*-Heterocyclic Carbenes Enables Access to Inherently Chiral Eight-Membered Lactones

Vojtěch Dočekal,[1] Adam Kurčina,[1] Ivana Císařová[2] and Jan Veselý[1*]*

[1] Department of Organic Chemistry, Faculty of Science, Charles University, Hlavova 2030/8, 128 00 Prague 2, Czech Republic, e-mail: vojtech.docekal@natur.cuni.cz, jan.vesely@natur.cuni.cz
[2] Department of Inorganic Chemistry, Faculty of Science, Charles University, Hlavova 2030/8, 128 00 Prague 2, Czech Republic

Supplementary Information

Table of contents

Table of contents **2**

General **3**

Starting materials **3**

General procedure for preparation for Suzuki coupling *3*

Characterization data of starting materials *4*

Organocatalytic esterification reaction **12**

Complete reaction condition optimization survey *12*

General procedure for organocatalytic esterification *17*

Characterization data of products *17*

Unsuccessful starting material derivatization and follow-up transformations **26**

Crystallographic data **27**

Computational methods **29**

XYZ of stationary points *31*

NMR spectra **48**

Chiral HPLC **111**

References **137**

General

Chemicals and solvents were purchased from commercial suppliers and purified using standard techniques. Thin-layer chromatography (TLC) was performed on silica gel plates Merck 60 F_{254}. The compounds were visualized by irradiation with UV light and/or by treatment with a phosphomolybdic acid (AMC) solution followed by heating. Column chromatography was performed on silica gel Fluka (40-63 µm) or SiliCycle-SiliaFlash P60 (particle size: 40-63 µm, pore diameter: 60 Å. ^{1}H, ^{13}C NMR, and ^{19}F spectra were recorded with Bruker AVANCE III 400. Chemical shifts for protons are given in δ relative to tetramethylsilane (TMS) and referenced to residual protium in the NMR solvent (chloroform-*d*: δ_H = 7.26 ppm). Chemical shifts for carbon are referenced to the carbon of the NMR solvent (chloroform-*d*: δ_C = 77.16 ppm). IR DRIFT spectra were recorded on a Nicolet AVATAR 370 FT-IR [1]. Chiral HPLC was performed on a LC20AD Shimadzu liquid chromatograph with an SPD-M20A diode array detector with AD, IA, IC, and OD-H Daicel Chiralpak® columns. For chiral HPLC, the samples were prepared by dissolving them in $CHCl_3$/*i*-PrOH (1:1, *v/v*). Chloroform eluted as fist first eluting signal in all samples. Optical rotations were measured on an AU-Tomatica polarimeter, Autopol III. Specific optical rotations are expressed as concentration, *c* [g/100 ml]. The samples were prepared by dissolving them in solvent specified solvent for each compound. All melting points were measured on a Büchi melting point B-545 apparatus, in an open glass capillary. All values are uncorrected. High-resolution mass spectra were recorded on an LCQ Fleet spectrometer with a Bruker Compact QTOF-MS controlled by Compass 1.9 Control software. The monoisotopic mass values were calculated using Data analysis software v 4.4. The analysis was conducted in the positive ion mode at a scan range from *m*/*z* 50 to 1000, using nitrogen as the nebulizer gas at a pressure of 4 psi and a flow of 3 l/min for the dry gas. The capillary voltage and temperature were set at 4500 V and 220 °C, respectively. For HRMS, the samples were prepared by dissolving them in methanol.

Starting materials

General procedure for preparation for Suzuki coupling

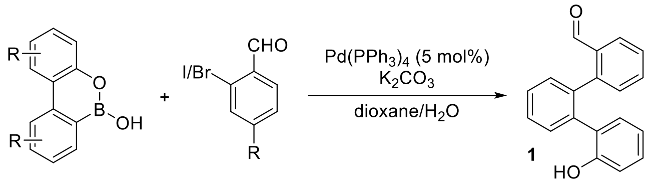

The round-bottom flask was charged with corresponding oxaborininol (1.0 equiv.), corresponding 2-halobenzaldehyde (1.5 equiv., in the case of liquid aldehydes, added them after dilution with solvent), $Pd(Ph_3P)_4$ (0.05 equiv.), K_2CO_3 (2.0 equiv.). The flask was degassed and refilled with argon. Then, 1,4-dioxane (120 ml per 30 mmol of oxaborininol) and water (40 ml per 30 mmol of oxaborininol) were added, and the reaction flask was degassed again. Reaction mixture was heated up to 85 °C (oil bath) and left to stir overnight at this temperature. Once the starting oxaborininol was no longer detected by thin-layer chromatography (TLC) or NMR, the mixture was diluted with saturated solution of ammonium chloride (100 ml per 30 mmol of oxaborininol) and EtOAc (100 ml per 30 mmol of oxaborininol). Once the organic phase was separated, the water phase was extracted with EtOAc (3×100 ml, per 30 mmol of oxaborininol). The organic phases were collected, washed with brine (1×50 ml, per 30 mmol of oxaborininol) and dried under anhydrous $MgSO_4$. After filtration of the solid, the filtrate was concentrated under reduced pressure. The crude product was purified by column chromatography (eluting by hexane/EtOAc mixtures).

Characterization data of starting materials

2''-Hydroxy-[1,1':2',1''-terphenyl]-2-carbaldehyde (1a)

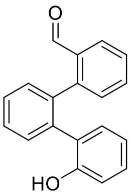

The title compound was synthesized according to the general procedure with 6*H*-dibenzo[*c*,*e*][1,2]oxaborinin-6-ol[1] (5.7 g, 28.9 mmol) and 2-bromobenzaldehyde (8.0 g, 43.3 mmol). The crude product was purified by column chromatography (hexane/EtOAc, 8:1), affording **1a** (6.4 g, 81%) as a white amorphous solid.

Notes: If the product purity is insufficient after column chromatography, it can be further purified by trituration with a minimal amount of toluene (using an ultrasound bath), with only a minimal loss in yield.

We did not observe hemiacetal form as documented by 2D NMR spectroscopy, which revealed the presence of isolated aromatic systems (in DMSO and DCM). For 2D spectra, please refer to the section NMR spectra.

^{1}H NMR (400 MHz, dimethyl sulfoxide-*d*$_6$): δ 9.77 (s, 1H), 9.22 (s, 1H), 7.71 (dd, *J* = 7.8, 1.5 Hz, 1H), 7.53 – 7.41 (m, 3H), 7.36 (ddd, *J* = 17.0, 7.4, 1.7 Hz, 3H), 7.18 (d, *J* = 7.6 Hz, 1H), 7.02 – 6.93 (m, 1H), 6.90 (dd, *J* = 7.7, 1.8 Hz, 1H), 6.68 – 6.54 (m, 2H) ppm. ^{13}C{^{1}H} NMR (101 MHz, dimethyl sulfoxide-*d*$_6$): δ 191.8, 154.1, 145.57, 138.7, 137.3, 133.0, 132.8, 131.5, 131.2, 130.84, 130.78, 128.5, 127.9, 127.3, 127.2, 126.9, 125.8, 118.6, 115.2 ppm. ^{1}H NMR (400 MHz, dichloromethane-*d*$_2$): δ 9.83 (s, 1H), 7.78 (dd, *J* = 7.7, 1.5 Hz, 1H), 7.58 – 7.42 (m, 4H), 7.41 – 7.34 (m, 2H), 7.23 (d, *J* = 7.6 Hz, 1H), 7.07 (td, *J* = 7.7, 1.7 Hz, 1H), 6.96 (dd, *J* = 7.5, 1.7 Hz, 1H), 6.76 (td, *J* = 7.5, 1.2 Hz, 1H), 6.67 (dd, *J* = 8.1, 1.1 Hz, 1H), 5.59 (br s, 1H) ppm. ^{13}C{^{1}H} NMR (101 MHz, dichloromethane-*d*$_2$): δ 193.3, 153.3, 144.8, 138.9, 137.4, 134.2, 133.4, 132.1, 131.7, 131.5, 131.3, 129.4, 128.9, 128.6, 128.3, 128.0, 127.6, 120.5, 115.9 ppm. IR (ATR): ν = 3350, 1356 (O-H, phenol), 1678 (C=O, aldehyde) cm^{-1}. HRMS (ESI-) *m*/*z*: calcd. for $C_{15}H_{13}O_2$ [M - H]$^-$: 273.0910, found: 273.09111.

2''-Hydroxy-5-methyl-[1,1':2',1''-terphenyl]-2-carbaldehyde (1b)

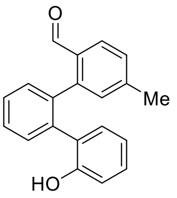

The title compound was synthesized according to the general procedure with 6*H*-dibenzo[*c*,*e*][1,2]oxaborinin-6-ol[1] (500 mg, 2.55 mmol) and 2-bromo-4-methylbenzaldehyde (762 mg, 3.83 mmol). The crude product was purified by column chromatography (hexane/EtOAc, 9:1-8:1), affording **1b** (565 mg, 77%) as a white amorphous solid.

^{1}H NMR (400 MHz, dimethyl sulfoxide-*d*$_6$): δ 9.69 (s, 1H), 9.24 (s, 1H), 7.60 (d, *J* = 7.9 Hz, 1H), 7.45 (dtd, *J* = 17.7, 7.3, 1.6 Hz, 2H), 7.35 (ddd, *J* = 17.0, 7.3, 1.7 Hz, 2H), 7.21 – 7.14 (m, 1H), 7.05 (s, 1H), 6.97 (td, *J* = 7.7, 1.7 Hz, 1H), 6.86 (dd, *J* = 7.6, 1.8 Hz, 1H), 6.70 – 6.56 (m, 2H), 2.26 (s, 3H) ppm. ^{13}C{^{1}H} NMR (101 MHz, dimethyl sulfoxide-*d*$_6$): δ 191.3, 154.2, 145.6, 143.2, 138.7, 137.3, 132.0, 131.2, 130.8 (2C), 130.7, 128.5, 128.1, 127.8, 127.2, 126.9, 125.9, 118.5, 115.2, 21.2 ppm. IR (ATR): ν = 3386, 1346 (O-H, phenol), 1685 (C=O, aldehyde) cm^{-1}. HRMS (ESI-) *m*/*z*: calcd. for $C_{20}H_{15}O_2$ [M - H]$^-$: 287.1078, found: 287.1074.

2''-Hydroxy-3-methyl-[1,1':2',1''-terphenyl]-2-carbaldehyde (1c)

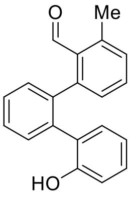

The title compound was synthesized according to the general procedure with 6*H*-dibenzo[*c*,*e*][1,2]oxaborinin-6-ol[1] (150 mg, 0.77 mmol) and 2-bromo-6-methylbenzaldehyde (229 mg, 1.15 mmol). The crude product was purified by column chromatography (hexane/EtOAc, 10:1-7:1), affording **1c** (150 mg, 68%) as a white amorphous solid.

^{1}H NMR (400 MHz, dichloromethane-*d*$_2$): δ 10.14 (s, 1H), 7.53 – 7.40 (m, 3H), 7.26 (t, *J* = 7.6 Hz, 2H), 7.14 (dt, *J* = 7.6, 0.9 Hz, 1H), 7.09 (ddd, *J* = 8.1, 7.4, 1.7 Hz, 1H), 7.00 (dd, *J* = 11.4, 7.4 Hz, 2H), 6.79 (td, *J* = 7.4, 1.2 Hz, 1H), 6.68 (dd, *J* = 8.1, 1.2 Hz, 1H), 5.89

(br s, 1H), 2.56 (s, 3H) ppm. $^{13}C\{^1H\}$ NMR (101 MHz, dichloromethane-d_2): δ 195.3, 153.5, 145.1, 140.7, 140.3, 137.1, 133.2, 132.2, 131.6, 131.5, 131.4, 131.1, 130.2, 129.3, 128.5, 128.2, 127.8, 120.3, 115.9, 20.7 ppm. IR (ATR): ν = 3292, 1361 (O-H, phenol), 1674 (C=O, aldehyde) cm^{-1}. HRMS (ESI+) *m/z*: calcd. for $C_{20}H_{15}O$ $[M + H – H_2O]^+$: 271.1117, found: 271.1123.

2''-Hydroxy-4-methyl-[1,1':2',1''-terphenyl]-2-carbaldehyde (1d)

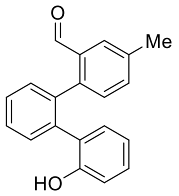

The title compound was synthesized according to the general procedure with 6*H*-dibenzo[*c*,*e*][1,2]oxaborinin-6-ol[1] (500 mg, 2.55 mmol) and 2-bromo-5-methylbenzaldehyde (762 mg, 1.15 mmol). The crude product was purified by column chromatography (hexane/EtOAc, 10:1-7:1), affording **1d** (543 mg, 74%) as a white amorphous solid.

^1H NMR (400 MHz, dichloromethane-d_2): δ 9.80 (s, 1H), 7.59 (d, *J* = 1.9 Hz, 1H), 7.54 – 7.41 (m, 3H), 7.38 – 7.31 (m, 1H), 7.28 (dd, *J* = 7.8, 1.9 Hz, 1H), 7.15 – 7.04 (m, 2H), 6.97 (dd, *J* = 7.6, 1.7 Hz, 1H), 6.77 (td, *J* = 7.4, 1.2 Hz, 1H), 6.68 (dd, *J* = 8.1, 1.2 Hz, 1H), 5.49 (br s, 1H), 2.36 (s, 3H) ppm. $^{13}C\{^1H\}$ NMR (101 MHz, dichloromethane-d_2): δ 193.4, 153.3, 141.8, 139.0, 138.1, 137.3, 134.3, 134.0, 132.0, 131.72, 131.68, 131.4 (2C), 129.3, 128.7, 128.3, 127.7, 120.5, 115.9, 21.1 ppm. IR (ATR): ν = 3267, 1377 (O-H, phenol), 1666 (C=O, aldehyde) cm^{-1}. HRMS (ESI-) *m/z*: calcd. for $C_{20}H_{15}O$ $[M + H – H_2O]^+$: 271.1117, found: 271.1122.

2''-Hydroxy-6-methyl-[1,1':2',1''-terphenyl]-2-carbaldehyde (1e)

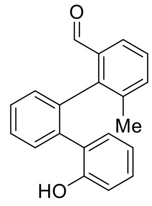

The title compound was synthesized according to the general procedure with modification of catalytic system using 6*H*-dibenzo[*c*,*e*][1,2]oxaborinin-6-ol[1] (150 mg, 0.77 mmol) and 2-bromo-3-methylbenzaldehyde (228 mg, 1.15 mmol). Instead of $Pd(PPh_3)_4$ and K_2CO_3 palladium(II) acetate (17 mg, 0.08 mmol, 0.1 equiv.), SPhos (63 mg, 0.15 mmol, 0.2 equiv.), and K_3PO_4 (490 mg, 2.31 mmol, 3.0 equiv.) were used and reaction mixture was heated up to 100 °C (oil bath) and left to stir overnight. The crude product was purified by column chromatography (hexane/EtOAc, 8:1-6:1), affording **1e** (123 mg, 55%) as a white amorphous solid.

Note: Under reaction conditions from general procedure only traces of product were detected.

^1H NMR (400 MHz, dichloromethane-d_2): δ 9.77 (d, *J* = 0.6 Hz, 1H), 7.64 (dd, *J* = 7.4, 1.5 Hz, 1H), 7.56 – 7.42 (m, 3H), 7.36 (ddd, *J* = 7.6, 1.5, 0.8 Hz, 1H), 7.33 – 7.23 (m, 2H), 7.05 (ddd, *J* = 8.0, 7.3, 1.7 Hz, 1H), 6.91 (dd, *J* = 7.6, 1.7 Hz, 1H), 6.72 (td, *J* = 7.5, 1.2 Hz, 1H), 6.68 (dd, *J* = 8.1, 1.2 Hz, 1H), 5.62 (s, 1H), 2.03 (s, 3H) ppm. $^{13}C\{^1H\}$ NMR (101 MHz, dichloromethane-d_2): δ 194.1, 153.3, 143.8, 138.2, 137.83, 137.80, 135.6, 134.7, 131.4, 131.12, 131.05, 129.3, 128.6, 128.3, 127.9, 127.7, 126.8, 120.2, 115.8, 20.0 ppm. IR (ATR): ν = 3390, 1344 (O-H, phenol), 1664 (C=O, aldehyde) cm^{-1}. HRMS (ESI+) *m/z*: calcd. for $C_{20}H_{15}O$ $[M + H – H_2O]^+$: 271.1117, found: 271.1123.

2''-Hydroxy-5-methoxy-[1,1':2',1''-terphenyl]-2-carbaldehyde (1f)

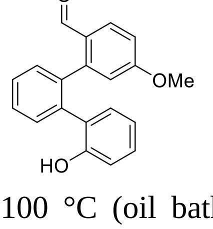

The title compound was synthesized according to the general procedure with modification of catalytic system using 6*H*-dibenzo[*c*,*e*][1,2]oxaborinin-6-ol[1] (500 mg, 2.55 mmol) and 2-bromo-4-methoxybenzaldehyde (824 mg, 3.83 mmol). Instead of $Pd(PPh_3)_4$ and K_2CO_3 palladium(II) acetate (57 mg, 0.26 mmol, 0.1 equiv.), SPhos (209 mg, 0.51 mmol, 0.2 equiv.), and K_3PO_4 (1624 mg, 7.65 mmol, 3.0 equiv.) were used and reaction mixture was heated up to 100 °C (oil bath) and left to stir overnight. The crude product was purified by column chromatography (hexane/EtOAc, 6:1-2:1), affording **1f** (333 mg, 43%) as a white amorphous solid.

Notes: Under reaction conditions from general procedure only traces of product were detected. Yield is lowered due to problematic separation with similarly eluting by-product.
^{1}H NMR (400 MHz, dimethyl sulfoxide-*d*$_6$): *δ* 9.62 (s, 1H), 9.26 (s, 1H), 7.68 (d, *J* = 8.7 Hz, 1H), 7.46 (dtd, *J* = 18.8, 7.3, 1.6 Hz, 2H), 7.36 (ddd, *J* = 9.0, 7.3, 1.6 Hz, 2H), 6.99 (td, *J* = 7.6, 1.8 Hz, 1H), 6.94 – 6.87 (m, 2H), 6.71 (d, *J* = 2.5 Hz, 1H), 6.70 – 6.58 (m, 2H), 3.70 (s, 3H) ppm. ^{13}C{^{1}H} NMR (101 MHz, dimethyl sulfoxide-*d*$_6$): *δ* 190.2, 162.4, 154.2, 147.9, 138.6, 137.1, 131.1, 130.8, 130.6, 128.5, 128.3, 127.9, 127.4, 126.8, 126.7, 118.5, 115.9, 115.2, 113.8, 55.4 ppm. IR (ATR): *ν* = 3307, 1350 (O-H, phenol), 1660 (C=O, aldehyde) cm^{-1}. HRMS (ESI-) *m*/*z*: calcd. for $C_{20}H_{15}O_2$ [M - H]$^-$: 303.1027, found: 303.1023.

5-Fluoro-2''-hydroxy-[1,1':2',1''-terphenyl]-2-carbaldehyde (1g)

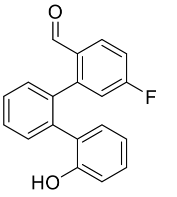

The title compound was synthesized according to the general procedure with 6*H*-dibenzo[*c*,*e*][1,2]oxaborinin-6-ol[1] (500 mg, 2.55 mmol) and 2-bromo-4-fluorobenzaldehyde (778 mg, 3.83 mmol). The crude product was purified by column chromatography (hexane/EtOAc, 8:1-3:1), affording **1g** (661 mg, 89%) as a white amorphous solid.
^{1}H NMR (400 MHz, dimethyl sulfoxide-*d*$_6$): *δ* 9.66 (s, 1H), 9.28 (s, 1H), 7.77 (dd, *J* = 8.7, 6.1 Hz, 1H), 7.49 (dtd, *J* = 21.5, 7.4, 1.4 Hz, 2H), 7.39 (dd, *J* = 7.5, 1.6 Hz, 2H), 7.22 (td, *J* = 8.6, 2.6 Hz, 1H), 7.05 – 6.93 (m, 3H), 6.71 – 6.61 (m, 2H) ppm. ^{13}C{^{1}H} NMR (101 MHz, dimethyl sulfoxide-*d*$_6$): *δ* 190.7, 164.6 (d, *J* = 252.6 Hz), 154.5, 148.9 (d, *J* = 9.6 Hz), 139.1, 136.5 (d, *J* = 1.6 Hz), 131.7, 131.3, 131.0, 130.5 (d, *J* = 2.3 Hz), 129.5 (d, *J* = 10.1 Hz), 129.3, 128.9, 127.5, 127.3, 119.2, 118.5 (d, *J* = 21.9 Hz), 115.7, 115.3 (d, *J* = 22.0 Hz) ppm. ^{19}F NMR (376 MHz, dimethyl sulfoxide-*d*$_6$): *δ* -105.59 (td, *J* = 9.1, 6.1 Hz, 1F) ppm. IR (ATR): *ν* = 3396, 1350 (O-H, phenol), 1674 (C=O, aldehyde) cm^{-1}. HRMS (ESI+) *m*/*z*: calcd. for $C_{19}H_{13}FNaO_2$ [M + Na]$^+$: 315.0792, found: 315.0792.

5-Chloro-2''-hydroxy-[1,1':2',1''-terphenyl]-2-carbaldehyde (1h)

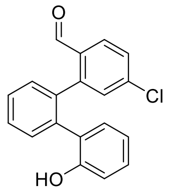

The title compound was synthesized according to the general procedure with 6*H*-dibenzo[*c*,*e*][1,2]oxaborinin-6-ol[1] (500 mg, 2.55 mmol) and 2-bromo-4-chlorobenzaldehyde (841 mg, 3.83 mmol). The crude product was purified by column chromatography (hexane/EtOAc, 8:1-3:1), affording **1h** (545 mg, 69%) as a white amorphous solid.
^{1}H NMR (400 MHz, dimethyl sulfoxide-*d*$_6$): *δ* 9.67 (s, 1H), 9.28 (s, 1H), 7.70 (d, *J* = 8.3 Hz, 1H), 7.55 – 7.35 (m, 5H), 7.25 (s, 1H), 7.06 – 6.90 (m, 2H), 6.73 – 6.58 (m, 2H) ppm. ^{13}C{^{1}H} NMR (101 MHz, dimethyl sulfoxide-*d*$_6$): *δ* 190.6, 154.0, 147.1, 138.7, 137.5, 135.8, 131.7, 131.2, 131.1, 130.8, 130.6, 128.8, 128.5, 127.8, 127.6, 127.1, 126.8, 118.7, 115.2 ppm. IR (ATR): *ν* = 3394, 1346 (O-H, phenol), 1697 (C=O, aldehyde) cm^{-1}. HRMS (ESI+) *m*/*z*: calcd. for $C_{19}H_{13}ClNaO_2$ [M + Na]$^+$: 331.0496, found: 331.0492.

5-Bromo-2''-hydroxy-[1,1':2',1''-terphenyl]-2-carbaldehyde (1i)

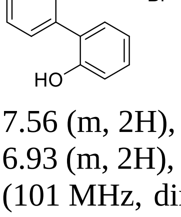

The title compound was synthesized according to the general procedure with 6*H*-dibenzo[*c*,*e*][1,2]oxaborinin-6-o[1] (500 mg, 2.55 mmol) and 4-bromo-2-iodobenzaldehyde (1190 mg, 3.83 mmol). The crude product was purified by column chromatography (hexane/EtOAc, 9:1-2:1), affording **1i** (510 mg, 57%) as a white amorphous solid.
^{1}H NMR (400 MHz, dimethyl sulfoxide-*d*$_6$): *δ* 9.67 (s, 1H), 9.27 (s, 1H), 7.64 – 7.56 (m, 2H), 7.50 (dtd, *J* = 22.1, 7.4, 1.6 Hz, 2H), 7.39 (ddd, *J* = 7.4, 3.7, 1.5 Hz, 3H), 7.05 – 6.93 (m, 2H), 6.69 (td, *J* = 7.4, 1.1 Hz, 1H), 6.63 (dd, *J* = 8.1, 1.1 Hz, 1H) ppm. ^{13}C{^{1}H} NMR (101 MHz, dimethyl sulfoxide-*d*$_6$): *δ* 190.8, 154.0, 147.1, 138.7, 135.7, 134.0, 132.0, 131.2, 130.8, 130.6, 130.5, 128.8, 128.5, 127.8, 127.1, 126.8, 126.7, 118.7, 115.2 ppm. IR (ATR): *ν* =

3392, 1344, (O-H, phenol), 1684 (C=O, aldehyde) cm^{-1}. HRMS (ESI+) *m*/*z*: calcd. for $C_{19}H_{13}BrNaO_2$ $[M + Na]^+$: 374.9991, found: 374.9990.

6-Formyl-2''-hydroxy-[1,1':2',1''-terphenyl]-3-carbonitrile (1j)

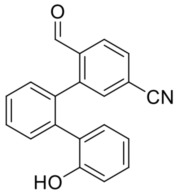

The title compound was synthesized according to the general procedure with 6*H*-dibenzo[*c*,*e*][1,2]oxaborinin-6-ol[1] (500 mg, 2.55 mmol) and 3-bromo-4-formylbenzonitrile (804 mg, 3.83 mmol). The crude product was purified by column chromatography (hexane/EtOAc, 6:1-4:1), affording **1j** (576 mg, 75%) as a white amorphous solid.

^{1}H NMR (400 MHz, dimethyl sulfoxide-*d*$_6$): δ 9.73 (s, 1H), 9.28 (s, 1H), 7.91 – 7.73 (m, 2H), 7.68 (s, 1H), 7.53 (dtd, *J* = 22.4, 7.4, 1.5 Hz, 2H), 7.43 (ddd, *J* = 13.3, 7.5, 1.5 Hz, 2H), 7.02 (ddt, *J* = 6.3, 4.5, 2.0 Hz, 2H), 6.71 (td, *J* = 7.5, 1.1 Hz, 1H), 6.60 (dd, *J* = 8.5, 1.1 Hz, 1H) ppm. ^{13}C{^{1}H} NMR (101 MHz, dimethyl sulfoxide-*d*$_6$): δ 191.1, 154.3, 146.2, 139.3, 136.2, 136.0, 135.9, 131.8, 131.4, 131.3, 131.0, 129.5, 129.3, 127.7, 127.1, 127.0, 119.4, 118.5, 115.7, 115.1 ppm. IR (ATR): ν = 3411, 1348 (O-H, phenol), 1682 (C=O, aldehyde), 2225 (CN, nitrile) cm^{-1}. HRMS (ESI+) *m*/*z*: calcd. for $C_{20}H_{13}NNaO_2$ $[M + Na]^+$: 322.0838, found: 322.0840.

2''-Hydroxy-5-(trifluoromethyl)-[1,1':2',1''-terphenyl]-2-carbaldehyde (1k)

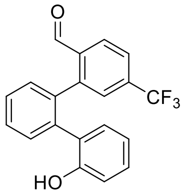

The title compound was synthesized according to the general procedure with 6*H*-dibenzo[*c*,*e*][1,2]oxaborinin-6-ol[1] (500 mg, 2.55 mmol) and 2-bromo-4-(trifluoromethyl)benzaldehyde (0.58 ml, 3.83 mmol). The crude product was purified by column chromatography (hexane/EtOAc, 9:1-6:1), affording **1k** (563 mg, 65%) as a white amorphous solid.

^{1}H NMR (400 MHz, dimethyl sulfoxide-*d*$_6$): δ 9.80 (s, 1H), 9.24 (s, 1H), 7.89 (d, *J* = 8.1 Hz, 1H), 7.72 (dd, *J* = 8.1, 1.9 Hz, 1H), 7.55 (td, *J* = 7.4, 1.6 Hz, 1H), 7.52 – 7.38 (m, 4H), 7.00 (t, *J* = 7.6 Hz, 2H), 6.69 (t, *J* = 7.4 Hz, 1H), 6.59 (d, *J* = 8.0 Hz, 1H) ppm. ^{13}C{^{1}H} NMR (101 MHz, dimethyl sulfoxide-*d*$_6$): δ 191.4, 154.3, 146.3, 139.3, 136.4, 136.3, 132.4 (q, *J* = 31.8 Hz), 131.7, 131.3, 131.2, 129.4, 129.2, 128.9 (q, *J* = 3.6 Hz), 127.6, 127.4, 127.2, 124.4 (q, *J* = 3.6 Hz), 124.0 (q, *J* = 273.0 Hz), 119.3, 115.6 ppm. ^{19}F NMR (376 MHz, dimethyl sulfoxide-*d*$_6$): δ -61.84 (s, 3F) ppm. IR (ATR): ν = 3410, 1333 (O-H, phenol), 1682 (C=O, aldehyde) cm^{-1}. HRMS (ESI-) *m*/*z*: calcd. for $C_{20}H_{12}F_3O_2$ $[M - H]^-$: 341.0795, found: 341.0791.

2''-Hydroxy-5-nitro-[1,1':2',1''-terphenyl]-2-carbaldehyde (1l)

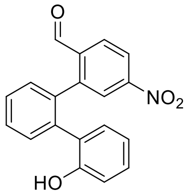

The title compound was synthesized according to the general procedure with 6*H*-dibenzo[*c*,*e*][1,2]oxaborinin-6-ol[1] (500 mg, 2.55 mmol) and 2-bromo-4-nitrobenzaldehyde (880 mg, 3.83 mmol). The crude product was purified by column chromatography (hexane/EtOAc, 4:1), affording **1l** (594 mg, 73%) as a white amorphous solid.

^{1}H NMR (400 MHz, dimethyl sulfoxide-*d*$_6$): δ 9.77 (s, 1H), 9.29 (s, 1H), 8.20 (dd, *J* = 8.6, 2.3 Hz, 1H), 8.13 – 7.75 (m, 2H), 7.61 – 7.41 (m, 4H), 7.09 – 6.97 (m, 2H), 6.71 (td, *J* = 7.4, 1.1 Hz, 1H), 6.58 (d, *J* = 8.0 Hz, 1H) ppm. ^{13}C{^{1}H} NMR (101 MHz, dimethyl sulfoxide-*d*$_6$): δ 190.4, 153.7, 149.1, 146.3, 138.8, 137.0, 135.4, 131.3, 130.8, 130.5, 129.2, 129.0, 127.6, 127.4, 126.4, 122.1, 119.0, 115.2 ppm. IR (ATR): ν = 3429, 1346 (O-H, phenol), 1674 (C=O, aldehyde), 1520 (N-O, nitro) cm^{-1}. HRMS (ESI-) *m*/*z*: calcd. for $C_{19}H_{12}NO_4$ $[M - H]^-$: 318.0772, found: 318.0770.

2''-Hydroxy-5''-methyl-[1,1':2',1''-terphenyl]-2-carbaldehyde (1m)

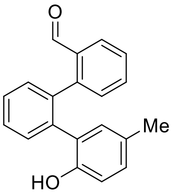

The title compound was synthesized according to the general procedure with 2-methyl-6*H*-dibenzo[*c*,*e*][1,2]oxaborinin-6-ol[1] (300 mg, 1.43 mmol) and 2-bromobenzaldehyde (392 mg, 2.14 mmol). The crude product was purified by column chromatography (hexane/EtOAc, 4:1), affording **1m** (351 mg, 85%) as a white amorphous solid.

^{1}H NMR (400 MHz, dimethyl sulfoxide-d_6): δ 9.76 (s, 1H), 8.95 (s, 1H), 7.72 (dd, J = 7.8, 1.4 Hz, 1H), 7.46 (dtd, J = 19.6, 7.4, 1.5 Hz, 3H), 7.40 – 7.34 (m, 2H), 7.32 (dd, J = 7.3, 1.6 Hz, 1H), 7.18 (d, J = 7.7 Hz, 1H), 6.77 (dd, J = 8.1, 2.3 Hz, 1H), 6.73 (d, J = 2.2 Hz, 1H), 2.06 (s, 3H) ppm. ^{13}C{^{1}H} NMR (101 MHz, dimethyl sulfoxide-d_6): δ 191.9, 151.8, 145.7, 138.8, 137.3, 133.0, 132.8, 131.6, 131.4, 130.8, 130.8, 129.0, 127.9, 127.2, 126.9, 126.81, 126.79, 125.8, 115.0, 20.0 ppm. IR (ATR): ν = 3408, 1333 (O-H, phenol), 1690 (C=O, aldehyde) cm^{-1}. HRMS (ESI+) *m*/*z*: calcd. for $C_{20}H_{16}NaO_2$ [M + Na]$^+$: 311.1043, found: 311.1042.

2''-Hydroxy-3''-methyl-[1,1':2',1''-terphenyl]-2-carbaldehyde (1n)

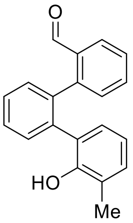

The title compound was synthesized according to the general procedure with 4-methyl-6*H*-dibenzo[*c*,*e*][1,2]oxaborinin-6-ol[1] (300 mg, 1.43 mmol) and 2-bromobenzaldehyde (392 mg, 2.14 mmol). The crude product was purified by column chromatography (hexane/EtOAc, 8:1-5:1), affording **1n** (334 mg, 81%) as a white amorphous solid.

^{1}H NMR (400 MHz, dichloromethane-d_2): δ 9.83 (s, 1H), 7.77 (dd, J = 7.6, 1.5 Hz, 1H), 7.58 – 7.42 (m, 4H), 7.38 (td, J = 7.5, 1.3 Hz, 2H), 7.25 – 7.19 (m, 1H), 6.99 – 6.92 (m, 1H), 6.81 – 6.72 (m, 1H), 6.65 (t, J = 7.5 Hz, 1H), 5.25 (br s, 1H), 2.09 (s, 3H) ppm. ^{13}C{^{1}H} NMR (101 MHz, dichloromethane-d_2): δ 192.9, 151.4, 144.0, 139.2, 137.2, 134.3, 133.3, 132.1, 131.6, 131.3 (2C), 130.6, 129.2, 129.0, 128.4, 128.0, 127.0, 124.6, 120.0, 16.1 ppm. IR (ATR): ν = 3500, 1321 (O-H, phenol), 1689 (C=O, aldehyde) cm^{-1}. HRMS (ESI+) *m*/*z*: calcd. for $C_{20}H_{15}O$ [M + H – H_2O]$^+$: 271.1117, found: 271.1123.

2''-Hydroxy-4''-methyl-[1,1':2',1''-terphenyl]-2-carbaldehyde (1o)

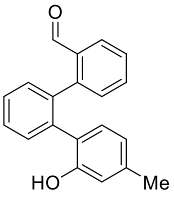

The title compound was synthesized according to the general procedure with 3-methyl-6*H*-dibenzo[*c*,*e*][1,2]oxaborinin-6-ol[1] (300 mg, 1.43 mmol) and 2-bromobenzaldehyde (392 mg, 2.14 mmol). The crude product was purified by column chromatography (hexane/EtOAc, 8:1-7:1), affording **1o** (273 mg, 66%) as a white amorphous solid.

^{1}H NMR (400 MHz, dichloromethane-d_2): δ 9.82 (s, 1H), 7.78 (dd, J = 7.7, 1.5 Hz, 1H), 7.55 – 7.41 (m, 4H), 7.41 – 7.34 (m, 2H), 7.23 (dd, J = 7.7, 1.3 Hz, 1H), 6.83 (d, J = 7.7 Hz, 1H), 6.58 (ddd, J = 7.7, 1.7, 0.8 Hz, 1H), 6.55 – 6.48 (m, 1H), 5.42 (s, 1H), 2.21 (s, 3H) ppm. ^{13}C{^{1}H} NMR (101 MHz, dichloromethane-d_2): δ 193.2, 153.0, 144.9, 139.7, 138.9, 137.5, 134.2, 133.4, 132.1, 131.53, 131.49, 131.46, 128.8, 128.5, 128.1, 127.9, 124.6, 121.4, 116.4, 21.2 ppm. IR (ATR): ν = 3421, 1402 (O-H, phenol), 1684 (C=O, aldehyde) cm^{-1}. HRMS (ESI+) *m*/*z*: calcd. for $C_{20}H_{15}O$ [M + H – H_2O]$^+$: 271.1117, found: 271.1118.

2''-Hydroxy-6''-methyl-[1,1':2',1''-terphenyl]-2-carbaldehyde (1p)

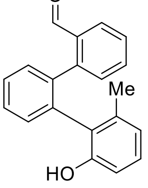

The title compound was synthesized according to the general procedure with 3-methyl-6*H*-dibenzo[*c*,*e*][1,2]oxaborinin-6-ol[1] (300 mg, 1.43 mmol) and 1-bromobenzaldehyde (392 mg, 2.14 mmol). The crude product was purified by column chromatography (hexane/EtOAc, 8:1-7:1), affording **1p** (286 mg, 69%) as a white amorphous solid.

Notes: The title compound was isolated as a mixture of rotamers (for thermal dependency, refer to the copies of NMR spectras).
Purity of compound is approximately 95% (based to NMR).
^{1}H NMR (400 MHz, dimethyl sulfoxide-*d*$_6$, 25 °C, major rotamer - H′, minor - H): *δ* 9.86 (s, 1H), 9.81 (s, 1H′), 9.29 (s, 1H), 9.05 (s, 1H′), 7.81 – 7.74 (m, 1H′+1H), 7.56 – 7.31 (m, 5H′+5H), 7.27 (d, *J* = 7.8 Hz, 1H′+2H), 7.05 (d, *J* = 7.5 Hz, 1H′), 6.90 (t, *J* = 7.8 Hz, 1H), 6.85 (t, *J* = 7.8 Hz, 1H′), 6.63 (d, *J* = 8.2 Hz, 1H), 6.58 (d, *J* = 7.5 Hz, 1H′), 6.46 (d, *J* = 7.6 Hz, 1H), 6.42 (d, *J* = 8.1 Hz, 1H′), 2.03 (s, 3H′), 1.69 (s, 3H) ppm. ^{1}H NMR (600 MHz, dimethyl sulfoxide-*d*$_6$, 80 °C): *δ* 9.85 (s, 1H), 8.76 (br s, 1H), 7.75 (dd, *J* = 7.7, 1.5 Hz, 1H), 7.49 (td, *J* = 7.5, 1.4 Hz, 1H), 7.44 (td, *J* = 7.5, 1.5 Hz, 1H), 7.40 (td, *J* = 7.5, 1.6 Hz, 1H), 7.38 – 7.31 (m, 2H), 7.26 (dd, *J* = 7.6, 1.4 Hz, 1H), 6.87 (t, *J* = 7.8 Hz, 1H), 6.59 – 6.42 (m, 2H), 1.97 (br s, 3H) ppm.^{13}C{^{1}H} NMR (101 MHz, dimethyl sulfoxide-*d*$_6$, *25 °C*, only major rotamer): *δ* 192.4, 154.0, 145.7, 137.8, 137.4, 136.6, 133.4, 132.5, 131.1, 130.9, 130.3, 128.0, 127.9, 127.3, 127.1, 126.6, 125.6, 120.2, 112.4, 20.4 ppm. IR (ATR): *ν* = 3311, 1336 (O-H, phenol), 1678 (C=O, aldehyde) cm^{-1}. HRMS (ESI+) *m*/*z*: calcd. for $C_{20}H_{15}O$ [M + H – H_2O]$^+$: 271.1117, found: 271.1125.

5''-Fluoro-2''-hydroxy-[1,1':2',1''-terphenyl]-2-carbaldehyde (1q)

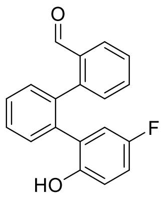

The title compound was synthesized according to the general procedure with 2-fluoro-6*H*-dibenzo[*c*,*e*][1,2]oxaborinin-6-ol[1] (300 mg, 1.40 mmol) and 2-bromobenzaldehyde (387 mg, 2.10 mmol). The crude product was purified by column chromatography (hexane/EtOAc, 9:1-6:1), affording **1q** (310 mg, 76%) as a colorless oil.

^{1}H NMR (400 MHz, dimethyl sulfoxide-*d*$_6$): *δ* 9.77 (s, 1H), 9.25 (s, 1H), 7.74 (dd, *J* = 7.8, 1.4 Hz, 1H), 7.54 – 7.44 (m, 3H), 7.38 (ddd, *J* = 18.5, 7.2, 1.9 Hz, 3H), 7.20 (dd, *J* = 7.6, 1.2 Hz, 1H), 6.85 – 6.74 (m, 2H), 6.60 (dd, *J* = 8.8, 4.8 Hz, 1H) ppm. ^{13}C{^{1}H} NMR (101 MHz, dimethyl sulfoxide-*d*$_6$): *δ* 191.9, 154.8 (d, *J* = 234.3 Hz), 150.5 (d, *J* = 1.8 Hz), 145.2, 137.5, 137.3, 133.1, 132.9, 131.5, 130.8, 130.7, 128.3 (d, *J* = 7.8 Hz), 128.0, 127.5, 127.3, 125.9, 117.2 (d, *J* = 22.9 Hz), 115.9 (d, *J* = 8.2 Hz), 114.8 (d, *J* = 22.5 Hz) ppm. ^{19}F NMR (376 MHz, dimethyl sulfoxide-*d*$_6$): *δ* -126.35 (td, *J* = 8.7, 4.8 Hz, 1F) ppm. IR (ATR): *ν* = 3244, 1302 (O-H, phenol), 1674 (C=O, aldehyde) cm^{-1}. HRMS (ESI+) *m*/*z*: calcd. for $C_{19}H_{13}FNaO_2$ [M + Na]$^+$: 315.0792, found: 315.0791.

5''-Chloro-2''-hydroxy-[1,1':2',1''-terphenyl]-2-carbaldehyde (1r)

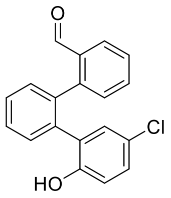

The title compound was synthesized according to the general procedure with 2-chloro-6*H*-dibenzo[*c*,*e*][1,2]oxaborinin-6-ol[2] (300 mg, 1.30 mmol) and 2-bromobenzaldehyde (361 mg, 1.95 mmol). The crude product was purified by column chromatography (hexane/EtOAc, 8:1-7:1), affording **1r** (300 mg, 75%) as a white amorphous solid.

^{1}H NMR (400 MHz, dichloromethane-*d*$_2$): *δ* 9.84 (s, 1H), 7.80 (dd, *J* = 7.6, 1.5 Hz, 1H), 7.57 – 7.46 (m, 3H), 7.46 – 7.39 (m, 2H), 7.38 – 7.30 (m, 1H), 7.23 – 7.16 (m, 1H), 7.03 (dd, *J* = 8.6, 2.6 Hz, 1H), 6.99 (d, *J* = 2.6 Hz, 1H), 6.61 (d, *J* = 8.6 Hz, 1H), 5.71 (br s, 1H) ppm. ^{13}C{^{1}H} NMR (101 MHz, dichloromethane-*d*$_2$): *δ* 193.6, 152.2, 143.9, 139.1, 135.9, 134.3, 133.5, 132.2, 131.5, 131.2 (2C), 131.1, 129.3, 129.1, 129.0, 128.7, 128.2, 125.0, 117.3 ppm. IR (ATR): *ν* = 3246, 1360 (O-H, phenol), 1678 (C=O, aldehyde) cm^{-1}. HRMS (ESI+) *m*/*z*: calcd. for $C_{19}H_{13}ClNaO_2$ [M + Na]$^+$: 331.0496, found: 331.0497.

5''-Bromo-2''-hydroxy-[1,1':2',1''-terphenyl]-2-carbaldehyde (1s)

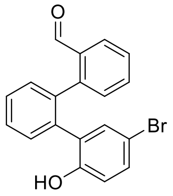

The title compound was synthesized according to the general procedure with 2-bromo-6*H*-dibenzo[*c*,*e*][1,2]oxaborinin-6-ol[3] (290 mg, 1.06 mmol) and 2-iodobenzaldehyde (367 mg, 1.58 mmol). The crude product was purified by column chromatography (hexane/EtOAc, 9:1-7:1), affording **1s** (100 mg, 27%) as a white foam.

^{1}H NMR (400 MHz, dichloromethane-d_2): δ 9.82 (s, 1H), 7.80 (dd, J = 7.7, 1.5 Hz, 1H), 7.56 – 7.46 (m, 3H), 7.46 – 7.38 (m, 2H), 7.38 – 7.29 (m, 1H), 7.24 – 7.18 (m, 1H), 7.18 – 7.08 (m, 2H), 6.57 (d, J = 8.6 Hz, 1H), 6.13 (br s, 1H). ppm. ^{13}C{^{1}H} NMR (101 MHz, dichloromethane-d_2): δ 193.9, 152.7, 144.2, 139.0, 136.1, 134.2, 134.0, 133.6, 132.2, 132.0, 131.4, 131.2, 129.8, 129.2, 128.9, 128.6, 128.2, 117.8, 112.1 ppm. IR (ATR): ν = 3246, 1356 (O-H, phenol), 1678 (C=O, aldehyde) cm^{-1}. HRMS (ESI+) *m*/*z*: calcd. for $C_{19}H_{13}BrNaO_2$ [M + Na]$^+$: 374.9990, found: 374.9987.

2''-Hydroxy-5'-methyl-[1,1':2',1''-terphenyl]-2-carbaldehyde (1t)

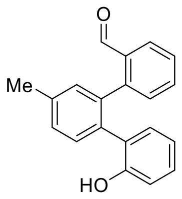

The title compound was synthesized according to the general procedure with 8-methyl-6*H*-dibenzo[*c*,*e*][1,2]oxaborinin-6-ol[2] (300 mg, 1.43 mmol) and 2-bromobenzaldehyde (0.25 ml, 2.14 mmol). The crude product was purified by column chromatography (hexane/EtOAc, 8:1-7:1), affording **1t** (324 mg, 79%) as a white amorphous solid.

^{1}H NMR (400 MHz, dimethyl sulfoxide-d_6): δ 9.77 (d, J = 0.9 Hz, 1H), 9.18 (s, 1H), 7.70 (dd, J = 7.8, 1.4 Hz, 1H), 7.48 (td, J = 7.5, 1.5 Hz, 1H), 7.39 – 7.33 (m, 1H), 7.32 – 7.24 (m, 2H), 7.19 (dd, J = 7.7, 1.3 Hz, 1H), 7.17 – 7.14 (m, 1H), 6.98 – 6.92 (m, 1H), 6.86 (dd, J = 7.7, 1.7 Hz, 1H), 6.65 – 6.58 (m, 2H), 2.39 (s, 3H) ppm. ^{13}C{^{1}H} NMR (101 MHz, dimethyl sulfoxide-d_6): δ 191.9, 154.2, 145.7, 137.1, 136.1, 135.8, 133.0, 132.8, 131.5, 131.33, 131.31, 130.7, 128.5, 128.4, 127.2, 127.1, 125.7, 118.5, 115.2, 20.6 ppm. IR (ATR): ν = 3273, 1367 (O-H, phenol), 1672 (C=O, aldehyde) cm^{-1}. HRMS (ESI+) *m*/*z*: calcd. for $C_{20}H_{16}NaO_2$ [M + Na]$^+$: 311.1043, found: 311.1041.

2''-Hydroxy-4'-methyl-[1,1':2',1''-terphenyl]-2-carbaldehyde (1u)

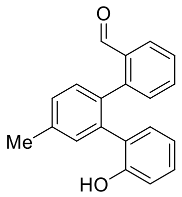

The title compound was synthesized according to the general procedure with 9-methyl-6*H*-dibenzo[*c*,*e*][1,2]oxaborinin-6-ol[1] (300 mg, 1.43 mmol) and 2-bromobenzaldehyde (0.25 ml, 2.14 mmol). The crude product was purified by column chromatography (hexane/EtOAc, 7:1-6:1), affording **1u** (324 mg, 79%) as a white amorphous solid.

^{1}H NMR (400 MHz, dimethyl sulfoxide-d_6): δ 9.78 (s, 1H), 9.18 (s, 1H), 7.70 (d, J = 7.7 Hz, 1H), 7.46 (t, J = 7.4 Hz, 1H), 7.35 (t, J = 7.5 Hz, 1H), 7.29 – 7.17 (m, 3H), 7.15 (d, J = 7.6 Hz, 1H), 6.96 (td, J = 7.7, 1.7 Hz, 1H), 6.93 – 6.86 (m, 1H), 6.63 (t, J = 7.4 Hz, 2H), 2.40 (s, 3H) ppm. ^{13}C{^{1}H} NMR (101 MHz, dimethyl sulfoxide-d_6): δ 192.0, 154.1, 145.7, 138.6, 137.1, 134.4, 133.1, 132.8, 131.6, 131.4, 131.2, 130.8, 128.5, 127.5, 127.3, 127.1, 125.8, 118.5, 115.2, 20.7 ppm. IR (ATR): ν = 3278, 1363 (O-H, phenol), 1676 (C=O, aldehyde) cm^{-1}. HRMS (ESI+) *m*/*z*: calcd. for $C_{20}H_{16}NaO_2$ [M + Na]$^+$: 311.1043, found: 311.1039.

2''-Hydroxy-5,5''-dimethyl-[1,1':2',1''-terphenyl]-2-carbaldehyde (1v)

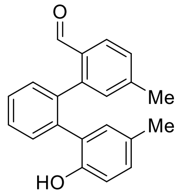

The title compound was synthesized according to the general procedure with 2-methyl-6*H*-dibenzo[*c*,*e*][1,2]oxaborinin-6-ol[1] (300 mg, 1.43 mmol) and 2-bromo-4-methylbenzaldehyde (426 mg, 2.14 mmol). The crude product was purified by column chromatography (hexane/EtOAc, 8:1), affording **1v** (343 mg, 79%) as a white amorphous solid.

^{1}H NMR (400 MHz, dimethyl sulfoxide-*d*$_6$): δ 9.67 (d, *J* = 0.8 Hz, 1H), 8.95 (s, 1H), 7.59 (d, *J* = 8.0 Hz, 1H), 7.44 (dtd, *J* = 17.8, 7.4, 1.6 Hz, 2H), 7.33 (ddd, *J* = 15.1, 7.4, 1.7 Hz, 2H), 7.26 – 7.15 (m, 1H), 7.05 (d, *J* = 1.7 Hz, 1H), 6.77 (dd, *J* = 8.3, 2.3 Hz, 1H), 6.69 (d, *J* = 2.2 Hz, 1H), 6.52 (d, *J* = 8.2 Hz, 1H), 2.27 (s, 3H), 2.05 (s, 3H) ppm. ^{13}C{^{1}H} NMR (101 MHz, dimethyl sulfoxide-*d*$_6$): δ 191.3, 151.9, 145.7, 143.1, 138.8, 137.3, 132.0, 131.6, 130.79, 130.75, 130.6, 128.9, 128.0, 127.7, 126.9, 126.8, 126.7, 125.8, 115.0, 21.2, 20.0 ppm. IR (ATR): ν = 3313, 1306 (O-H, phenol), 1662 (C=O, aldehyde) cm^{-1}. HRMS (ESI+) *m*/*z*: calcd. for $C_{21}H_{18}NaO_2$ [M + Na]$^+$: 325.1199, found: 325.1197.

5,5''-Difluoro-2''-hydroxy-[1,1':2',1''-terphenyl]-2-carbaldehyde (1w)

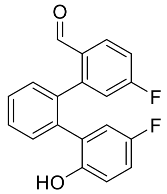

The title compound was synthesized according to the general procedure with 2-fluoro-6*H*-dibenzo[*c*,*e*][1,2]oxaborinin-6-ol[1] (300 mg, 1.40 mmol) and 2-bromo-4-fluorobenzaldehyde (426 mg, 2.10 mmol). The crude product was purified by column chromatography (hexane/EtOAc, 8:1), affording **1w** (343 mg, 79%) as a white amorphous solid.

^{1}H NMR (400 MHz, dichloromethane-*d*$_2$): δ 9.72 (s, 1H), 7.83 (dd, *J* = 8.7, 5.9 Hz, 1H), 7.62 – 7.42 (m, 3H), 7.37 (dd, *J* = 7.4, 1.6 Hz, 1H), 7.13 – 7.01 (m, 1H), 6.94 (dd, *J* = 9.3, 2.5 Hz, 1H), 6.80 (ddd, *J* = 8.9, 8.1, 3.1 Hz, 1H), 6.70 (dd, *J* = 8.9, 3.1 Hz, 1H), 6.64 (dd, *J* = 8.9, 4.7 Hz, 1H), 5.70 (s, 1H) ppm. ^{13}C{^{1}H} NMR (101 MHz, dichloromethane-*d*$_2$): δ 191.89, 165.50 (d, *J* = 256.4 Hz), 156.77 (d, *J* = 238.1 Hz), 149.51, 147.60 (d, *J* = 8.2 Hz), 137.48, 136.55, 131.60, 131.25, 131.24, 130.90 (d, *J* = 2.3 Hz), 129.32, 128.66, 128.38 (d, *J* = 7.8 Hz), 118.88 (d, *J* = 22.1 Hz), 117.67 (d, *J* = 23.2 Hz), 116.96 (d, *J* = 8.3 Hz), 115.86 (d, *J* = 23.0 Hz), 115.52 (d, *J* = 22.0 Hz) ppm. ^{19}F NMR (376 MHz, dichloromethane-*d*$_2$): δ -104.51 (s, 1F), -125.16 (s, 1F) ppm. IR (ATR): ν = 3327, 1350 (O-H, phenol), 1670 (C=O, aldehyde) cm^{-1}. HRMS (ESI+) *m*/*z*: calcd. for $C_{19}H_{12}F_2NaO_2$ [M + Na]$^+$: 333.0698, found: 333.0694.

5-Fluoro-2''-hydroxy-5''-methyl-[1,1':2',1''-terphenyl]-2-carbaldehyde (1x)

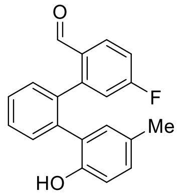

The title compound was synthesized according to the general procedure with 2-methyl-6*H*-dibenzo[*c*,*e*][1,2]oxaborinin-6-ol[1] (300 mg, 1.43 mmol) and 2-bromo-4-fluorobenzaldehyde (435 mg, 2.14 mmol). The crude product was purified by column chromatography (hexane/EtOAc, 8:1-7:1), affording **1x** (338 mg, 77%) as a white amorphous solid.

^{1}H NMR (400 MHz, dichloromethane-*d*$_2$): δ 9.73 (s, 1H), 7.81 (dd, *J* = 8.7, 6.0 Hz, 1H), 7.61 – 7.41 (m, 3H), 7.36 (dd, *J* = 7.5, 1.5 Hz, 1H), 7.06 (td, *J* = 8.4, 2.6 Hz, 1H), 6.78 (d, *J* = 2.2 Hz, 1H), 6.56 (d, *J* = 8.2 Hz, 1H), 5.03 (br s, 1H), 2.15 (s, 3H) ppm. ^{13}C{^{1}H} NMR (101 MHz, dichloromethane-*d*$_2$): δ 191.3, 165.4 (d, *J* = 255.6 Hz), 150.8, 147.9 (d, *J* = 9.6 Hz), 137.63, 137.55, 132.0, 131.4, 131.2, 131.1 (d, *J* = 5.1 Hz) 131.0 (d, *J* = 2.6 Hz), 130.0 (2C), 129.3, 128.2, 127.0, 118.8 (d, *J* = 21.3 Hz), 115.3 (d, *J* = 21.9 Hz), 115.2, 20.4 ppm. ^{19}F NMR (376 MHz, dichloromethane-*d*$_2$): δ -105.34 (d, *J* = 7.8 Hz, 1F) ppm. IR (ATR): ν = 3290, 1354 (O-H, phenol), 1670 (C=O, aldehyde) cm^{-1}. HRMS (ESI+) *m*/*z*: calcd. for $C_{20}H_{15}FNaO_2$ [M + Na]$^+$: 329.0948, found: 329.0947.

Organocatalytic esterification reaction

Complete reaction condition optimization survey

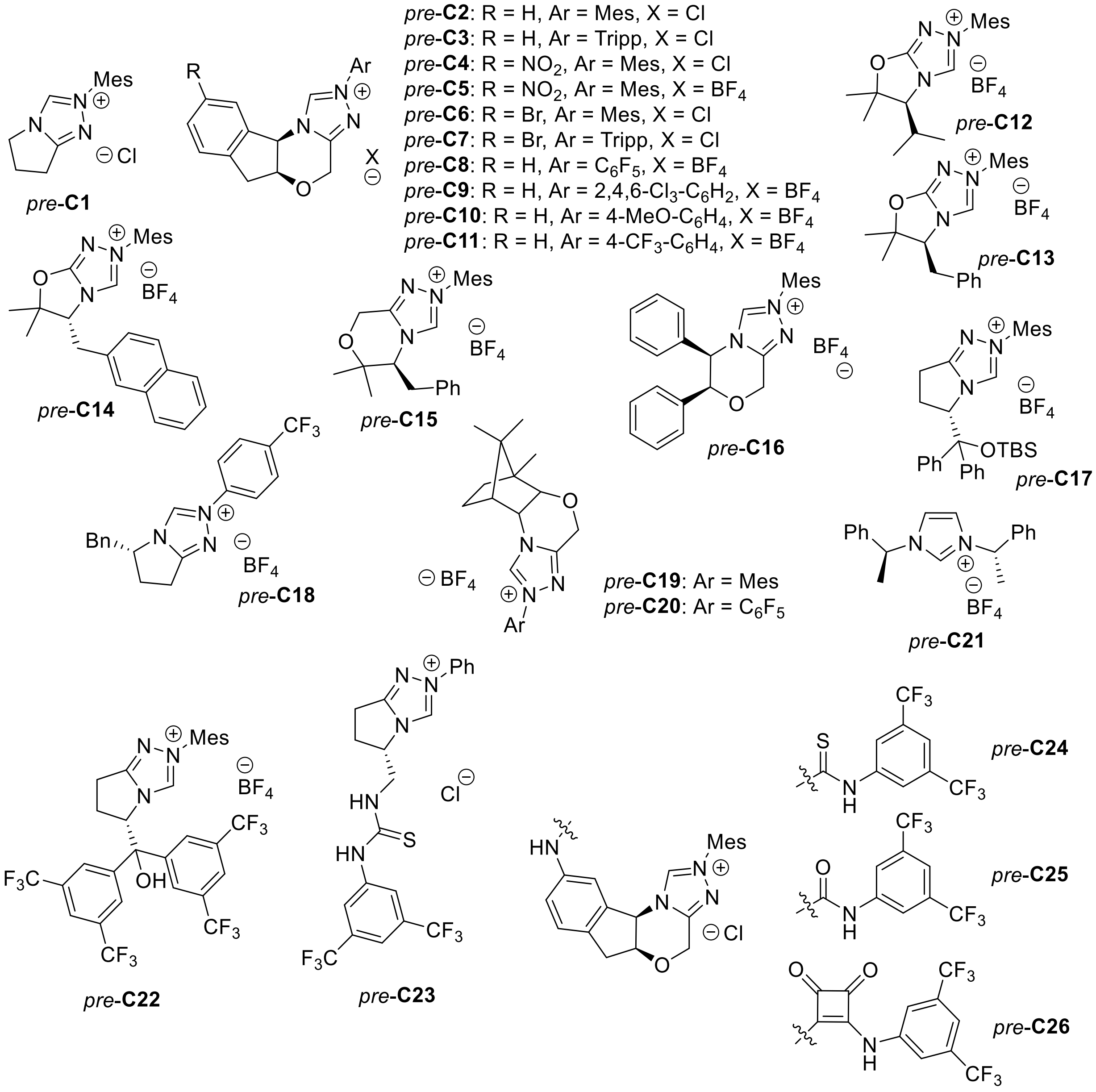

Figure S1. Screened precursors.

Table S1. Precursor screening.

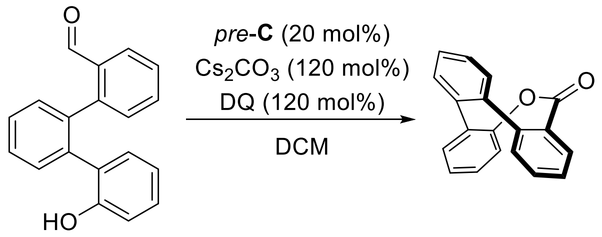

Entry	*pre*-**C**	Time	Conversion	Yield (%)[a]	*er*[b]
1	*pre*-**C1**	20 min	full	73	50:50
2	*pre*-**C2**	7 h	full	94	32:68
3	*pre*-**C3**	4 h	full	63	16:84
4	*pre*-**C4**	1 h	full	70	13:87
5	*pre*-**C5**	4 h	full	74	19:81
6	*pre*-**C6**	3 h	full	69	20:80
7	*pre*-**C7**	3 h	full	30	37:63
8	*pre*-**C8**	2 h	full	82	65:35
9	*pre*-**C9**	6 h	full	95	64:36
10	*pre*-**C10**	1 h	full	61	29:71
11	*pre*-**C11**	2 h	full	60	46:54
12	*pre*-**C12**	7 h	almost full	68	80:20
13	*pre*-**C13**	1 h	full	71	92:8
14	*pre*-**C14**	3 h	full	75	46:54
15	*pre*-**C15**	1 h	full	95	79:21
16	*pre*-**C16**	24 h	full	70	57:43
17	*pre*-**C17**	72 h	not full	4	48:52
18	*pre*-**C18**	10 min	full	51	59:41
19	*pre*-**C19**	72 h	full	85	32:68
20	*pre*-**C20**	24 h	full	24	69:31
21	*pre*-**C21**	72 h	no	-	-
22	*pre*-**C22**	3 h	full	49	73:27
23	*pre*-**C23**	1 h	full	54	62:38
24	*pre*-**C24**	24 h	almost full	53	45:55
25	*pre*-**C25**	24 h	full	31	58:42
26	*pre*-**C26**	72 h	not full	26	42:58

[a] Isolated after column chromatography. [b] Determined by chiral HPLC.

Table S2. Base screening.

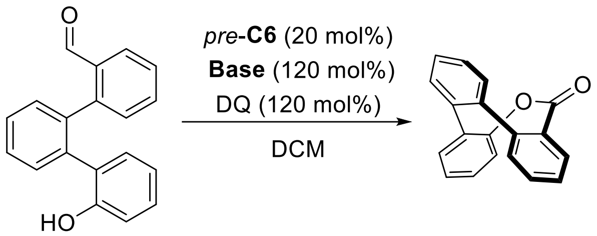

Entry	Base	Time	Conversion	Yield (%)[a]	*er*[b]
1	Cs_2CO_3	1 h	full	71	92:8
2	Rb_2CO_3	2 h	full	98	91:9
3	K_2CO_3	24 h	full	69	89:11
4	Na_2CO_3	8 h	full	95	87:13
5	$NaHCO_3$	72 h	not full	75	80:20
6	KO*t*Bu	15 min	full	65	76:24
7	AcONa	72 h	not full	38	54:46
8	TEA	72 h	almost full	64	80:20
9	DIPEA	24 h	full	75	85:15
10	DABCO	72 h	not full	59	81:19
11	DBU	1 h	full	49	90:10
12	pyridine	72 h	no	-	-
13	2,6-lutidine	72 h	no	-	-

[a] Isolated after column chromatography. [b] Determined by chiral HPLC.

Table S3. Solvent screening.

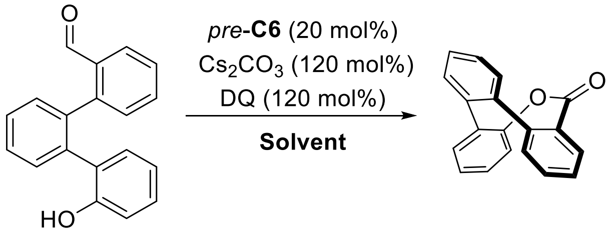

Entry	Base	Time	Conversion	Yield (%)[a]	*er*[b]
1	DCM	1 h	full	71	92:8
2	$CHCl_3$	15 min	full	98	84:16
3	1,2-DCE	30 min	full	98	93:7
4	CCl_4	24 h	full	62	81:19
5	benzene	30 min	full	73	85:15
6	toluene	15 min	full	67	85:15
7	MeCN	18 h	full	47	52:48
9	EtOAc	5 min	full	93	86:14
10	MTBE	24 h	full	75	75:25
11	THF	6 h	full	54	63:37
12	DMSO	72 h	not full	28	49:51
13	*n*-heptane	72 h	not full	63	72:28

[a] Isolated after column chromatography. [b] Determined by chiral HPLC.

Table S4. Oxidant screening.

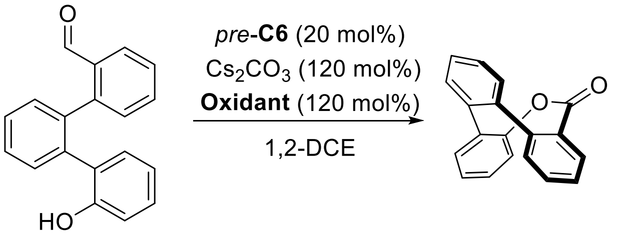

Entry	Oxidant	Time	Conversion	Yield (%)[a]	*er*[b]
1	DQ	30 min	full	98	93:7
2	DDQ	72 h	no	-	-
3	TEMPO	48 h	full	19	62:38
4	acridine	72 h	not full	12	48:52
5	nitrobenzene	72 h	not full	4	49:51
6	MnO_2	72 h	not full	11	49:51
7	CAN	72 h	not full	traces	-

[a] Isolated after column chromatography. [b] Determined by chiral HPLC.

Table S5. Additive screening.

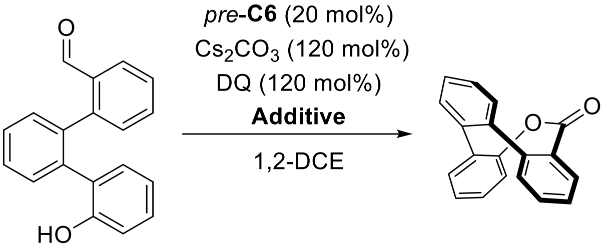

Entry	Additive	Time	Conversion	Yield (%)[a]	*er*[b]
1	none	30 min	full	98	93:7
2	H_2O (100 mol%)	30 min	full	86	92:8
3	MS (50mg, 3A)	45 min	full	97	92:8
4	LiCl (50 mol%)	60 min	full	98	92:8
5	(*R,R*)-TUC (20 mol%)	90 min	full	98	92:8

[a] Isolated after column chromatography. [b] Determined by chiral HPLC. MS = molecular sieves, (*R,R*)-TUC = 1-[3,5-Bis(trifluoromethyl)phenyl]-3-[(1*R*,2*R*)-(−)-2-(dimethylamino)cyclohexyl]thiourea, CAS No.: 620960-26-1

Table S6. Catalyst, base, oxidant loading screening.

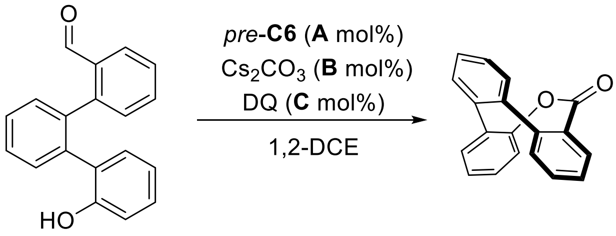

Entry	A	B	C	Time	Conversion	Yield (%)[a]	*er*[b]
1	20	120	120	30 min	full	98	93:7
2	25	120	120	60 min	full	98	92:8
3	10	120	120	45 min	full	97	72:28
4	5	120	120	24 h	full	45	49:51
5	1	120	120	72 h	not full	traces	-
6	20	200	120	30 min	full	81	92:8
7	20	150	120	30 min	full	98	92:8
8	20	100	120	1 h	full	97	92:8
9	20	50	120	6 h	full	98	90:10
10	20	150	150	90 min	full	96	92:8
11[d]	20	120	120	2 h	full	89	91:9
12[d]	20	120	100	2 h	almost full	98	91:9

[a] Isolated after column chromatography. [b] Determined by chiral HPLC. [d] Under inert atmosphere.

Table S7. Concentration and temperature screening.

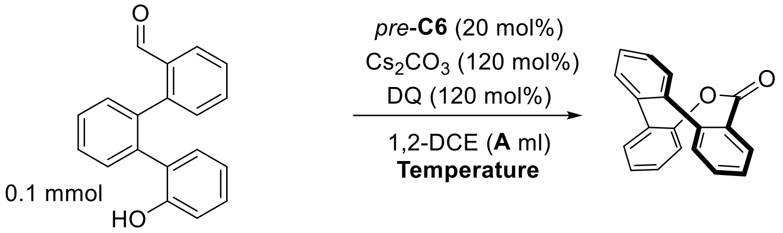

Entry	A	Temperature	Time	Conversion	Yield (%)[a]	*er*[b]
1	2.0	r.t. (~21°C)	30 min	full	98	93:7
2	3.0	r.t (~21°C)	2 h	full	98	92:8
3	1.0	r.t. (~21°C)	2 h	full	98	92:8
4	2.0	40	30 min	full	74	88:12
5	2.0	10	24 h	full	98	97:3
6	2.0	0	48 h	full	89	96:4

[a] Isolated after column chromatography. [b] Determined by chiral HPLC.

General procedure for organocatalytic esterification

The Schlenk tube (10 ml) was charged with aldehyde **1** (0.1 mmol, 1.0 equiv.), *pre*-**C6** (5.3 mg, 0.02 mmol, 0.2 equiv.), DQ (49.0 mg, 0.12 mmol, 1.2 equiv.), Cs_2CO_3 (37.4 mg, 0.12 mmol, 2.0 equiv.), and dissolved in precooled (to 10 °C) 1,2-DCE (2.0 ml). At 10 °C (cryocooler), the reaction mixture was stirred for the indicated time. Once the aldehyde **1** was no longer detected by TLC, the reaction mixture was directly loaded to the silica gel column chromatography, and the product was eluted by hexane/EtOAc mixtures.

*Note: Racemic samples were prepared in reactions with pre-**C1** at room temperature.*

Characterization data of products

10*H*-Tribenzo[*b*,*d*,*f*]oxocin-10-one (2a)

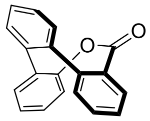

The title compound was synthesized according to the general procedure for organocatalytic esterification (reaction time: 30 h), using aldehyde **1a** (27.4 mg, 0.1 mmol). The crude product was purified by column chromatography (hexane/EtOAc – 9:1), affording **2a** (26.7 mg, 98%) as a white amorphous solid.

Notes: We recommend loading the crude reaction mixture directly onto a silica gel column. Dry loading may cause inconsistent results due to crystallization of the product on the column. For the same reason, we recommend using the smallest column possible and avoiding prolonged standing of the sample on the column.

In the large-scale reaction, solids were filtered before partial evaporation of the solvent, resulting in a nearly saturated solution, which was directly loaded onto a short pad of silica and eluted.

Crystals suitable for X-ray analysis were obtained by diffusion of methanol to chloroform at room temperature. M.p. = 263.0-263.5 °C ($CHCl_3$/MeOH).

Er = 97:3 (*ee* = 94%), the enantiomeric excess of product **2a** was determined by high-performance liquid chromatography (HPLC) on a Chiralpak® IG column (*n*-heptane/*i*-PrOH - 90:10, flow rate = 1.0 ml/min, λ = 190 nm, t = 25 °C): t_R = 11.3 min (minor), t_R = 12.8 min (major). $[\alpha]_D^{20}$ = -62.1 (c = 0.8, $CHCl_3$). ^{1}H NMR (400 MHz, dichloromethane-d_2): δ 7.60 – 7.50 (m, 2H), 7.49 – 7.34 (m, 5H), 7.33 – 7.27 (m, 1H), 7.25 – 7.12 (m, 4H) ppm. $^{13}C\{^1H\}$ NMR (101 MHz, dichloromethane-d_2): δ 169.6, 150.9, 139.7, 138.6, 136.2, 134.9, 132.2, 131.4,

131.0, 130.1, 130.0, 129.7, 129.4, 129.1, 129.0, 128.4, 127.5, 126.9, 121.5 ppm. IR (ATR): ν = 1724 (C=O, ester) cm^{-1}. HRMS (ESI+) *m*/*z*: calcd. for $C_{19}H_{12}NaO_2$ $[M + Na]^+$: 295.0730, found: 295.0730.

10*H*-Tribenzo[*b*,*d*,*f*]oxocin-10-one (*ent*-2a)

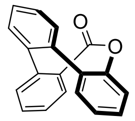

The title compound was synthesized according to the modified general procedure for organocatalytic esterification (reaction time: 28 h), using aldehyde **1a** (27.4 mg, 0.1 mmol) and *ent*-*pre*-**C6** instead of *pre*-**C6**. The crude product was purified by column chromatography (hexane/EtOAc – 9:1), affording *ent*-**2a** (26.8 mg, 98%) as a white amorphous solid.

Er = 96:4 (*ee* = 91%), the enantiomeric excess of product *ent*-**2a** was determined by HPLC on a Chiralpak® IG column (*n*-heptane/*i*-PrOH - 90:10, flow rate = 1.0 ml/min, λ = 190 nm, *t* = 25 °C): t_R = 11.3 min (major), t_R = 12.9 min (minor). $[\alpha]_D^{20}$ = +65.9 (*c* = 1.1, $CHCl_3$). Other analytical data agree with the data on the opposite enantiomer (**2a**).

13-Methyl-10*H*-tribenzo[*b*,*d*,*f*]oxocin-10-one (2b)

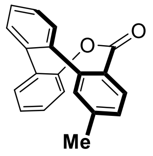

The title compound was synthesized according to the general procedure for organocatalytic esterification (reaction time: 18 h), using aldehyde **1b** (28.8 mg, 0.1 mmol). The crude product was purified by column chromatography (hexane/EtOAc – 9:1), affording **2b** (28.4 mg, 99%) as a white amorphous solid. *Er* = 97:3 (*ee* = 95%), the enantiomeric excess of product **2b** was determined by HPLC on a Chiralpak® IB column (*n*-heptane/*i*-PrOH - 80:20, flow rate = 1.0 ml/min, λ = 190 nm, *t* = 25 °C): t_R = 5.9 min (major), t_R = 6.9 min (minor). $[\alpha]_D^{20}$ = -57.3 (*c* = 1.1, $CHCl_3$). ^1H NMR (400 MHz, dichloromethane-d_2): δ 7.59 – 7.50 (m, 2H), 7.45 – 7.40 (m, 1H), 7.40 – 7.35 (m, 2H), 7.30 (ddd, *J* = 7.9, 5.6, 3.6 Hz, 1H), 7.22 (dd, *J* = 4.2, 1.4 Hz, 2H), 7.18 (dt, *J* = 7.8, 0.9 Hz, 2H), 6.98 – 6.95 (m, 1H), 2.32 (s, 3H) ppm. $^{13}C\{^1H\}$ NMR (101 MHz, dichloromethane-d_2): δ 169.9, 151.0, 142.1, 139.8, 138.7, 136.2, 135.0, 130.94, 130.91, 130.1, 129.7, 129.3, 129.2, 129.1, 129.0, 128.9, 127.8, 126.8, 121.5, 21.5 ppm. IR (ATR): ν = 1732 (C=O, ester) cm^{-1}. HRMS (ESI+) *m*/*z*: calcd. for $C_{20}H_{14}NaO_2$ $[M + Na]^+$: 309.0886, found: 309.0883.

11-Methyl-10*H*-tribenzo[*b*,*d*,*f*]oxocin-10-one (2c)

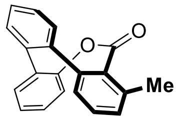

The title compound was synthesized according to the general procedure for organocatalytic esterification (reaction time: 48 h), using aldehyde **1c** (28.8 mg, 0.1 mmol). The crude product was purified by column chromatography (hexane/EtOAc – 9:1), affording **2c** (25.2 mg, 88%) as a white amorphous solid.

Er = 59:41 (*ee* = 18%), the enantiomeric excess of product **2c** was determined by HPLC on a Chiralpak® IG column (*n*-heptane/*i*-PrOH - 80:20, flow rate = 1.0 ml/min, λ = 190 nm, *t* = 25 °C): t_R = 9.0 min (minor), t_R = 9.4 min (major). $[\alpha]_D^{20}$ = -11.5 (*c* = 1.0, $CHCl_3$). ^1H NMR (400 MHz, chloroform-*d*): δ 7.50 (ddd, *J* = 7.4, 4.7, 3.6 Hz, 2H), 7.46 – 7.41 (m, 1H), 7.41 – 7.35 (m, 1H), 7.31 – 7.20 (m, 2H), 7.20 – 7.07 (m, 4H), 6.99 – 6.92 (m, 1H), 2.42 (s, 3H) ppm. $^{13}C\{^1H\}$ NMR (101 MHz, chloroform-*d*): δ 168.95, 150.58, 139.76, 138.19, 136.10, 134.52, 134.21, 132.34, 130.92, 129.97, 129.47, 129.36, 129.20, 129.11, 128.92, 128.87, 126.59, 126.45, 120.08, 19.72 ppm. IR (ATR): ν = 1738 (C=O, ester) cm^{-1}. HRMS (ESI+) *m*/*z*: calcd. for $C_{20}H_{15}O_2$ $[M + H]^+$: 287.1067, found: 287.1077.

12-Methyl-10*H*-tribenzo[*b*,*d*,*f*]oxocin-10-one (2d)

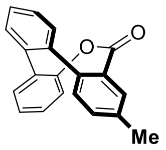

The title compound was synthesized according to the general procedure for organocatalytic esterification (reaction time: 20 h), using aldehyde **1d** (28.8 mg, 0.1 mmol). The crude product was purified by column chromatography (hexane/EtOAc – 9:1), affording **2d** (26.0 mg, 91%) as a white amorphous solid. *Er* = 95:5 (*ee* = 90%), the enantiomeric excess of product **2d** was determined by HPLC on a Chiralpak® IB column (*n*-heptane/*i*-PrOH - 80:20, flow rate = 1.0 ml/min, λ = 215 nm, t = 25 °C): t_R = 5.7 min (major), t_R = 7.0 min (minor). $[\alpha]_D^{20}$ = -40.5 (c = 0.7, $CHCl_3$). ^1H NMR (400 MHz, chloroform-*d*): δ 7.54 – 7.45 (m, 2H), 7.39 (dd, J = 6.9, 2.1 Hz, 1H), 7.37 – 7.32 (m, 1H), 7.31 – 7.24 (m, 2H), 7.23 – 7.13 (m, 4H), 7.01 (d, J = 7.9 Hz, 1H), 2.33 (s, 3H) ppm. $^{13}C\{^1H\}$ NMR (101 MHz, chloroform-*d*): δ 170.1, 150.7, 139.4, 138.1, 136.0, 135.7, 134.7, 132.0, 131.6, 130.6, 129.9, 129.8, 129.3, 128.8, 128.73, 128.71, 127.9, 126.6, 121.3, 21.0 ppm. IR (ATR): ν = 1732 (C=O, ester) cm^{-1}. HRMS (ESI+) *m*/*z*: calcd. for $C_{20}H_{15}O_2$ $[M + H]^+$: 287.1067, found: 287.1078.

11-Methyl-10*H*-tribenzo[*b*,*d*,*f*]oxocin-10-one (2e)

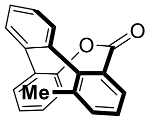

The title compound was synthesized according to the general procedure for organocatalytic esterification (reaction time: 48 h, without full conversion of starting material), using aldehyde **1e** (28.8 mg, 0.1 mmol). The crude product was purified by column chromatography (hexane/EtOAc – 9:1), affording **2e** (12.3 mg, 43%) as a white amorphous solid and recovered enantioenriched staring material **(-)-1e** (13.2 mg, 47%) as a white amorphous solid.

Er = 96:4 (*ee* = 91%), the enantiomeric excess of product **2e** was determined by HPLC on a Chiralpak® IB column (*n*-heptane/*i*-PrOH - 80:20, flow rate = 1.0 ml/min, λ = 190 nm, t = 25 °C): t_R = 6.2 min (major), t_R = 7.2 min (minor). $[\alpha]_D^{20}$ = -73.7 (c = 0.5, $CHCl_3$). ^1H NMR (400 MHz, dichloromethane-d_2): δ 7.53 (pd, J = 7.5, 1.7 Hz, 2H), 7.45 (dd, J = 7.2, 1.8 Hz, 1H), 7.36 – 7.31 (m, 1H), 7.29 – 7.22 (m, 5H), 7.21 – 7.15 (m, 1H), 7.13 – 7.08 (m, 1H), 2.00 (s, 3H) ppm. $^{13}C\{^1H\}$ NMR (101 MHz, dichloromethane-d_2): δ 169.9, 151.0, 142.1, 139.8, 138.7, 136.2, 135.0, 130.94, 130.91, 130.1, 129.7, 129.3, 129.2, 129.1, 129.0, 128.9, 127.8, 126.8, 121.5, 21.5 ppm. IR (ATR): ν = 1728 (C=O, ester) cm^{-1}. HRMS (ESI+) *m*/*z*: calcd. for $C_{20}H_{15}O_2$ $[M + H]^+$: 287.1067, found: 287.1075.

2''-Hydroxy-6-methyl-[1,1':2',1''-terphenyl]-2-carbaldehyde **[(-)-1e]**

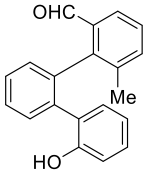

Er = 98:2 (*ee* = 96%), the enantiomeric excess of product **(-)-1e** was determined by HPLC on a Chiralpak® IB column (*n*-heptane/*i*-PrOH - 80:20, flow rate = 1.0 ml/min, λ = 190 nm, t = 25 °C): t_R = 4.6 min (minor), t_R = 6.7 min (major). $[\alpha]_D^{20}$ = -32.3 (c = 1.1, $CHCl_3$). Other analytical data agree with the data for racemic compound (**1e**).

13-Methoxy-10*H*-tribenzo[*b*,*d*,*f*]oxocin-10-one (2f)

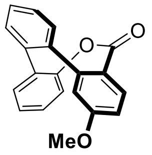

The title compound was synthesized according to the general procedure for organocatalytic esterification (reaction time: 18 h), using aldehyde **1f** (30.4 mg, 0.1 mmol). The crude product was purified by column chromatography (hexane/EtOAc – 5:1), affording **2f** (30.0 mg, 99%) as a white amorphous solid. *Er* = 96:4 (*ee* = 93%), the enantiomeric excess of product **2f** was determined by HPLC on a Chiralpak® IB column (*n*-heptane/*i*-PrOH - 80:20, flow rate = 1.0 ml/min, λ = 190 nm, t = 25 °C): t_R = 7.3 min (major), t_R = 9.1 min (minor). $[\alpha]_D^{20}$ = -30.2 (c = 1.3, $CHCl_3$). ^1H NMR (400 MHz, dichloromethane-d_2): δ 7.54 (pd, J = 7.4, 1.7 Hz, 2H), 7.43 (td, J = 5.2, 2.9 Hz, 2H), 7.39 (dd, J = 7.3, 1.8 Hz, 1H), 7.34 – 7.27 (m, 1H), 7.23 (qd, J = 7.5, 1.9 Hz, 2H), 7.18 (d, J = 7.7 Hz, 1H), 6.88 (dd, J = 8.6, 2.6 Hz, 1H), 6.64 (d, J = 2.5 Hz, 1H), 3.77 (s, 3H) ppm. $^{13}C\{^1H\}$ NMR (101 MHz, dichloromethane-d_2): δ 169.7, 161.9, 151.1, 140.9, 139.7,

136.1, 134.9, 130.8, 130.2, 130.1, 129.7, 129.3, 129.0, 128.9, 126.8, 124.5, 121.6, 115.6, 113.9, 55.8 ppm. IR (ATR): ν = 1739 (C=O, ester) cm^{-1}. HRMS (ESI+) *m*/*z*: calcd. for $C_{20}H_{14}NaO_3$ $[M + Na]^+$: 325.0835, found: 325.0834.

13-Fluoro-10*H*-tribenzo[*b*,*d*,*f*]oxocin-10-one (2g)

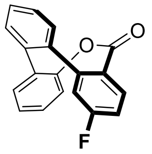

The title compound was synthesized according to the general procedure for organocatalytic esterification (reaction time: 18 h), using aldehyde **1g** (29.2 mg, 0.1 mmol). The crude product was purified by column chromatography (hexane/EtOAc – 10:1), affording **2g** (28.5 mg, 98%) as a white amorphous solid. *Er* = 96:4 (*ee* = 91%), the enantiomeric excess of product **2g** was determined by HPLC on a Chiralpak® IB column (*n*-heptane/*i*-PrOH - 80:20, flow rate = 1.0 ml/min, λ = 190 nm, *t* = 25 °C): t_R = 5.9 min (major), t_R = 7.1 min (minor). $[\alpha]_D^{20}$ = -59.8 (*c* = 1.2, $CHCl_3$). ^1H NMR (400 MHz, dichloromethane-*d*$_2$): δ 7.62 – 7.51 (m, 2H), 7.49 (dd, *J* = 8.6, 5.6 Hz, 1H), 7.44 (dd, *J* = 7.0, 2.0 Hz, 1H), 7.40 – 7.35 (m, 1H), 7.32 (dt, *J* = 8.0, 4.6 Hz, 1H), 7.25 – 7.21 (m, 2H), 7.19 (dt, *J* = 7.9, 0.9 Hz, 1H), 7.06 (td, *J* = 8.4, 2.6 Hz, 1H), 6.87 (dd, *J* = 9.3, 2.5 Hz, 1H) ppm. $^{13}C\{^1H\}$ NMR (101 MHz, dichloromethane-*d*$_2$): δ 168.5, 163.6 (d, *J* = 251.9 Hz), 150.4, 141.1 (d, *J* = 8.6 Hz), 138.1 (d, *J* = 1.6 Hz), 135.7, 134.1, 130.6, 129.9 (d, *J* = 9.2 Hz), 129.49, 129.46, 129.4, 128.8, 128.7, 128.2 (d, *J* = 3.4 Hz), 126.6, 121.1, 116.8 (d, *J* = 22.5 Hz), 115.2 (d, *J* = 22.1 Hz) ppm. ^{19}F NMR (376 MHz, dichloromethane-*d*$_2$): δ -108.82 (td, *J* = 8.8, 5.6 Hz, 1F) ppm. IR (ATR): ν = 1722 (C=O, ester) cm^{-1}. HRMS (ESI+) *m*/*z*: calcd. for $C_{19}H_{11}FNaO_2$ $[M + Na]^+$: 313.0635, found: 313.0635.

13-Chloro-10*H*-tribenzo[*b*,*d*,*f*]oxocin-10-one (2h)

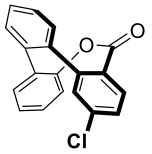

The title compound was synthesized according to the general procedure for organocatalytic esterification (reaction time: 6 h), using aldehyde **1h** (30.9 mg, 0.1 mmol). The crude product was purified by column chromatography (hexane/EtOAc – 9:1), affording **2h** (29.5 mg, 96%) as a colorless oil. *Er* = 97:3 (*ee* = 95%), the enantiomeric excess of product **2h** was determined by HPLC on a Chiralpak® IB column (*n*-heptane/*i*-PrOH - 80:20, flow rate = 1.0 ml/min, λ = 190 nm, *t* = 25 °C): t_R = 5.7 min (major), t_R = 7.1 min (minor). $[\alpha]_D^{20}$ = -87.4 (*c* = 0.7, $CHCl_3$). ^1H NMR (400 MHz, dichloromethane-*d*$_2$): δ 9.57 (s, 1H), 7.93 – 7.79 (m, 3H), 7.73 – 7.65 (m, 3H), 7.54 – 7.44 (m, 2H), 7.35 (dd, *J* = 8.8, 2.3 Hz, 1H), 7.05 – 6.96 (m, 2H), 6.80 (dq, *J* = 8.0, 2.0 Hz, 2H), 6.76 – 6.68 (m, 1H), 6.49 – 6.34 (m, 3H), 4.50 (td, *J* = 19.5, 19.0, 13.7 Hz, 4H), 4.11 – 3.71 (m, 8H), 3.40 – 3.14 (m, 4H), 2.01 – 1.83 (m, 8H), 1.05 (td, *J* = 7.5, 6.1 Hz, 6H), 0.99 (q, *J* = 7.6 Hz, 6H) ppm. $^{13}C\{^1H\}$ NMR (101 MHz, dichloromethane-*d*$_2$): δ 168.8, 150.7, 140.5, 138.3, 137.2, 136.2, 134.5, 131.1, 130.8, 130.2, 129.89, 129.87, 129.8, 129.23, 129.17 (2C), 128.6, 127.1, 121.5 ppm. IR (ATR): ν = 1732 (C=O, ester) cm^{-1}. HRMS (ESI+) *m*/*z*: calcd. for $C_{19}H_{11}ClNaO_2$ $[M + Na]^+$: 329.0340, found: 329.0338.

13-Bromo-10*H*-tribenzo[*b*,*d*,*f*]oxocin-10-one (2i)

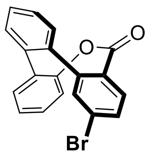

The title compound was synthesized according to the general procedure for organocatalytic esterification (reaction time: 40 h), using aldehyde **1i** (35.3 mg, 0.1 mmol). The crude product was purified by column chromatography (hexane/EtOAc – 10:1), affording **2i** (30.4 mg, 87%) as a white amorphous solid. *Er* = 94:6 (*ee* = 87%), the enantiomeric excess of product **2i** was determined by HPLC on a Chiralpak® IB column (*n*-heptane/*i*-PrOH - 80:20, flow rate = 1.0 ml/min, λ = 190 nm, *t* = 25 °C): t_R = 5.9 min (minor), t_R = 7.4 min (major). $[\alpha]_D^{20}$ = -79.8 (*c* = 1.3, $CHCl_3$). ^1H NMR (400 MHz, dichloromethane-*d*$_2$): δ 7.61 – 7.52 (m, 2H), 7.50 (dt, *J* = 8.2, 1.9 Hz, 1H), 7.46 – 7.42 (m, 1H), 7.40 – 7.36 (m, 1H), 7.36 – 7.28 (m, 3H), 7.27 – 7.21 (m, 2H), 7.20 – 7.16 (m, 1H) ppm. $^{13}C\{^1H\}$ NMR (101 MHz, dichloromethane-*d*$_2$): δ 168.9, 150.7, 140.5, 138.2,

136.2, 134.5, 133.1, 131.6, 131.2, 131.1, 129.91, 129.88, 129.85, 129.23, 129.20, 129.17, 127.1, 125.5, 121.5 ppm. IR (ATR): ν = 1734 (C=O, ester) cm^{-1}. HRMS (ESI+) *m*/*z*: calcd. for $C_{19}H_{11}BrNaO_2$ $[M + Na]^+$: 372.9835, found: 372.9833.

10-oxo-10*H*-tribenzo[*b*,*d*,*f*]oxocine-13-carbonitrile (2j)

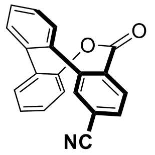

The title compound was synthesized according to the general procedure for organocatalytic esterification (reaction time: 24 h), using aldehyde **1j** (29.9 mg, 0.1 mmol). The crude product was purified by column chromatography (hexane/EtOAc – 5:1), affording **2j** (28.8 mg, 97%) as a white amorphous solid. *Er* = 83:17 (*ee* = 67%), the enantiomeric excess of product **2j** was determined by HPLC on a Chiralpak® IB column (*n*-heptane/*i*-PrOH - 80:20, flow rate = 1.0 ml/min, λ = 190 nm, t = 25 °C): t_R = 10.2 min (minor), t_R = 13.1 min (major). $[\alpha]_D^{20}$ = -84.1 (c = 1.3, $CHCl_3$). ^1H NMR (400 MHz, dichloromethane-d_2): δ 7.69 – 7.54 (m, 4H), 7.50 – 7.44 (m, 2H), 7.43 – 7.38 (m, 1H), 7.33 (ddd, J = 7.9, 6.8, 2.4 Hz, 1H), 7.27 – 7.17 (m, 3H) ppm. $^{13}C\{^1H\}$ NMR (101 MHz, dichloromethane-d_2): δ 167.9, 150.4, 139.7, 137.5, 136.3, 136.1, 134.1, 133.6, 131.9, 131.3, 130.3, 130.1, 129.7, 129.5, 129.4, 128.2, 127.4, 121.4, 117.9, 115.2 ppm. IR (ATR): ν = 1749 (C=O, ester) 2229 (CN, nitrile) cm^{-1}. HRMS (ESI+) *m*/*z*: calcd. for $C_{20}H_{11}NNaO_2$ $[M + Na]^+$: 320.0682, found: 320.0684.

13-(Trifluoromethyl)-10*H*-tribenzo[*b*,*d*,*f*]oxocin-10-one (2k)

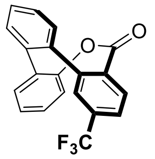

The title compound was synthesized according to the general procedure for organocatalytic esterification (reaction time: 18 h), using aldehyde **1k** (34.2 mg, 0.1 mmol). The crude product was purified by column chromatography (hexane/EtOAc – 10:1), affording **2k** (26.4 mg, 78%) as a white amorphous solid. *Er* = 78:22 (*ee* = 57%), the enantiomeric excess of product **2k** was determined by HPLC on a Chiralpak® IB column (*n*-heptane/*i*-PrOH - 80:20, flow rate = 1.0 ml/min, λ = 190 nm, t = 25 °C): t_R = 5.2 min (major), t_R = 6.9 min (minor). $[\alpha]_D^{20}$ = -35.5 (c = 1.1, $CHCl_3$). ^1H NMR (400 MHz, dichloromethane-d_2): δ 7.65 – 7.53 (m, 4H), 7.49 – 7.40 (m, 3H), 7.32 (ddd, J = 7.9, 6.2, 3.0 Hz, 1H), 7.27 – 7.18 (m, 3H) ppm. $^{13}C\{^1H\}$ NMR (101 MHz, dichloromethane-d_2): δ 168.4, 150.6, 139.5, 138.2, 136.2, 135.7, 134.3, 132.9 (q, J = 32.8 Hz), 131.3, 130.04, 130.01, 129.9, 129.40, 129.38, 128.2, 127.3, 127.0 (q, J = 3.8 Hz), 125.3 (q, J = 3.7 Hz), 123.9 (q, J = 272.9 Hz), 121.4 ppm. ^{19}F NMR (376 MHz, dichloromethane-d_2): δ -61.44 (s, 3F) ppm. IR (ATR): ν = 1755 (C=O, ester) cm^{-1}. HRMS (ESI+) *m*/*z*: calcd. for $C_{20}H_{11}F_3NaO_2$ $[M + Na]^+$: 363.0603, found: 363.0605.

13-Nitro-10*H*-tribenzo[*b*,*d*,*f*]oxocin-10-one (2l)

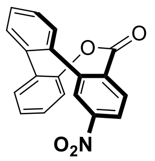

The title compound was synthesized according to the general procedure for organocatalytic esterification (reaction time: 6 h), using aldehyde **1l** (31.9 mg, 0.1 mmol). The crude product was purified by column chromatography (hexane/EtOAc – 5:1), affording **2l** (29.8 mg, 94%) as an yellow amorphous solid. *Er* = 77:23 (*ee* = 53%), the enantiomeric excess of product **2l** was determined by HPLC on a Chiralpak® IB column (*n*-heptane/*i*-PrOH - 80:20, flow rate = 1.0 ml/min, λ = 190 nm, t = 25 °C): t_R = 10.1 min (major), t_R = 12.4 min (minor). $[\alpha]_D^{20}$ = -57.1 (c = 1.3, $CHCl_3$). ^1H NMR (400 MHz, dichloromethane-d_2): δ 8.18 (dd, J = 8.5, 2.2 Hz, 1H), 8.03 (d, J = 2.2 Hz, 1H), 7.68 – 7.56 (m, 3H), 7.47 (ddd, J = 7.5, 5.8, 1.8 Hz, 2H), 7.33 (ddd, J = 7.7, 6.1, 3.1 Hz, 1H), 7.25 – 7.18 (m, 3H) ppm. $^{13}C\{^1H\}$ NMR (101 MHz, dichloromethane-d_2): δ 167.8, 150.3, 149.3, 140.3, 138.0, 137.5, 136.1, 134.1, 131.4, 130.4, 130.2, 129.8, 129.54, 129.47, 128.7, 127.4, 125.0, 123.3, 121.4 ppm. IR (ATR): ν = 1746 (C=O, ester), 1522 (N-O, nitro) cm^{-1}. HRMS (ESI+) *m*/*z*: calcd. for $C_{19}H_{11}NNaO_4$ $[M + Na]^+$: 340,0580, found: 340.0578.

6-Methyl-10*H*-tribenzo[*b*,*d*,*f*]oxocin-10-one (2m)

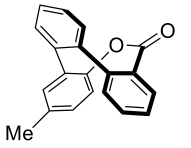

The title compound was synthesized according to the general procedure for organocatalytic esterification (reaction time: 18 h), using aldehyde **1m** (28.8 mg, 0.1 mmol). The crude product was purified by column chromatography (hexane/EtOAc – 9:1), affording **2m** (24.8 mg, 87%) as a white amorphous solid.

Er = 93:7 (*ee* = 87%), the enantiomeric excess of product **2m** was determined by HPLC on a Chiralpak® IB column (*n*-heptane/*i*-PrOH - 80:20, flow rate = 1.0 ml/min, λ = 190 nm, t = 25 °C): t_R = 6.1 min (major), t_R = 6.7 min (minor). $[\alpha]_D^{20}$ = -31.6 (c = 1.2, $CHCl_3$). ^{1}H NMR (400 MHz, dichloromethane-d_2): δ 7.61 – 7.49 (m, 2H), 7.49 – 7.33 (m, 5H), 7.16 (dd, J = 7.6, 1.3 Hz, 1H), 7.13 – 7.04 (m, 2H), 7.01 (d, J = 2.0 Hz, 1H), 2.28 (s, 3H) ppm. ^{13}C{^{1}H} NMR (101 MHz, dichloromethane-d_2): δ 169.9, 148.8, 139.6, 138.7, 136.9, 136.4, 134.4, 132.4, 131.5, 131.3, 130.20, 130.16, 130.0, 129.3, 129.0 (2C), 128.3, 127.5, 121.1, 20.9 ppm. IR (ATR): ν = 1730 (C=O, ester) cm^{-1}. HRMS (ESI+) m/z: calcd. for $C_{20}H_{14}NaO_2$ $[M + Na]^+$: 309.0886, found: 309.0884.

5-Methyl-10*H*-tribenzo[*b*,*d*,*f*]oxocin-10-one (2n)

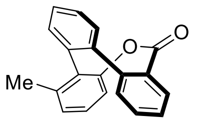

The title compound was synthesized according to the general procedure for organocatalytic esterification (reaction time: 20 h), using aldehyde **1n** (28.8 mg, 0.1 mmol). The crude product was purified by column chromatography (hexane/EtOAc – 9:1), affording **2n** (23.2 mg, 81%) as a white amorphous solid.

Er = 58:42 (*ee* = 16%), the enantiomeric excess of product **2n** was determined by HPLC on a Chiralpak® IB column (*n*-heptane/*i*-PrOH - 80:20, flow rate = 1.0 ml/min, λ = 190 nm, t = 25 °C): t_R = 6.1 min (major), t_R = 7.1 min (minor). $[\alpha]_D^{20}$ = -3.5 (c = 1.1, $CHCl_3$). ^{1}H NMR (400 MHz, chloroform-d): δ 7.53 – 7.44 (m, 2H), 7.43 – 7.34 (m, 4H), 7.30 (td, J = 7.5, 1.3 Hz, 1H), 7.17 (dd, J = 7.6, 1.4 Hz, 1H), 7.13 (t, J = 7.8 Hz, 1H), 7.06 – 6.99 (m, 2H), 2.04 (s, 3H) ppm. ^{13}C{^{1}H} NMR (101 MHz, chloroform-d): δ 169.9, 151.1, 139.4, 138.6, 137.9, 134.2, 133.4, 132.1, 131.0, 129.9, 129.4, 128.68, 128.66, 128.51, 128.46, 128.3, 128.0, 127.3, 118.5, 20.3 ppm. IR (ATR): ν = 1726 (C=O, ester) cm^{-1}. HRMS (ESI+) m/z: calcd. for $C_{20}H_{15}O_2$ $[M + H]^+$: 287.1067, found: 287.1078.

7-Methyl-10*H*-tribenzo[*b*,*d*,*f*]oxocin-10-one (2o)

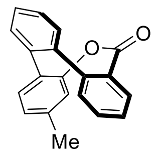

The title compound was synthesized according to the general procedure for organocatalytic esterification (reaction time: 48 h), using aldehyde **1o** (28.8 mg, 0.1 mmol). The crude product was purified by column chromatography (hexane/EtOAc – 9:1), affording **2o** (26.9 mg, 94%) as a white amorphous solid.

Er = 93:7 (*ee* = 87%), the enantiomeric excess of product **2o** was determined by HPLC on a Chiralpak® IB column (*n*-heptane/*i*-PrOH - 80:20, flow rate = 1.0 ml/min, λ = 210 nm, t = 25 °C): t_R = 6.2 min (major), t_R = 7.4 min (minor). $[\alpha]_D^{20}$ = -90.7 (c = 1.1, $CHCl_3$). ^{1}H NMR (400 MHz, chloroform-d): δ 7.55 – 7.45 (m, 3H), 7.43 – 7.30 (m, 4H), 7.13 (dd, J = 7.4, 1.4 Hz, 1H), 7.09 – 7.04 (m, 1H), 6.98 (dd, J = 6.6, 0.9 Hz, 2H), 2.31 (s, 3H) ppm. ^{13}C{^{1}H} NMR (101 MHz, chloroform-d): δ 169.9, 150.4, 139.7, 139.4, 138.6, 135.9, 131.9, 131.5, 131.0, 130.3, 129.8 (2C), 128.94, 128.87, 128.6, 128.0, 127.6, 127.4, 121.6, 21.1 ppm. IR (ATR): ν = 1726 (C=O, ester) cm^{-1}. HRMS (ESI+) m/z: calcd. for $C_{20}H_{15}O_2$ $[M + H]^+$: 287.1067, found: 287.1065.

6-Methyl-10*H*-tribenzo[*b*,*d*,*f*]oxocin-10-one (2p)

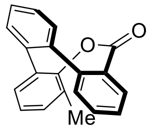

The title compound was synthesized according to the general procedure for organocatalytic esterification (reaction time: 20 h), using aldehyde **1p** (28.8 mg, 0.1 mmol). The crude product was purified by column chromatography (hexane/EtOAc – 9:1), affording **2p** (24.8 mg, 87%) as a white amorphous solid. *Er* = 90:10 (*ee* = 80%), the enantiomeric excess of product **2p** was determined by HPLC on a Chiralpak® IB column (*n*-heptane/*i*-PrOH - 80:20, flow rate = 1.0 ml/min, λ = 190 nm, t = 25 °C): t_R = 5.6 min (major), t_R = 6.0 min (minor). $[\alpha]_D^{20}$ = -93.9 (c = 1.1, $CHCl_3$). ^1H NMR (400 MHz, chloroform-*d*): δ 7.56 – 7.45 (m, 3H), 7.44 – 7.29 (m, 4H), 7.16 – 7.09 (m, 2H), 7.06 (t, J = 7.4 Hz, 1H), 7.01 (dd, J = 7.5, 2.0 Hz, 1H), 2.33 (s, 3H) ppm. $^{13}C\{^1H\}$ NMR (101 MHz, chloroform-*d*): δ 170.1, 149.1, 139.3, 138.7, 136.4, 134.4, 132.1, 131.2, 130.6, 130.3, 129.9, 129.8, 128.9, 128.58, 128.56, 128.11, 128.06, 126.6, 126.2, 16.8 ppm. IR (ATR): ν = 1726 (C=O, ester) cm^{-1}. HRMS (ESI+) *m*/*z*: calcd. for $C_{20}H_{15}O_2$ $[M + H]^+$: 287.1067, found: 287.1067.

6-Fluoro-10*H*-tribenzo[*b*,*d*,*f*]oxocin-10-one (2q)

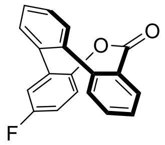

The title compound was synthesized according to the general procedure for organocatalytic esterification (reaction time: 40 h), using aldehyde **1q** (29.2 mg, 0.1 mmol). The crude product was purified by column chromatography (hexane/EtOAc – 9:1), affording **2q** (28.0 mg, 96%) as a white amorphous solid. *Er* = 94:6 (*ee* = 88%), the enantiomeric excess of product **2q** was determined by HPLC on a Chiralpak® IG column (*n*-heptane/*i*-PrOH - 90:10, flow rate = 1.0 ml/min, λ = 190 nm, t = 25 °C): t_R = 9.1 min (minor), t_R = 10.6 min (major). $[\alpha]_D^{20}$ = -26.4 (c = 1.3, $CHCl_3$). ^1H NMR (400 MHz, dichloromethane-d_2): δ 7.60 – 7.52 (m, 2H), 7.50 – 7.33 (m, 5H), 7.20 – 7.13 (m, 2H), 6.98 (ddd, J = 8.8, 7.9, 3.1 Hz, 1H), 6.93 (dd, J = 8.5, 3.1 Hz, 1H) ppm. $^{13}C\{^1H\}$ NMR (101 MHz, dichloromethane-d_2): δ 169.5, 160.5 (d, J = 246.2 Hz), 147.0 (d, J = 2.8 Hz), 139.5, 138.4, 136.6 (d, J = 8.7 Hz), 135.2 (d, J = 1.6 Hz), 131.9, 131.5, 130.3, 130.2, 129.5, 129.4, 128.8, 128.5, 127.6, 123.1 (d, J = 9.1 Hz), 117.4 (d, J = 23.7 Hz), 116.2 (d, J = 23.4 Hz) ppm. ^{19}F NMR (376 MHz, dichloromethane-d_2): δ -116.18 (td, J = 8.2, 4.8 Hz, 1F) ppm. IR (ATR): ν = 1734 (C=O, ester) cm^{-1}. HRMS (ESI+) *m*/*z*: calcd. for $C_{19}H_{11}FNaO_2$ $[M + Na]^+$: 313.0635, found: 313.0633.

6-Chloro-10*H*-tribenzo[*b*,*d*,*f*]oxocin-10-one (2r)

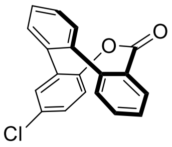

The title compound was synthesized according to the general procedure for organocatalytic esterification (reaction time: 30 h), using aldehyde **1r** (30.8 mg, 0.1 mmol). The crude product was purified by column chromatography (hexane/EtOAc – 9:1), affording **2r** (24.7 mg, 81%) as a white amorphous solid. *Er* = 94:6 (*ee* = 88%), the enantiomeric excess of product **2r** was determined by HPLC on a Chiralpak® IG column (*n*-heptane/*i*-PrOH - 90:10, flow rate = 1.0 ml/min, λ = 190 nm, t = 25 °C): t_R = 8.2 min (minor), t_R = 9.7 min (major). $[\alpha]_D^{20}$ = -10.1 (c = 1.2, $CHCl_3$). ^1H NMR (400 MHz, dichloromethane-d_2): δ 7.60 – 7.52 (m, 2H), 7.50 – 7.34 (m, 5H), 7.26 (dd, J = 8.6, 2.6 Hz, 1H), 7.21 (d, J = 2.5 Hz, 1H), 7.16 (dd, J = 11.3, 7.8 Hz, 2H) ppm. $^{13}C\{^1H\}$ NMR (101 MHz, dichloromethane-d_2): δ 169.2, 149.5, 139.6, 138.3, 136.5, 135.0, 132.0, 131.8, 131.6, 130.7, 130.3, 130.2, 129.6 (2C), 129.5, 128.8, 128.6, 127.6, 123.0 ppm. IR (ATR): ν = 1739 (C=O, ester) cm^{-1}. HRMS (ESI+) *m*/*z*: calcd. for $C_{19}H_{11}ClNaO_2$ $[M + Na]^+$: 329.0340, found: 329.0337.

6-Bromo-10*H*-tribenzo[*b,d,f*]oxocin-10-one (2s)

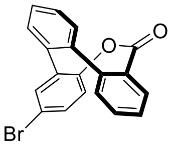

The title compound was synthesized according to the general procedure for organocatalytic esterification (reaction time: 40 h), using aldehyde **1s** (35.3 mg, 0.1 mmol). The crude product was purified by column chromatography (hexane/EtOAc – 9:1), affording **2s** (32.5 mg, 93%) as a white amorphous solid. *Er* = 94:6 (*ee* = 88%), the enantiomeric excess of product **2s** was determined by HPLC on a Chiralpak® IG column (*n*-heptane/*i*-PrOH - 90:10, flow rate = 1.0 ml/min, λ = 190 nm, t = 25 °C): t_R = 8.4 min (minor), t_R = 10.0 min (major). $[\alpha]_D^{20}$ = -25.2 (c = 1.2, $CHCl_3$). ^1H NMR (400 MHz, dichloromethane-d_2): δ 7.60 – 7.52 (m, 2H), 7.47 (ddd, J = 7.9, 3.9, 2.3 Hz, 2H), 7.44 – 7.38 (m, 4H), 7.37 (d, J = 2.4 Hz, 1H), 7.17 (dd, J = 7.9, 1.3 Hz, 1H), 7.08 (d, J = 8.5 Hz, 1H) ppm. $^{13}C\{^1H\}$ NMR (101 MHz, dichloromethane-d_2): δ 169.1, 150.1, 139.6, 138.3, 136.9, 134.9, 133.7, 132.6, 131.8, 131.6, 130.3, 130.2, 129.6, 129.5, 128.9, 128.6, 127.6, 123.3, 119.7 ppm. IR (ATR): ν = 1736 (C=O, ester) cm^{-1}. HRMS (ESI+) *m*/*z*: calcd. for $C_{19}H_{11}BrNaO_2$ $[M + Na]^+$: 372.9835, found: 372.9832.

2-Methyl-10*H*-tribenzo[*b,d,f*]oxocin-10-one (2t)

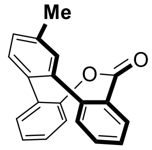

The title compound was synthesized according to the general procedure for organocatalytic esterification (reaction time: 24 h), using aldehyde **1t** (28.8 mg, 0.1 mmol). The crude product was purified by column chromatography (hexane/EtOAc – 9:1), affording **2t** (28.0 mg, 98%) as a white amorphous solid. *Er* = 96:4 (*ee* = 92%), the enantiomeric excess of product **2t** was determined by HPLC on a Chiralpak® IB column (*n*-heptane/*i*-PrOH - 80:20, flow rate = 1.0 ml/min, λ = 190 nm, t = 25 °C): t_R = 5.5 min (major), t_R = 7.6 min (minor). $[\alpha]_D^{20}$ = -46.6 (c = 1.2, $CHCl_3$). ^1H NMR (400 MHz, dichloromethane-d_2): δ 7.48 – 7.40 (m, 2H), 7.37 (ddt, J = 7.5, 6.2, 1.1 Hz, 2H), 7.34 – 7.26 (m, 2H), 7.24 – 7.13 (m, 5H), 2.47 (s, 3H) ppm. $^{13}C\{^1H\}$ NMR (101 MHz, dichloromethane-d_2): δ 169.7, 151.0, 139.5, 139.2, 138.8, 134.8, 133.3, 132.3, 131.3, 131.1, 130.7, 130.1, 130.0, 129.5, 128.9, 128.3, 127.4, 126.8, 121.4, 21.3 ppm. IR (ATR): ν = 1728 (C=O, ester) cm^{-1}. HRMS (ESI+) *m*/*z*: calcd. for $C_{20}H_{14}NaO_2$ $[M + Na]^+$: 309.0886, found: 309.0884.

3-Methyl-10*H*-tribenzo[*b,d,f*]oxocin-10-one (2u)

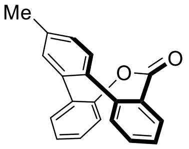

The title compound was synthesized according to the general procedure for organocatalytic esterification (reaction time: 18 h), using **1u** (28.8 mg, 0.1 mmol). The crude product was purified by column chromatography (hexane/EtOAc – 9:1), affording **2u** (27.9 mg, 98%) as a white amorphous solid.

Er = 95:5 (*ee* = 89%), the enantiomeric excess of product **2u** was determined by high-performance liquid chromatography (HPLC) on a Chiralpak® IB column (*n*-heptane/*i*-PrOH - 80:20, flow rate = 1.0 ml/min, λ = 221 nm, t = 25 °C): t_R = 6.0 min (minor), t_R = 6.3 min (major). $[\alpha]_D^{20}$ = -80.0 (c = 1.2, $CHCl_3$). ^1H NMR (400 MHz, dichloromethane-d_2): δ 7.47 – 7.39 (m, 2H), 7.38 – 7.23 (m, 5H), 7.23 – 7.16 (m, 3H), 7.14 (dd, J = 7.6, 1.3 Hz, 1H), 2.48 (s, 3H) ppm. $^{13}C\{^1H\}$ NMR (101 MHz, dichloromethane-d_2): δ 169.7, 150.9, 139.5, 138.7, 136.8, 136.0, 135.0, 132.4, 131.3, 131.0, 130.2, 129.88, 129.85, 129.63, 129.59, 128.2, 127.5, 126.9, 121.4, 21.3 ppm. IR (ATR): ν = 1736 (C=O, ester) cm^{-1}. HRMS (ESI+) *m*/*z*: calcd. for $C_{20}H_{14}NaO_2$ $[M + Na]^+$: 309.0886, found: 309.0885.

6,13-Dimethyl-10*H*-tribenzo[*b*,*d*,*f*]oxocin-10-one (2v)

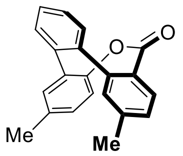

The title compound was synthesized according to the general procedure for organocatalytic esterification (reaction time: 28 h), using aldehyde **1v** (30.2 mg, 0.1 mmol). The crude product was purified by column chromatography (hexane/EtOAc – 10:1), affording **2v** (29.5 mg, 98%) as a white amorphous solid.

Er = 90:10 (*ee* = 79%), the enantiomeric excess of product **2v** was determined by HPLC on a Chiralpak® IG column (*n*-heptane/*i*-PrOH - 90:10, flow rate = 1.0 ml/min, λ = 190 nm, *t* = 25 °C): t_R = 11.5 min (minor), t_R = 14.3 min (major). $[\alpha]_D^{20}$ = -22.4 (*c* = 1.4, $CHCl_3$). ^{1}H NMR (400 MHz, dichloromethane-*d*$_2$): δ 7.53 (dqd, *J* = 7.4, 5.6, 2.7 Hz, 2H), 7.39 (ddt, *J* = 18.4, 8.7, 3.2 Hz, 3H), 7.25 – 7.14 (m, 1H), 7.14 – 6.99 (m, 3H), 6.97 (q, *J* = 4.3 Hz, 1H), 2.33 (s, 3H), 2.29 (s, 3H) ppm. ^{13}C{^{1}H} NMR (101 MHz, dichloromethane-*d*$_2$): δ 170.1, 148.9, 142.0, 139.8, 138.7, 136.8, 136.3, 134.5, 131.4, 131.0, 130.2, 130.1, 129.5, 129.2, 129.1, 128.92, 128.90, 127.8, 121.1, 21.5, 20.9 ppm. IR (ATR): ν = 1730 (C=O, ester) cm^{-1}. HRMS (ESI+) *m*/*z*: calcd. for $C_{21}H_{16}NaO_2$ [M + Na]$^+$: 323.1043, found: 323.1041.

6,13-Difluoro-10*H*-tribenzo[*b*,*d*,*f*]oxocin-10-one (2w)

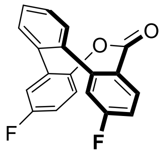

The title compound was synthesized according to the general procedure for organocatalytic esterification (reaction time: 24 h), using aldehyde **1w** (31.0 mg, 0.1 mmol). The crude product was purified by column chromatography (hexane/EtOAc – 10:1), affording **2w** (30.2 mg, 98%) as a white amorphous solid.

Er = 91:9 (*ee* = 82%), the enantiomeric excess of product **2w** was determined by high-performance liquid chromatography (HPLC) on a Chiralpak® IG column (*n*-heptane/*i*-PrOH - 90:10, flow rate = 1.0 ml/min, λ = 221 nm, *t* = 25 °C): t_R = 7.9 min (minor), t_R = 8.6 min (major). $[\alpha]_D^{20}$ = -24.3 (*c* = 1.1, $CHCl_3$). ^{1}H NMR (400 MHz, dichloromethane-*d*$_2$): δ 7.58 (tt, *J* = 7.5, 5.7 Hz, 2H), 7.49 (dd, *J* = 8.6, 5.6 Hz, 1H), 7.46 – 7.36 (m, 2H), 7.17 (dd, *J* = 8.9, 4.8 Hz, 1H), 7.09 (td, *J* = 8.4, 2.6 Hz, 1H), 7.00 (ddd, *J* = 8.8, 7.9, 3.1 Hz, 1H), 6.95 (dd, *J* = 8.5, 3.1 Hz, 1H), 6.89 (dd, *J* = 9.3, 2.6 Hz, 1H) ppm. ^{13}C{^{1}H} NMR (101 MHz, dichloromethane-*d*$_2$): δ 168.3, 163.7 (d, *J* = 252.2 Hz), 160.2 (d, *J* = 246.2 Hz), 146.6 (d, *J* = 3.0 Hz), 140.8 (d, *J* = 8.7 Hz), 135.9 (d, *J* = 8.7 Hz), 134.7 (d, *J* = 1.6 Hz), 130.0, 129.9, 129.7, 129.5, 129.2, 128.5, 127.9 (d, *J* = 3.5 Hz), 122.7 (d, *J* = 9.0 Hz), 117.1 (d, *J* = 13.5 Hz), 116.9 (d, *J* = 12.0 Hz), 116.0 (d, *J* = 23.5 Hz), 115.4 (d, *J* = 22.1 Hz) ppm. ^{19}F NMR (376 MHz, dichloromethane-*d*$_2$): δ -108.34 – -108.90 (m, 1F), -115.97 (td, *J* = 8.2, 4.8 Hz, 1F) ppm. IR (ATR): ν = 1738 (C=O, ester) cm^{-1}. HRMS (ESI+) *m*/*z*: calcd. for $C_{19}H_{10}F_2NaO_2$ [M + Na]$^+$: 331.0541, found: 331.0540.

13-Fluoro-6-methyl-10*H*-tribenzo[*b*,*d*,*f*]oxocin-10-one (2x)

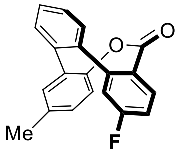

The title compound was synthesized according to the general procedure for organocatalytic esterification (reaction time: 24 h), using **1x** (30.6 mg, 0.1 mmol). The crude product was purified by column chromatography (hexane/EtOAc – 10:1), affording **2x** (29.7 mg, 98%) as a white amorphous solid.

Er = 88:12 (*ee* = 76%), the enantiomeric excess of product **2x** was determined by HPLC on a Chiralpak® IG column (*n*-heptane/*i*-PrOH - 90:10, flow rate = 1.0 ml/min, λ = 190 nm, *t* = 25 °C): t_R = 8.6 min (minor), t_R = 10.0 min (major). $[\alpha]_D^{20}$ = -21.5 (*c* = 1.3, $CHCl_3$). ^{1}H NMR (400 MHz, dichloromethane-*d*$_2$): δ 7.61 – 7.50 (m, 2H), 7.48 (dd, *J* = 8.6, 5.6 Hz, 1H), 7.43 (dd, *J* = 7.4, 1.6 Hz, 1H), 7.40 – 7.34 (m, 1H), 7.14 – 7.04 (m, 3H), 7.03 (d, *J* = 2.1 Hz, 1H), 6.87 (dd, *J* = 9.3, 2.6 Hz, 1H), 2.29 (s, 3H) ppm. ^{13}C{^{1}H} NMR (101 MHz, dichloromethane-*d*$_2$): δ 169.2, 164.0 (d, *J* = 251.8 Hz), 148.7, 141.6 (d, *J* = 8.6 Hz), 138.5 (d, *J* = 1.7 Hz), 137.0, 136.3, 134.1, 131.5, 130.34, 130.28, 130.2, 129.9, 129.7, 129.1, 128.7 (d, *J* = 3.5 Hz), 121.1, 117.2 (d, *J* =

22.2 Hz), 115.6 (d, J = 21.9 Hz), 20.9 ppm. ^{19}F NMR (376 MHz, dichloromethane-d_2): δ -108.99 (td, J = 8.8, 5.7 Hz, 1F) ppm. IR (ATR): ν = 1726 (C=O, ester) cm^{-1}. HRMS (ESI+) m/z: calcd. for $C_{20}H_{13}FNaO_2$ $[M + Na]^+$: 327.0792, found: 327.0790.

Unsuccessful starting material derivatization and follow-up transformations

As an access to nitrogen-containing strating material analogue, we tested the substitution reaction of 2-bromopropanamide with phenol, which resulted in quantitative formation of the expected intermediate, which we subjected to the Smiles rearrangement (KOH, DMSO, 100 °C). Unfortunately, we observed only decomposition of the starting material without formation of the expected product. The second approach is based on a coupling reaction with various nitrogen sources. At the outset, we successfully transformed the phenolic oxygen to an OTf group under standard conditions in quantitative yield. On the other hand, coupling reactions using $BocNH_2$ as well as benzophenone imine did not provide the expected N-protected intermediates.

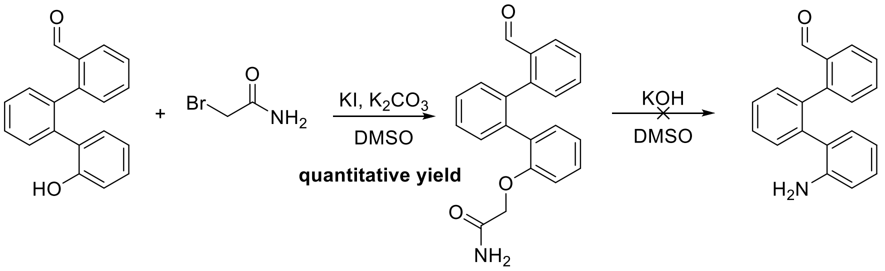

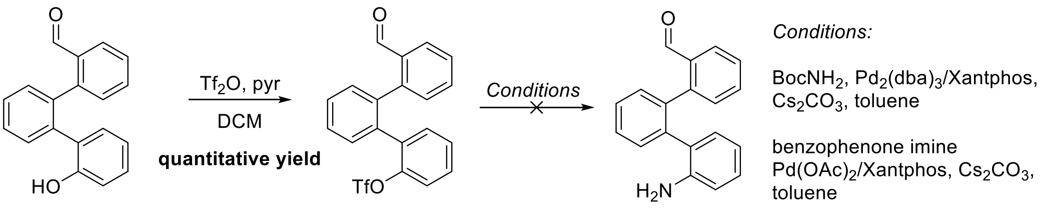

Additionally, we tested the transformation of **1a** to the sulfur-containing analogue. We began with reaction of **1a** with dimethylthiocarbamoyl chloride, which resulted in the formation of the expected intermediate in moderate yield. This intermediate was directly subjected to a thermally-induced Newman-Kwart rearrangement yielding the expected product in moderate yield. Unfortunately, our attempts to hydrolyse the intermediate resulted in decomposition to a complex mixture without significant formation of the expected product.

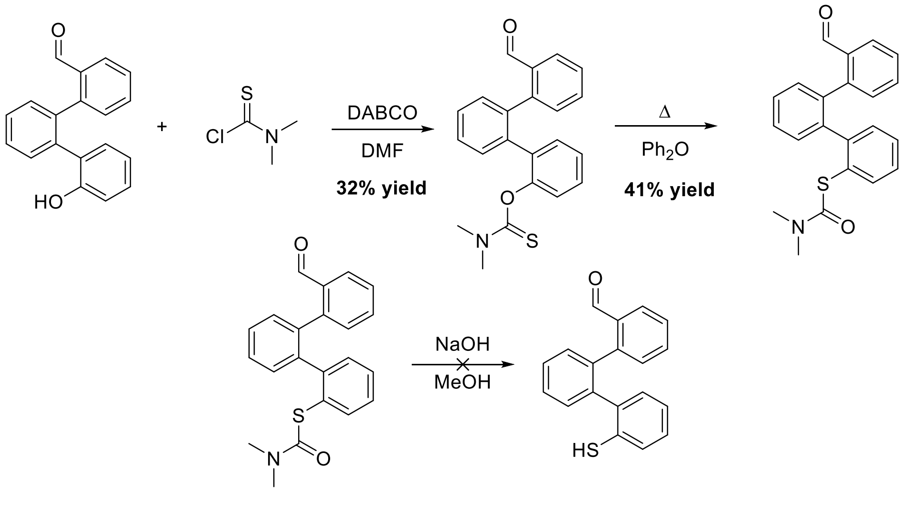

We performed several attempts for electrophilic aromatic substitution reactions such as bromination, iodination or nitration. None of these reactions provided desired product. Briefly, brominations and iodination did not provide any conversion of the starting material and the starting material was decomposed during nitration.

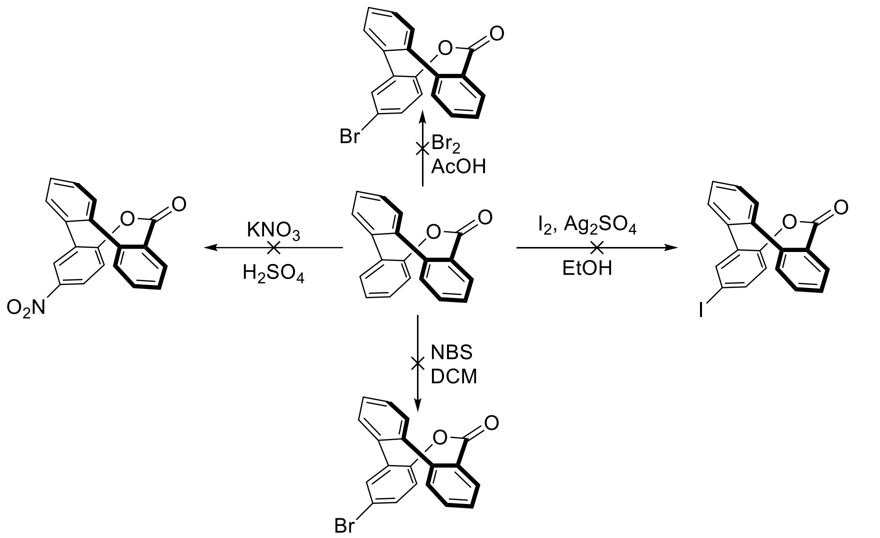

Crystallographic data

For **2a**, the diffraction experiment was performed on Bruker D8 VENTURE Kappa Duo PHOTONIII using IμS micro-focus sealed tube CuKα (λ= 1.54178 Å). The structure was solved by direct methods (XT)[4] and refined by full matrix least squares based on F^2 (SHELXL2019).[5] The hydrogen atoms on carbon were fixed into idealized positions (riding model) and assigned temperature factors $H_{iso}(H) = 1.2\ U_{eq}$(pivot atom). The determination of absolute structure was based of anomalous dispersion of oxygen atoms. Absolute structure parameter: 0.03 (5).[6]

Crystal data for **2a**, $C_{19}H_{12}O_2$, M_r = 272.29; Monoclinic, *C*2 (No 5), *a* = 16.6502 (7) Å, *b* = 7.3452 (3) Å, *c* = 21.9150 (9) Å, *β* = 97.814 (1)° , *V* = 2655.30 (19) Å^3, Z = 8, D_x = 1.362 Mg m^{-3}, temperature of sample 100(2) K, colourless prism of dimensions 0.51 × 0.28 × 0.13 mm, multi-scan absorption correction (μ = 0.70 mm^{-1}), T_{min} = 0.718, T_{max} = 0.913; a total of 27614 measured reflections (θ_{max}= 79.5°), from which 5527 were unique (R_{int} = 0.018) and 5524 observed according to the $I > 2\sigma(I)$ criterion. The refinement converged ($\Delta/\sigma_{max} < 0.001$) to *R* = 0.030 for observed reflections and w$R(F^2)$ = 0.080, *GOF* = 1.04 for 379 parameters and all 5527 reflections. The final difference map displayed no peaks of chemical significance ($\Delta\rho_{max}$ = 0.19, $\Delta\rho_{min}$ -0.19 e.Å^{-3})

X-ray crystallographic data have been deposited with the Cambridge Crystallographic Data Centre under deposition number CCDC 2466579 and can be obtained free of charge from the Centre via its website (https://www.ccdc.cam.ac.uk/structures/).

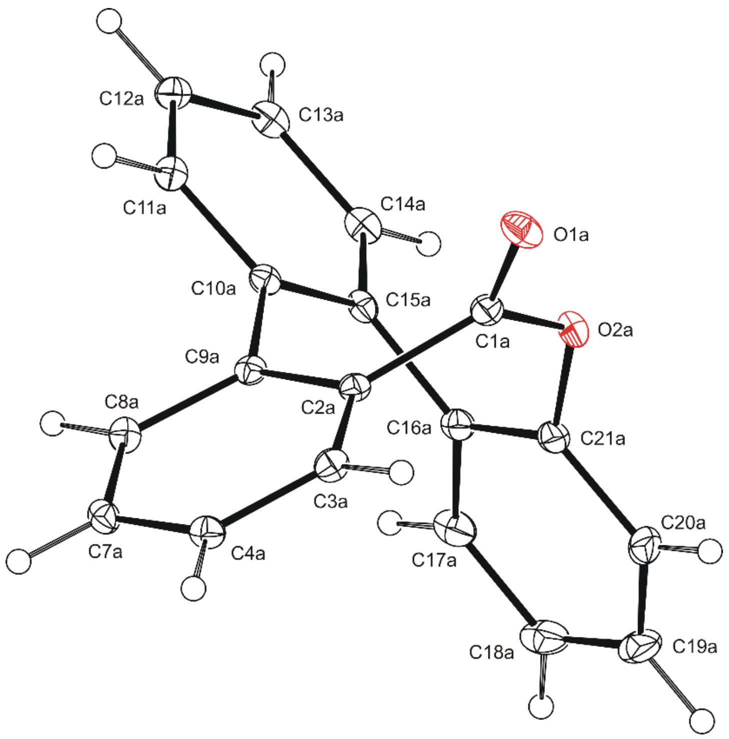

Figure S2. View on one of two symmetrically independent molecule of **2a** with atom numbering schema displaying. Displacement ellipsoids are drawn on 30% probability level (CCDC: 2466579).

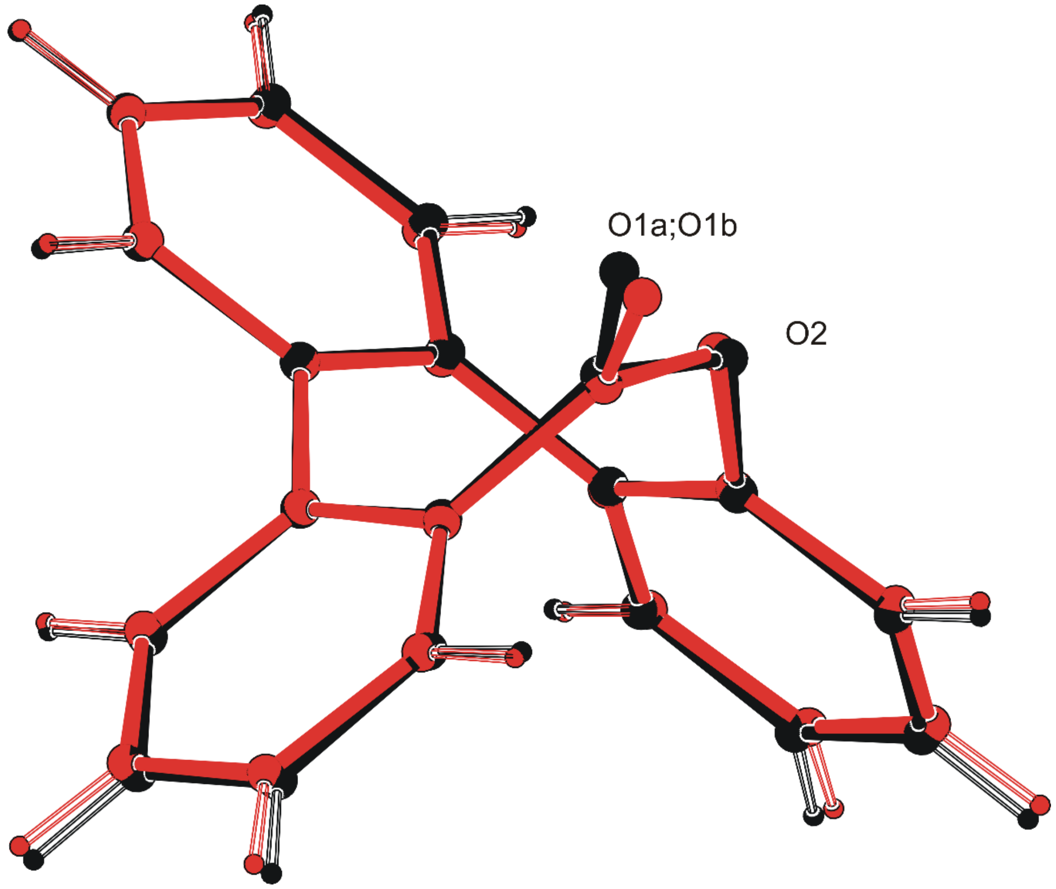

Figure S3. Results of overlap of symmetrically independent molecules A and B. Maximal distance is between O1a – O1b: 0.249 Å.

Computational methods

For our preliminary DFT calculations we used Orca 6.0.1,[7] Turbomole7.8,[8] xTB 6.6.1[9] and Crest 2.12,[10] software. Geometry optimizations and characterization of stationary points by calculation of vibrational frequencies were performed at B3LYP(D3BJ)/def2-TVZP level of theory (298.15 K), employing an implicit description of dichloromethane solvent using conductor-like PCM (CPCM) model as implemented in Orca. Except for the Nudged Elastic Band (NEB) calculations,[11] for each compound as well as transition states, relevant conformers were generated by the modified Crest/xTB procedure; geometries were optimized at the GFN-2 level with dichloromethane solvent modeled by the ALPB implicit solvation model After that, DFT single point calculations were performed on these geometries with the BP86. density functional (as implemented Turbomole, with RI approximation) in dichloromethane modeled by COSMO implicit solvent model, as implemented in Turbomole (with FINE cavity,[12] dgauss-dzvp basis,[13] and modified D3-BJ dispersion correction (parameters: s6 = 1.0, a1 = 0.7182, a2 = 3.8572, and s8 = 3.2176).[14] The conformational screening of transition states was done with fixed bond (phenolate oxygen atom and carbon atom of the acyl azolium), the distance for each transition state was determined by initial optimization of random conformer of respective transition state. The resulting 10 most stable conformers were then reoptimized at B3LYP(D3BJ)/def2-TVZP level and the energetically lowest conformer was chosen as the optimal one. All calculations assumed the singlet state of the molecules. The obtained Gibbs free energies were corrected to 1M standard state (referred as “free_energy” in the xyz file) by adding R*298.15 K*ln((R*298.15 K/(0.001 m³))/(101325 Pa))/4184/627.509 ~ 0.003 hartree/mol . The structures were visualized with ChimeraX (https://www.cgl.ucsf.edu/chimerax/) with Seqcrow plugin (https://github.com/QChASM/SEQCROW)

The racemization barrier of the saddle-shaped ester **R1 (2a)** (Figure S4) was calculated using the Nudged Elastic Band (NEB) method (as implemented in Orca) using 40 images, B3LYP(D3BJ)/def2-TVZP level of theory and a CPCM description of dichloromethane solvent. Then, the stationary points (**INT1**, **TS1**, and **TS2**) were obtained from the resulting minimum energy path (MEP) and reoptimized.

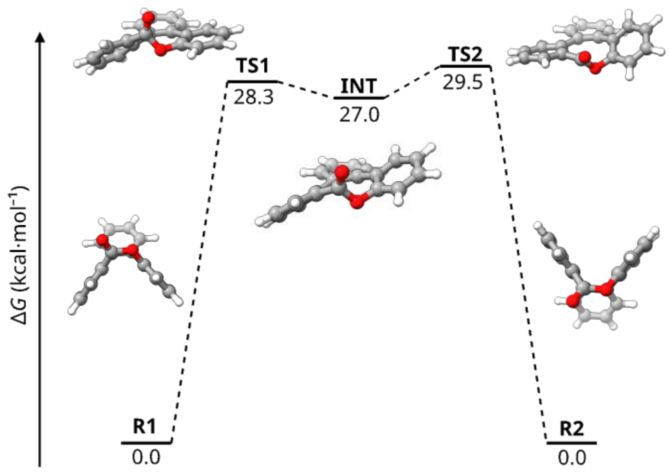

Figure S4. Reaction scheme showing the isomerization of saddle-shaped ester. The stationary points were obtained with NEB method.

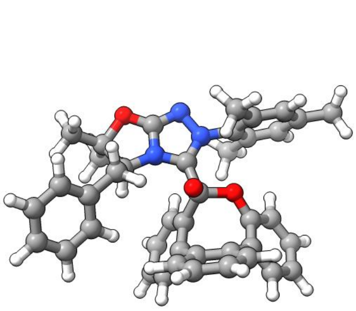

I2LR

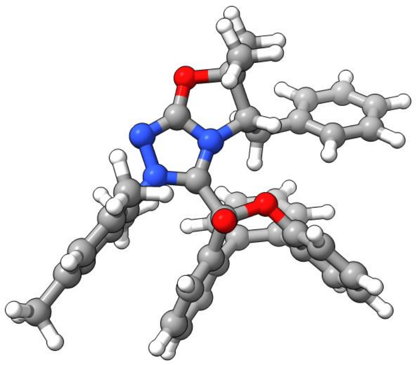

I2PR

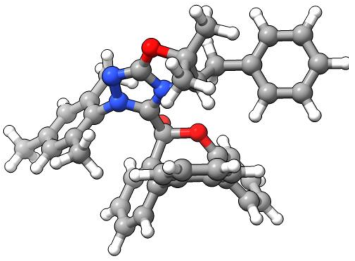

I2LS

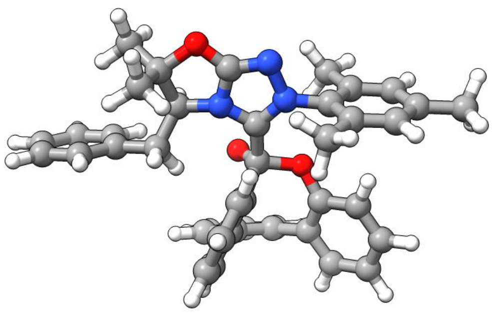

I2PS

Figure S5 Structure of the I2L and I2P series of intermediates. Visualized with ChimeraX.

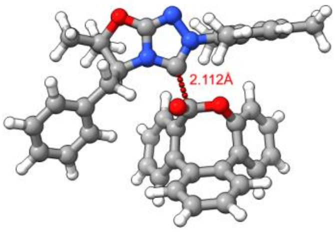

I2LRTS

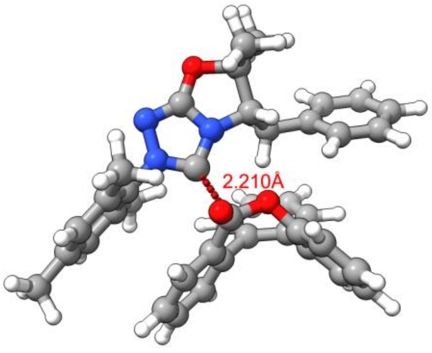

I2PRTS

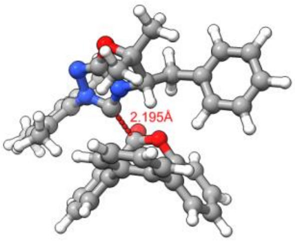

I2LSTS

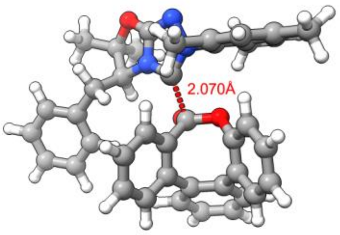

I2PSTS

Figure S6. Structure of the **I2L** and **I2P** series of transition states. Visualized with ChimeraX.

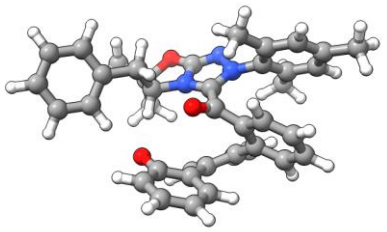

I1

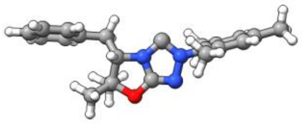

C

Figure S7. Structure of the phenolate **I1** and NHC catalyst **C**. Visualized with ChimeraX**.**

XYZ of stationary points

33
R1 charge=0 multiplicity=1 free_energy=-881.38670839
H 3.973555285 -1.171204731 -1.658804317
H 2.613775691 -1.562443980 0.361199829
O -1.134764849 1.996410960 -0.280429372
C -1.585804235 0.771352653 -0.784610891
C -2.233400789 0.784534934 -2.008558056
C -2.798572035 -0.389662787 -2.489147843
C -2.719526714 -1.555221541 -1.733951609
C -2.068043780 -1.546077698 -0.507632039
C -1.481498675 -0.380296863 -0.011101314
C -0.762822561 -0.375679778 1.281985984
C -1.439098028 -0.706738498 2.456347459
C -0.773975468 -0.748063998 3.674093064
C 0.586690809 -0.467153204 3.727153732
C 1.271854054 -0.151534766 2.561917006
C 0.611425707 -0.096631208 1.333359710
C 1.392913270 0.174801540 0.099907263
C 1.169980834 1.300371113 -0.702626762
C 0.175046055 2.343563692 -0.321631037
O 0.473617957 3.479915285 -0.056844329
C 1.955978706 1.539262519 -1.828616663
C 2.953649310 0.645169598 -2.186593283
C 3.190049425 -0.472862724 -1.394107614
C 2.423795749 -0.695614174 -0.258334357
H 3.549408796 0.825885430 -3.071554120
H -2.296730990 1.710449185 -2.564409215
H -3.302862140 -0.390363130 -3.446516828
H -3.161842960 -2.472110783 -2.101045593
H -1.994580729 -2.454576766 0.076202534
H -2.497630487 -0.928273002 2.408000439
H -1.316080107 -0.998193614 4.576968328
H 1.114034008 -0.495552532 4.672003718
H 2.333125199 0.058793322 2.597534565
H 1.778333690 2.427745547 -2.420158390
33
R2 charge=0 multiplicity=1 free_energy=-881.38672659
H -3.171190918 -1.895413329 -2.499755510
H -2.048929297 -2.258139775 -0.334092572
O 0.511449518 2.247332393 0.201570177
C 1.371583450 1.304223042 -0.372152103
C 2.144537617 1.714798938 -1.445428037

C 3.093645024 0.846364650 -1.968835843
C 3.265174434 -0.413285393 -1.403967874
C 2.481889556 -0.804241326 -0.326055852
C 1.512800351 0.047604333 0.207801236
C 0.658084735 -0.377708833 1.338165797
C 1.237488723 -0.715657504 2.561234165
C 0.457183160 -1.154859363 3.622071479
C -0.919203647 -1.271488432 3.464577556
C -1.503759557 -0.952548626 2.246466421
C -0.730582842 -0.499695556 1.176192865
C -1.382300467 -0.235237306 -0.132024199
C -1.398551406 1.034217832 -0.722853438
C -0.824042159 2.223044541 -0.029776376
O -1.483227069 3.165731225 0.327135110
C -2.056785592 1.248482557 -1.932659211
C -2.680722724 0.195307217 -2.584184240
C -2.677810881 -1.068865806 -2.004714976
C -2.044500750 -1.275095291 -0.786775880
H -3.175003980 0.362853046 -3.532017713
H 2.003430987 2.707739992 -1.851022206
H 3.699249091 1.156545125 -2.810190525
H 4.006166096 -1.092658095 -1.804544286
H 2.605431535 -1.788215960 0.107686208
H 2.310190947 -0.626629431 2.676419440
H 0.922176317 -1.404393403 4.567150166
H -1.536195592 -1.612145234 4.286112968
H -2.573901855 -1.051309558 2.116445582
H -2.073772804 2.243343331 -2.357978328
33
TS1 charge=0 multiplicity=1 free_energy=-881.34161895
H -3.415469371 -3.376184600 1.649092889
H -2.295976554 -2.530810380 -0.351783514
O 1.009845259 -0.066592925 1.582840025
C 1.936748787 0.517454426 0.748668041
C 3.146719933 0.890357784 1.310855479
C 4.133440664 1.444114897 0.516057563
C 3.858512703 1.626107248 -0.832817800
C 2.638193909 1.244614455 -1.366272766
C 1.609716815 0.641788798 -0.606962636
C 0.289145735 0.255222034 -1.254124150
C 0.269384437 0.482446711 -2.648375637
C -0.840153907 0.391255545 -3.463153187
C -2.057897738 0.066652792 -2.896115589
C -2.075375668 -0.257169759 -1.556038342
C -0.945547896 -0.254674569 -0.705925110
C -1.362128426 -0.860218972 0.619238368
C -1.197542752 -0.262404676 1.879507803
C -0.076537830 0.680790089 1.966687096
O -0.032681765 1.841149425 2.263846939
C -1.876977932 -0.693612110 3.008627103
C -2.705988891 -1.806056570 2.928686904
C -2.813388587 -2.479357420 1.719590720
C -2.166113224 -2.004145918 0.583442929
H -3.230213968 -2.160097718 3.806364925
H 3.286609642 0.735829746 2.372176139
H 5.084248866 1.738688917 0.939743181
H 4.595198814 2.075849012 -1.485652248
H 2.506461580 1.452369695 -2.412744277
H 1.180995393 0.750131899 -3.147712490
H -0.744006044 0.596114018 -4.521470576

H -2.971071834 0.041581585 -3.476061595
H -3.023390331 -0.534324314 -1.121418033
H -1.714759820 -0.186869147 3.951201847
33
TS2 charge=0 multiplicity=1 free_energy=-881.34041963
H -1.848586334 -2.765060825 3.983826741
H -2.361256340 -2.218527202 1.748201191
O 1.923201417 -0.570419089 -0.017015649
C 1.921223224 0.495695074 -0.909095166
C 3.101020182 1.110474694 -1.286560195
C 3.038353169 2.281010824 -2.030002584
C 1.796351121 2.825334289 -2.342918252
C 0.631102779 2.165317048 -1.981669974
C 0.662086492 0.948712595 -1.280576031
C -0.601482155 0.157610247 -1.138614809
C -1.313754737 0.202696922 -2.357308161
C -2.592577901 -0.279192356 -2.534826004
C -3.236358624 -0.806966544 -1.430813841
C -2.540907190 -0.929865152 -0.245452621
C -1.190947246 -0.556267069 -0.039894446
C -0.641899702 -0.970132811 1.320004774
C 0.588674926 -0.623796695 1.936174024
C 1.745837870 -0.042692257 1.253879971
O 2.515370960 0.753864561 1.721608302
C 0.862079996 -0.919867632 3.278777943
C -0.008114086 -1.656395782 4.053483080
C -1.160846447 -2.126907558 3.443825451
C -1.455515610 -1.789919104 2.133572271
H 0.224633376 -1.890704991 5.083380482
H 4.043498683 0.686774409 -0.968955181
H 3.949463077 2.781111330 -2.331238764
H 1.733427256 3.763377565 -2.878971799
H -0.324369867 2.596156576 -2.247011554
H -0.816867473 0.645205769 -3.207297671
H -3.074587272 -0.213387921 -3.501393936
H -4.266559011 -1.134919112 -1.479631609
H -3.111017177 -1.328867541 0.571063083
H 1.809322645 -0.589452261 3.681450936
33
INT1 charge=0 multiplicity=1 free_energy=-881.34366057
H -4.422258685 -2.545817419 -0.604779399
H -2.446878930 -1.801274349 -1.788131338
O 0.363083591 -0.684644161 1.778322463
C 1.536084910 0.010159701 1.545467287
C 2.425544102 0.215135130 2.584308199
C 3.544387536 1.006245381 2.373475666
C 3.712765727 1.609114788 1.132200456
C 2.812825499 1.369578891 0.105092515
C 1.707333976 0.508474287 0.254560427
C 0.847701762 0.215646553 -0.945868381
C 1.585265185 0.334898903 -2.144696045
C 1.032289193 0.405680301 -3.407108361
C -0.346937803 0.391766152 -3.519795431
C -1.092314487 0.138413547 -2.385933249
C -0.555401703 -0.075578284 -1.095334223
C -1.614971602 -0.644621117 -0.175482999
C -1.823838106 -0.378533309 1.191537924
C -0.716613884 0.151898159 1.986807518
O -0.687895847 1.108110512 2.710527147
C -2.981260039 -0.759800224 1.862848856

C -3.949283031 -1.513538922 1.218215108
C -3.717596335 -1.902382882 -0.093682643
C -2.584034050 -1.471023026 -0.769864437
H -4.842554515 -1.825663649 1.742492357
H 2.213669392 -0.228923211 3.547377510
H 4.249677457 1.180463775 3.175249695
H 4.545478375 2.278406253 0.959347510
H 2.964062765 1.886039691 -0.830177062
H 2.660106257 0.382034696 -2.072156862
H 1.672285715 0.504514278 -4.274146982
H -0.841159251 0.539283372 -4.471110007
H -2.164087728 0.096026814 -2.497915695
H -3.085475445 -0.500090630 2.908352477
84
I2PSTS charge=0 multiplicity=1 free_energy=-1973.65888345
C 5.372684162 4.340677088 1.443650548
C 1.003008532 2.581774621 3.222837514
C 4.082605622 -0.451185310 0.609386872
C -2.185518255 -1.996039977 3.755778616
C -1.354841265 -4.315936482 3.252000295
C -3.877920634 -5.012962773 -1.613043874
C -3.548437474 -3.665689227 -1.737520276
C -3.112326770 -5.832911667 -0.792119582
C -4.521751389 2.149308347 -2.320293782
C -4.087214873 3.457199053 -2.135883942
C -0.180833126 -1.373105298 -3.876560014
C 1.555559304 4.120307011 -3.207528359
C 0.771823671 -1.599654669 -2.892775166
C 2.390191926 3.697063781 -2.179274855
C -2.465354577 -3.145966946 -1.041549505
C -2.027403914 -5.307657896 -0.095544284
C -3.592625382 1.122361678 -2.420387946
C -2.726868123 3.727966036 -2.058864785
C -1.170142039 -0.421421457 -3.664473923
C 0.206530494 3.789143605 -3.178355486
C 0.700511886 -0.908195834 -1.689447849
C 1.871332625 2.949438579 -1.130759465
C 3.218487359 3.386772353 2.331680929
C 4.680674487 1.943406679 1.100244163
C -0.516248533 -3.369014398 0.523852890
C 4.413012437 3.199171401 1.636894946
C 2.292109684 2.361682237 2.485563297
C 3.781503767 0.885650977 1.225649912
C -1.693393062 -3.960168128 -0.208184125
C -2.226890005 1.382084849 -2.322628094
C -1.784442667 2.703218808 -2.145797607
C -1.235725436 0.291172545 -2.468709070
C -0.335496593 3.034573581 -2.136193312
C -0.311429959 0.009775214 -1.450014684
C 0.522277345 2.622011129 -1.109791989
C -0.921769874 -2.336276723 1.587744232
C 2.593023612 1.123255426 1.914093144
C 0.723602875 -0.322314522 1.181950238
C 0.723860420 -1.541082849 3.029315434
C -1.126281074 -2.837111313 3.065026612
C -0.546534937 0.682950150 -0.106817279
N 1.679539410 -0.695663179 3.256118163
N 0.140559098 -1.365335946 1.819934057
N 1.654627518 0.056460996 2.059824723
O -1.605649696 0.511130979 0.496290160

O 0.025423463 1.979671906 0.005061424
O 0.187591271 -2.515712111 3.741849861
H 6.378951243 3.980273698 1.228903837
H 5.411192869 4.979521266 2.327168695
H 5.057513976 4.965668957 0.602970313
H 0.150401941 2.384042262 2.571236187
H 0.937166130 3.609260505 3.577773587
H 0.920062041 1.913403342 4.082079998
H 3.776027457 -1.273974087 1.255577904
H 5.148793858 -0.545244487 0.406903188
H 3.552370487 -0.567119585 -0.339207564
H -3.159894410 -2.218216371 3.318309754
H -1.986551225 -0.931300873 3.625487924
H -2.216261613 -2.226340703 4.820486779
H -0.537376611 -4.906295252 2.843669671
H -2.280669597 -4.608473957 2.757290100
H -1.448887313 -4.536466987 4.315431410
H -4.721950308 -5.419955852 -2.155499558
H -4.132208138 -3.020160556 -2.381984315
H -3.357575245 -6.883004872 -0.692438502
H -5.579839100 1.929395904 -2.384999973
H -4.803606090 4.264982274 -2.056800414
H -0.145502525 -1.918891961 -4.810710715
H 1.950307411 4.704792809 -4.028563060
H 1.563046019 -2.321080981 -3.051345550
H 3.443405604 3.947827648 -2.188851158
H -2.209547869 -2.099508428 -1.153898389
H -1.434024351 -5.952873955 0.540583291
H -3.923952441 0.101421947 -2.563117622
H -2.384087928 4.746872016 -1.929657434
H -1.890602651 -0.204454225 -4.442962422
H -0.447469729 4.112103069 -3.978631700
H 1.428828817 -1.098248208 -0.915890440
H 2.998935128 4.358061339 2.759427695
H 5.609242532 1.778791650 0.566454992
H 0.108601559 -2.846460011 -0.198531961
H 0.102747668 -4.149483427 0.964518827
H -1.812554607 -1.805215744 1.264608111
H 2.499525704 2.621529507 -0.318020264
84
I2PRTS charge=0 multiplicity=1 free_energy=-1973.66752571
C -4.971367671 1.984224555 -4.743968823
C -3.219420648 3.357263829 -0.227430663
C -0.096421845 1.384011971 -3.670615411
C 1.521331017 3.470971708 3.084679850
C 3.737536688 2.844137906 2.069421946
C 4.533999023 -2.016793826 4.011779662
C 5.255147224 -1.374718319 3.011319548
C 3.149514819 -1.878790387 4.053477467
C 2.658075700 -2.036293503 -1.838370501
C 3.041251539 -2.781850148 -0.731727701
C -2.768351386 -2.066586940 -3.126935608
C -1.246096109 -4.722998226 2.617372154
C -3.668080391 -1.290752065 -2.410112597
C -1.967713644 -3.701005390 3.227839654
C 4.595296709 -0.599403124 2.063345433
C 2.495695702 -1.106141640 3.102425668
C 1.327066458 -1.677296707 -1.998668780
C 2.087808747 -3.138212998 0.209766552
C -1.492971378 -2.273625337 -2.623611090

C -0.362486230 -4.429526769 1.586498292
C -3.272565072 -0.743899027 -1.202018184
C -1.800350207 -2.386058075 2.810725980
C -4.018998654 2.690887759 -2.522586845
C -2.542068386 1.723607103 -4.138926146
C 2.486138527 0.324851686 1.030793033
C -3.820097783 2.146259495 -3.790167882
C -2.986433105 2.784182204 -1.596370782
C -1.474154620 1.806294437 -3.248993549
C 3.210423382 -0.454721641 2.095244564
C 0.353162932 -2.016547563 -1.054723211
C 0.752830664 -2.753932958 0.074719962
C -1.068999497 -1.727175037 -1.405379393
C -0.196833995 -3.117417694 1.141546620
C -1.991810295 -0.944818889 -0.684582228
C -0.919646820 -2.108050691 1.774142381
C 1.586138472 1.440894474 1.564330785
C -1.726739790 2.315142296 -1.976456389
C -0.381450581 1.337958076 -0.170619554
C 0.921978060 3.111773957 0.082800951
C 2.237097406 2.833089567 1.907351811
C -1.721017986 -0.235479627 0.611630136
N 0.140524461 3.482386733 -0.882239993
N 0.643828009 1.864495351 0.537974186
N -0.672176632 2.331982968 -1.012694847
O -2.573207546 0.410354300 1.197066200
O -0.672420095 -0.802416165 1.386333033
O 1.933279039 3.693321422 0.703950203
H -5.702163707 2.785296044 -4.624708894
H -5.489247729 1.038515422 -4.557500870
H -4.630809438 1.973252584 -5.779842335
H -3.043317682 2.600685147 0.538500756
H -4.244088270 3.715060109 -0.133829119
H -2.543165391 4.191329403 -0.028980014
H -0.148777727 0.619366289 -4.445255120
H 0.479820701 0.995588241 -2.834332773
H 0.455777659 2.235583570 -4.078041104
H 1.866772375 4.494656149 3.228025753
H 1.734031975 2.897610150 3.988247011
H 0.441402154 3.477534757 2.927852063
H 4.019554902 2.226911573 2.921640292
H 4.074392848 3.864228069 2.254911782
H 4.239115811 2.464147643 1.181634450
H 5.044623072 -2.621578318 4.750525528
H 6.332384977 -1.477238701 2.966958747
H 2.576586275 -2.381034267 4.823090087
H 3.388322420 -1.739365691 -2.580415237
H 4.074689479 -3.071476388 -0.592496029
H -3.051203054 -2.507564201 -4.074403970
H -1.372029081 -5.747132900 2.944201987
H -4.664353001 -1.105477155 -2.789905400
H -2.654611791 -3.925469518 4.033807894
H 5.163035614 -0.105279336 1.284097157
H 1.416561869 -1.027183852 3.133345151
H 1.029492008 -1.124531164 -2.877616071
H 2.380502746 -3.696666653 1.088928948
H -0.796057923 -2.883820754 -3.182817467
H 0.197844900 -5.222546111 1.107541815
H -3.952062989 -0.123855156 -0.636877088
H -5.006147858 3.039915236 -2.242024094

H -2.368794443 1.307802496 -5.124782639
H 1.825297799 -0.356516829 0.496600018
H 3.187696622 0.722757545 0.297913308
H 1.038115042 1.086712484 2.433476093
H -2.335435374 -1.573794969 3.283193490
84
I2LSTS charge=0 multiplicity=1 free_energy=-1973.66806049
C 5.973356139 -3.742319413 2.067336219
C 3.701747065 -1.033125893 -1.516613982
C 2.886503243 0.027795680 3.345602088
C -0.498156036 4.989142440 0.031203047
C 0.093850442 3.716472996 -2.050936143
C -5.429272841 3.550601926 0.935961561
C -4.574752298 4.303406047 1.733739621
C -4.937016027 2.430215045 0.272629575
C -0.654761879 0.690967546 -3.741281046
C -2.022849668 0.865767088 -3.578673637
C 1.385157171 -4.177471184 -1.882123676
C -4.614696094 -3.046571029 -0.176873905
C 1.667530174 -4.329764609 -0.532145532
C -4.166408884 -3.012039730 1.140549263
C -3.236839554 3.942527015 1.859426247
C -3.601615423 2.072435641 0.404915296
C 0.014467968 -0.256139661 -2.979247405
C -2.700531219 0.100605168 -2.640591230
C 0.613075088 -3.103453099 -2.301555210
C -3.931427362 -2.344221241 -1.161827212
C 1.161667677 -3.411378825 0.373181106
C -3.042479982 -2.265877028 1.474437422
C 4.833203014 -2.322836727 0.325845880
C 4.433513677 -1.832829281 2.636068491
C -1.289723493 2.410239983 1.340376779
C 5.069277613 -2.608532339 1.666826398
C 3.966466936 -1.304753791 -0.064593358
C 3.565399084 -0.801906050 2.293765761
C -2.731154314 2.827432728 1.194080741
C -0.653336328 -1.036048723 -2.031774116
C -2.033273690 -0.840672298 -1.855043733
C 0.099248581 -2.160874450 -1.404190574
C -2.788540409 -1.608409278 -0.848245555
C 0.377964710 -2.333012145 -0.035973497
C -2.366574707 -1.571447743 0.480331716
C -0.519277243 2.364940957 0.014273745
C 3.337435644 -0.568418002 0.935919546
C 1.097775546 0.334862405 0.453753142
C 1.803962276 2.410763133 0.143647225
C 0.081755743 3.715458666 -0.533873184
C -0.076356177 -1.415449286 1.065738649
N 2.909073791 1.777231947 0.377347762
N 0.714035987 1.601748344 0.166323696
N 2.420025892 0.464992012 0.570744604
O 0.219598065 -1.600989821 2.233529466
O -1.304457193 -0.745098879 0.791852572
O 1.525567912 3.679194562 -0.096950846
H 6.629748146 -4.034055304 1.247175166
H 5.385997783 -4.620341137 2.351327109
H 6.589075913 -3.473037524 2.927152413
H 4.433468925 -1.544215310 -2.141011152
H 3.735697303 0.033873361 -1.739587599
H 2.711554631 -1.393971944 -1.796204434

H 3.095970472 1.090278131 3.205492314
H 3.225560373 -0.262406563 4.339257054
H 1.804904962 -0.106210351 3.294042709
H 0.039848801 5.841550476 -0.383885163
H -0.422655562 5.022251255 1.115843814
H -1.546918787 5.072796450 -0.250438701
H 0.576358968 2.819700431 -2.437696448
H 0.621193762 4.595363176 -2.421462965
H -0.931613070 3.735417710 -2.421507643
H -6.470086632 3.831226691 0.835111654
H -4.947916147 5.173979728 2.258773867
H -5.594619727 1.829906967 -0.343903619
H -0.107949379 1.287916753 -4.459519513
H -2.557469028 1.598664096 -4.169121329
H 1.765971694 -4.883534685 -2.609049102
H -5.493698486 -3.622268586 -0.436551645
H 2.279188389 -5.152514172 -0.185267177
H -4.696905710 -3.557184130 1.910736473
H -2.576695487 4.536025630 2.480252261
H -3.235024689 1.191764658 -0.104282908
H 1.076580650 -0.401699915 -3.122975571
H -3.764322022 0.240101105 -2.496203274
H 0.398603891 -2.980935580 -3.355126282
H -4.272377481 -2.376471429 -2.188973715
H 1.380042708 -3.506636815 1.426888031
H -2.690687634 -2.205570949 2.495435324
H 5.327727028 -2.909535366 -0.439450884
H 4.619350033 -2.033551955 3.684973129
H -1.258967408 1.395992410 1.738127513
H -0.771981545 3.052587816 2.052850988
H -1.135114256 1.911584068 -0.758114472
84
I2LRTS charge=0 multiplicity=1 free_energy=-1973.65824966
C 6.449155160 1.160854520 2.730451332
C 4.050828808 -0.302484046 -1.450810436
C 2.021176936 3.234343462 1.513125171
C -1.757439287 2.419537745 -4.658218812
C -0.118031349 0.521799492 -4.839626067
C -6.049971983 -0.131255137 -2.736299401
C -5.826192012 1.212333912 -3.014742399
C -5.036617861 -0.888404898 -2.154663792
C -3.628101039 -0.732782978 4.165108136
C -2.639681971 -0.727580940 5.143047005
C -1.897844814 -3.564129864 -0.594111829
C 2.168872393 -3.513282583 3.562574085
C -0.867258182 -2.959677904 -1.300718409
C 3.005522101 -2.775617526 2.732353809
C -4.595641317 1.792024332 -2.719959272
C -3.811541685 -0.304621778 -1.859510468
C -3.309229839 -1.102667783 2.865495893
C -1.341414253 -1.096016139 4.814163412
C -2.283575626 -3.039503501 0.632643552
C 0.871497783 -3.074834741 3.798276100
C -0.270125574 -1.806737329 -0.804753169
C 2.538392424 -1.609903320 2.139737242
C 5.223384686 0.511343074 0.628013204
C 4.243396720 2.169578099 2.053304933
C -2.242144734 1.657962011 -1.788471043
C 5.289377895 1.289312121 1.781388113
C 4.135947196 0.581675781 -0.239023213

C 3.142350411 2.282935413 1.210007472
C -3.572045421 1.042900737 -2.143299400
C -2.005128489 -1.454393527 2.522194537
C -1.005406251 -1.457108990 3.509055738
C -1.673590236 -1.897630385 1.148651798
C 0.380048917 -1.907452679 3.211330771
C -0.689070465 -1.241068774 0.392498598
C 1.237599921 -1.186240202 2.372770055
C -1.064327567 1.030681823 -2.545037033
C 3.110175867 1.471553460 0.077014376
C 0.859077174 0.855869150 -0.736757291
C 0.975451066 2.151006991 -2.530804320
C -0.691047245 1.617156397 -3.955985084
C -0.210551735 0.104791160 0.922307641
N 2.103075847 2.391560635 -1.942655930
N 0.204608614 1.260731258 -1.856382543
N 1.990379336 1.558389209 -0.806478478
O -1.005240496 1.026769461 1.094042845
O 0.847364847 0.030355893 1.854753997
O 0.440620397 2.571221060 -3.663376741
H 6.692667882 2.119716375 3.190145509
H 7.336338938 0.781878232 2.222345943
H 6.207716190 0.463784225 3.538266746
H 3.229469322 -1.017032506 -1.349928345
H 4.975164845 -0.863219397 -1.581315577
H 3.863631450 0.276521774 -2.356368828
H 1.094650516 2.690193870 1.703313495
H 1.835115243 3.905472554 0.672036124
H 2.256538161 3.835974348 2.389971622
H -2.612433905 1.781225158 -4.877793600
H -1.360292349 2.800006554 -5.599467720
H -2.093269533 3.260033946 -4.054863486
H -0.910855494 -0.181270274 -5.098820262
H 0.671504606 -0.025833941 -4.322391636
H 0.287430463 0.949383504 -5.756274107
H -7.006358744 -0.584082301 -2.965516960
H -6.609173763 1.812044696 -3.462144646
H -5.202284943 -1.933334742 -1.923123644
H -4.643131437 -0.449338719 4.412946196
H -2.878226352 -0.441244701 6.159446671
H -2.383195622 -4.450828764 -0.981290619
H 2.523700275 -4.423467802 4.028599430
H -0.531654396 -3.373788318 -2.242739575
H 4.020654269 -3.102629451 2.546247477
H -4.428745867 2.839307481 -2.940036441
H -3.037777742 -0.901034495 -1.392912135
H -4.073740554 -1.109339049 2.098810424
H -0.571859134 -1.104475969 5.575752261
H -3.047415908 -3.534339046 1.218951895
H 0.217386779 -3.643987109 4.446834238
H 0.525282060 -1.338449612 -1.364178501
H 3.175405565 -1.009779736 1.507577894
H 6.030198953 -0.175719926 0.401106958
H 4.285886321 2.787750428 2.942570543
H -2.049285592 1.488099202 -0.728816027
H -2.257837427 2.737274069 -1.940582270
H -1.228288143 -0.038762725 -2.648983736
84
I2PS charge=0 multiplicity=1 free_energy=-1973.66935830
C -5.755494413 2.087346368 -3.860004873

C -3.343651397 2.411195522 0.545299615
C -0.743604220 1.692386875 -3.732783168
C 3.176779751 3.598110899 2.354186427
C 3.830495894 2.857850260 0.030688429
C 5.489308864 -0.172173019 4.439432795
C 4.118505576 -0.135296741 4.679798216
C 5.959899448 -0.057598950 3.136625810
C -0.412183349 -3.961614524 3.332717386
C -1.794022637 -4.048097753 3.203564619
C 2.260294682 -2.979640500 -1.712571570
C -3.597055444 -3.788818404 -2.215087376
C 2.053922495 -1.712665111 -2.240929675
C -3.665692691 -2.522867003 -2.786385730
C 3.228975784 0.023497371 3.625029719
C 5.065210412 0.104725224 2.083298741
C 0.347998304 -3.478961581 2.275835632
C -2.399625661 -3.667184490 2.012220381
C 1.510709663 -3.396266313 -0.618743692
C -2.928333852 -3.949129063 -1.010300421
C 1.120332216 -0.868079199 -1.652322747
C -3.065340246 -1.447754968 -2.150715221
C -4.483700371 2.235646266 -1.693236312
C -3.244754833 1.924010887 -3.718134504
C 2.716119714 0.330915663 1.177407434
C -4.469596238 2.078377527 -3.080427642
C -3.314521040 2.239258353 -0.945476117
C -2.042242181 1.909049891 -3.010144006
C 3.692093932 0.156072095 2.314058446
C -0.257263637 -3.061002005 1.092503306
C -1.646764946 -3.171572678 0.947480402
C 0.562070288 -2.559248294 -0.033520383
C -2.316383032 -2.879581001 -0.346404076
C 0.383202788 -1.267043075 -0.543147215
C -2.391801673 -1.605190503 -0.936054568
C 1.925597215 1.630299125 1.289034223
C -2.110566651 2.057150109 -1.629585604
C -0.215652645 1.175112239 -0.219848268
C 0.758291539 3.118262915 -0.051250682
C 2.724281605 2.961778628 1.062147245
C -0.540386787 -0.288326538 0.199896754
N -0.287379811 3.378264880 -0.772336588
N 0.864361407 1.809277204 0.284332052
N -0.889907005 2.118700019 -0.874153448
O -0.494885068 -0.298194424 1.489124608
O -1.946352047 -0.459098002 -0.353157190
O 1.693967103 3.887054450 0.455106193
H -5.584040338 1.844328451 -4.908345081
H -6.231994378 3.069961210 -3.812588078
H -6.464617243 1.365157793 -3.449787596
H -2.989024151 1.506798352 1.043058553
H -4.357310475 2.617628485 0.885175319
H -2.699685265 3.233784338 0.863255653
H -0.831387382 2.004575519 -4.772806370
H -0.478432871 0.632042616 -3.727349214
H 0.079013302 2.241652963 -3.276013854
H 3.917957746 2.956315229 2.830414836
H 2.337473127 3.728390102 3.036729740
H 3.634607568 4.567019833 2.154698513
H 3.478819888 2.386766848 -0.887784700
H 4.661593477 2.276954109 0.425407790

H 4.191985296 3.858870903 -0.202718230
H 6.183291746 -0.296003417 5.261100349
H 3.741650008 -0.236065507 5.689919004
H 7.023601650 -0.094824055 2.937405822
H 0.071199711 -4.266055299 4.252563794
H -2.397813018 -4.419990654 4.021762222
H 2.992010313 -3.645262135 -2.152542560
H -4.056413296 -4.640755473 -2.699577481
H 2.622729306 -1.375341871 -3.098025136
H -4.182423911 -2.370114780 -3.725899641
H 2.163569180 0.038459354 3.821764467
H 5.438505512 0.187943128 1.069929082
H 1.423798278 -3.400765377 2.372172395
H -3.473077516 -3.755685601 1.900079643
H 1.645949677 -4.393253346 -0.218180912
H -2.866717173 -4.931232667 -0.558009817
H 0.994051955 0.127332441 -2.055081457
H -5.430483532 2.362089660 -1.181178451
H -3.214799656 1.803598581 -4.794522873
H 1.989822309 -0.477783096 1.210062897
H 3.226257580 0.275599097 0.217749577
H 1.434203262 1.666341513 2.261167276
H -3.119127489 -0.457415875 -2.577072840
84
I2PR charge=0 multiplicity=1 free_energy=-1973.67962908
C 5.691638546 0.871412979 -4.333073214
C 1.923149280 3.598432890 -2.401177486
C 3.737391456 -0.383388118 0.121895254
C -1.556088132 4.066586812 2.059430487
C -1.280213333 2.685534102 4.144291617
C -4.935778784 -1.110863166 4.000116237
C -4.807437248 -0.730685042 2.667226511
C -3.831795160 -1.037170238 4.842195396
C 0.522030487 -3.196890282 2.299140748
C -0.836321764 -3.457027435 2.414265993
C 2.204362096 -2.667773352 -3.074751354
C -3.899522196 -2.703001465 -2.460884656
C 1.975886270 -1.480232247 -3.756070116
C -3.930074117 -1.370510799 -2.861390579
C -3.584968475 -0.283092817 2.183343315
C -2.610621549 -0.584837401 4.353398849
C 1.022592884 -2.649222020 1.123841917
C -1.682084963 -3.147562848 1.357801097
C 1.610659130 -2.866556841 -1.837744799
C -3.012172122 -3.106653427 -1.469556037
C 1.178974781 -0.508967631 -3.173281077
C -3.082551874 -0.441440591 -2.268338354
C 3.821526365 2.203626908 -3.299448592
C 4.666751217 0.307064966 -2.105742338
C -1.132947872 0.211542216 2.470216872
C 4.714811600 1.135821964 -3.221333807
C 2.865356698 2.433269694 -2.317260742
C 3.730425874 0.495368582 -1.093899199
C -2.471079214 -0.203909481 3.021216052
C 0.183720685 -2.315279330 0.059765478
C -1.195032873 -2.566624517 0.188099231
C 0.790686457 -1.899669057 -1.240835832
C -2.138929679 -2.192906620 -0.882986053
C 0.592972401 -0.684727520 -1.922023509
C -2.188066180 -0.853220292 -1.284763421

C -1.139962608 1.579184420 1.782338443
C 2.821389020 1.539975892 -1.244711882
C 0.578788074 1.224383296 -0.211518589
C 0.978082769 2.499300009 1.512105765
C -0.918890730 2.840869147 2.688577210
C -0.177089849 0.543781397 -1.387887997
N 2.092078699 2.548548837 0.850993104
N 0.039981294 1.715337904 0.919536810
N 1.815307977 1.723826047 -0.240981669
O -0.466593226 1.458376747 -2.245975005
O -1.393697956 0.032847118 -0.622555037
O 0.578763199 3.052511747 2.636548444
H 6.003844035 1.798173876 -4.816053674
H 5.232273092 0.240911040 -5.100242615
H 6.577396606 0.350959905 -3.967573159
H 0.890149171 3.249471996 -2.355565974
H 2.075116680 4.144529963 -3.331262345
H 2.084166064 4.288228984 -1.568900772
H 4.066759646 -1.389595546 -0.134004162
H 2.756307015 -0.449390244 0.583943570
H 4.425172231 0.015435226 0.872702739
H -2.641312356 3.965239210 2.102756300
H -1.263165009 4.175124610 1.013925833
H -1.264710100 4.962937622 2.606062159
H -2.347353021 2.486654832 4.236036833
H -1.054152684 3.611658206 4.672719070
H -0.731002697 1.870740687 4.610076533
H -5.887518407 -1.462994619 4.377494333
H -5.658535339 -0.791543001 2.000431610
H -3.919818578 -1.332183202 5.880464784
H 1.195942184 -3.427936099 3.114390555
H -1.239264061 -3.885942810 3.322662999
H 2.831499782 -3.439800019 -3.502766995
H -4.565063357 -3.424520600 -2.917255670
H 2.426370870 -1.304203317 -4.724613866
H -4.622327581 -1.050571128 -3.630223584
H -3.491319715 -0.014974459 1.138851640
H -1.753067371 -0.532870139 5.013314918
H 2.086478228 -2.488188299 1.025376275
H -2.745519354 -3.325952408 1.447565102
H 1.761957902 -3.804275631 -1.318468094
H -2.982848827 -4.142398806 -1.154234165
H 1.006358317 0.433680925 -3.670279473
H 3.866897975 2.874473205 -4.149358663
H 5.367398149 -0.515198183 -2.020978581
H -0.835869636 -0.511347152 1.714350903
H -0.371867664 0.189289367 3.250142653
H -2.038567714 1.709397035 1.189862158
H -3.106211831 0.601587839 -2.549502660
84
I2LS charge=0 multiplicity=1 free_energy=-1973.68056032
C 7.087759505 0.071349224 2.224639396
C 3.683550451 0.935123730 -1.371228898
C 2.439585991 1.526704681 3.495546181
C -3.061759018 3.862615609 -0.157775615
C -1.734475258 2.965189268 -2.093961631
C -6.633391714 0.217954151 0.692525270
C -5.604025992 -0.490892419 0.080267129
C -6.326354986 1.295274250 1.516166139
C -0.612059778 0.191337027 -4.007438027

C -1.888899829 -0.341302636 -3.893457319
C 3.325421002 -3.052769197 -1.552828535
C -2.247311589 -4.826066097 -0.180100201
C 3.550651178 -2.899396134 -0.191242622
C -1.979757674 -4.448780619 1.132713803
C -4.281109625 -0.121493253 0.288261804
C -5.000327695 1.663921996 1.718502465
C 0.390841137 -0.227116424 -3.140969419
C -2.154809248 -1.279602185 -2.903943588
C 2.203423149 -2.473988433 -2.127888845
C -1.993868440 -3.938905493 -1.220671903
C 2.648958474 -2.170225594 0.568162757
C -1.473718773 -3.183655343 1.409923108
C 5.332121479 0.541168730 0.479462778
C 4.743452560 0.791874894 2.787627517
C -2.521202880 1.343078859 1.347093598
C 5.696078693 0.471366506 1.818522819
C 4.040854789 0.889378904 0.085572582
C 3.446929017 1.153831961 2.448051487
C -3.961909598 0.964772068 1.106346386
C 0.138048659 -1.169363704 -2.144446334
C -1.158583559 -1.698183657 -2.023240824
C 1.287228217 -1.731225775 -1.373856501
C -1.461949304 -2.677858828 -0.961683606
C 1.520162215 -1.583433821 0.003631192
C -1.217157877 -2.301284865 0.364827776
C -1.746782521 1.605961268 0.051373912
C 3.116540269 1.162441877 1.089142583
C 0.705418048 0.744432446 0.608661229
C 0.225091618 2.835228115 0.225179880
C -1.872626239 3.040096457 -0.586481521
C 0.641234662 -0.767034504 0.975365073
N 1.496206531 2.869691905 0.477498596
N -0.297526677 1.582479088 0.286576821
N 1.778340155 1.526594310 0.723156484
O 0.865540550 -0.930455220 2.227707896
O -0.821077073 -1.021322076 0.585479015
O -0.647041515 3.768210884 -0.082716402
H 7.070395278 -0.852743465 2.807882958
H 7.548644749 0.838368786 2.851283463
H 7.723604807 -0.088261264 1.354079967
H 3.301733907 -0.032734030 -1.697160309
H 4.565857148 1.162167751 -1.968966796
H 2.919447677 1.681148089 -1.585382932
H 2.888991305 1.490295701 4.486899075
H 1.596168420 0.834916957 3.455872071
H 2.055986610 2.535817434 3.327756739
H -3.979632267 3.375215362 -0.483301465
H -3.006250700 4.844028944 -0.628793164
H -3.096859125 3.992140647 0.921508025
H -1.682164659 3.968600159 -2.515591815
H -2.600699245 2.450047442 -2.509935573
H -0.843015410 2.407879345 -2.379393011
H -7.664758094 -0.069093886 0.531086060
H -5.829982541 -1.336802088 -0.557008219
H -7.118728749 1.852218325 2.000602903
H -0.393655658 0.930443604 -4.767665048
H -2.676942710 -0.024002915 -4.564496872
H 4.015559814 -3.618221153 -2.166326948
H -2.650753620 -5.807752003 -0.392877113

H 4.424610667 -3.336742047 0.274675009
H -2.177530926 -5.136678772 1.945337910
H -3.492548550 -0.692195675 -0.183754978
H -4.769153749 2.508061408 2.357010485
H 1.390273047 0.174204382 -3.243521146
H -3.151377368 -1.690068287 -2.799773080
H 2.020292311 -2.598342227 -3.187588338
H -2.191999864 -4.229671088 -2.245053577
H 2.806308791 -2.021109410 1.626508495
H -1.286924976 -2.862574155 2.425035733
H 6.066154751 0.317382521 -0.285489816
H 5.020377117 0.767040946 3.835243683
H -2.019335847 0.515011814 1.845424935
H -2.456418940 2.208190410 2.006782912
H -1.991384887 0.858090506 -0.694964108
84
I2LR charge=0 multiplicity=1 free_energy=-1973.67008018
C 6.797056071 -2.399275362 0.089468676
C 3.022671773 -0.844649162 -2.862938326
C 3.589423334 1.121879850 1.741323063
C -1.362852443 4.911886962 -1.645737744
C -1.048969467 3.110861408 -3.377104650
C -5.738960622 3.291727589 0.816670485
C -4.881512228 4.329588944 1.164064789
C -5.208168586 2.091529437 0.352480751
C -2.636316466 -1.386374476 4.097536484
C -1.618256783 -2.213144149 4.558615631
C -3.312872858 -1.696914467 -1.656246232
C 0.631805666 -5.566365936 0.554688295
C -2.245807752 -1.202837399 -2.395779508
C 1.583421178 -5.004574314 -0.289770686
C -3.504774024 4.169405588 1.041535242
C -3.833014691 1.934994118 0.235163948
C -2.764772925 -1.149713795 2.735157889
C -0.751709524 -2.811983333 3.651558913
C -3.185937942 -1.836424722 -0.280294979
C -0.135615913 -4.732352594 1.353268418
C -1.080198865 -0.816845368 -1.745528005
C 1.740639937 -3.629259446 -0.324706827
C 4.918514490 -1.544136592 -1.354345902
C 5.176873252 -0.626339187 0.845230594
C -1.471792950 2.777054194 0.473992826
C 5.614209546 -1.501102970 -0.148274195
C 3.797543171 -0.750891130 -1.580486877
C 4.065186325 0.189898870 0.664446795
C -2.962627816 2.973788149 0.574333173
C -1.873257228 -1.714241323 1.826215764
C -0.864345420 -2.575095332 2.281657565
C -2.014064587 -1.461576048 0.376310808
C 0.005259308 -3.338213537 1.344313461
C -0.963319190 -0.905341979 -0.361285332
C 0.967007958 -2.784605927 0.478385922
C -1.007110926 2.417010881 -0.940267740
C 3.395781276 0.097525438 -0.553281787
C 0.971135882 0.711066514 -0.495003238
C 1.259623169 2.659022200 -1.435073270
C -0.737093730 3.578628149 -1.966949792
C 0.273673358 -0.361637010 0.376685892
N 2.464734949 2.184607237 -1.370610968
N 0.329605442 1.816666827 -0.924716009

N 2.253437992 0.936765016 -0.768584612
O 0.088331457 0.205885195 1.524288388
O 1.300081290 -1.468115244 0.402877668
O 0.761133242 3.782039155 -1.902539720
H 7.254660947 -2.708288518 -0.850642335
H 6.490004979 -3.304661669 0.621150861
H 7.553371748 -1.904055126 0.700019278
H 3.554388908 -1.460085408 -3.586973593
H 2.850225833 0.138469977 -3.303671718
H 2.044075106 -1.299028888 -2.687265813
H 4.234140713 1.055881483 2.616341802
H 2.566206142 0.880528121 2.039210603
H 3.587408043 2.157140403 1.392199278
H -2.448037646 4.820157292 -1.665442134
H -1.063551559 5.639990929 -2.399556701
H -1.058799227 5.273291803 -0.665891373
H -2.126488913 2.972694233 -3.475958785
H -0.560224752 2.159328631 -3.593314745
H -0.720328866 3.853917511 -4.103036589
H -6.810594872 3.414768498 0.909688604
H -5.282862844 5.266490173 1.530007399
H -5.865239960 1.272477261 0.087405246
H -3.325063075 -0.925834800 4.794651144
H -1.505139775 -2.402521319 5.618638875
H -4.232004126 -1.988685207 -2.148062585
H 0.488136157 -6.638418031 0.592348863
H -2.319720479 -1.109575365 -3.471682057
H 2.198382175 -5.632524873 -0.922455608
H -2.843410661 4.983540786 1.312489843
H -3.436676441 0.988561864 -0.110036280
H -3.547426693 -0.497661768 2.367847686
H 0.025166239 -3.477310581 4.007038587
H -3.997003497 -2.259823664 0.298794452
H -0.879231454 -5.161236056 2.013354763
H -0.260853352 -0.423923285 -2.334105983
H 2.481682454 -3.172777069 -0.965999636
H 5.249941388 -2.216113412 -2.137213682
H 5.713858694 -0.576853853 1.785315808
H -1.171386119 1.940500850 1.109927921
H -0.938819751 3.657469641 0.833473837
H -1.702582592 1.714348518 -1.389340275
84
I1 charge=0 multiplicity=1 free_energy=-1973.67487358
C -6.133919534 -2.928556269 1.831871679
C -2.164201344 -3.710649440 -1.134356158
C -1.676791896 -1.049686971 3.168234080
C 4.752768609 -1.310542041 0.701109011
C 3.530839647 -1.290398758 -1.498925774
C 5.971043658 3.076312956 2.509801176
C 5.147787551 3.247775577 1.401290030
C 5.707101832 2.044365037 3.404238136
C -0.230751692 -1.501170767 -4.022158671
C 0.870081948 -0.657520622 -4.152237034
C -4.752051581 0.701392926 -1.279150690
C -1.283885404 4.413154234 -1.608089146
C -4.563088417 1.103969350 0.039144809
C -0.114342750 4.945137793 -1.057127543
C 4.068229778 2.398036182 1.184792471
C 4.631507944 1.191214859 3.184110769
C -1.264346736 -1.129641853 -3.182225401

C 0.904766849 0.543814435 -3.468002010
C -3.665033805 0.343420482 -2.063429849
C -1.253443656 3.115493954 -2.096724245
C -3.285763943 1.107466038 0.572670461
C 1.045755857 4.197559737 -1.020124673
C -4.098518441 -3.252831952 0.388395502
C -3.860216156 -2.031327764 2.432057569
C 2.614085518 0.445258535 1.882902267
C -4.671720835 -2.735234191 1.547548721
C -2.762369980 -3.041919627 0.068656482
C -2.520755229 -1.778566104 2.158171621
C 3.802002051 1.356509398 2.075442525
C -1.221123864 0.058762748 -2.442973909
C -0.137745626 0.953276813 -2.619760126
C -2.368020821 0.346842993 -1.549138845
C -0.103806510 2.320371759 -2.059649679
C -2.200953789 0.689794757 -0.199124612
C 1.123242048 2.865309746 -1.528911421
C 2.444032075 -0.103142322 0.460462457
C -2.014405474 -2.249527101 0.942158352
C -0.150816908 -0.695933689 0.448079291
C 1.412518915 -2.192715454 0.483060008
C 3.414675506 -1.248411586 0.011271841
C -0.888834420 0.612760152 0.471463204
N 0.319676172 -2.892180879 0.627355956
N 1.179395233 -0.859242138 0.398416278
N -0.659643493 -1.921766520 0.606930227
O -0.398015326 1.512731733 1.118304303
O 2.226919716 2.207889919 -1.490953563
O 2.673726907 -2.522463143 0.419121660
H -6.718374064 -2.135800521 1.355390115
H -6.338389239 -2.890712308 2.902013596
H -6.492112554 -3.880248019 1.437904491
H -2.903274624 -3.797721875 -1.930239138
H -1.841092809 -4.720506199 -0.868001796
H -1.296399336 -3.184185193 -1.518917489
H -2.050423852 -1.254651583 4.170601939
H -1.713095589 0.032519964 3.029011694
H -0.631019923 -1.353676859 3.127777433
H 5.327371235 -0.417251312 0.460884797
H 5.299574694 -2.181958555 0.340791164
H 4.648622736 -1.378553145 1.781255203
H 2.550994220 -1.279400034 -1.975289443
H 4.073808373 -2.182468554 -1.809717317
H 4.076469440 -0.407563667 -1.833321721
H 6.810601918 3.739908386 2.675137814
H 5.344624799 4.049411487 0.699704602
H 6.340389304 1.899584605 4.270790188
H -0.279931079 -2.435862486 -4.565994226
H 1.691517366 -0.928827422 -4.803991050
H -5.747256253 0.688806677 -1.704868032
H -2.191383263 5.000233349 -1.664742644
H -5.405805510 1.400362693 0.649221270
H -0.113294494 5.955561112 -0.661790235
H 3.439109600 2.537842506 0.311710495
H 4.436580586 0.382505874 3.878824368
H -2.124477834 -1.773856459 -3.074320841
H 1.744137000 1.209797463 -3.592905189
H -3.812822304 0.094771550 -3.106163878
H -2.153839866 2.706853447 -2.537320853

H -3.125364745 1.391290707 1.603834016
H 1.954462870 4.616441651 -0.601685034
H -4.705292400 -3.844112433 -0.287220334
H -4.275866888 -1.669920550 3.364957620
H 1.701204506 0.999727390 2.106023525
H 2.662642658 -0.383657978 2.591965145
H 2.421815141 0.700123369 -0.285307789
51
C charge=0 multiplicity=1 free_energy=-1092.30253648
C 7.332200255 1.090402020 0.122690725
C 3.266522255 -0.790248980 2.424823725
C 2.934938255 0.850052020 -2.332781275
C -3.145689745 -2.458842980 0.208333725
C -2.430555745 -1.373115980 -1.952313275
C -6.601233745 1.078724020 0.838156725
C -6.180173745 1.159799020 -0.483821275
C -5.654694745 0.982112020 1.854412725
C -4.822179745 1.134479020 -0.786743275
C -4.300357745 0.959986020 1.546945725
C 5.263480255 0.150051020 1.206755725
C 5.106772255 0.930096020 -1.052489275
C -2.393136745 0.985359020 -0.105129275
C 0.770407255 0.521819020 0.345961725
C 5.876105255 0.712312020 0.087312725
C 3.913915255 -0.188293980 1.209676725
C 3.751833255 0.606220020 -1.095231275
C -3.865647745 1.027570020 0.221298725
C -1.728346745 -0.324174980 0.310793725
C 3.176206255 0.053203020 0.048317725
C 0.150974255 -1.534512980 -0.284416275
C -2.103483745 -1.619258980 -0.491543275
N 1.438238255 -1.603334980 -0.380524275
N -0.283291745 -0.314570980 0.121558725
N 1.790922255 -0.291016980 0.027270725
O -0.820482745 -2.411966980 -0.490293275
H 7.717811255 1.273649020 -0.880321275
H 7.932400255 0.305436020 0.586635725
H 7.481372255 2.000962020 0.709814725
H 2.467151255 -0.147622980 2.800548725
H 3.998069255 -0.931207980 3.219368725
H 2.815176255 -1.757147980 2.193397725
H 2.144609255 1.579454020 -2.141532275
H 2.446945255 -0.066153980 -2.671080275
H 3.563046255 1.226134020 -3.139180275
H -4.085221745 -1.906437980 0.247557725
H -2.835004745 -2.689593980 1.227255725
H -3.312821745 -3.388759980 -0.335744275
H -2.497068745 -2.330547980 -2.468534275
H -1.663380745 -0.766757980 -2.435090275
H -3.388384745 -0.864672980 -2.045900275
H -7.657126745 1.095853020 1.076671725
H -6.907153745 1.243022020 -1.282034275
H -5.972387745 0.927973020 2.888227725
H -4.502279745 1.201575020 -1.819427275
H -3.572569745 0.891520020 2.346888725
H 5.850382255 -0.031702980 2.099993725
H 5.570092255 1.359801020 -1.933139275
H -1.874046745 1.780609020 0.435279725
H -2.230961745 1.167095020 -1.166871275
H -1.931887745 -0.505321980 1.368190725

NMR spectra

2''-Hydroxy-[1,1':2',1''-terphenyl]-2-carbaldehyde (1a)

^{1}H NMR of **1a** (400 MHz, DMSO-d_6)

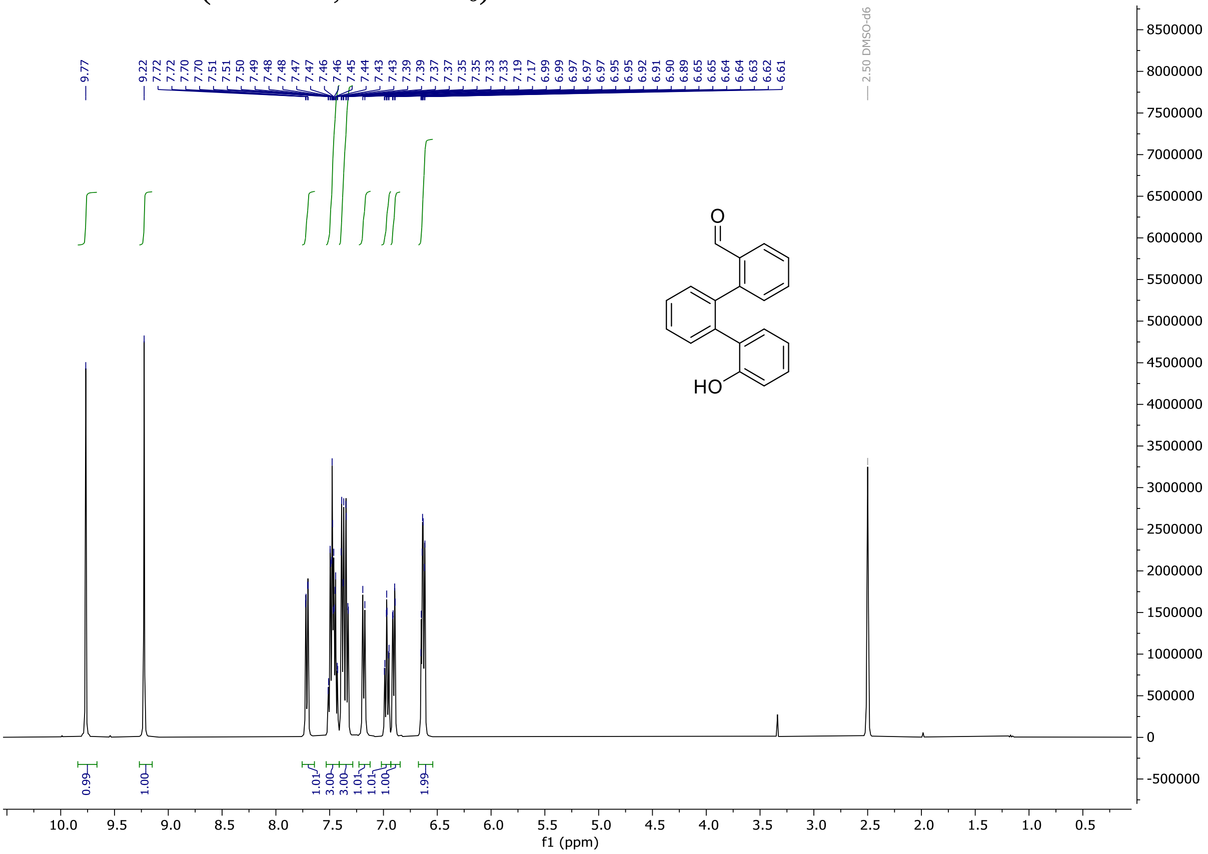

^{13}C{^{1}H} NMR of **1a** (101 MHz, DMSO-d_6)

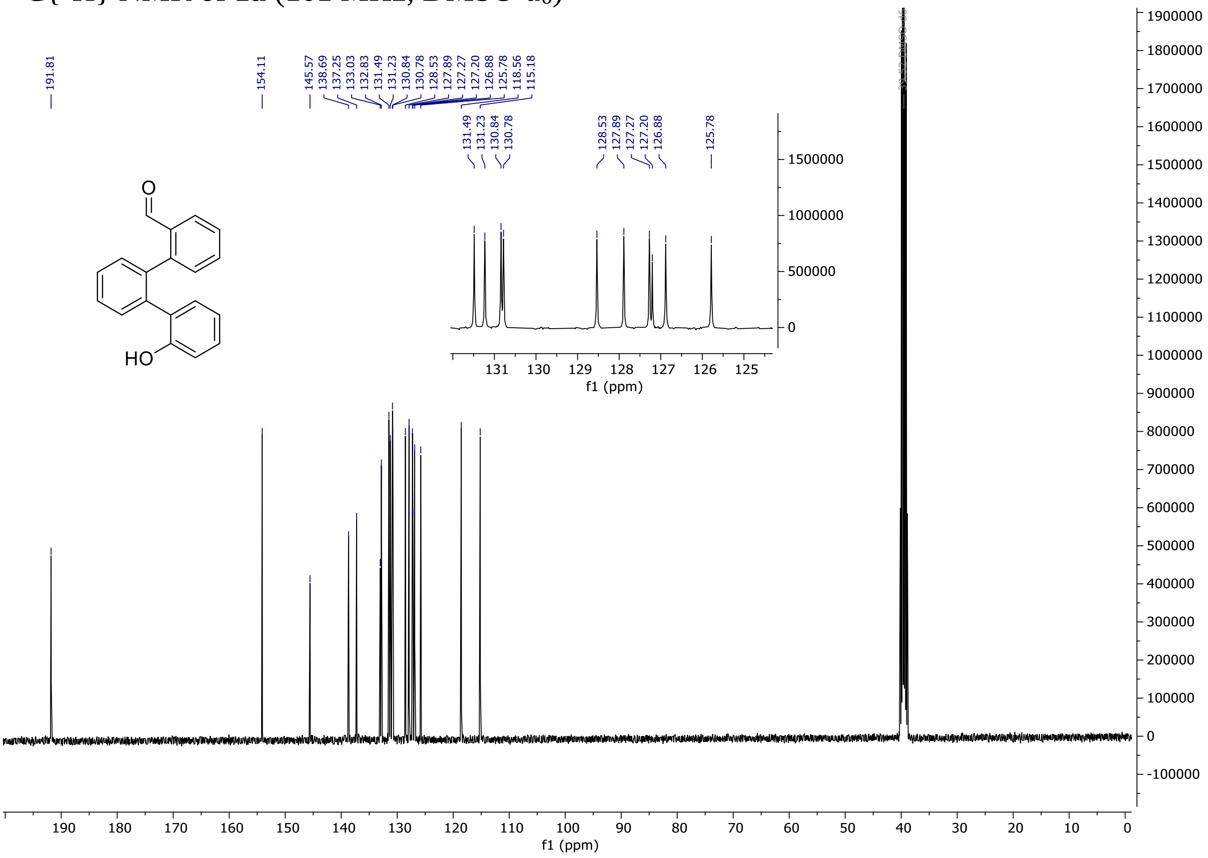

COSY of **1a** (DMSO-d_6)

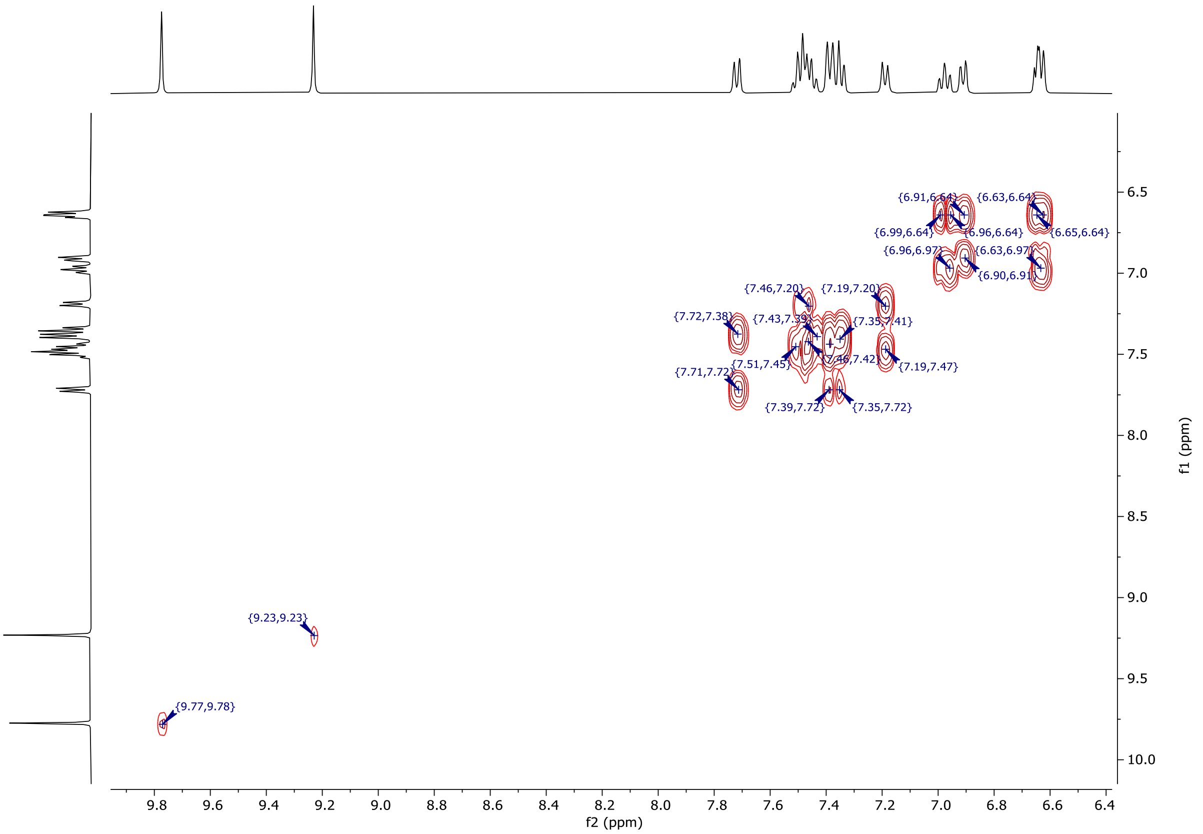

HSQC of **1a** (DMSO-d_6)

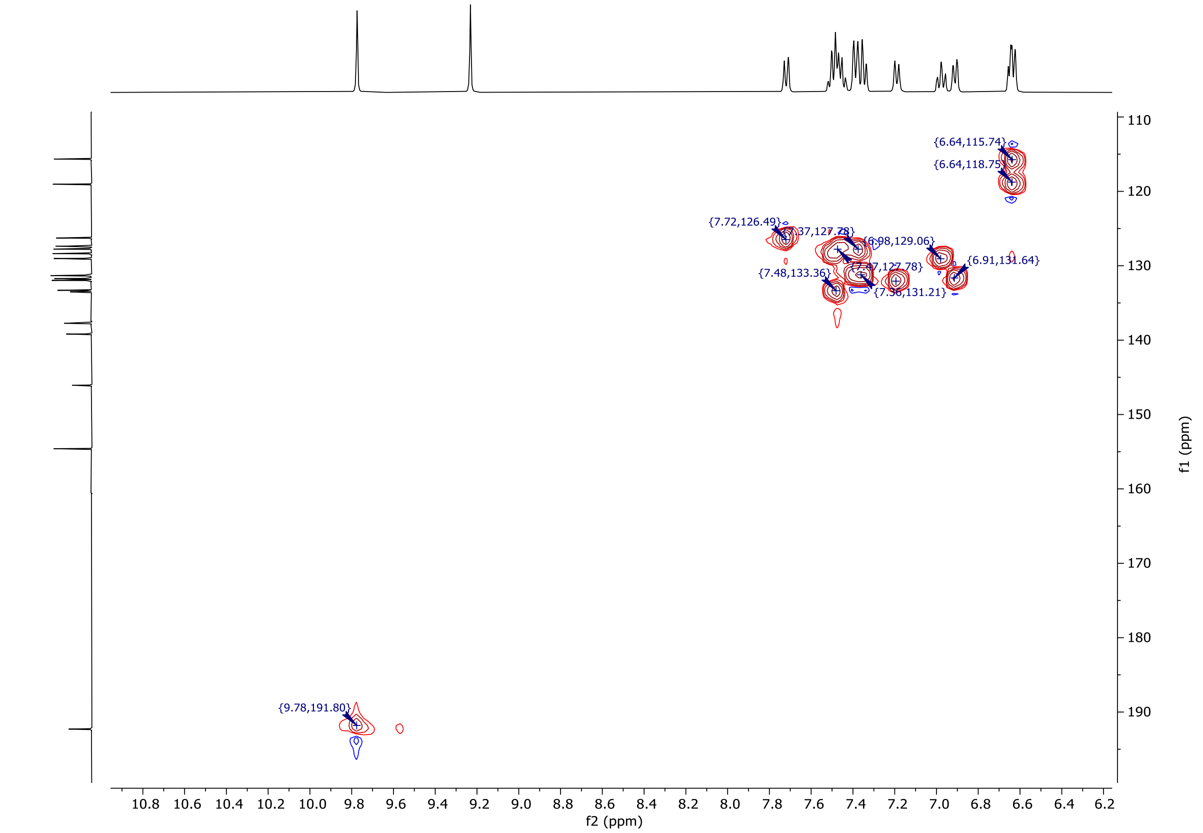

HMBC of **1a** (DMSO-d_6)

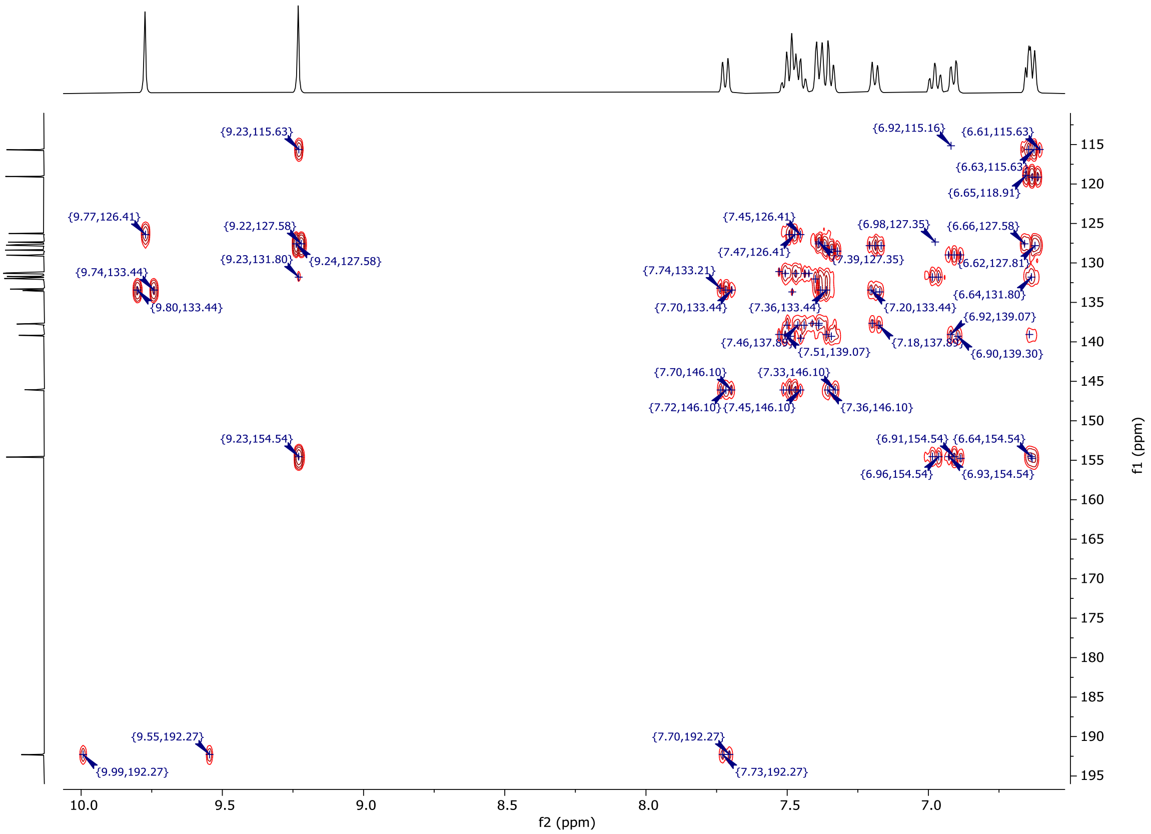

^{1}H NMR of **1a** (400 MHz, DCM-d_2)

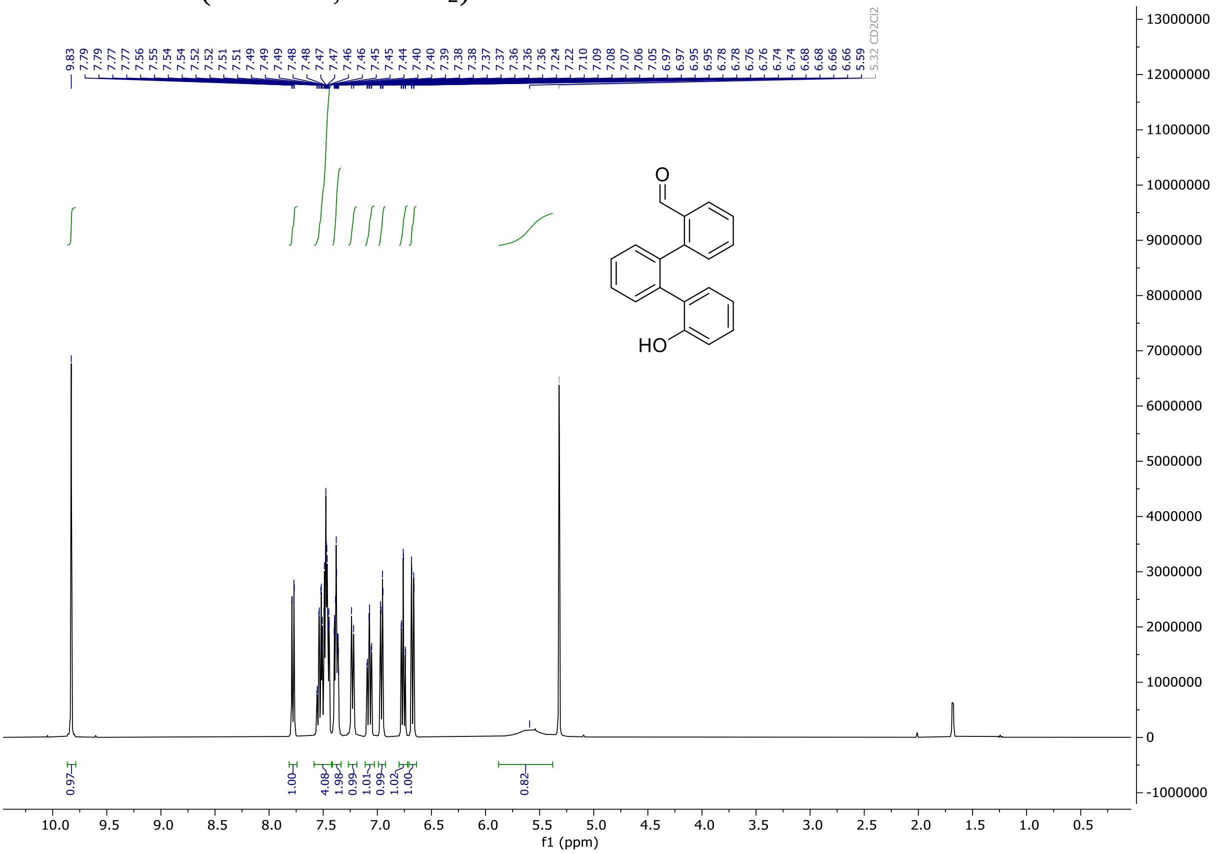

$^{13}C\{^{1}H\}$ NMR of **1a** (101 MHz, DCM-d_2)

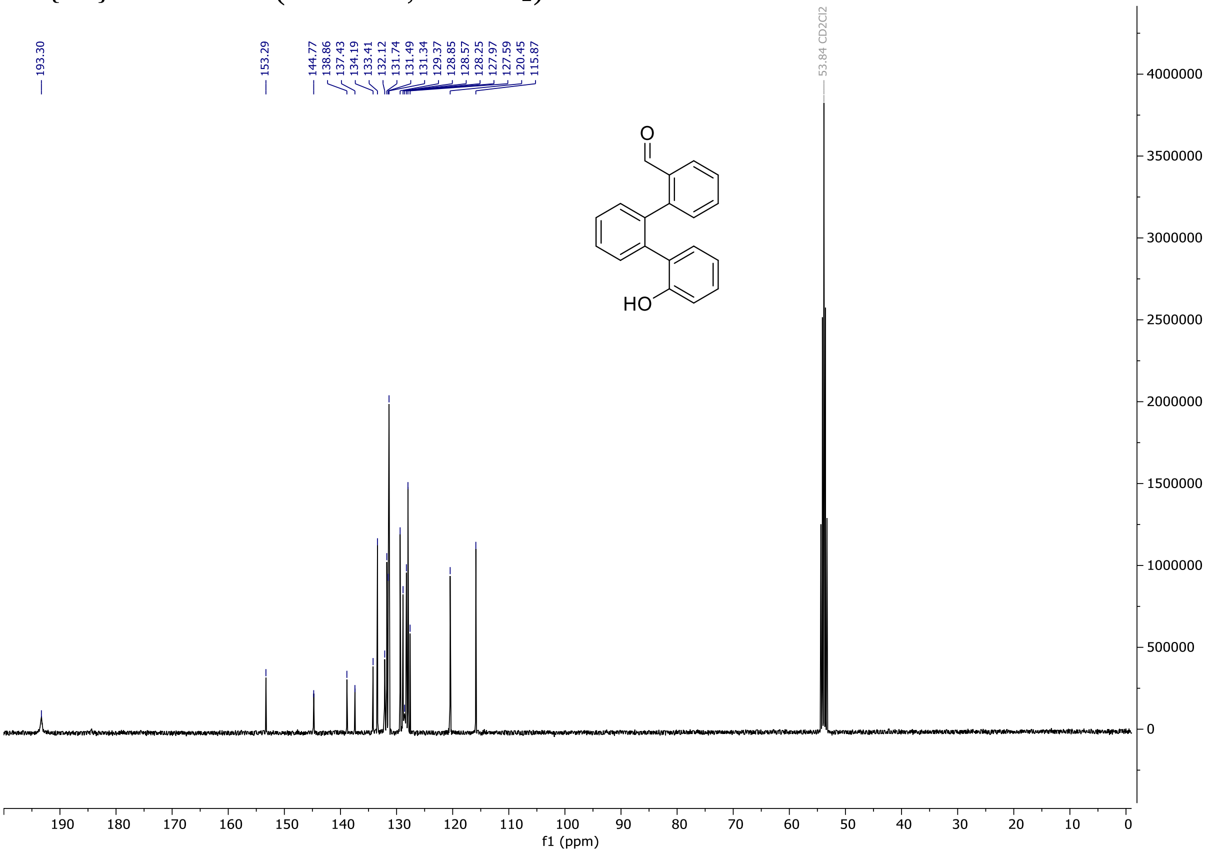

COSY of **1a** (DCM-d_2)

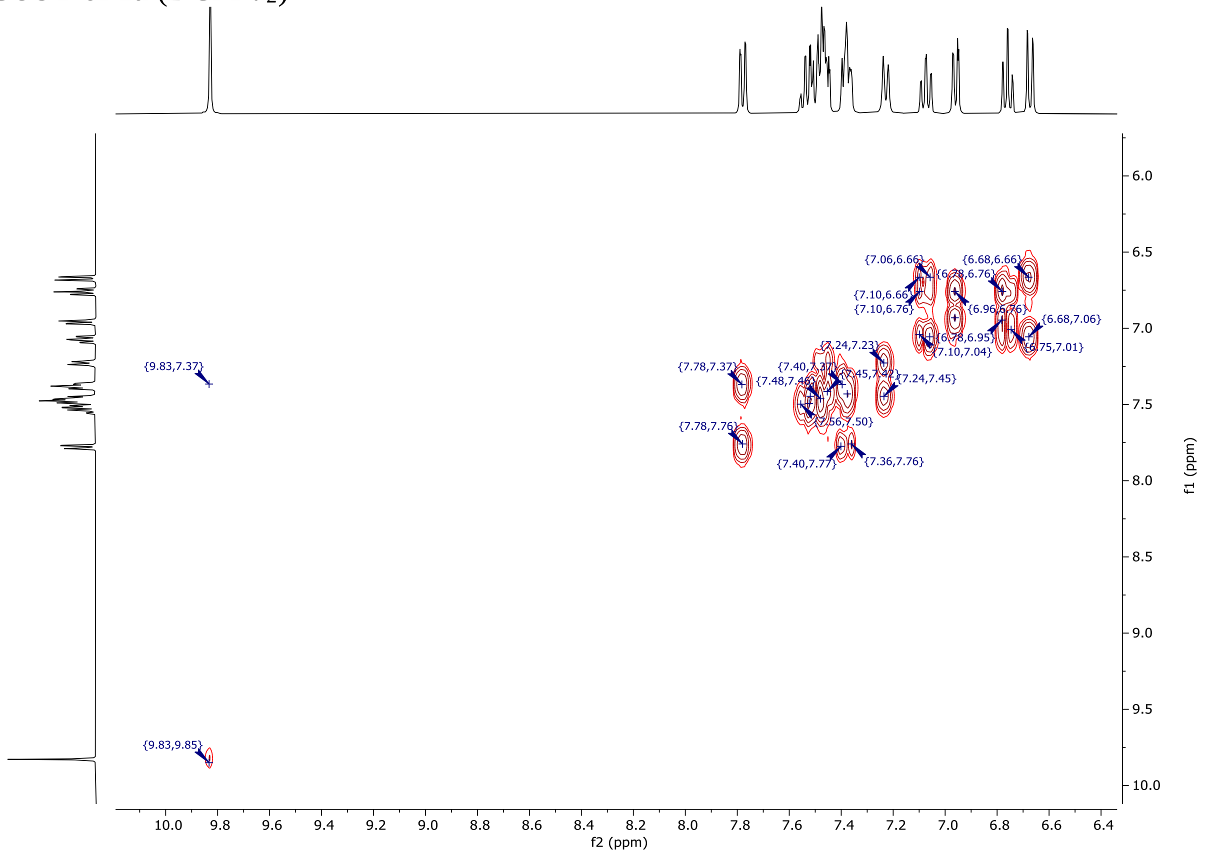

HSQC of **1a** (DCM-*d*$_2$)

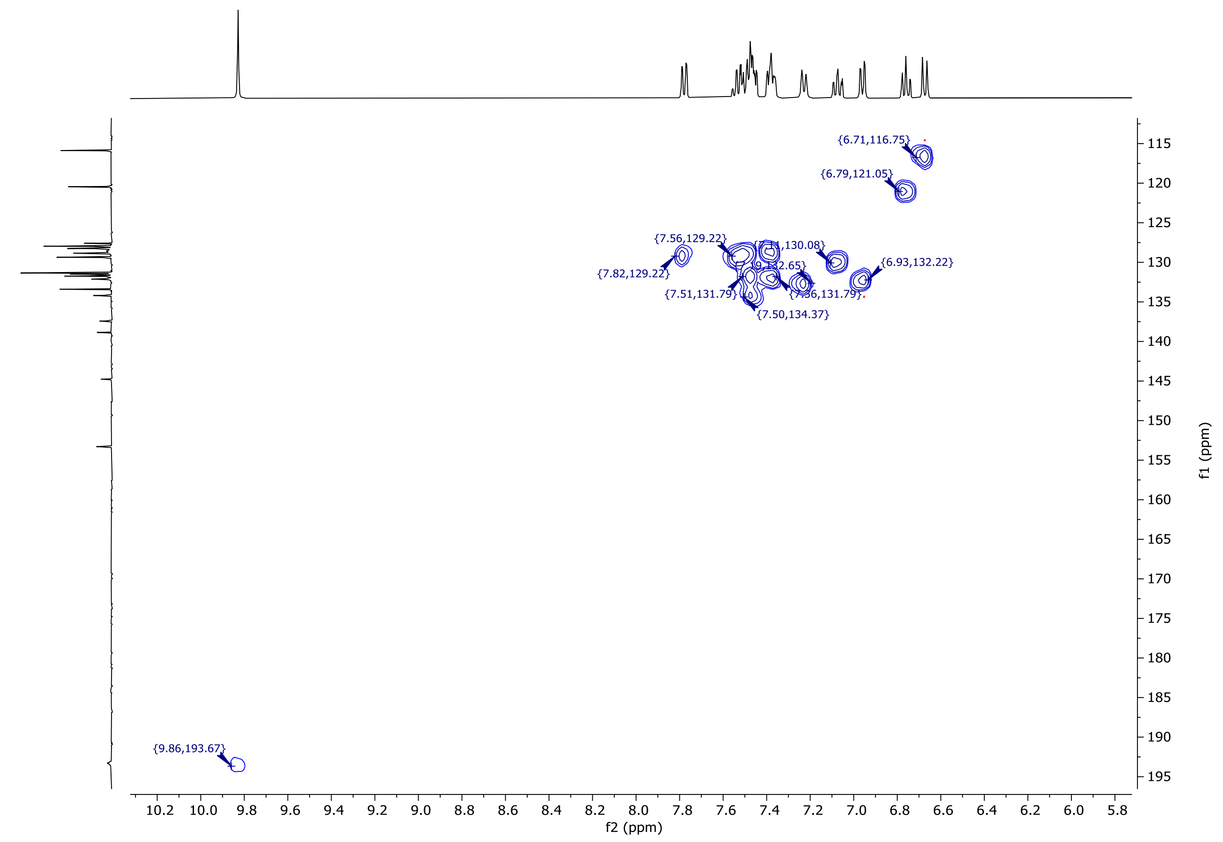

HMBC of **1a** (DCM-*d*$_2$)

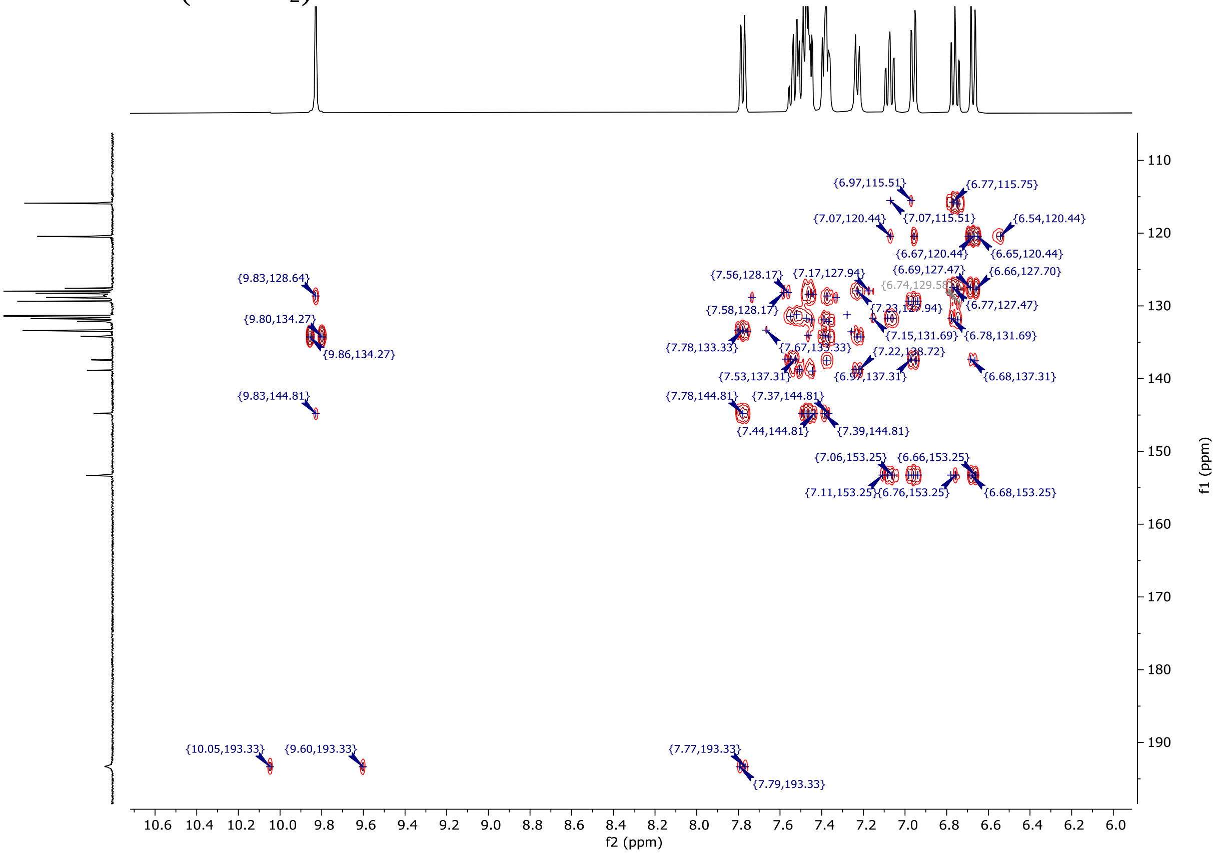

2''-Hydroxy-5-methyl-[1,1':2',1''-terphenyl]-2-carbaldehyde (1b)

^{1}H NMR of **1b** (400 MHz, DMSO-d_6)

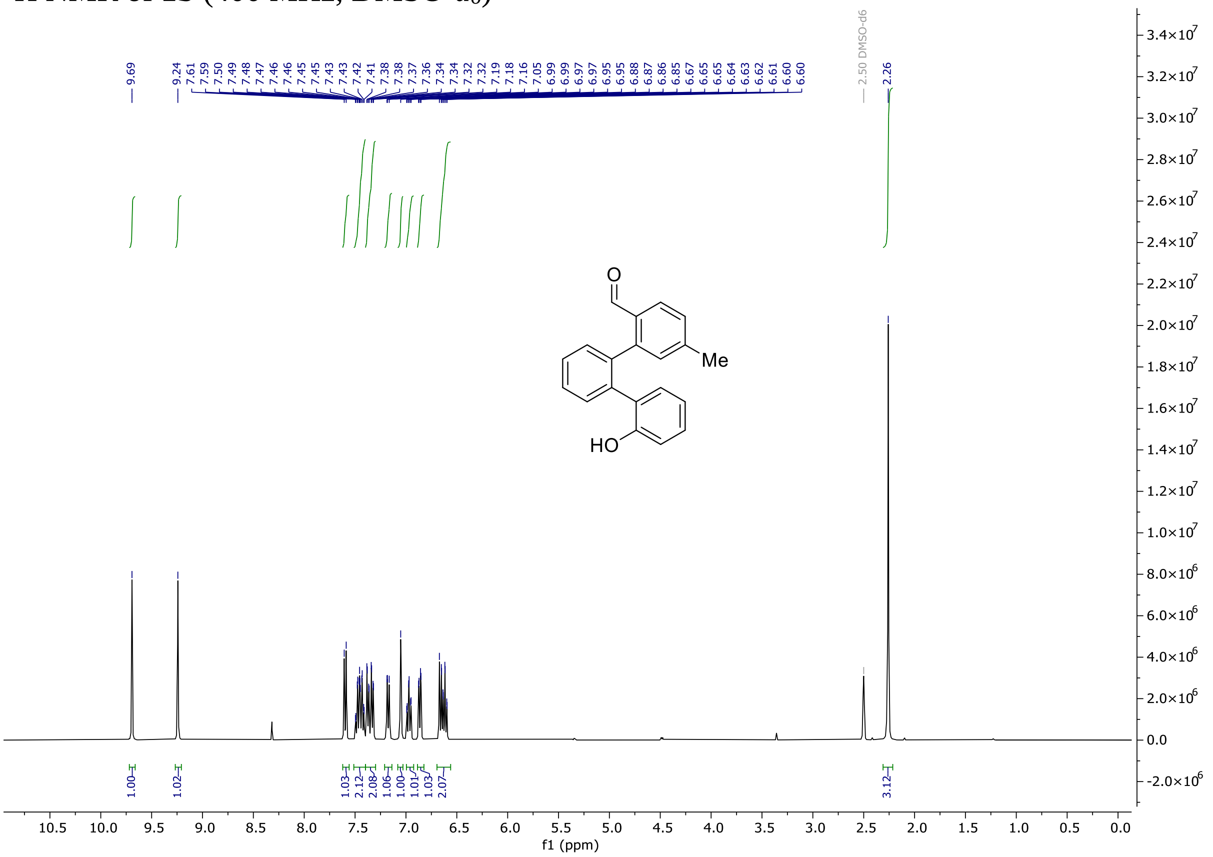

^{13}C{^{1}H} NMR of **1b** (101 MHz, DMSO-d_6)

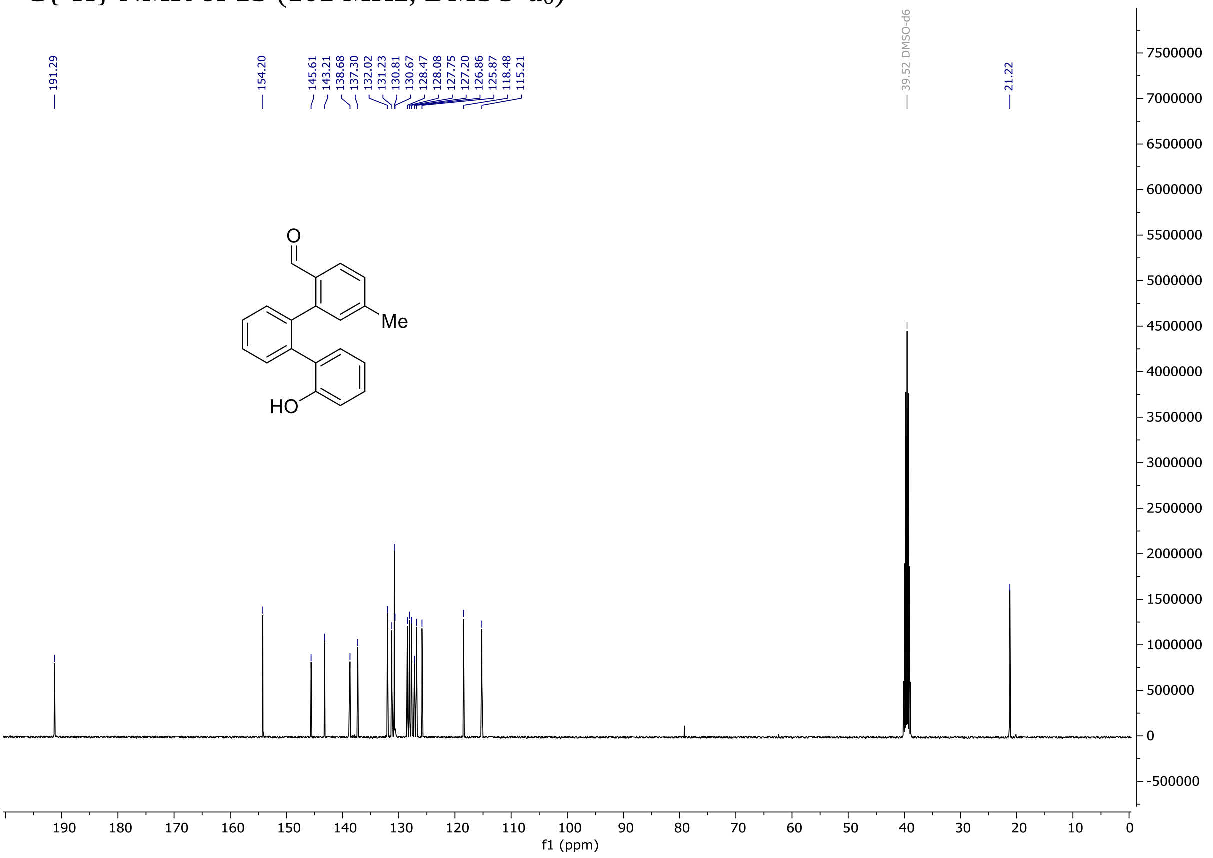

2''-Hydroxy-3-methyl-[1,1':2',1''-terphenyl]-2-carbaldehyde (1c)

^{1}H NMR of **1c** (400 MHz, DCM-d_2)

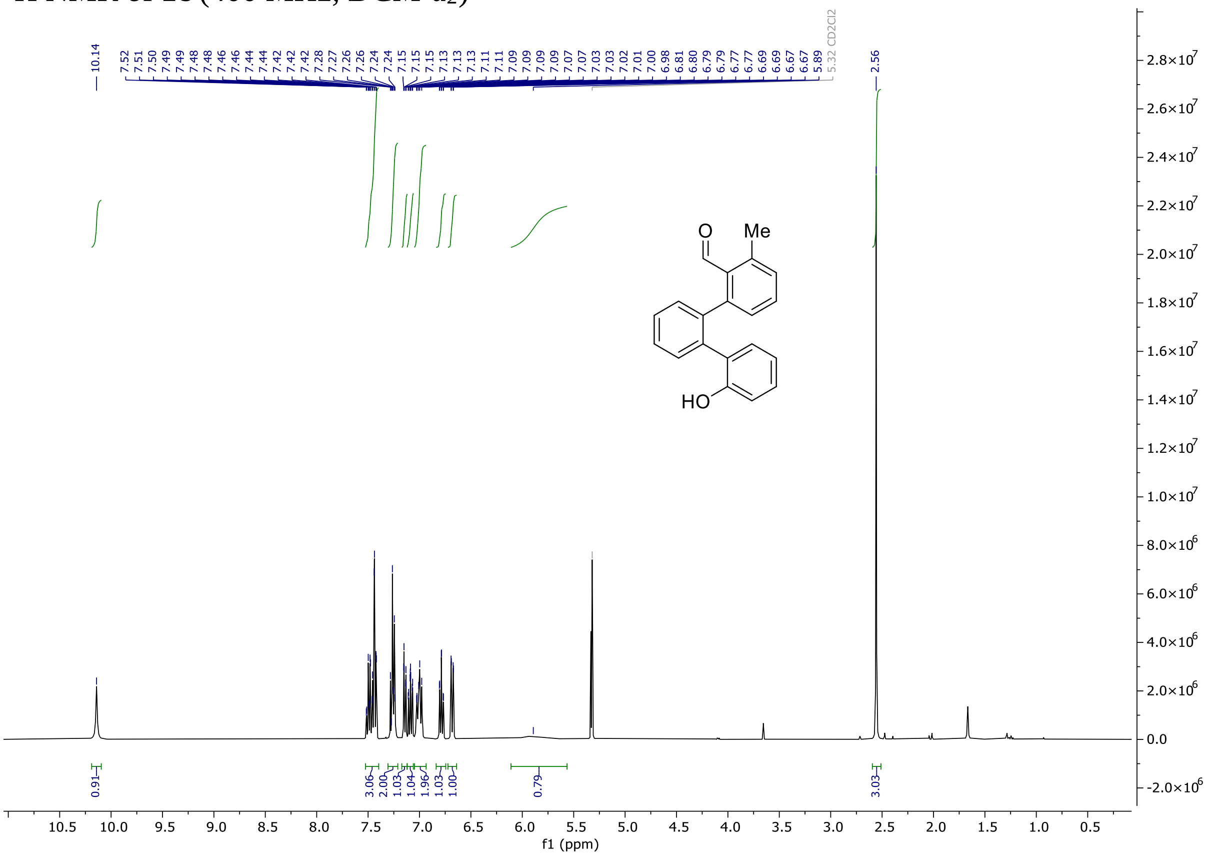

^{13}C{^{1}H} NMR of **1c** (101 MHz, DCM-d_2)

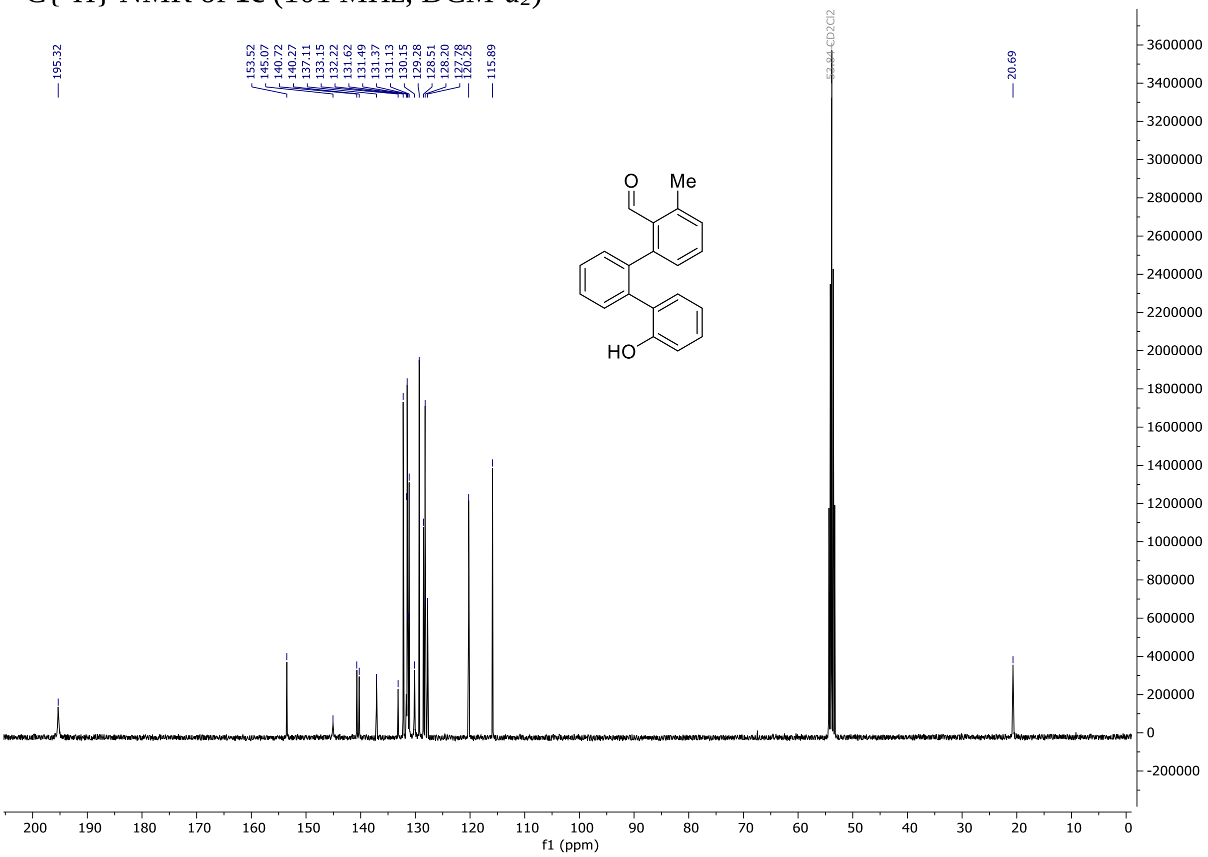

2''-Hydroxy-4-methyl-[1,1':2',1''-terphenyl]-2-carbaldehyde (1d)

^{1}H NMR of **1d** (400 MHz, DCM-d_2)

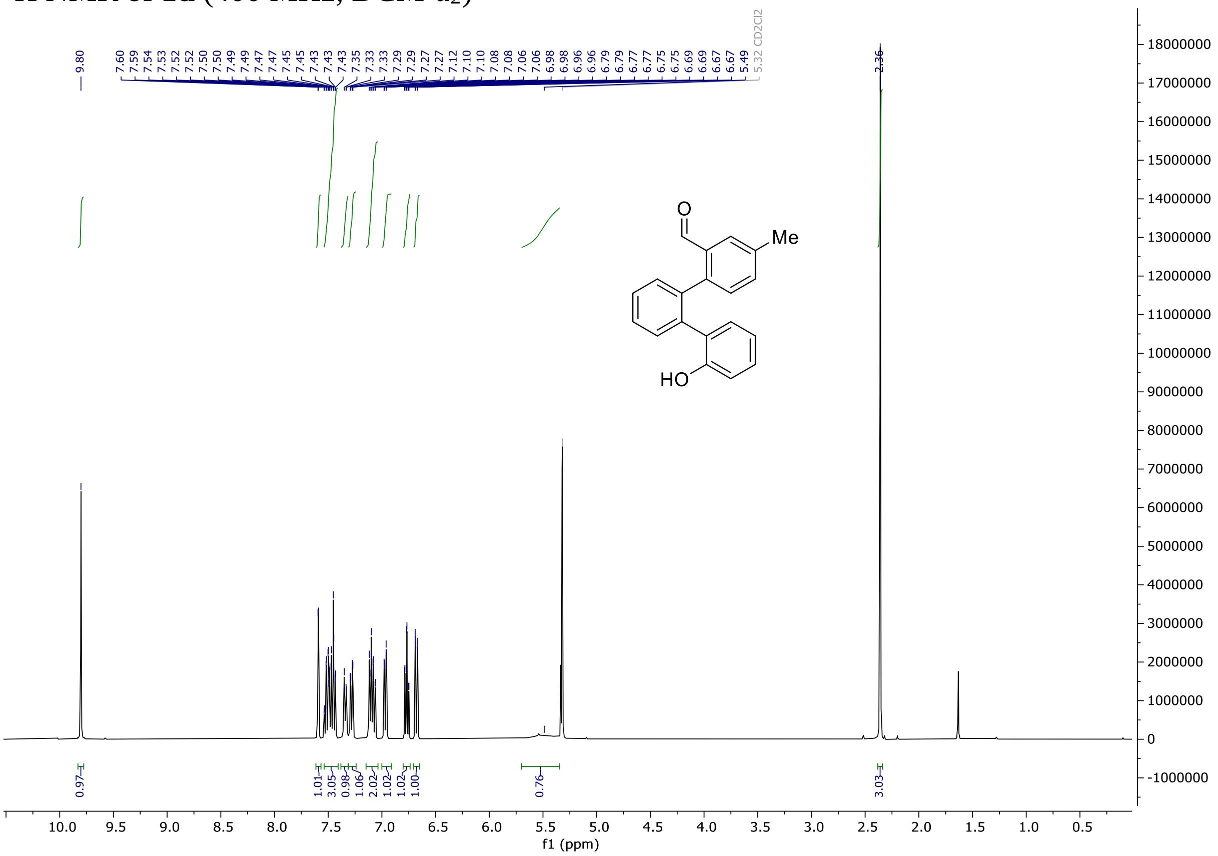

^{13}C{^{1}H} NMR of **1d** (101 MHz, DCM-d_2)

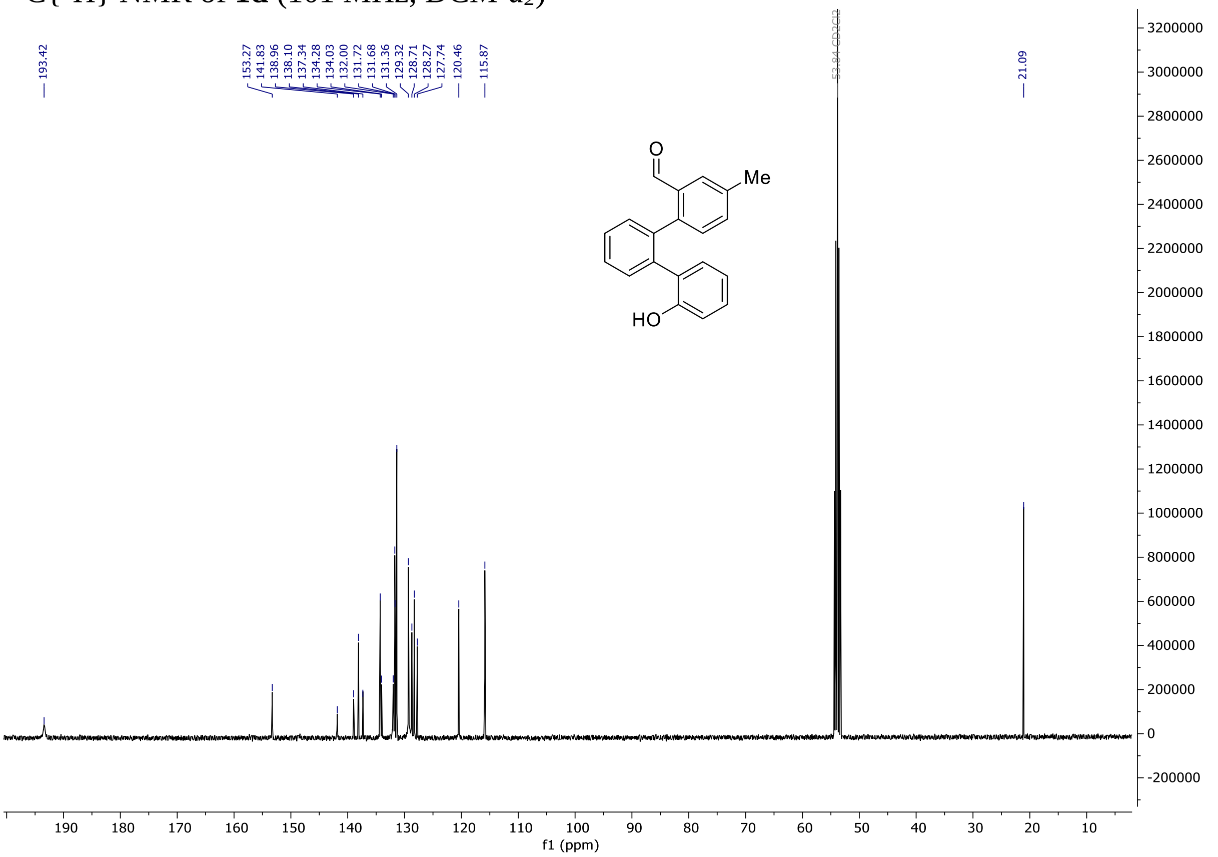

2''-Hydroxy-6-methyl-[1,1':2',1''-terphenyl]-2-carbaldehyde (1e)

^{1}H NMR of **1e** (400 MHz, DCM-d_2)

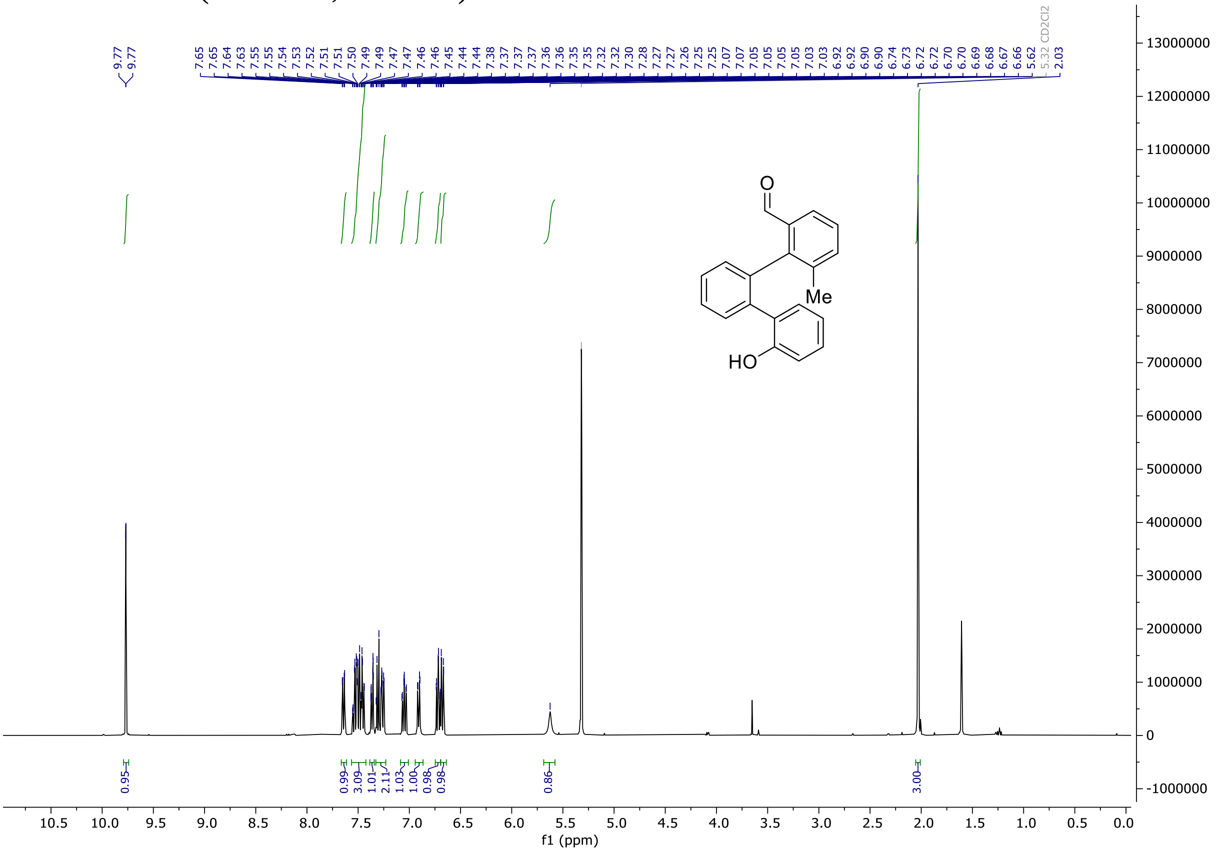

^{13}C{^{1}H} NMR of **1e** (101 MHz, DCM-d_2)

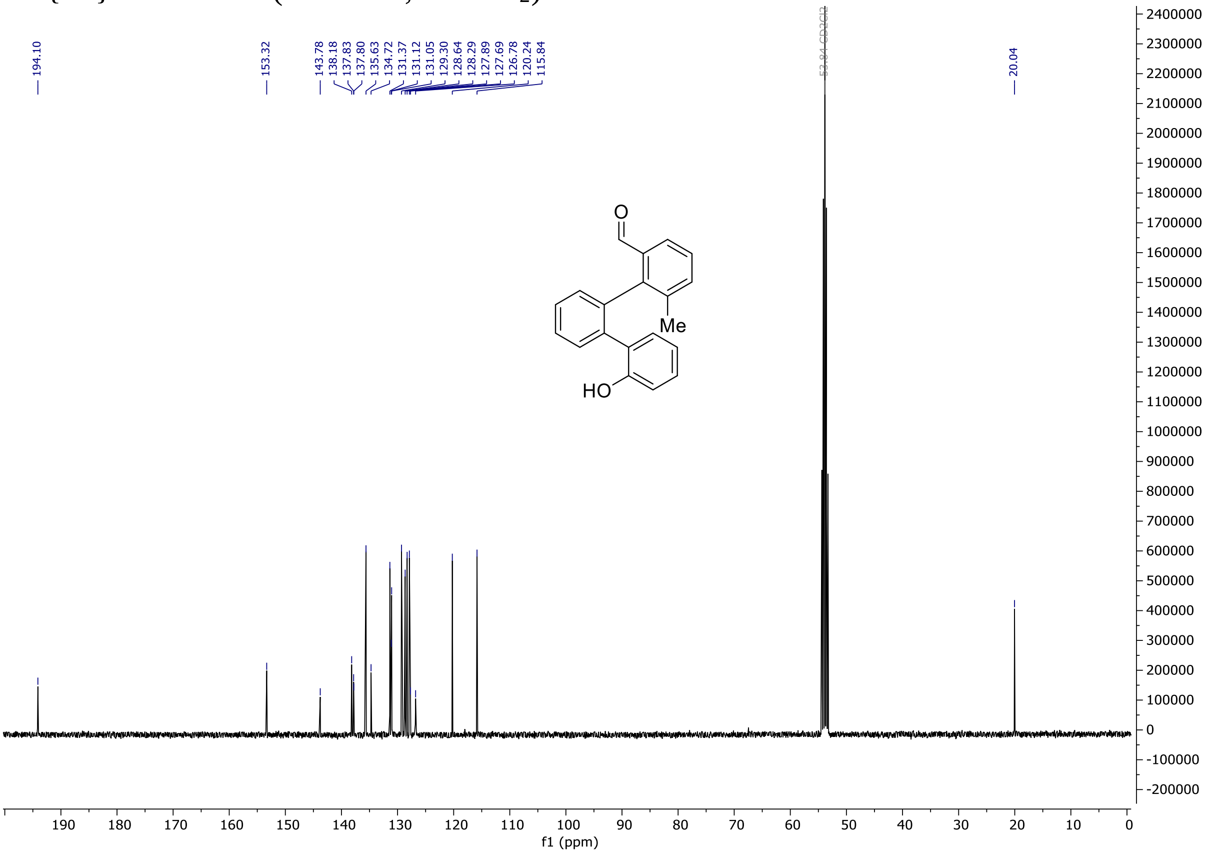

2''-Hydroxy-5-methoxy-[1,1':2',1''-terphenyl]-2-carbaldehyde (1f)

^{1}H NMR of **1f** (400 MHz, DMSO-d_6)

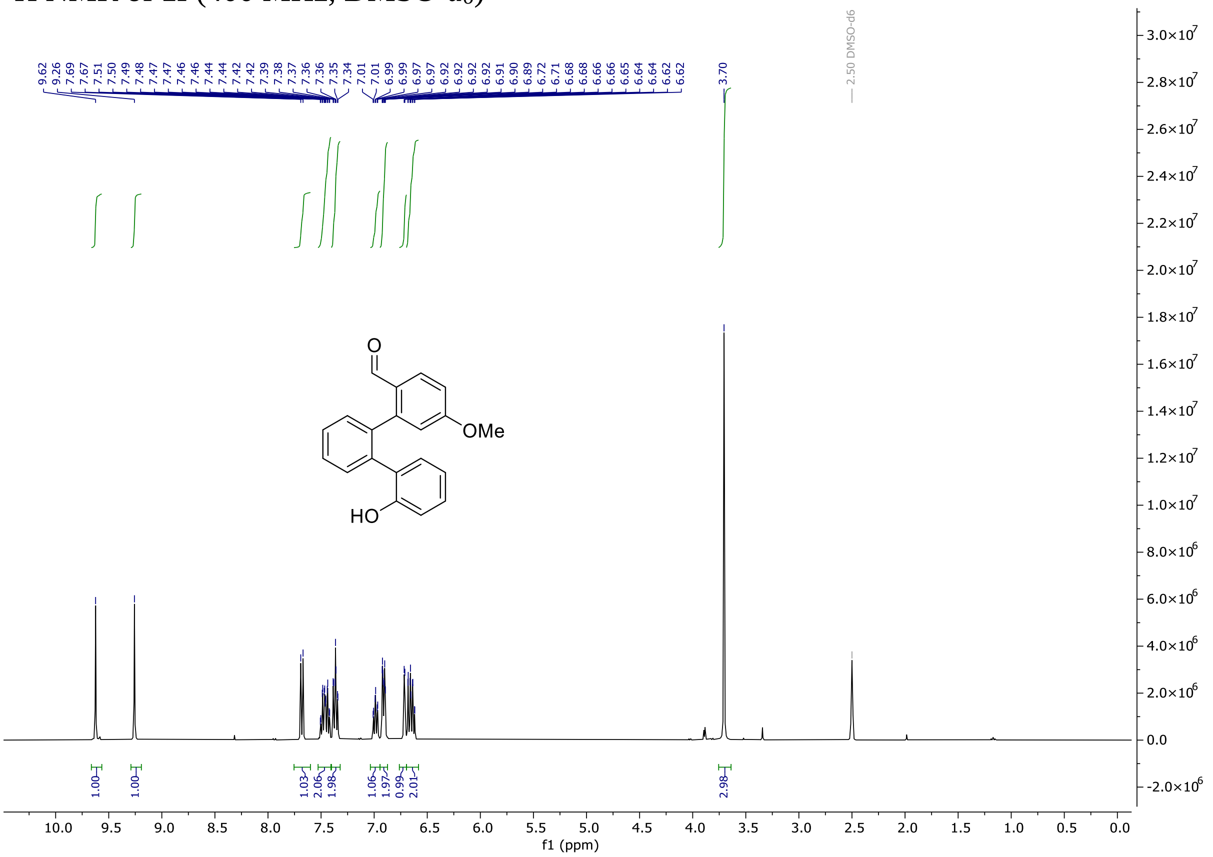

^{13}C{^{1}H} NMR of **1f** (101 MHz, DMSO-d_6)

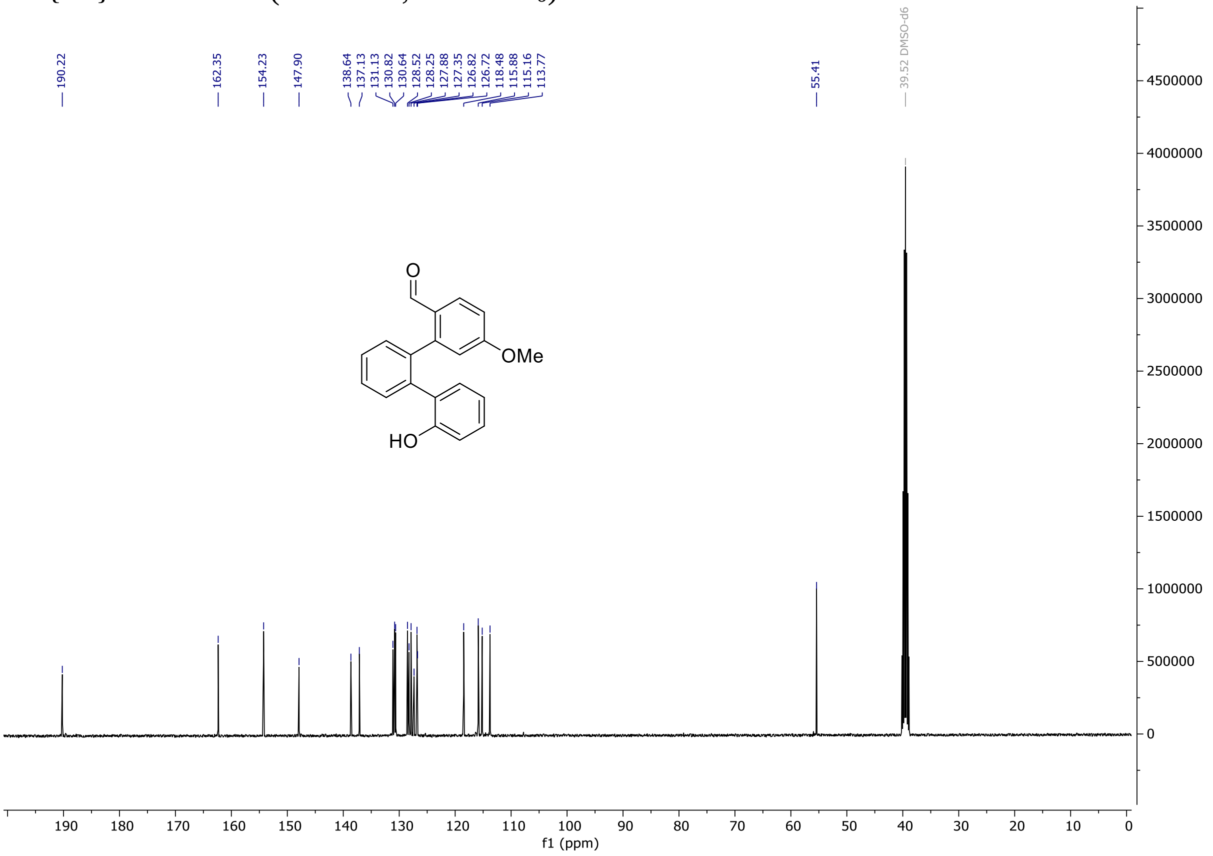

5-Fluoro-2''-hydroxy-[1,1':2',1''-terphenyl]-2-carbaldehyde (1g)

^{1}H NMR of **1g** (400 MHz, DMSO-d_6)

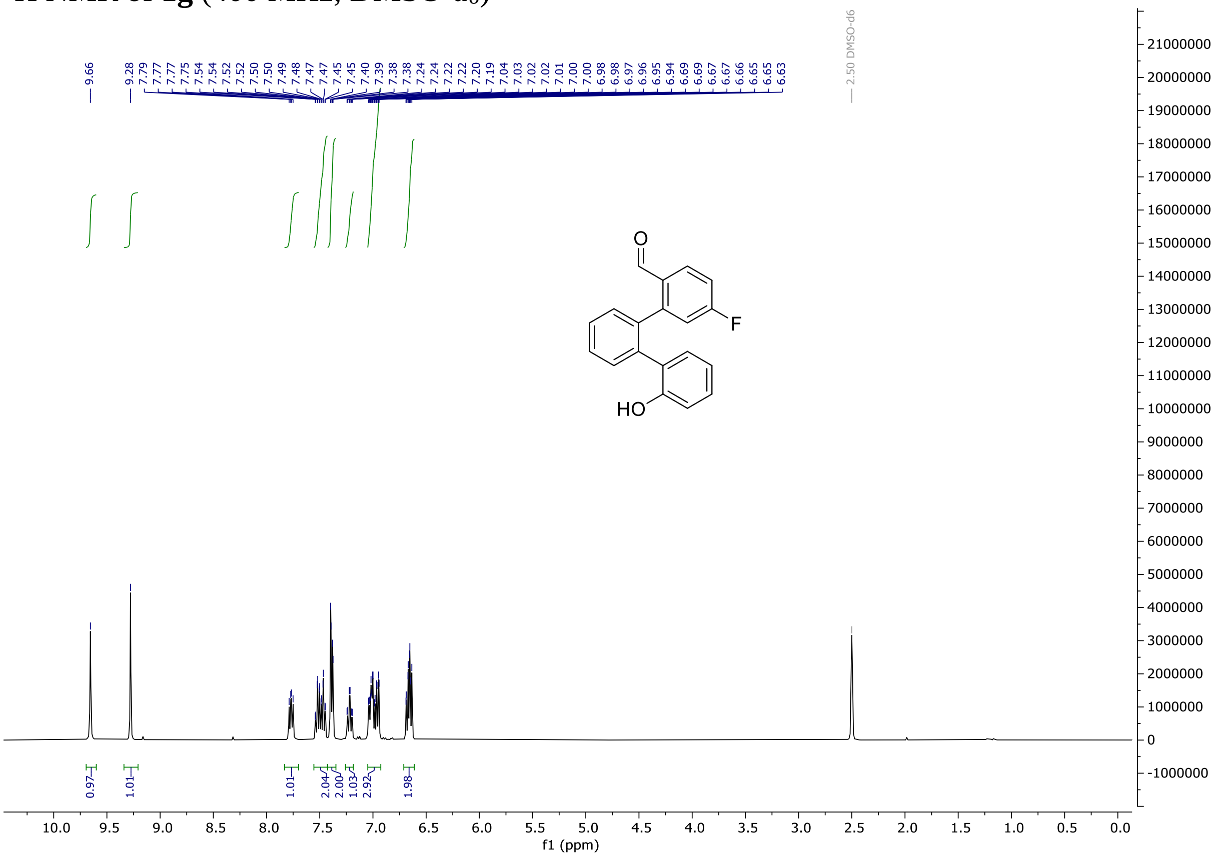

^{13}C{^{1}H} NMR of **1g** (101 MHz, DMSO-d_6)

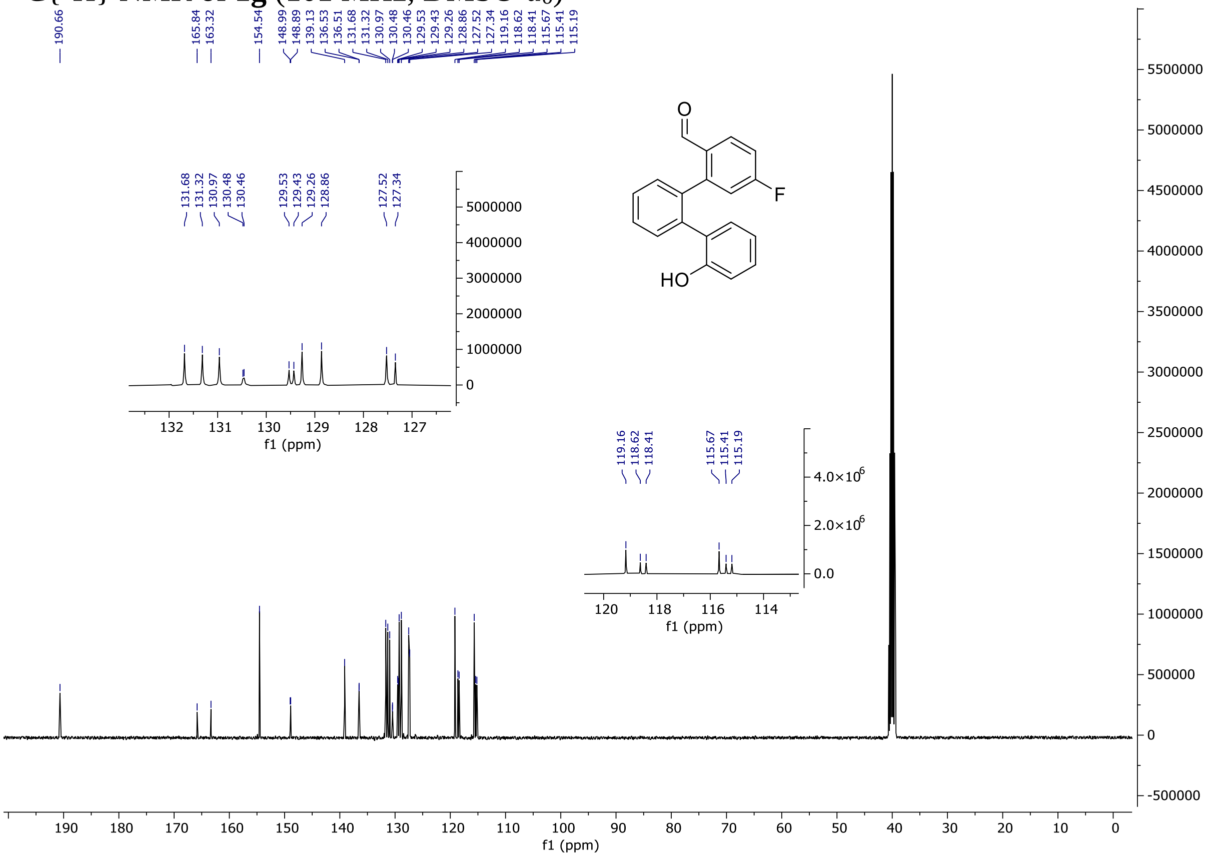

^{19}F NMR of **1g** (376 MHz, DMSO-d_6)

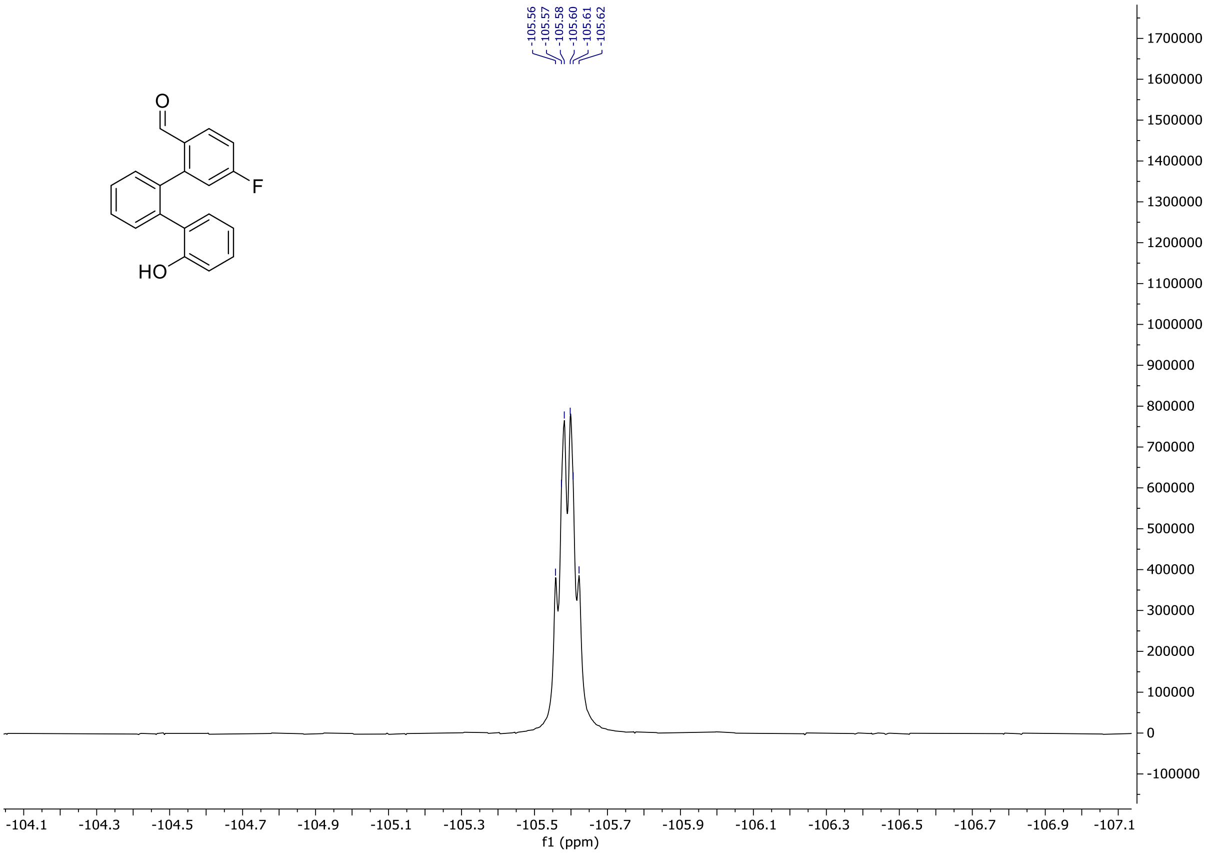

5-Chloro-2''-hydroxy-[1,1':2',1''-terphenyl]-2-carbaldehyde (1h)

^{1}H NMR of **1h** (400 MHz, DMSO-d_6)

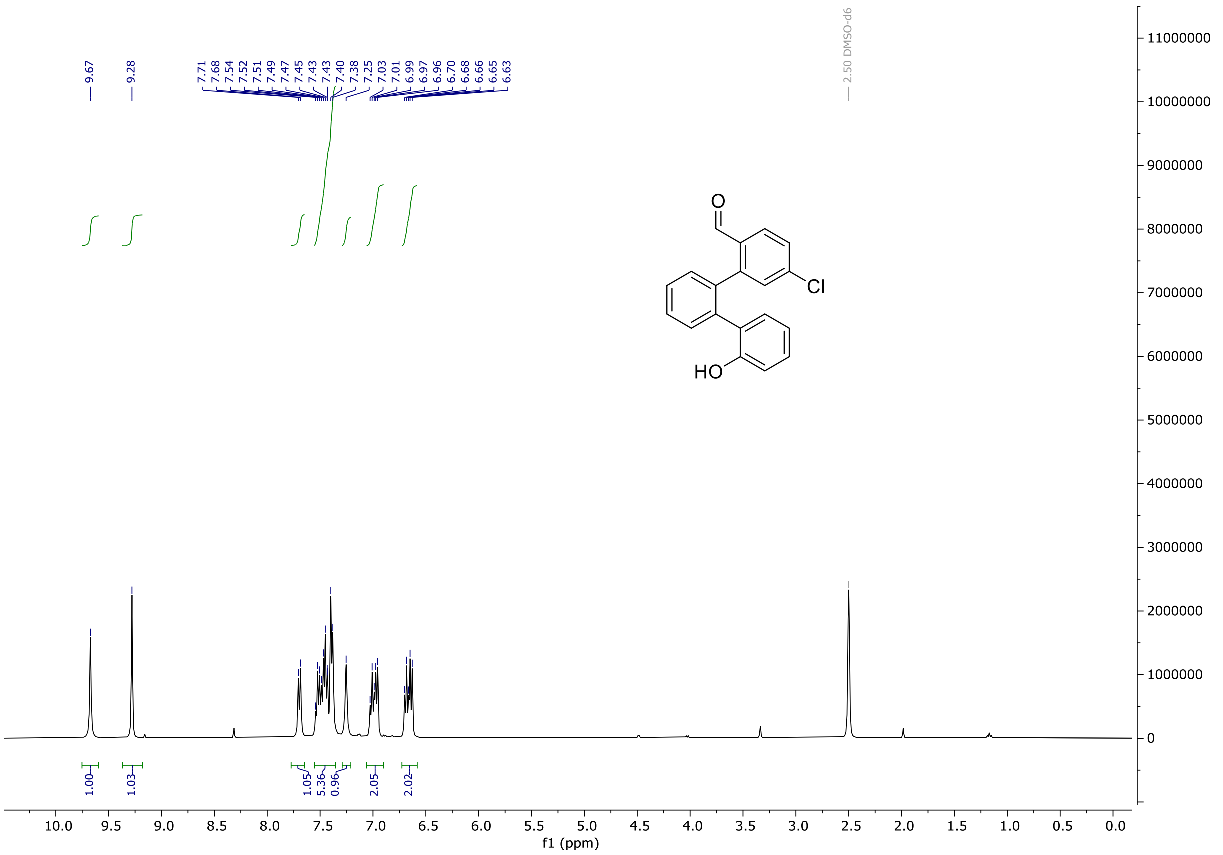

^{13}C{^{1}H} NMR of **1h** (101 MHz, DMSO-d_6)

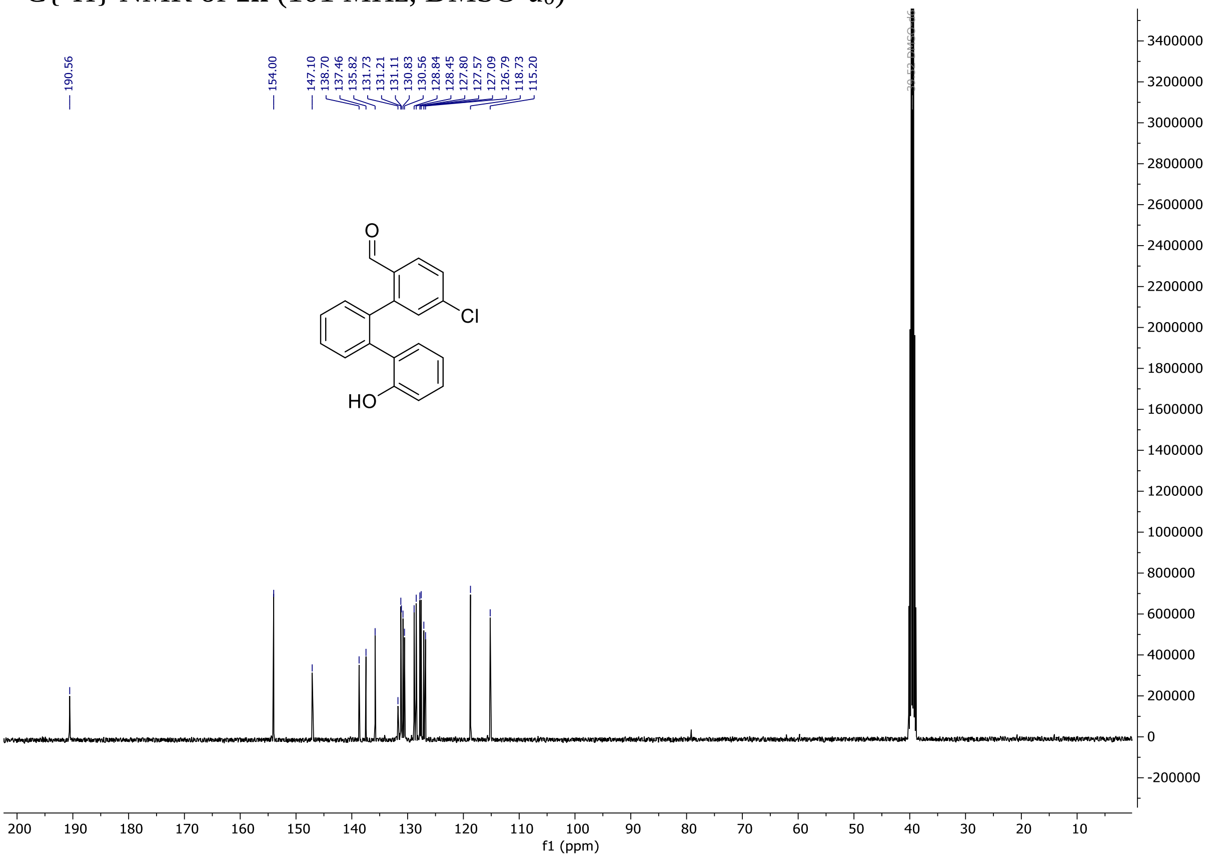

5-Bromo-2''-hydroxy-[1,1':2',1''-terphenyl]-2-carbaldehyde (1i)

^{1}H NMR of **1i** (400 MHz, DMSO-d_6)

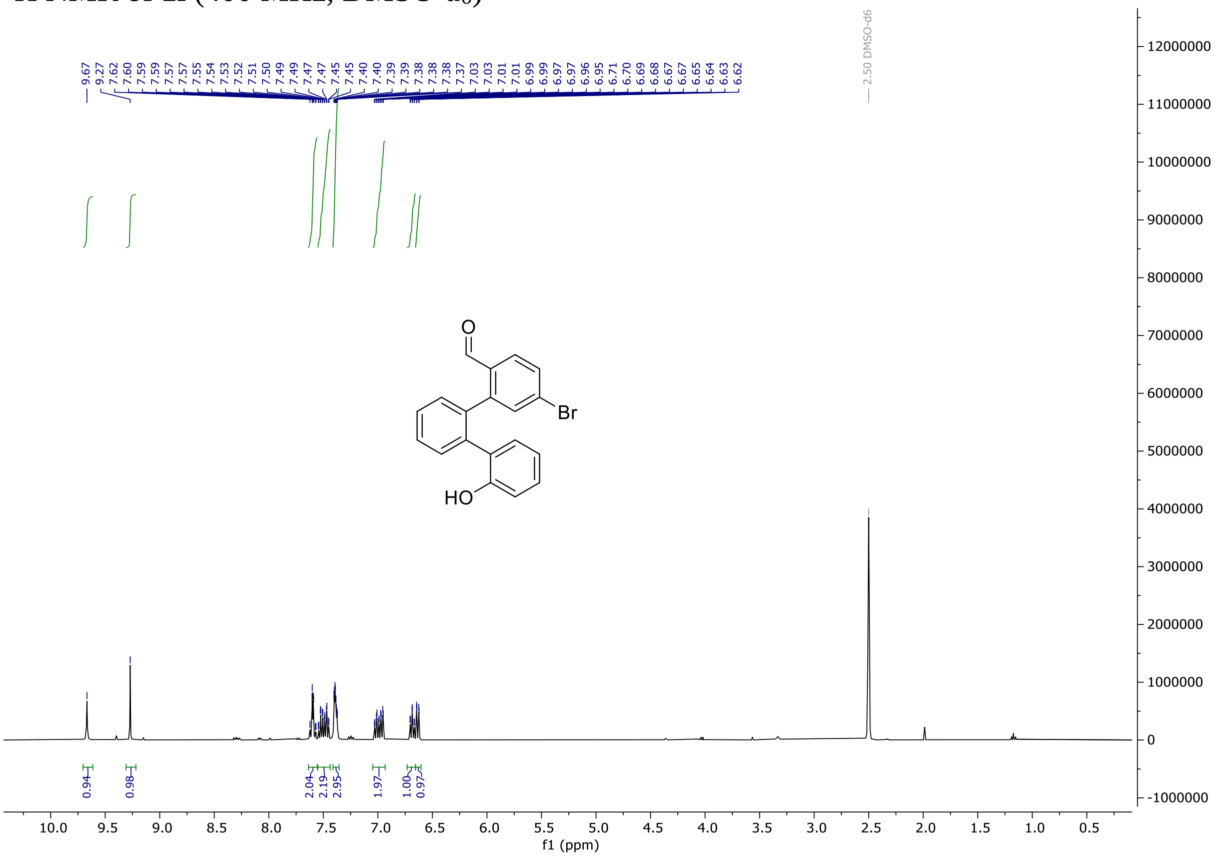

^{13}C{^{1}H} NMR of **1i** (101 MHz, DMSO-d_6)

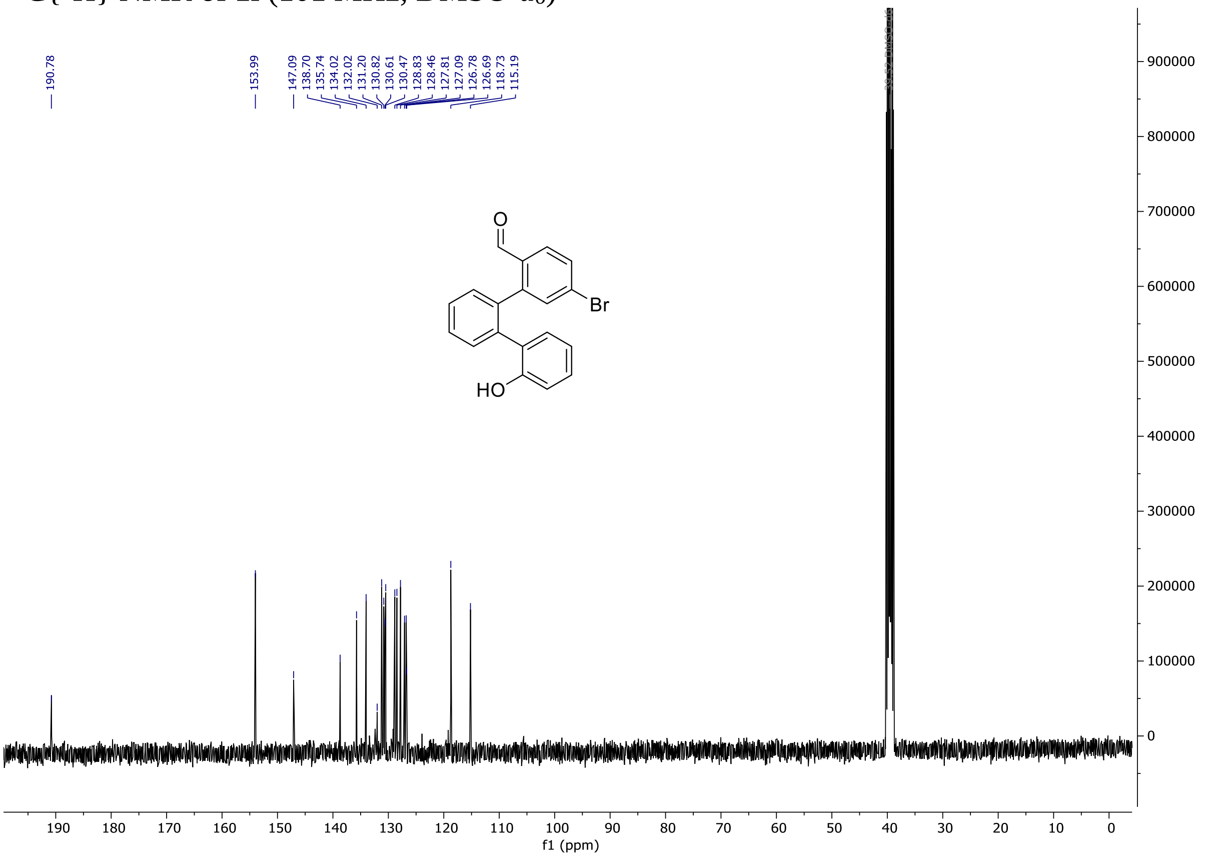

6-Formyl-2''-hydroxy-[1,1':2',1''-terphenyl]-3-carbonitrile (1j)

^{1}H NMR of **1j** (400 MHz, DMSO-*d*$_6$)

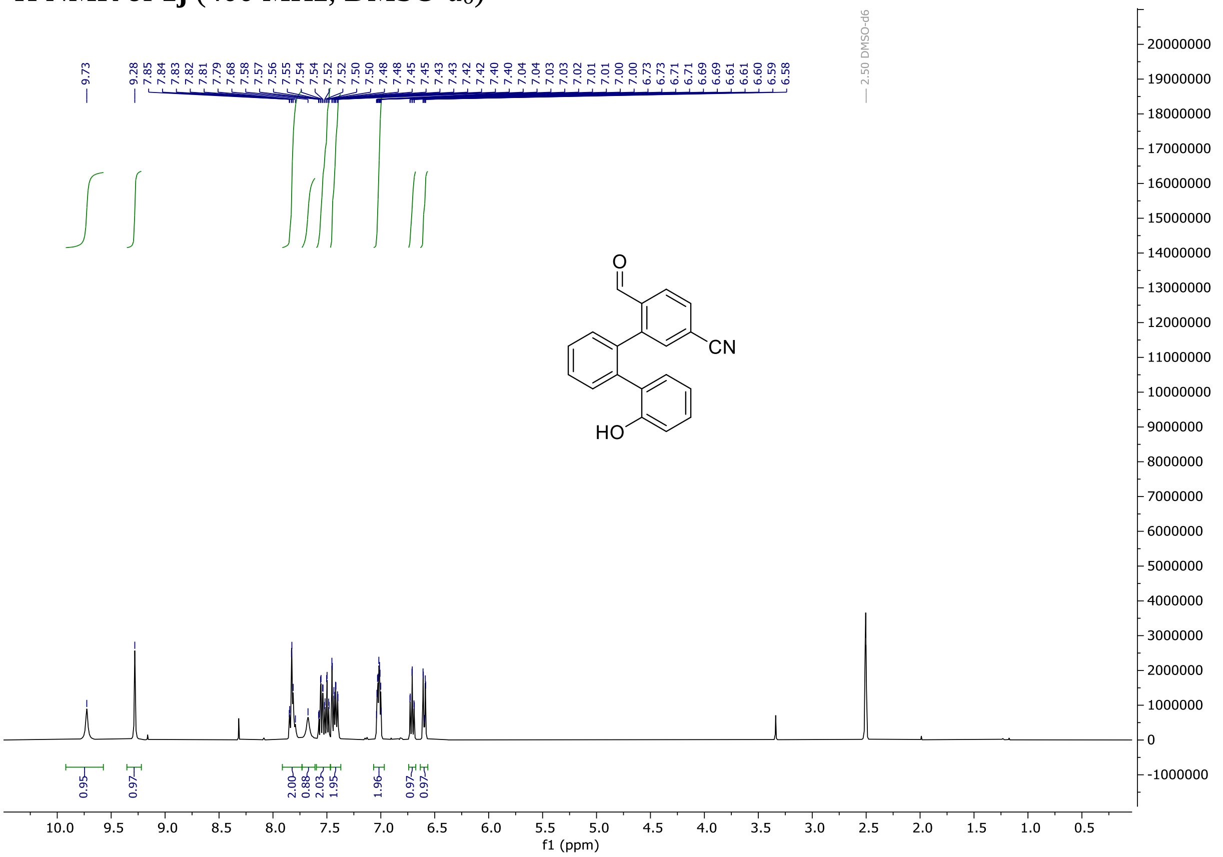

^{13}C{^{1}H} NMR of **1j** (101 MHz, DMSO-*d*$_6$)

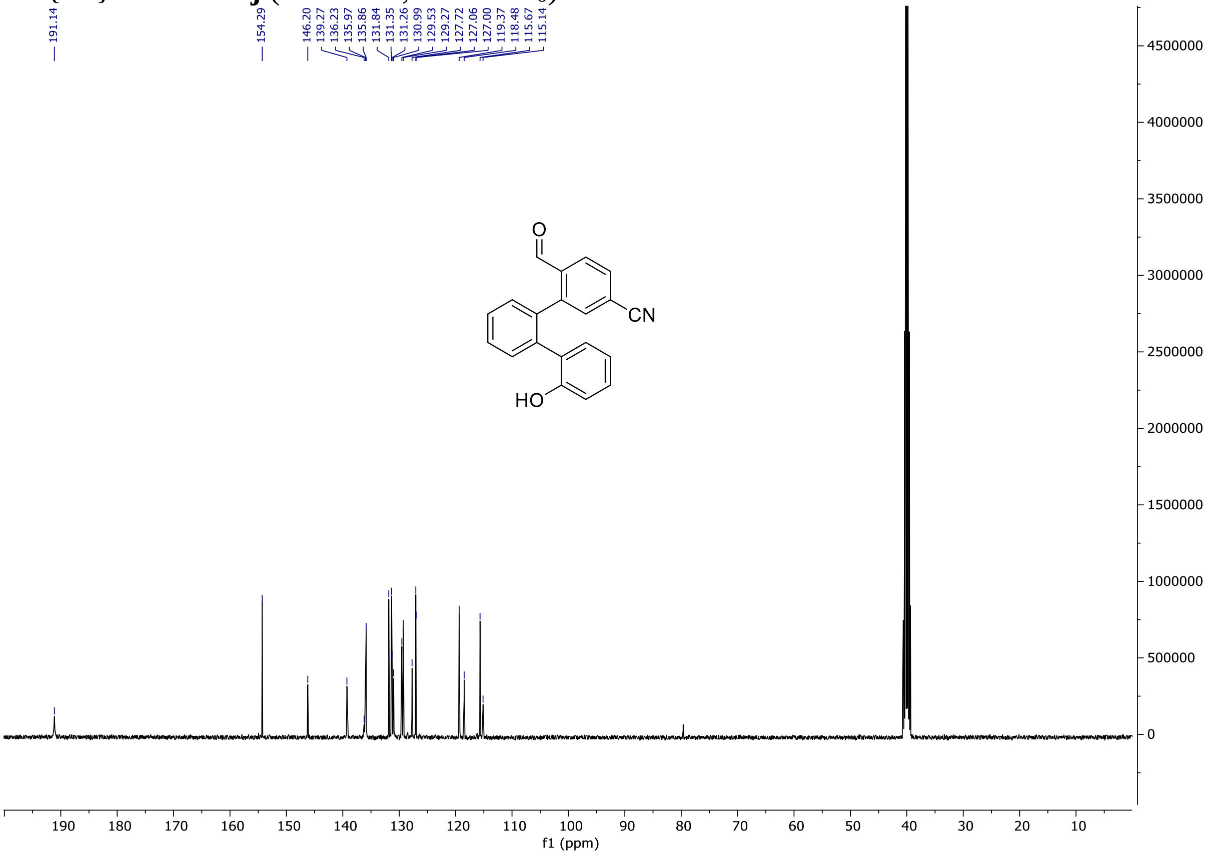

2''-Hydroxy-5-(trifluoromethyl)-[1,1':2',1''-terphenyl]-2-carbaldehyde (1k)

^{1}H NMR of **1k** (400 MHz, DMSO-d_6)

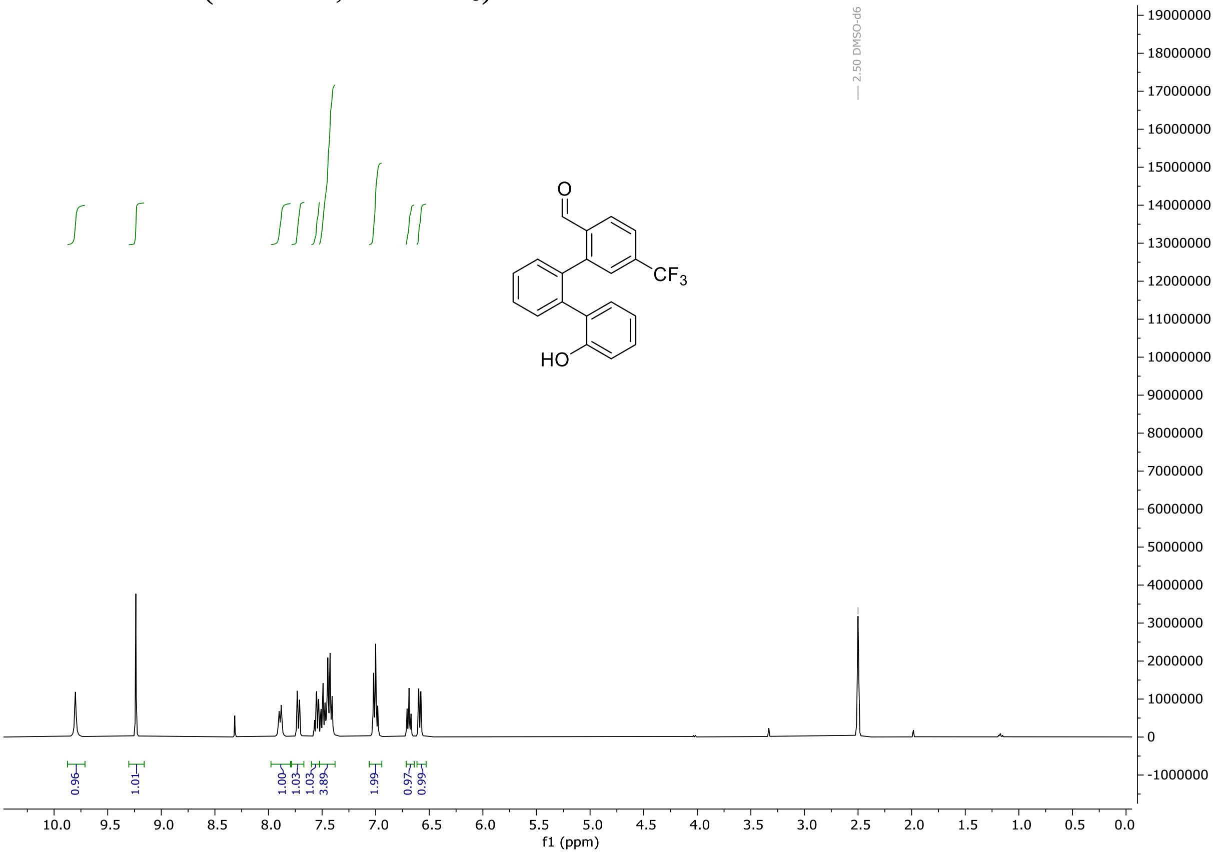

^{13}C{^{1}H} NMR of **1k** (101 MHz, DMSO-d_6)

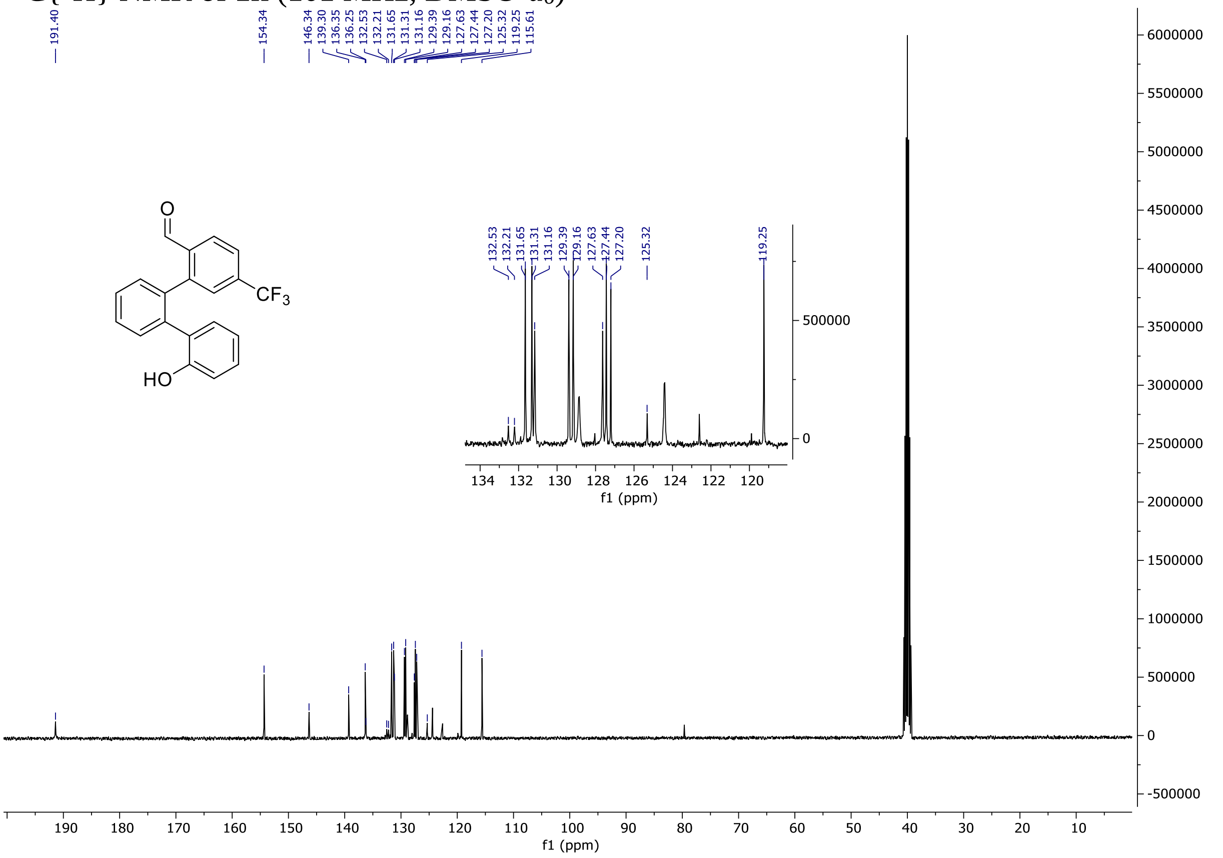

^{19}F NMR of **1k** (376 MHz, DMSO-d_6)

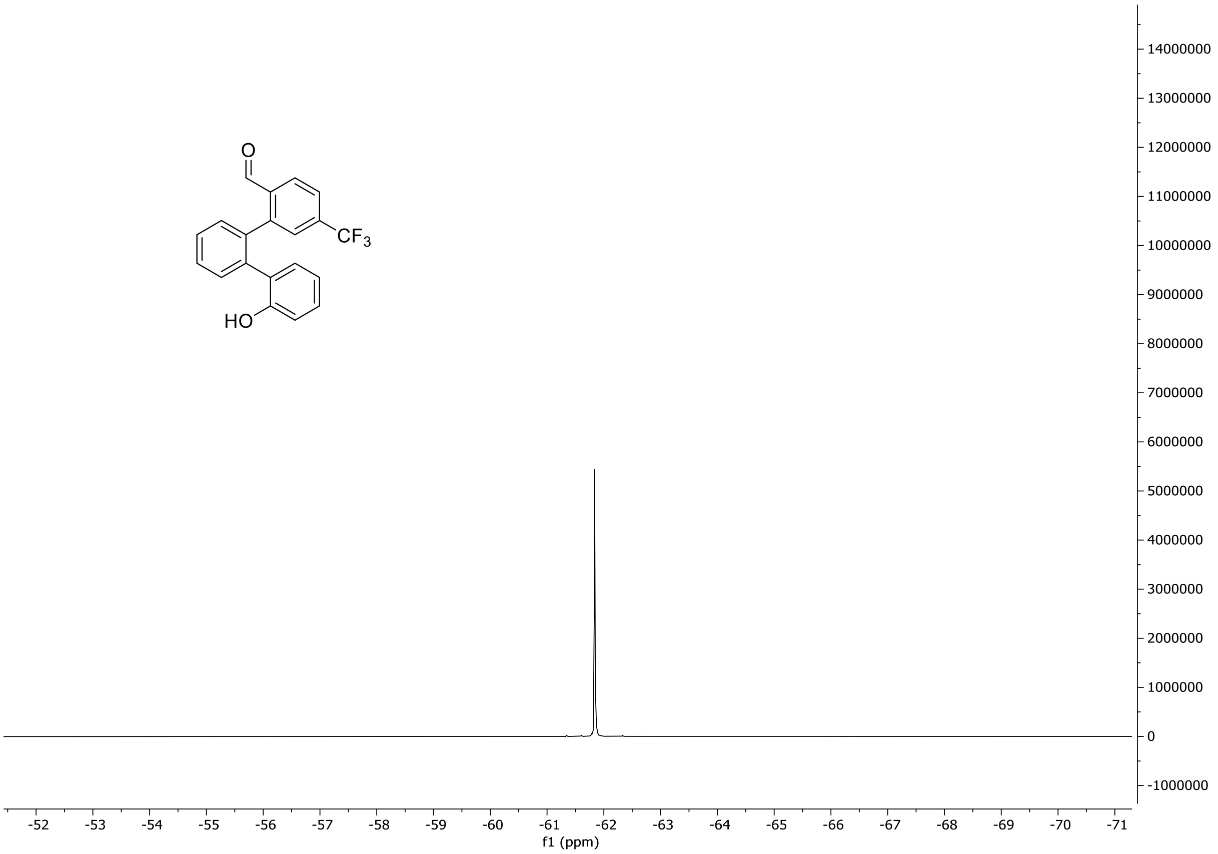

2''-Hydroxy-5-nitro-[1,1':2',1''-terphenyl]-2-carbaldehyde (1l)

^{1}H NMR of **1l** (400 MHz, DMSO-d_6)

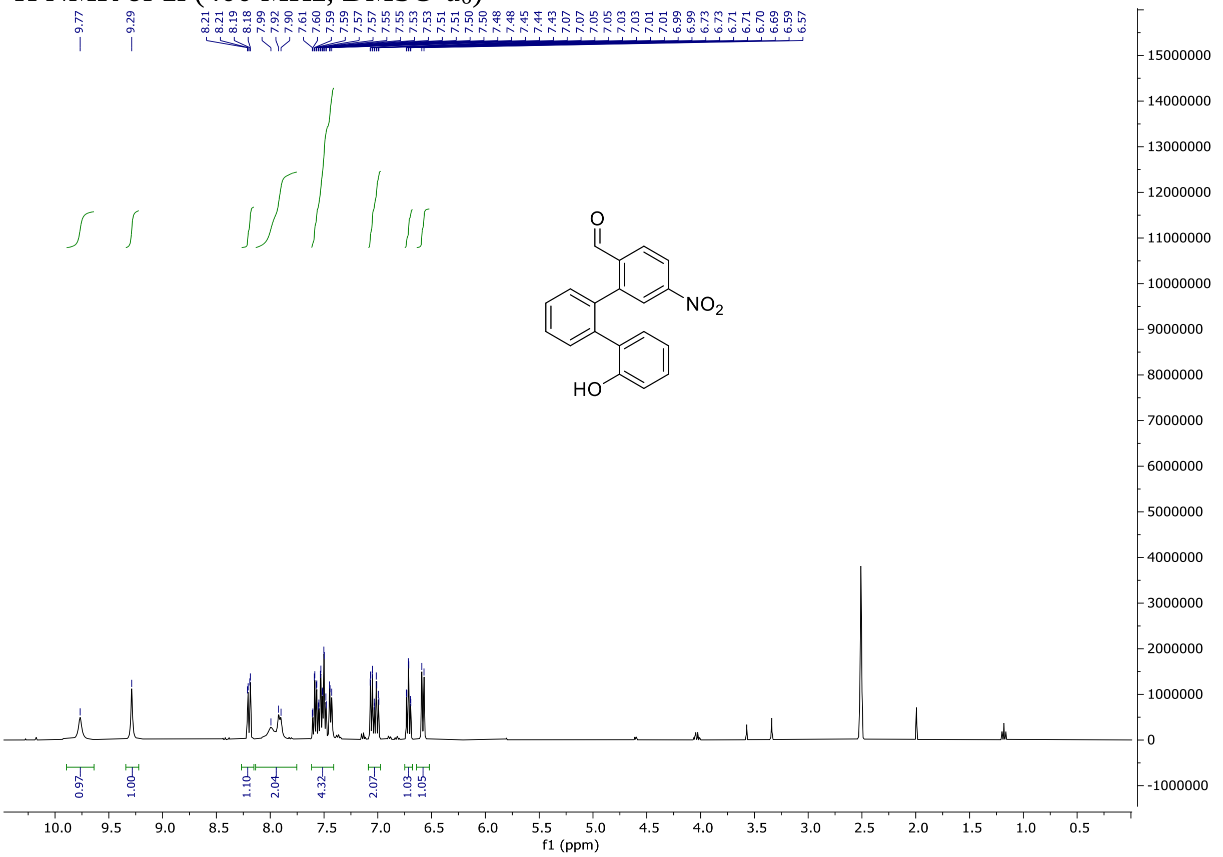

$^{13}C\{^{1}H\}$ NMR of **1l** (101 MHz, DMSO-d_6)

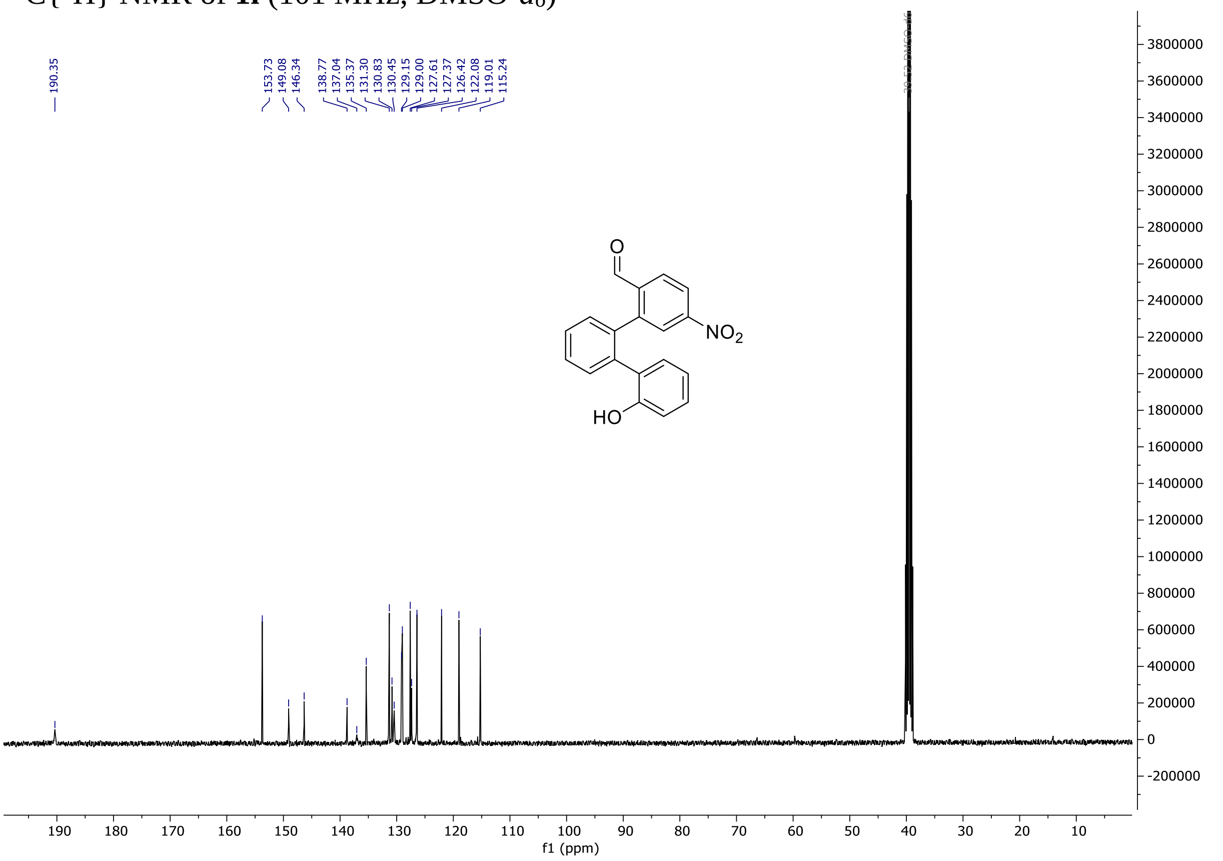

2''-Hydroxy-5''-methyl-[1,1':2',1''-terphenyl]-2-carbaldehyde (1m)

^{1}H NMR of **1m** (400 MHz, DMSO-d_6)

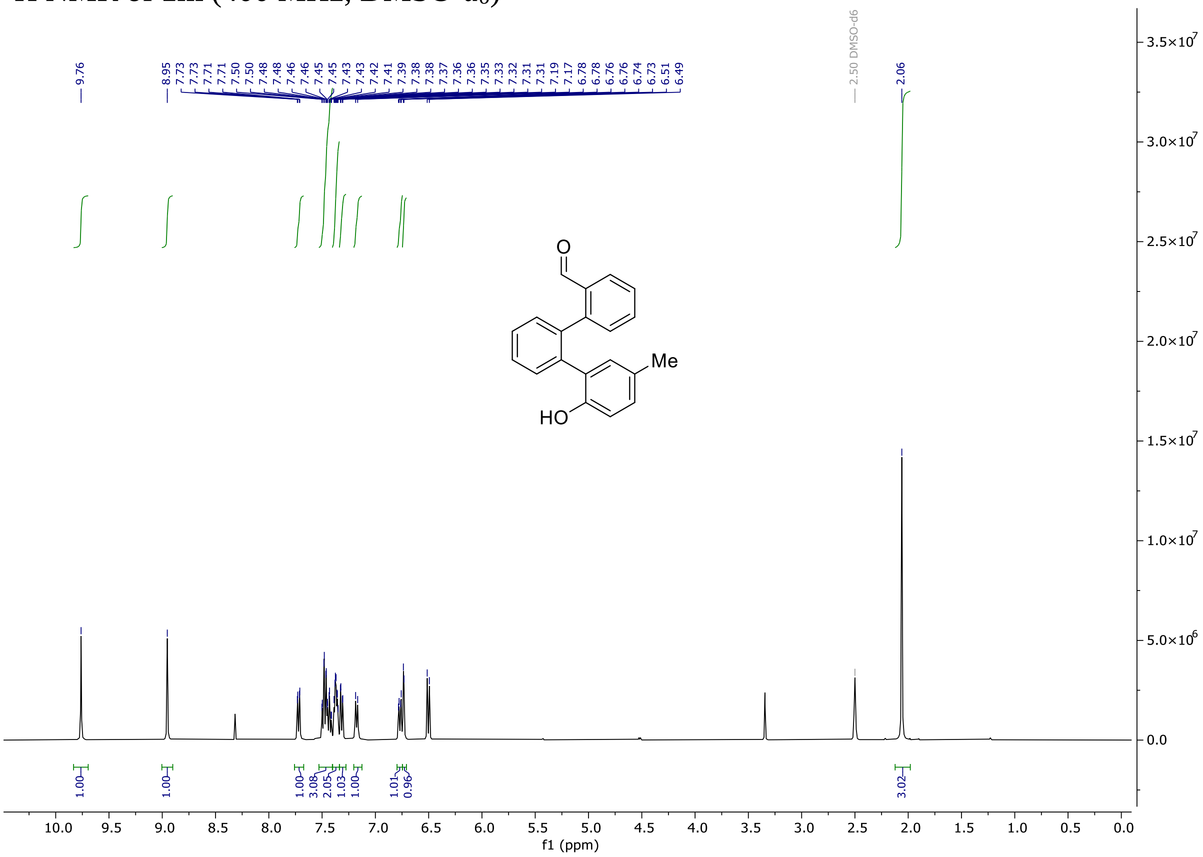

^{13}C{^{1}H} NMR of **1m** (101 MHz, DMSO-d_6)

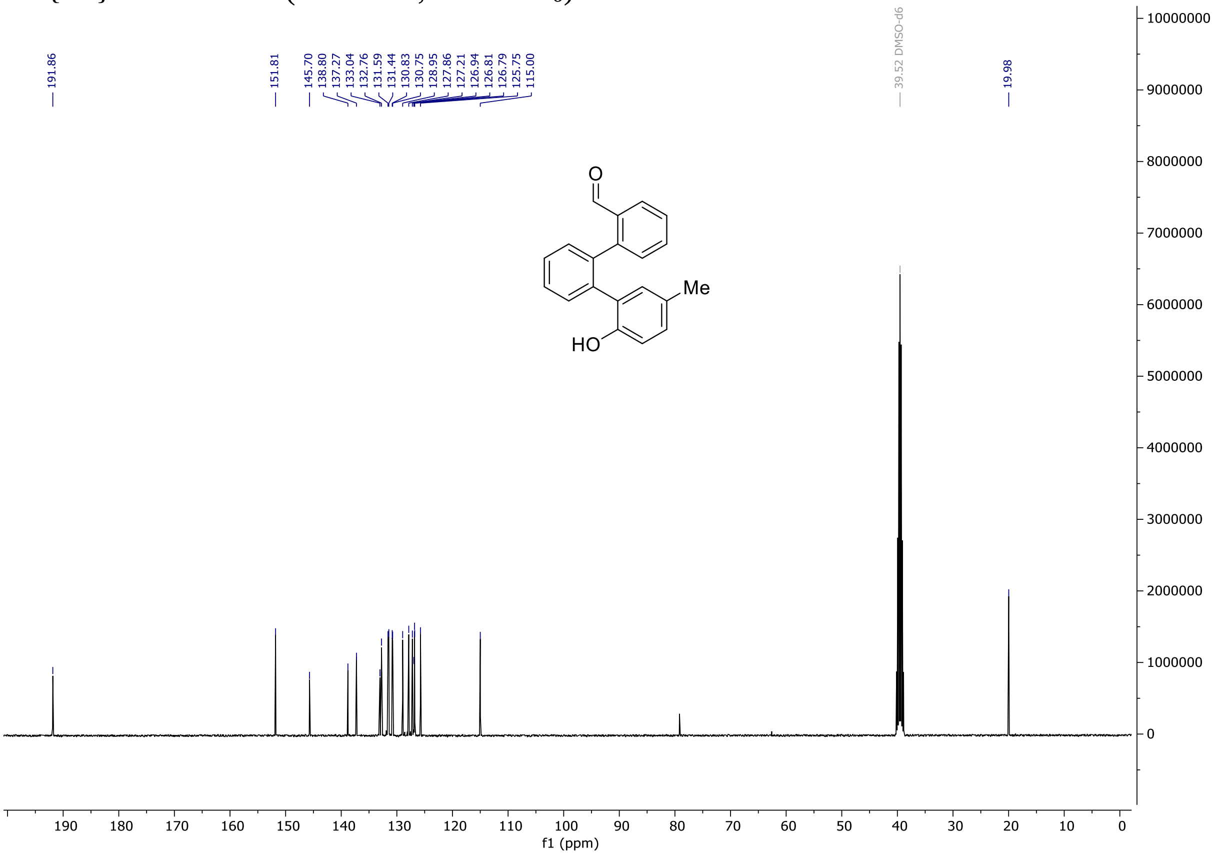

2''-Hydroxy-3''-methyl-[1,1':2',1''-terphenyl]-2-carbaldehyde (1n)

^{1}H NMR of **1n** (400 MHz, DCM-d_2)

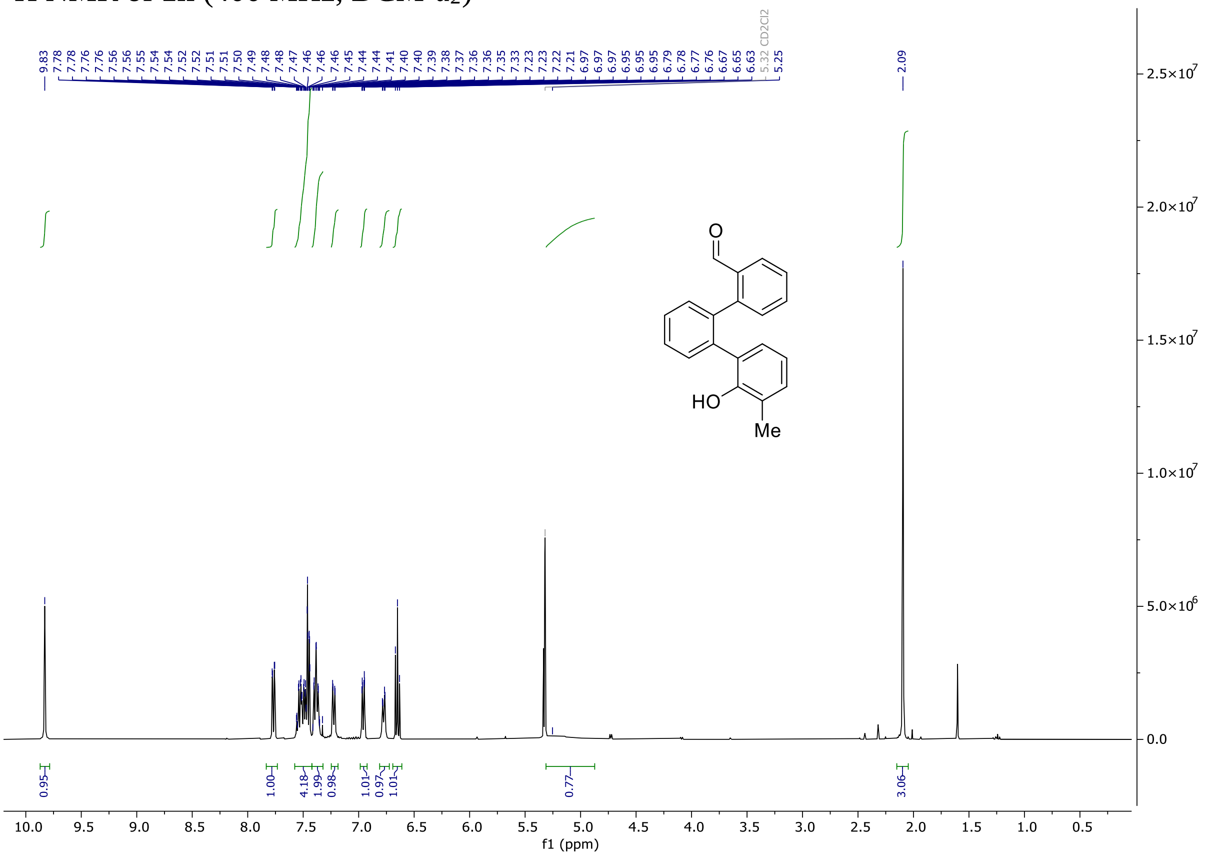

$^{13}C\{^{1}H\}$ NMR of **1n** (101 MHz, DCM-d_2)

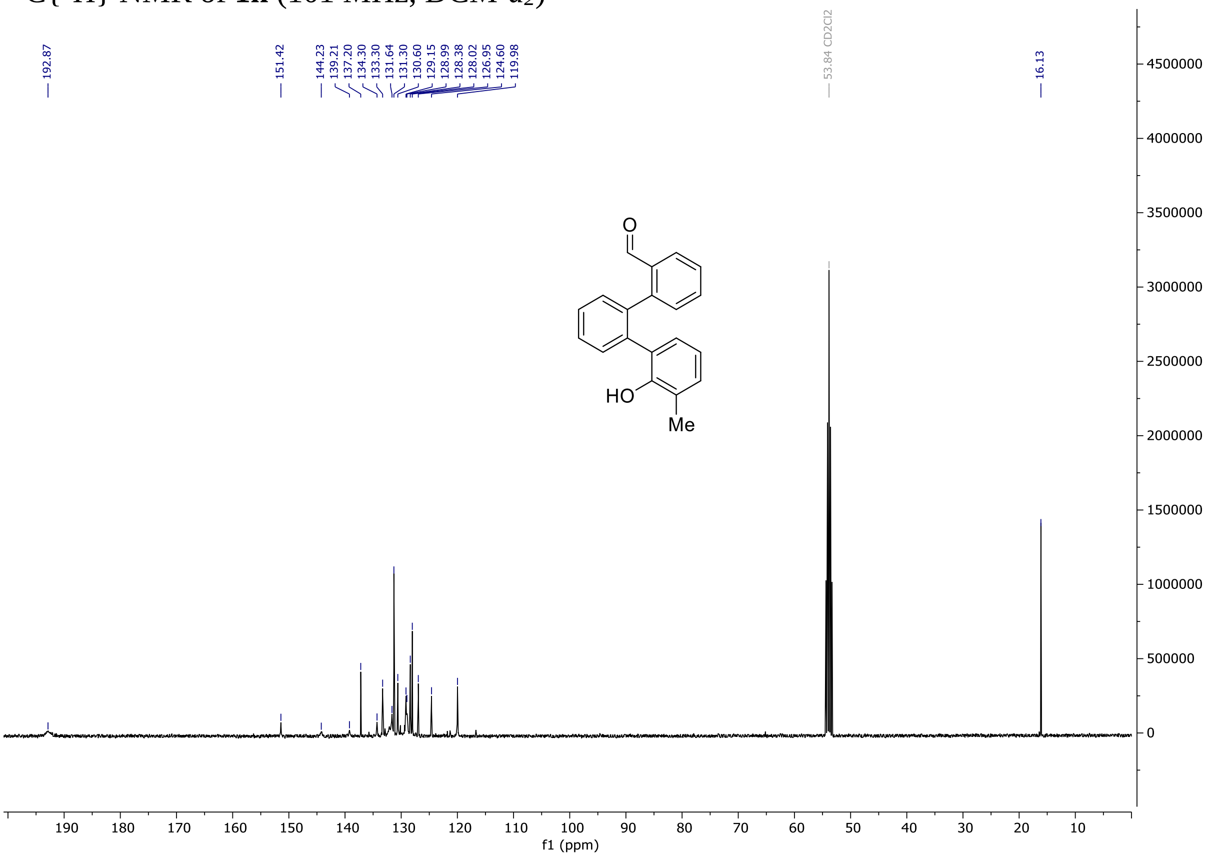

2''-Hydroxy-4''-methyl-[1,1':2',1''-terphenyl]-2-carbaldehyde (1o)

^{1}H NMR of **1o** (400 MHz, DCM-d_2)

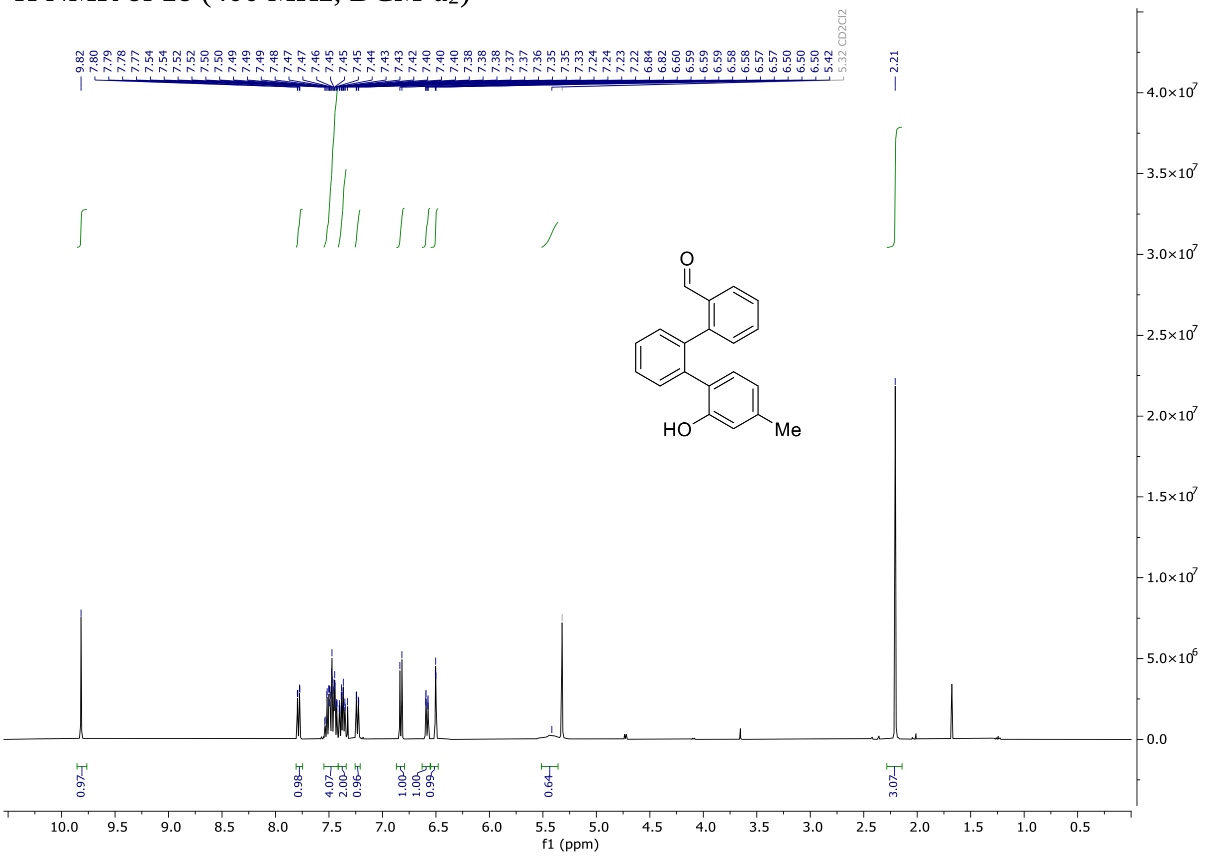

$^{13}C\{^{1}H\}$ NMR of **1o** (101 MHz, DCM-d_2)

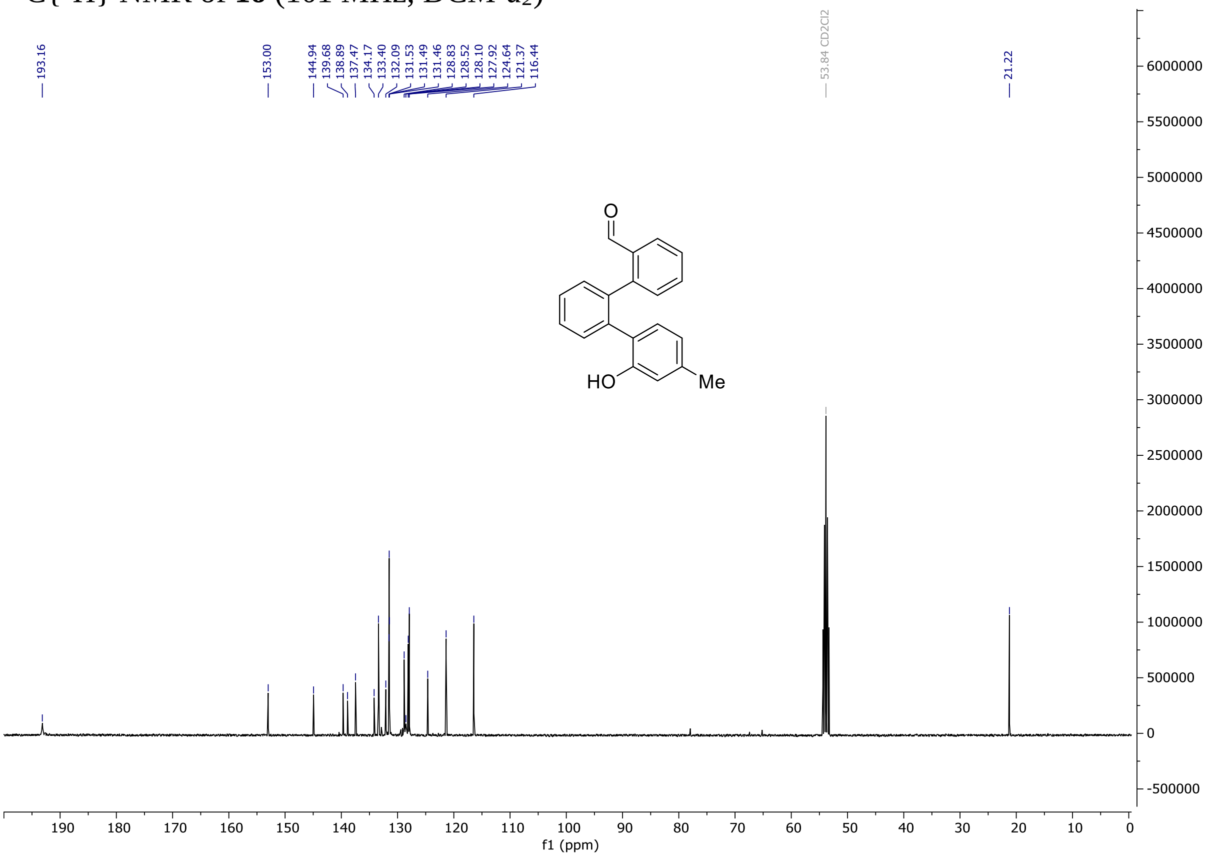

2''-Hydroxy-6''-methyl-[1,1':2',1''-terphenyl]-2-carbaldehyde (1p)

^{1}H NMR of **1p** (400 MHz, DMSO-d_6, 25 °C)

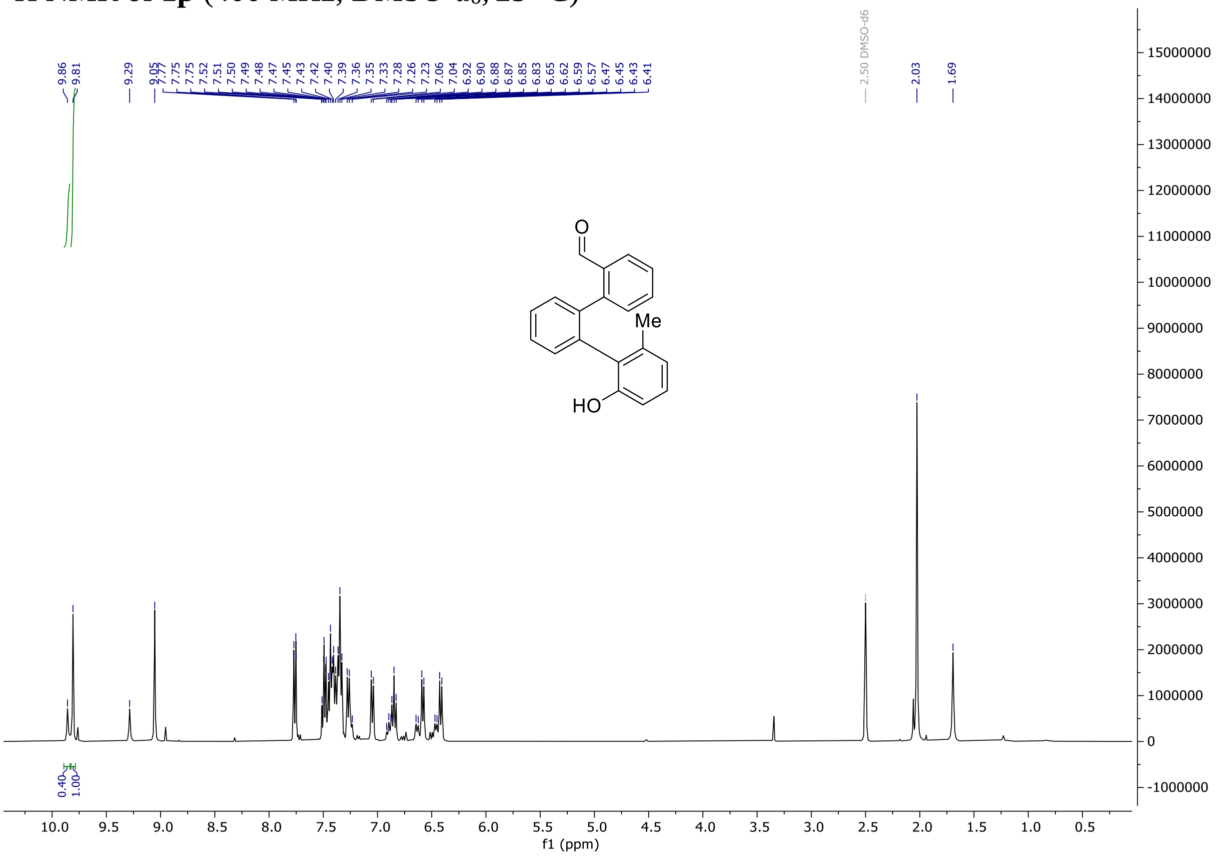

^{1}H NMR of **1p** (600 MHz, DMSO-d_6, 80 °C)

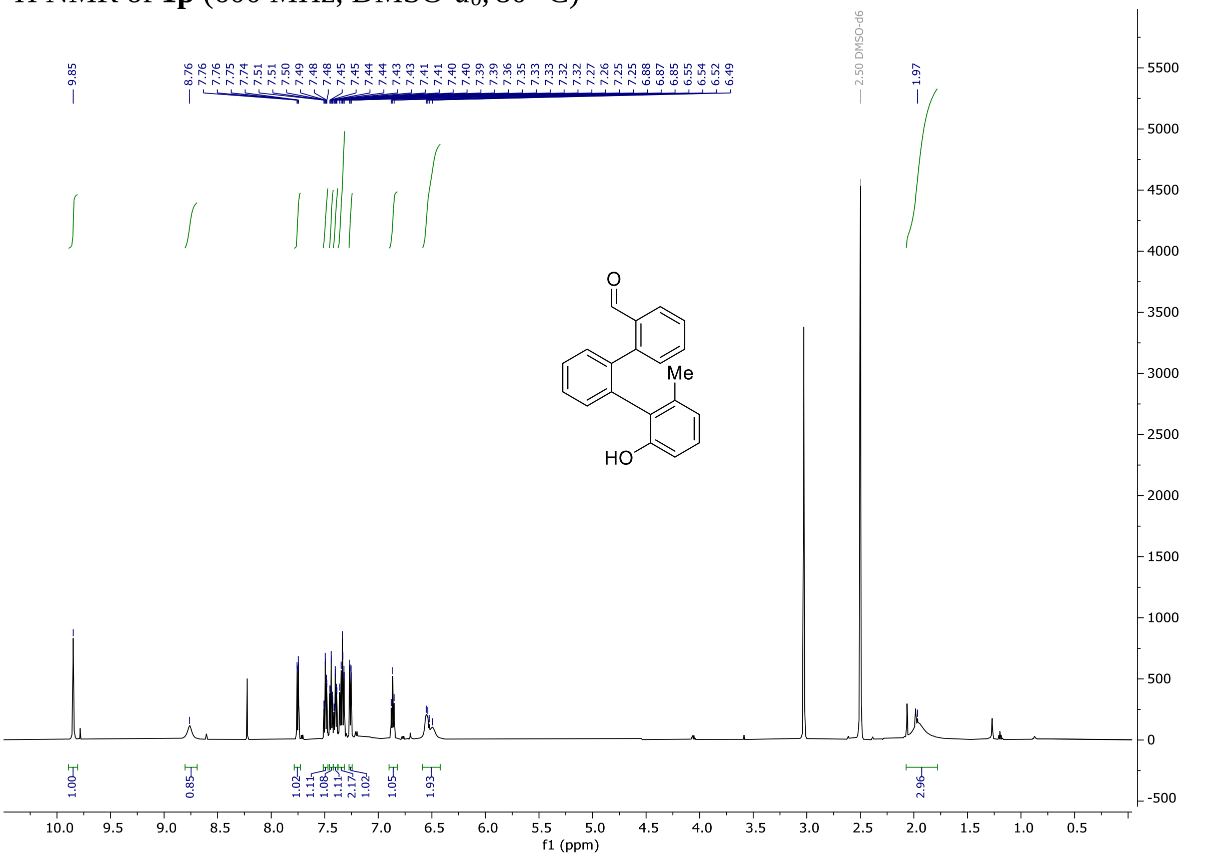

$^{13}C\{^{1}H\}$ NMR of **1p** (101 MHz, DMSO-*d*$_6$, 25 °C)

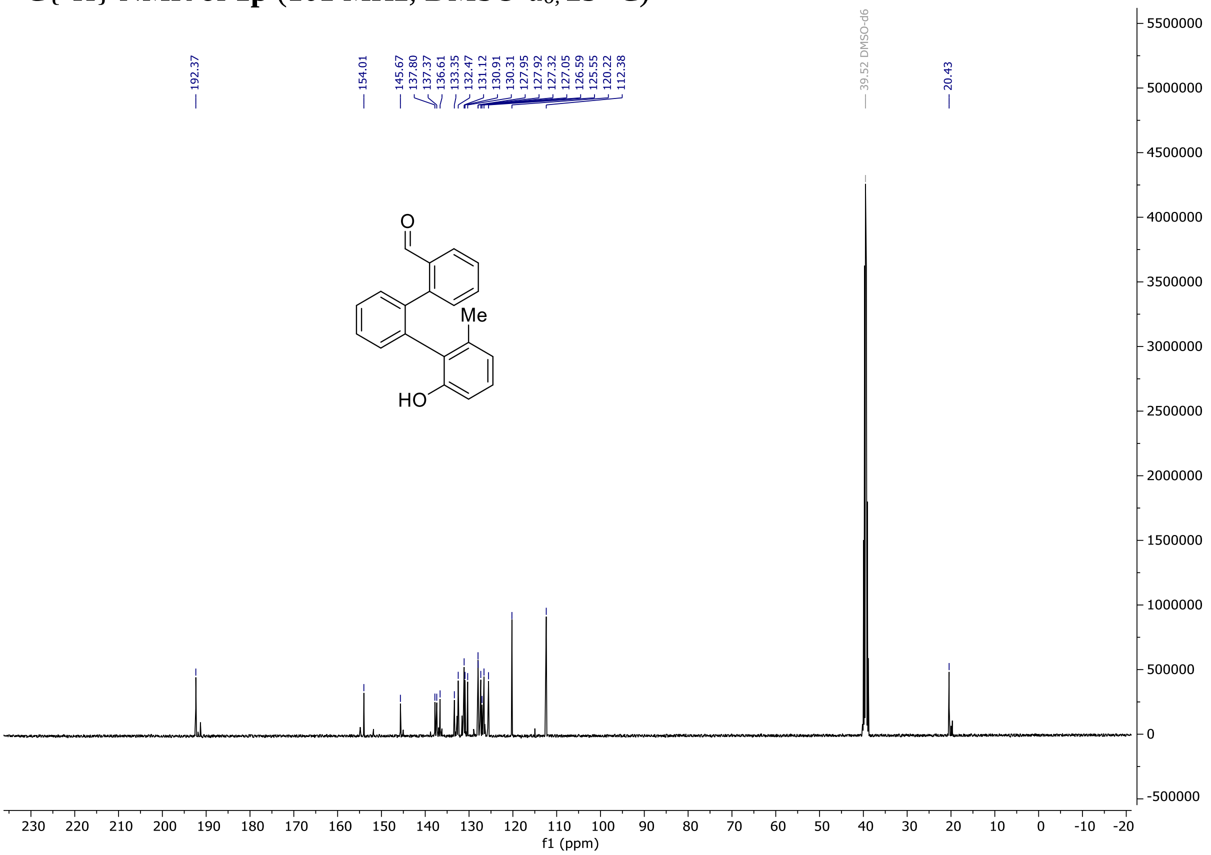

^{1}H NMR of **1p** (600 MHz, DMSO-*d*$_6$, various temperatures)

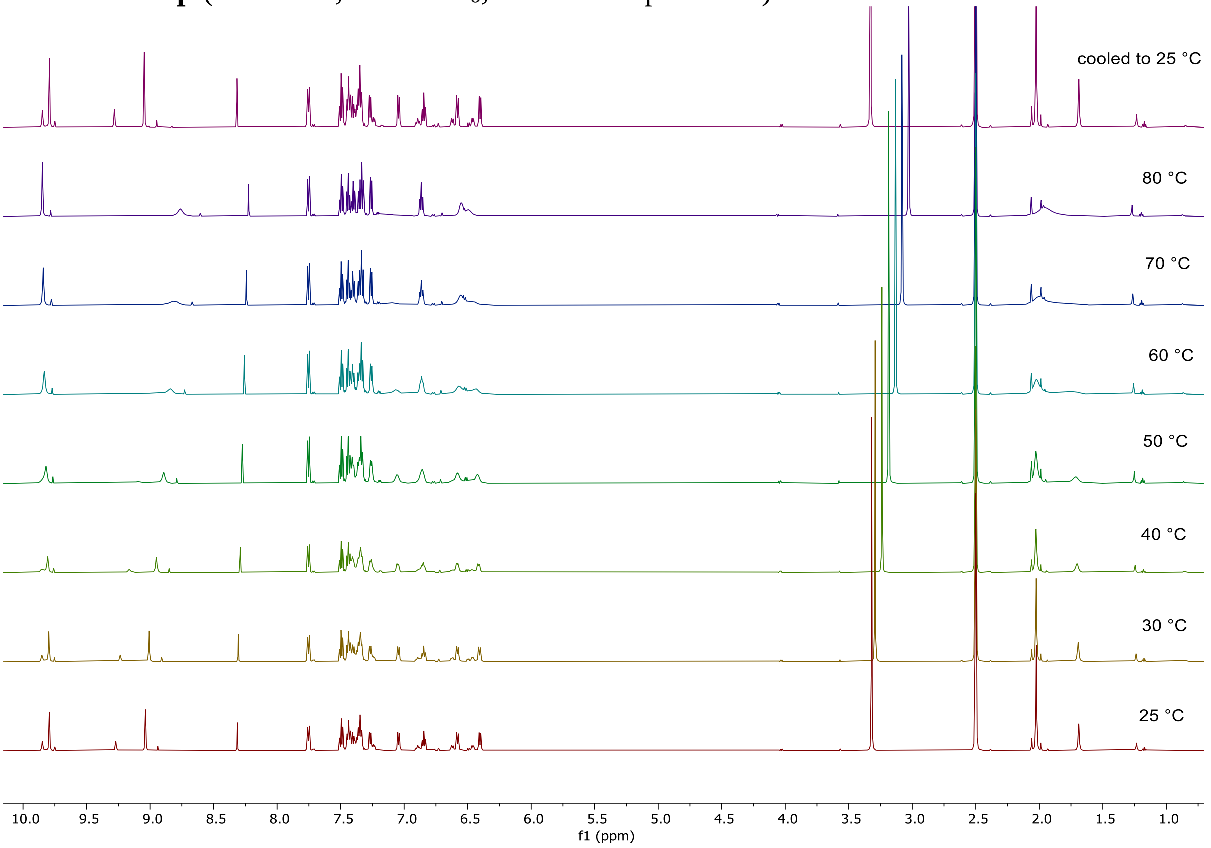

5''-Fluoro-2''-hydroxy-[1,1':2',1''-terphenyl]-2-carbaldehyde (1q)

^{1}H NMR of **1q** (400 MHz, DMSO-d_6)

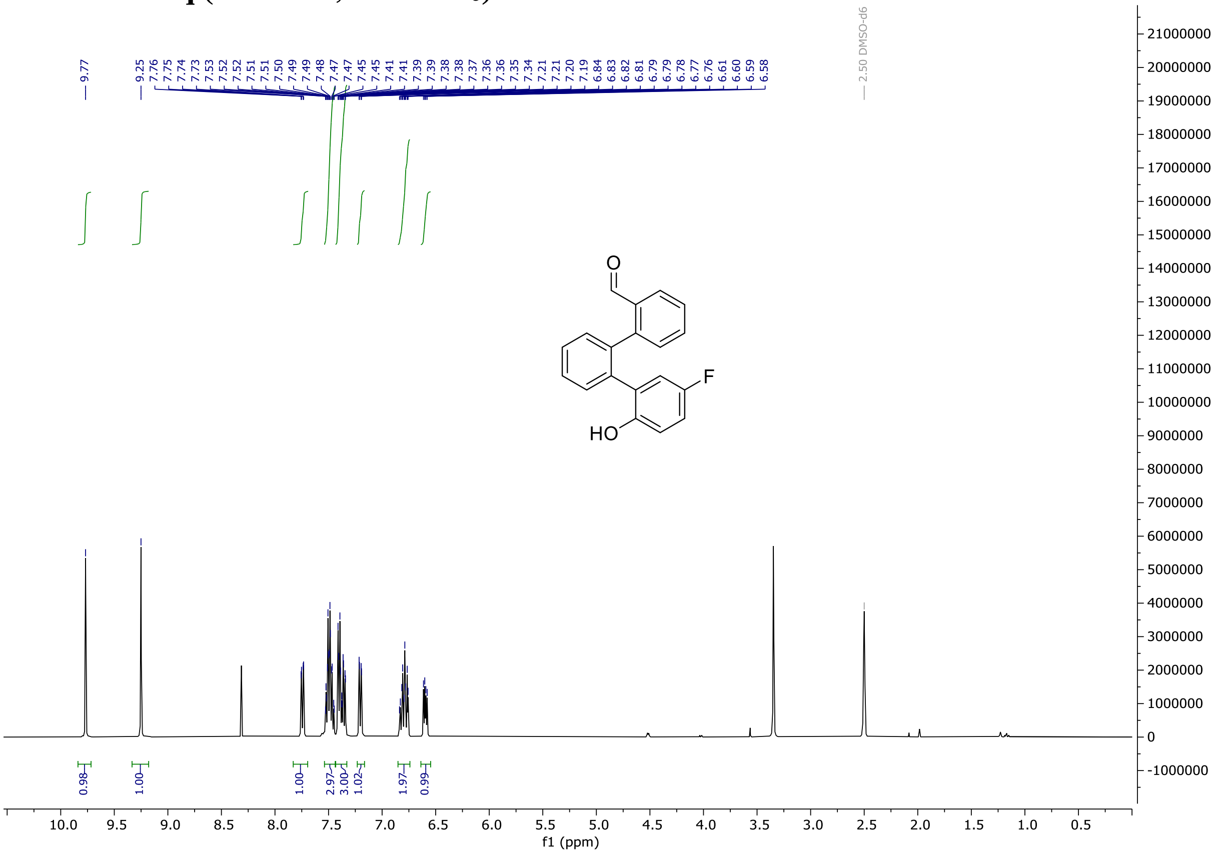

^{13}C{^{1}H} NMR of **1q** (101 MHz, DMSO-d_6)

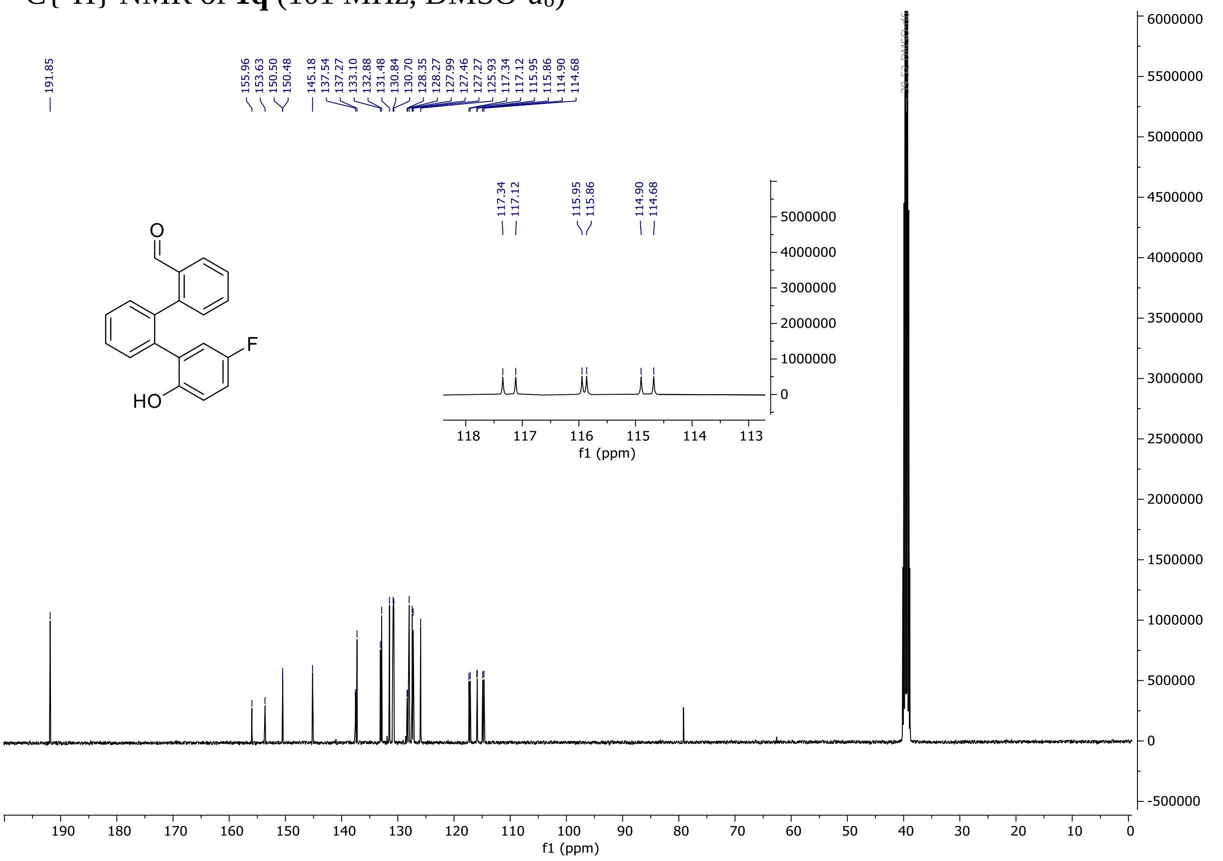

^{19}F NMR of **1q** (376 MHz, DMSO-d_6)

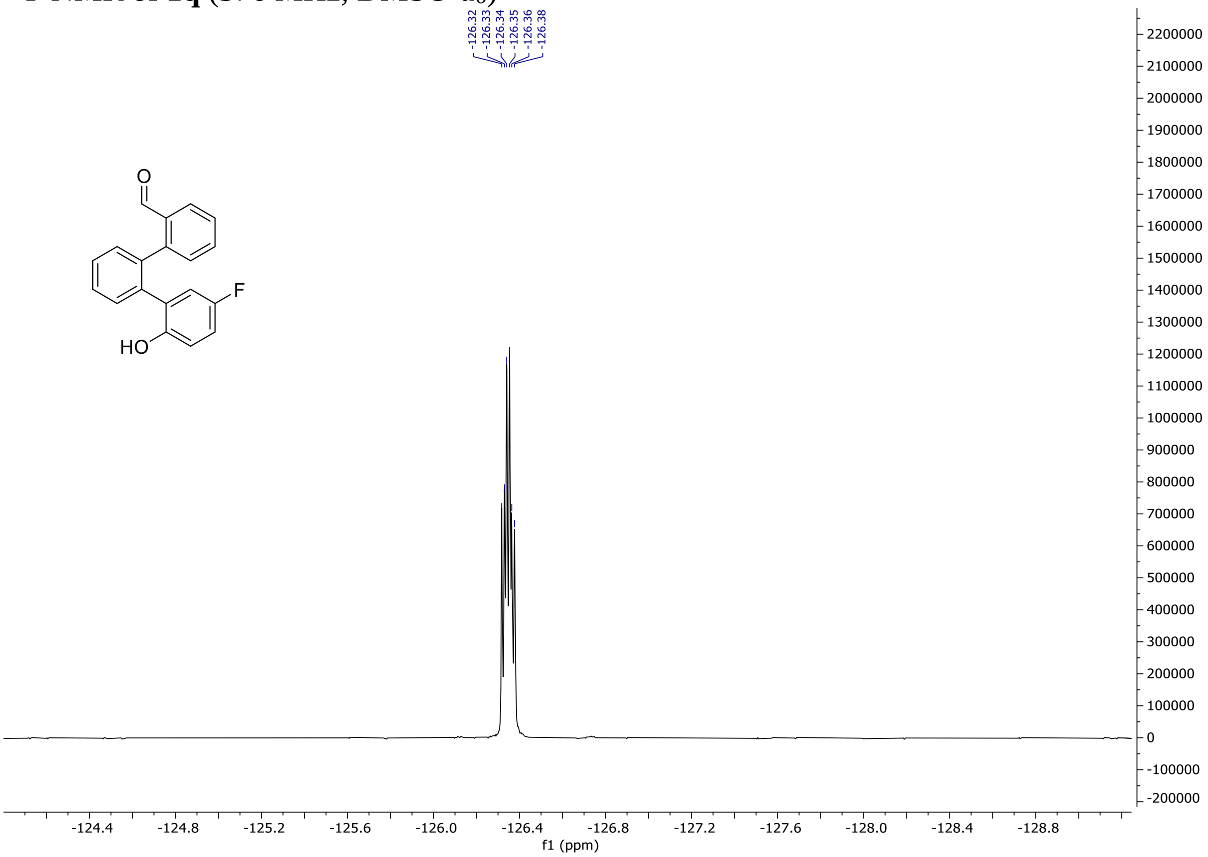

5''-Chloro-2''-hydroxy-[1,1':2',1''-terphenyl]-2-carbaldehyde (1r)

^{1}H NMR of **1r** (400 MHz, DCM-d_2)

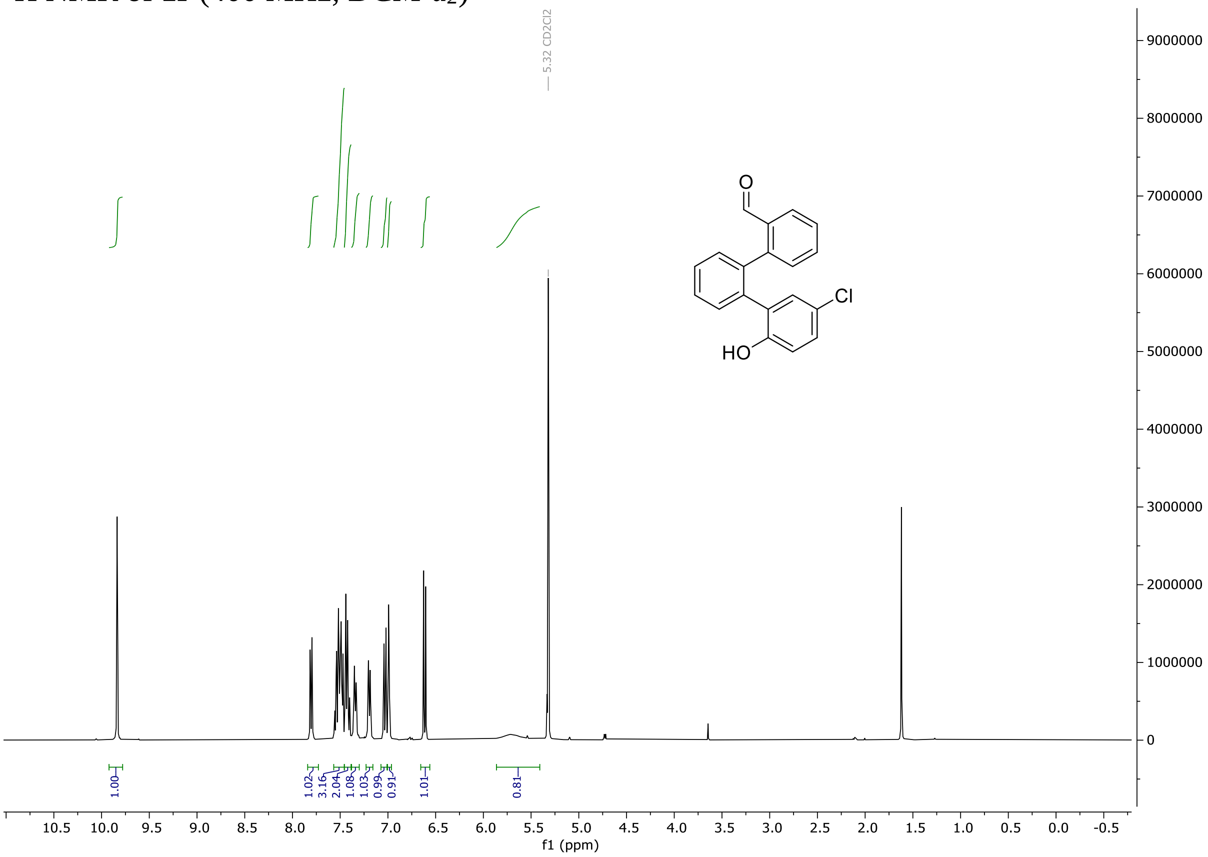

^{13}C{^{1}H} NMR of **1r** (101 MHz, DCM-d_2)

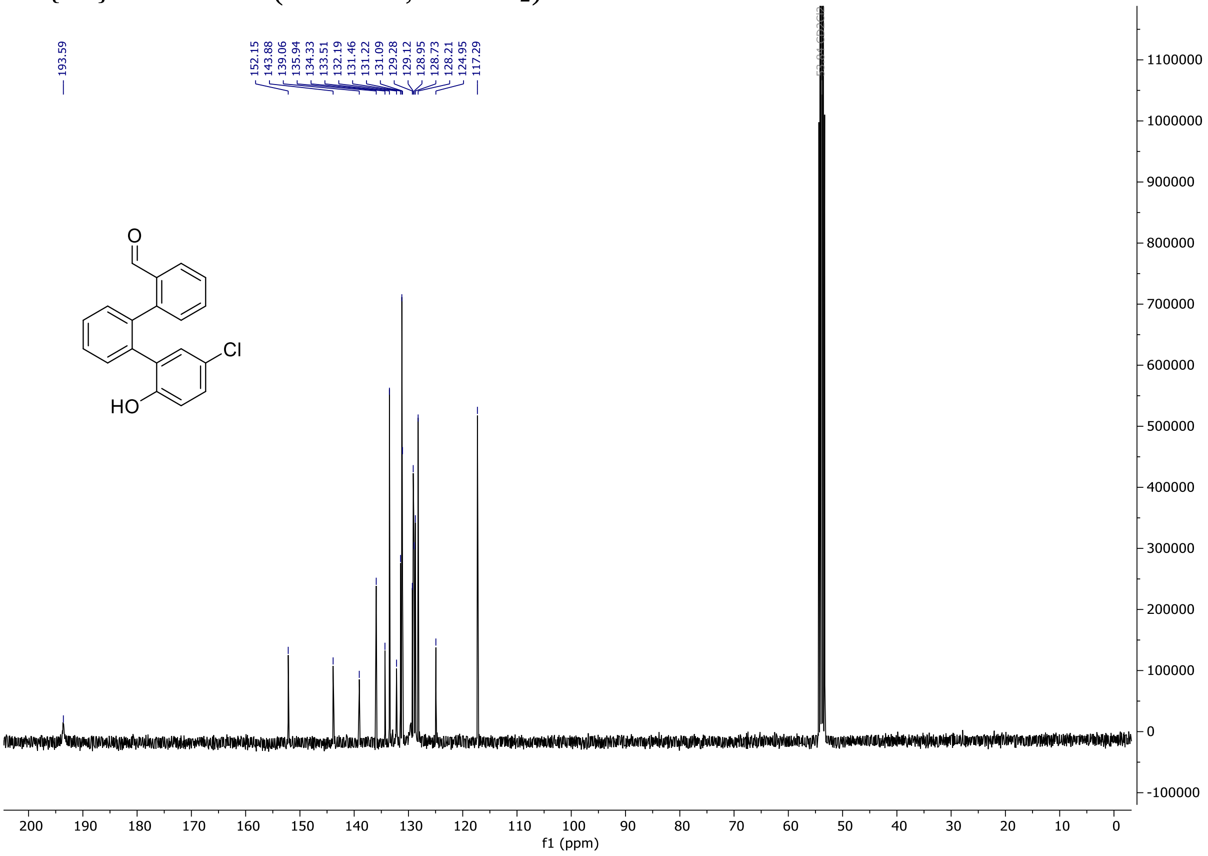

5''-Bromo-2''-hydroxy-[1,1':2',1''-terphenyl]-2-carbaldehyde (1s)

^{1}H NMR of **1s** (400 MHz, DCM-d_2)

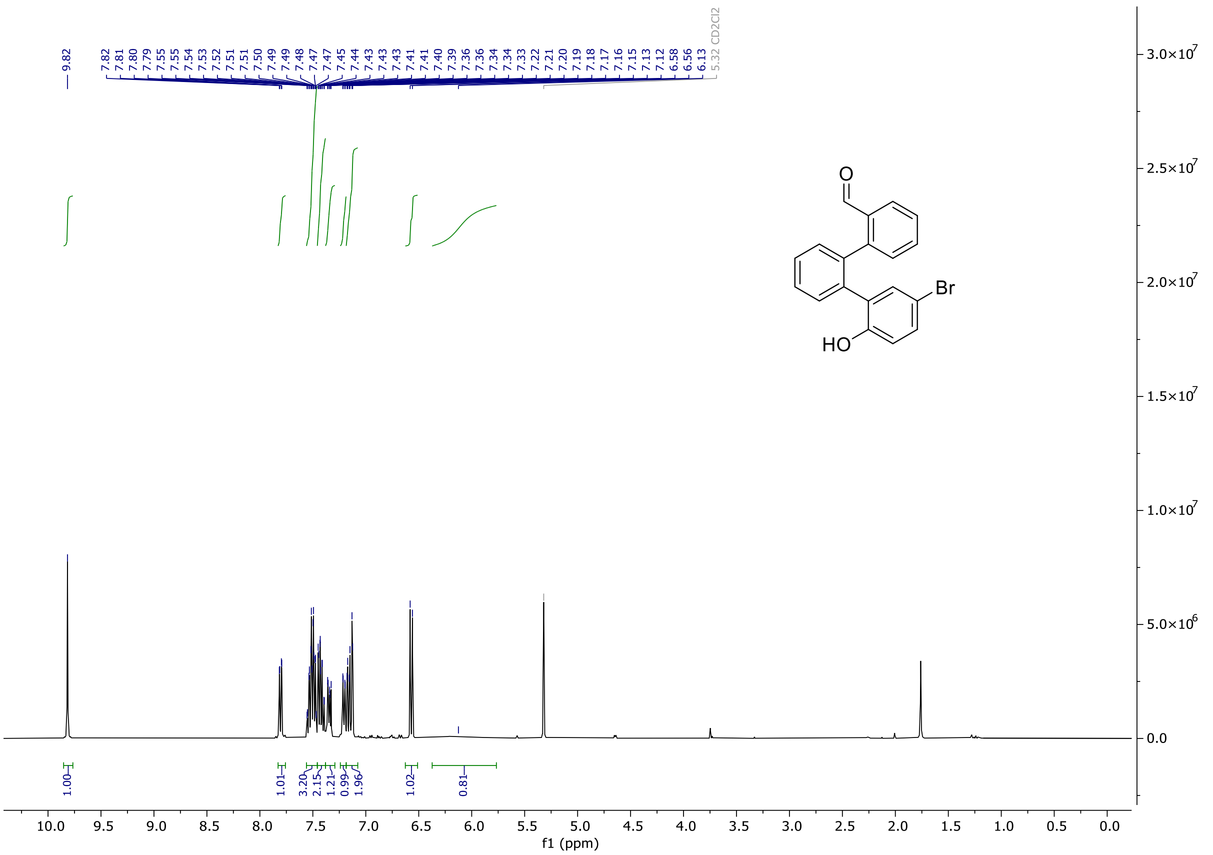

$^{13}C\{^{1}H\}$ NMR of **1s** (101 MHz, DCM-d_2)

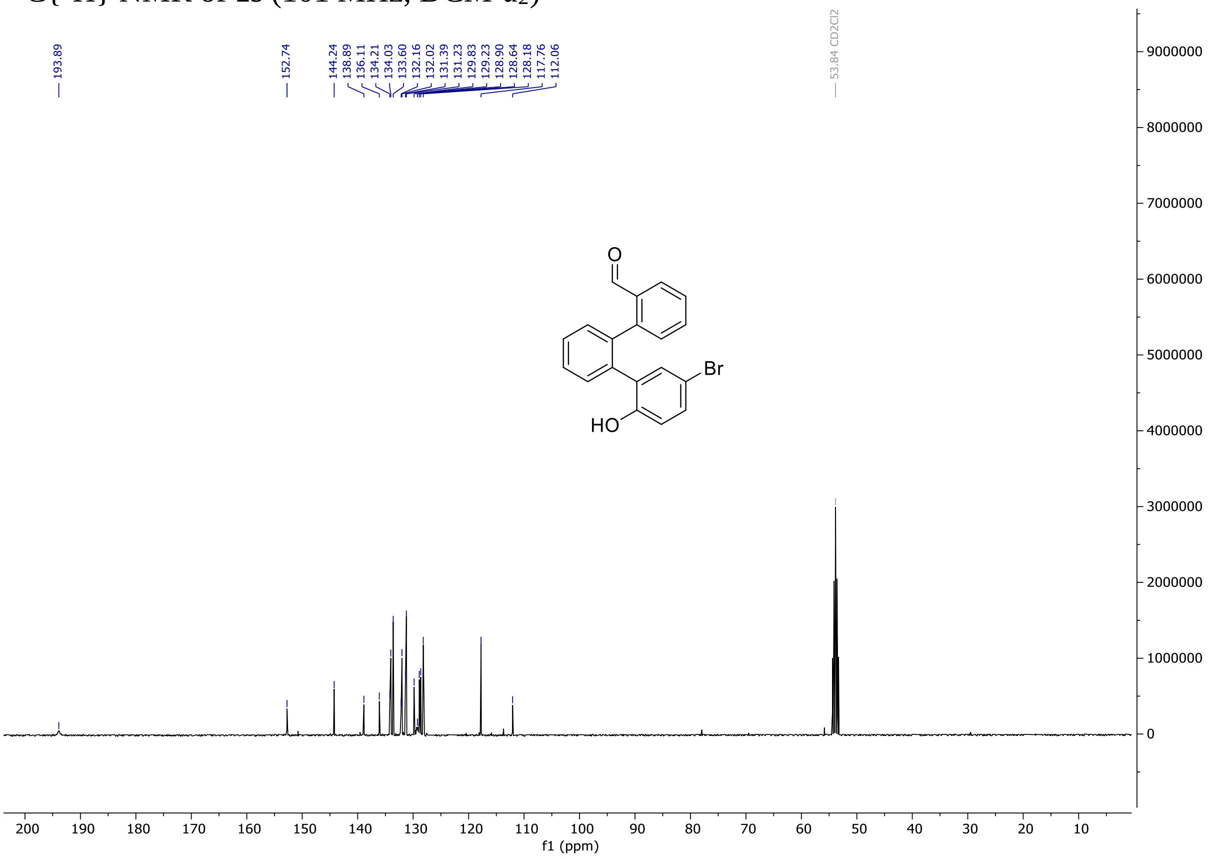

2''-Hydroxy-5'-methyl-[1,1':2',1''-terphenyl]-2-carbaldehyde (1t)

^{1}H NMR of **1t** (400 MHz, DMSO-d_6)

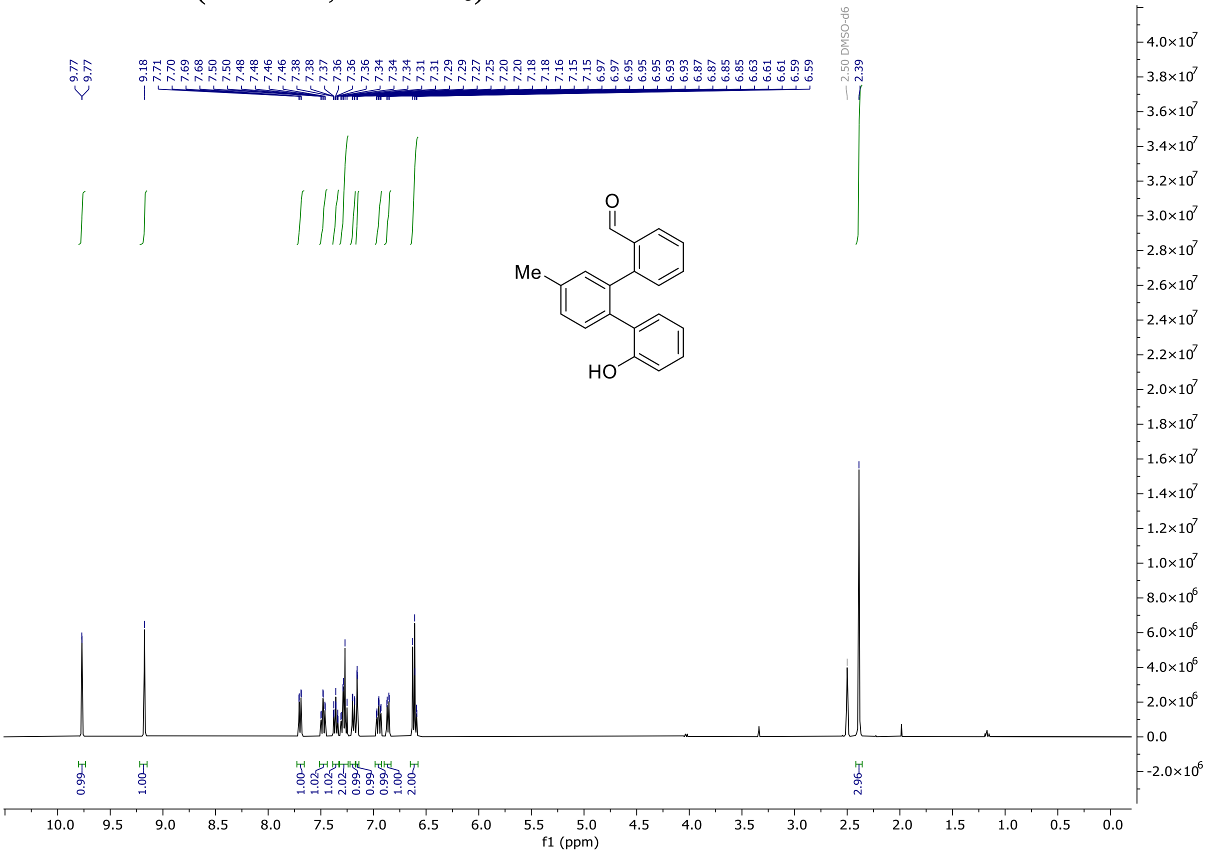

^{13}C{^{1}H} NMR of **1t** (101 MHz, DMSO-d_6)

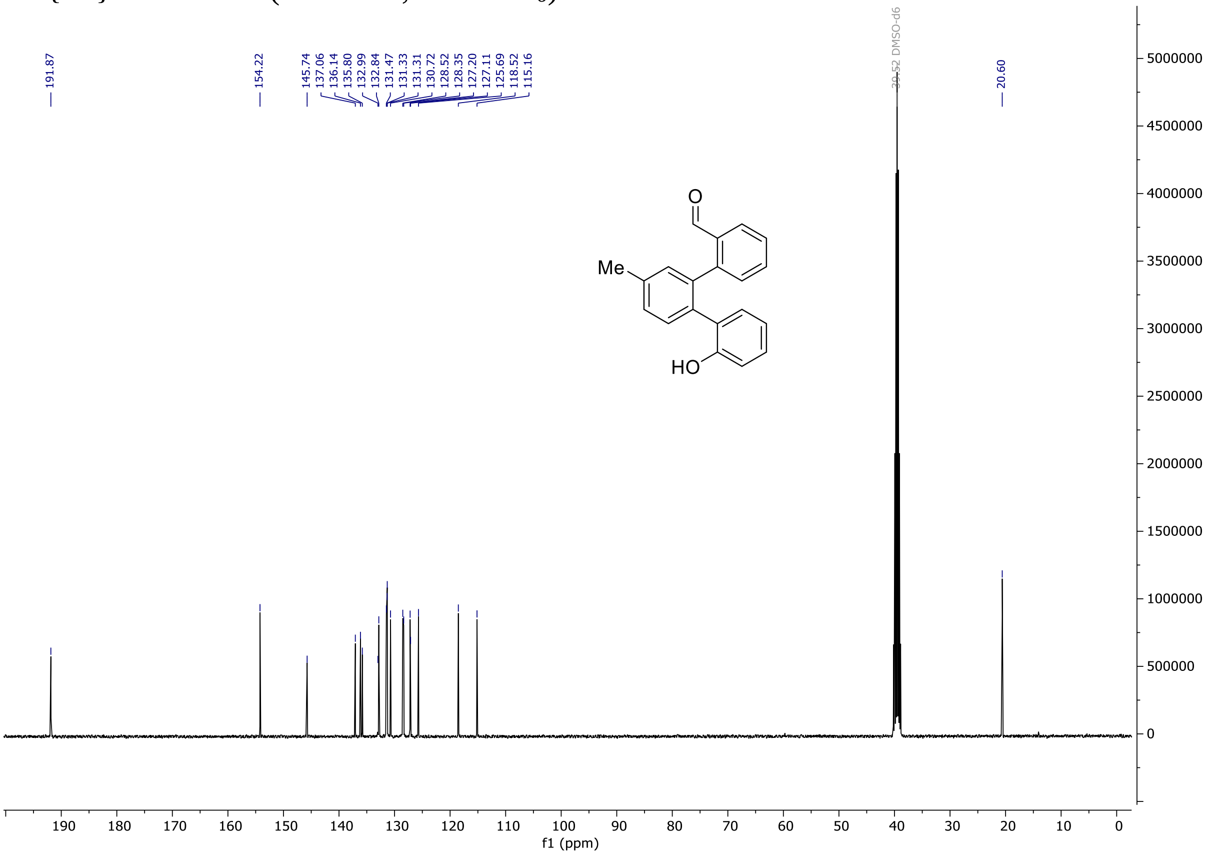

2''-Hydroxy-4'-methyl-[1,1':2',1''-terphenyl]-2-carbaldehyde (1u)

^{1}H NMR of **1u** (400 MHz, DMSO-d_6)

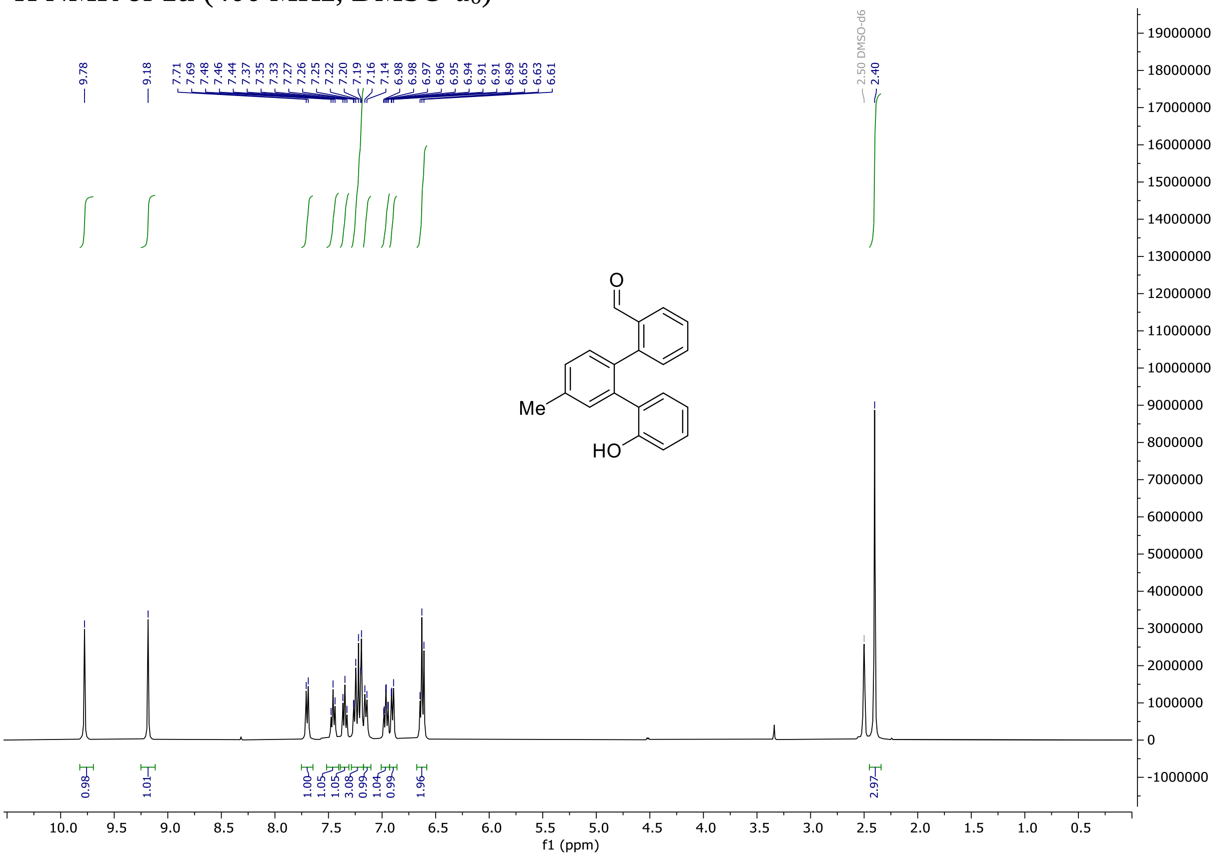

^{13}C{^{1}H} NMR of **1u** (101 MHz, DMSO-d_6)

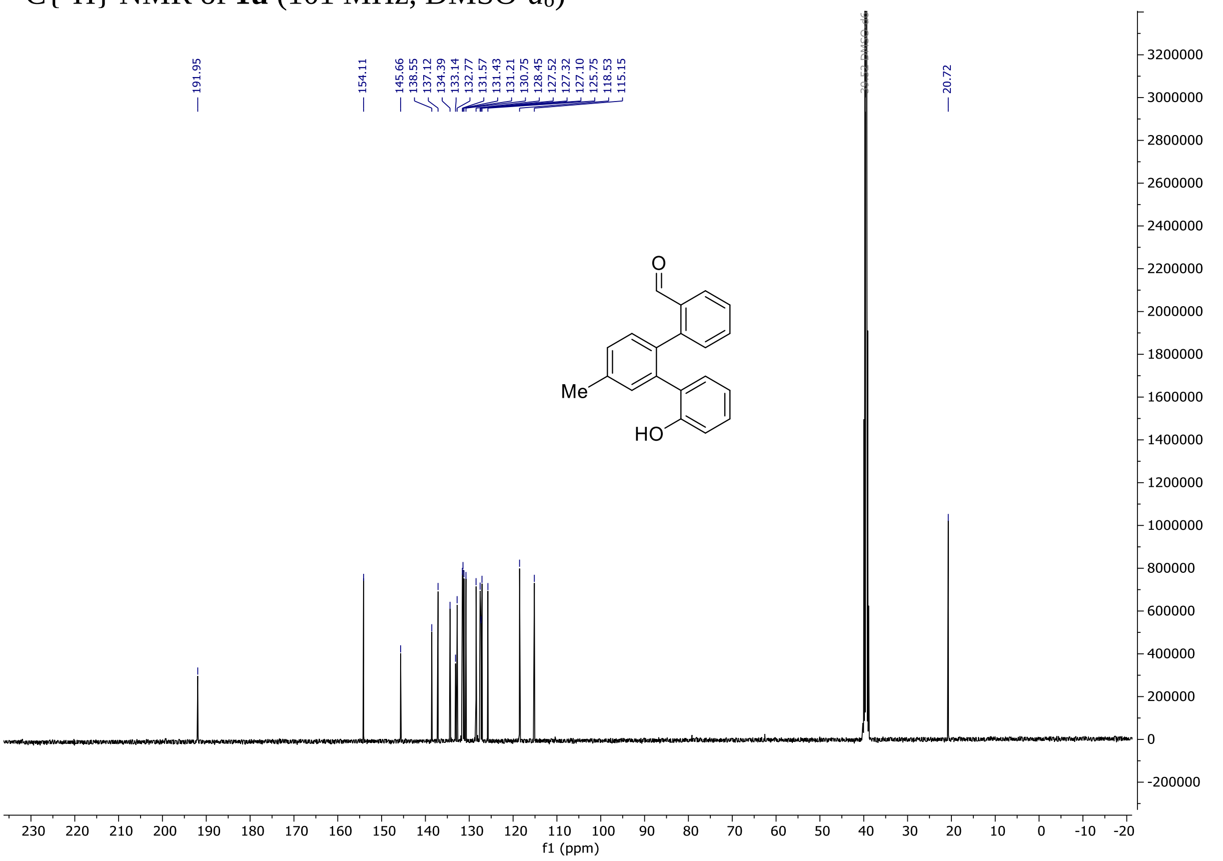

2''-Hydroxy-5,5''-dimethyl-[1,1':2',1''-terphenyl]-2-carbaldehyde (1v)

^{1}H NMR of **1v** (400 MHz, DMSO-d_6)

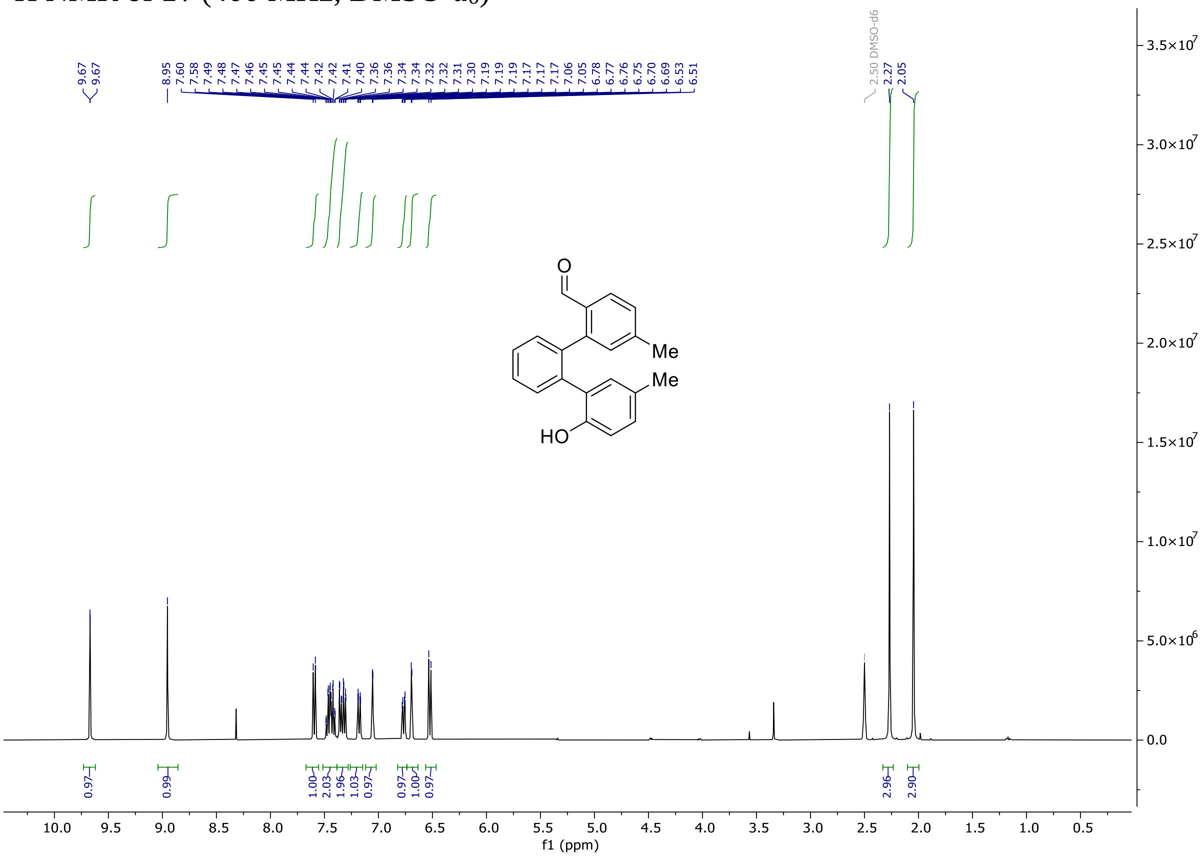

$^{13}C\{^{1}H\}$ NMR of **1v** (101 MHz, DMSO-d_6)

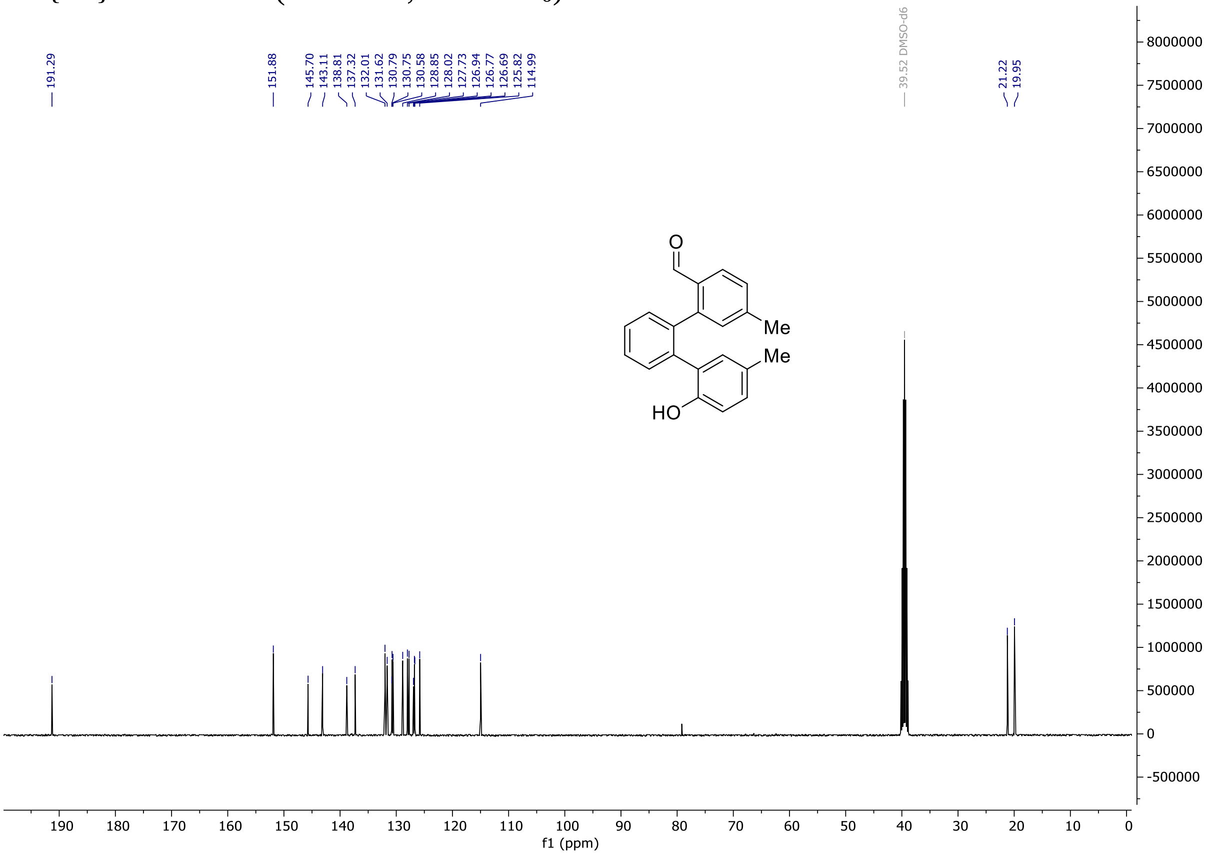

5,5''-Difluoro-2''-hydroxy-[1,1':2',1''-terphenyl]-2-carbaldehyde (1w)

^{1}H NMR of **1w** (400 MHz, DCM-d_2)

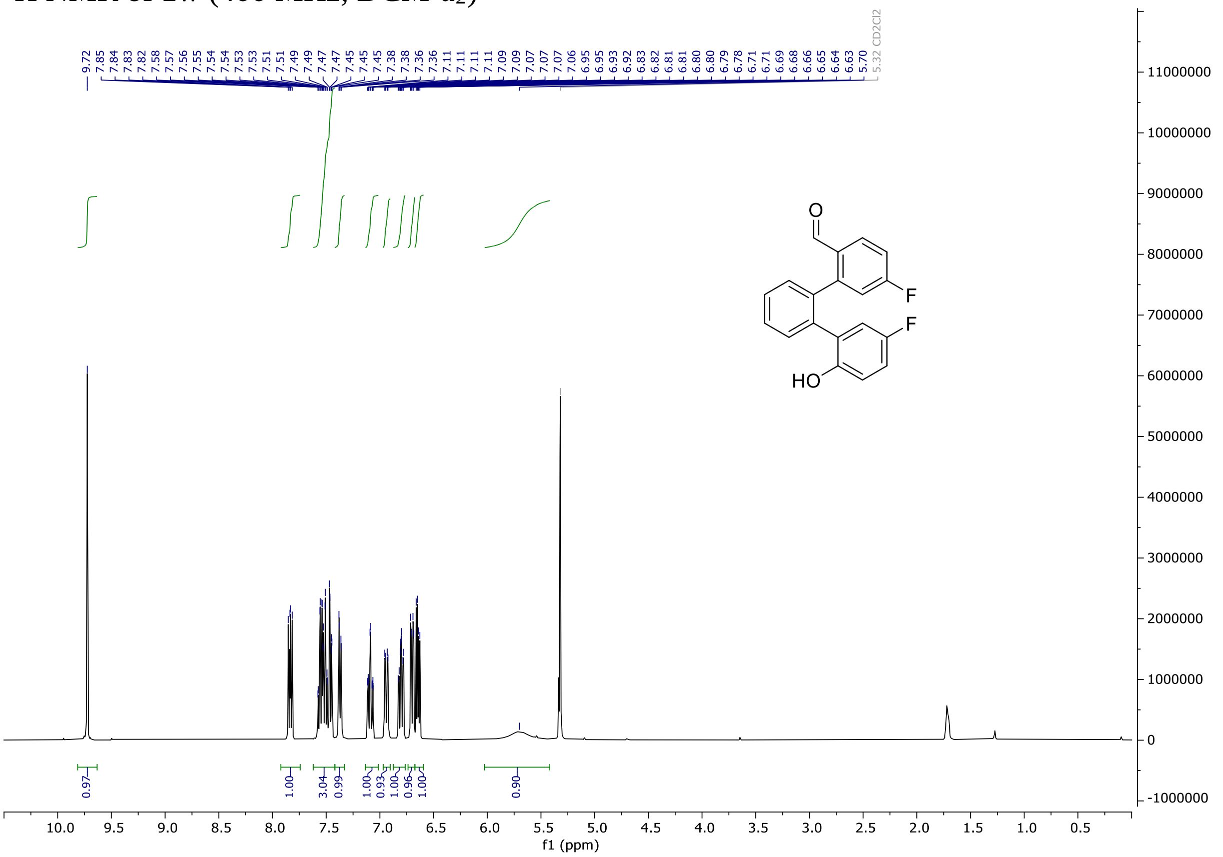

$^{13}C\{^{1}H\}$ NMR of **1w** (101 MHz, DCM-d_2)

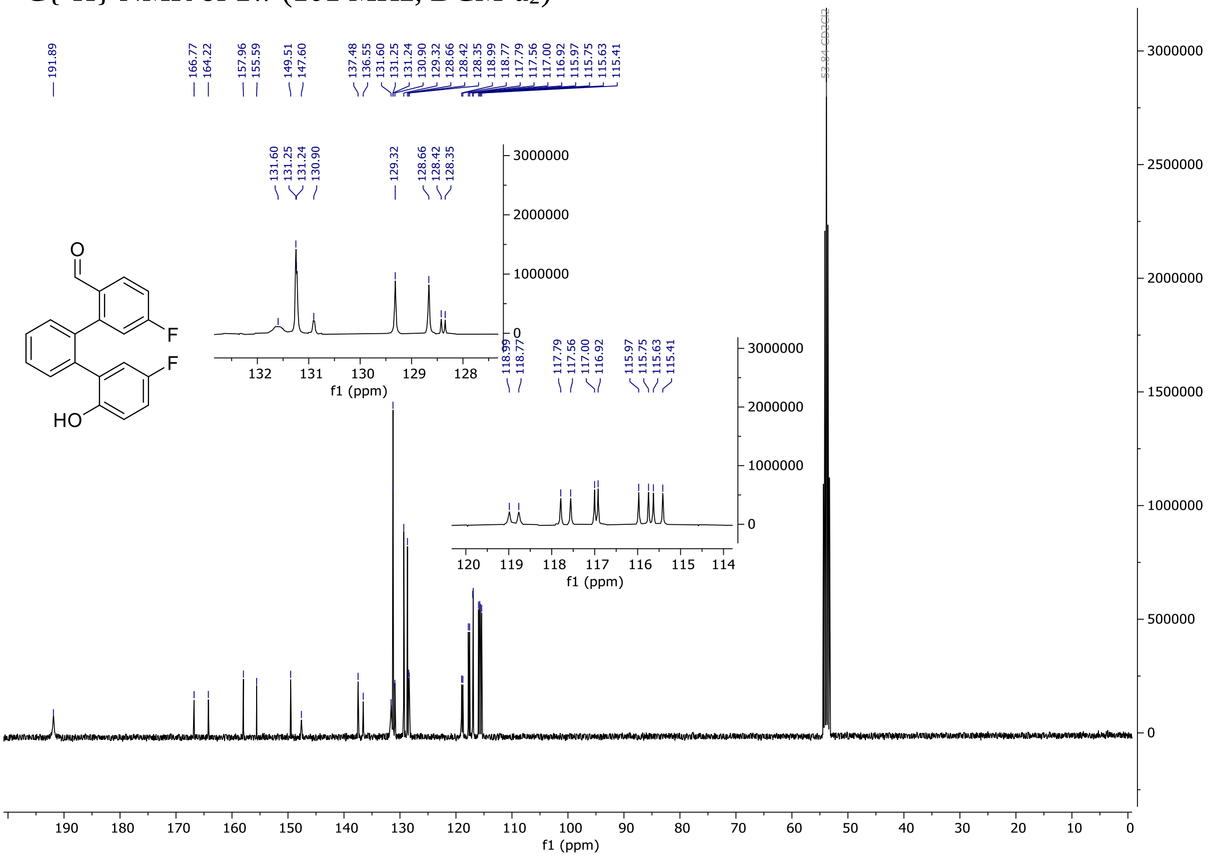

^{19}F NMR of **1w** (376 MHz, DCM-d_2)

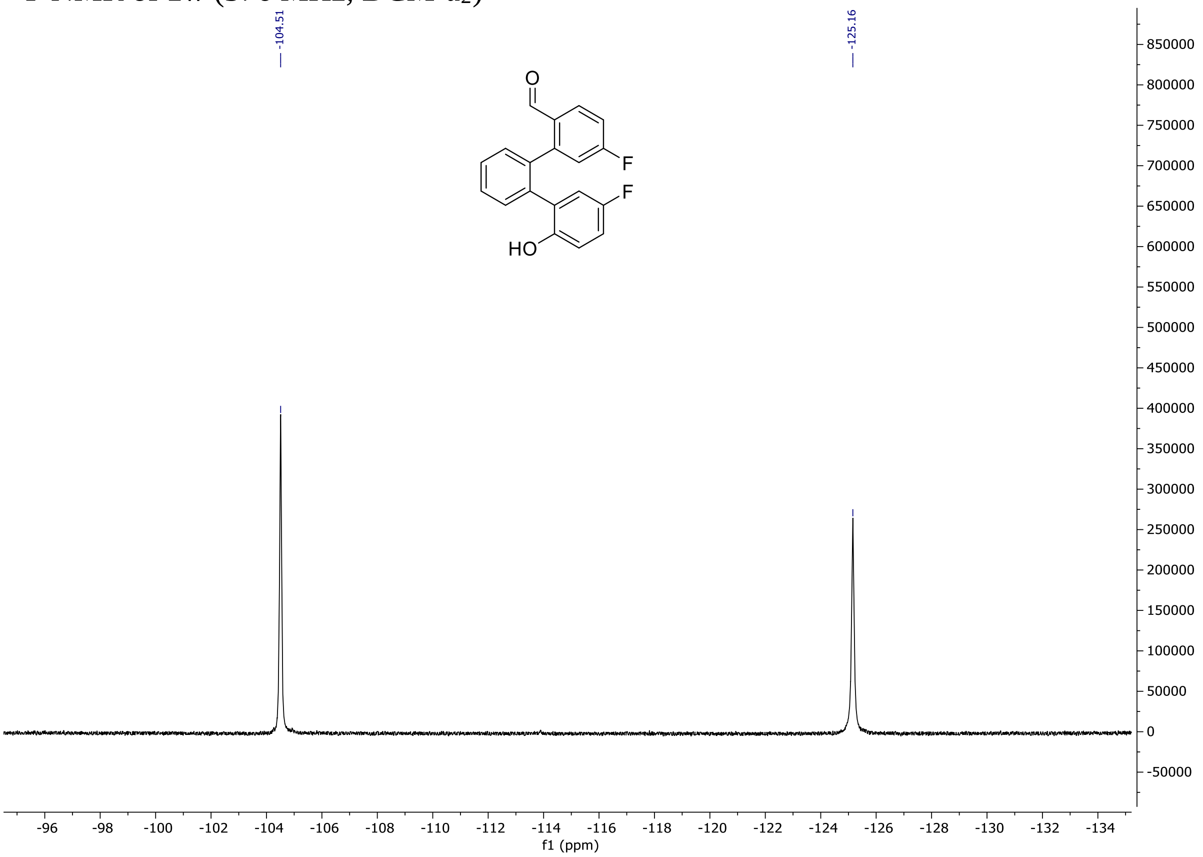

5-Fluoro-2''-hydroxy-5''-methyl-[1,1':2',1''-terphenyl]-2-carbaldehyde (1x)

^{1}H NMR of **1x** (400 MHz, DCM-d_2)

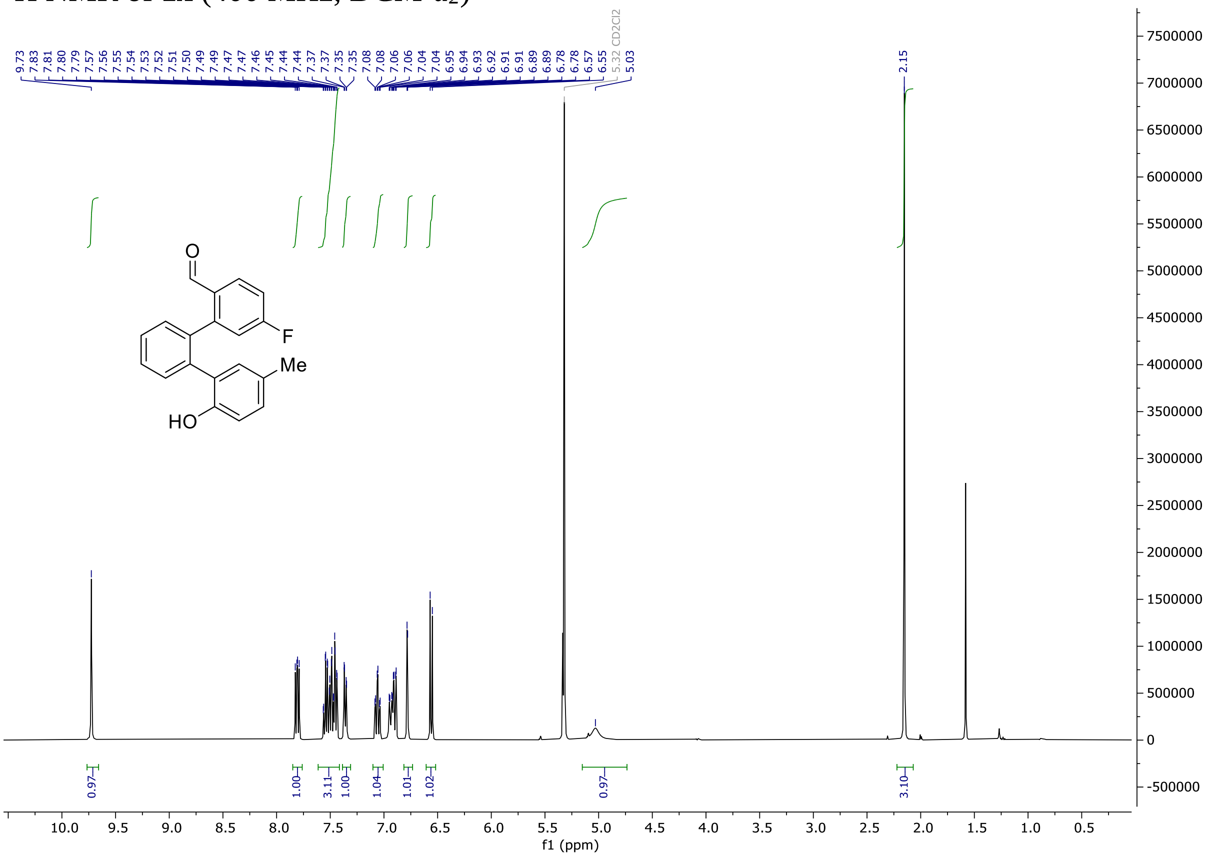

^{13}C{^{1}H} NMR of **1x** (101 MHz, DCM-d_2)

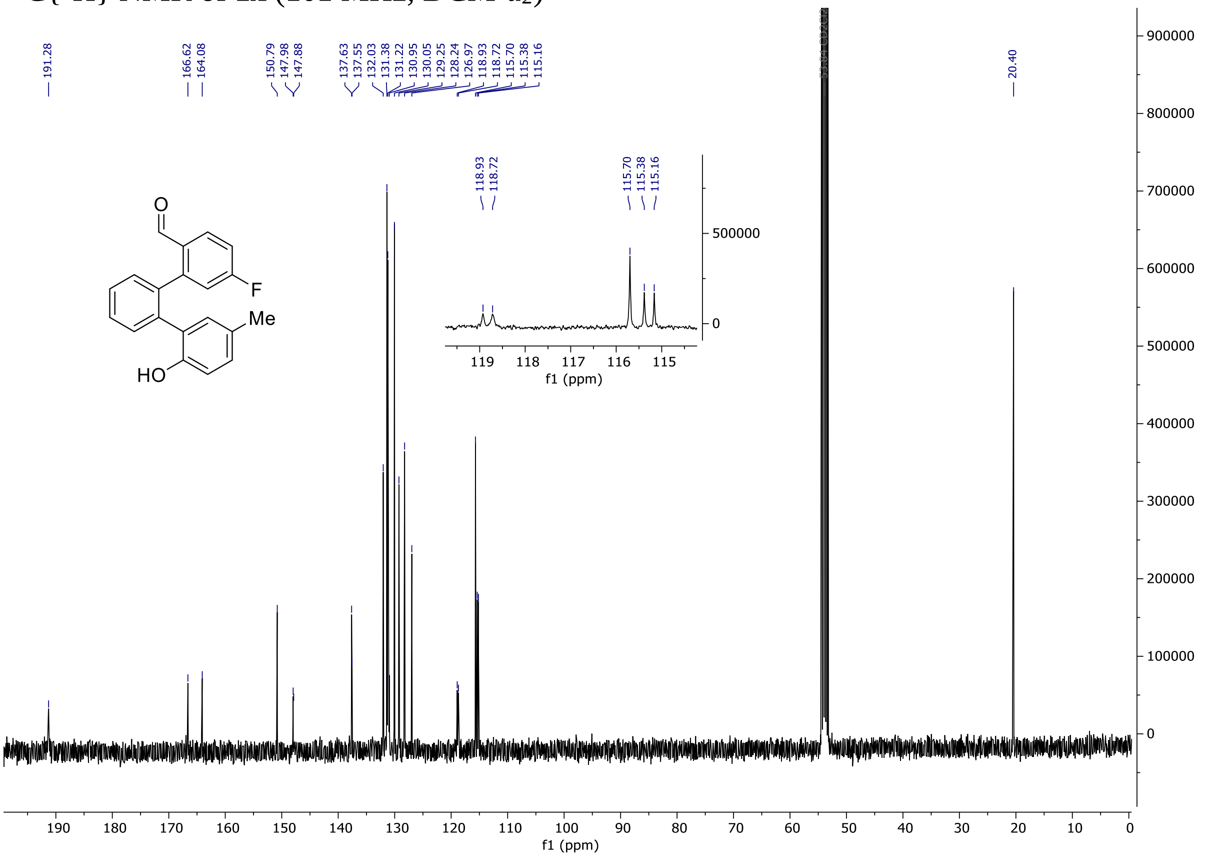

^{19}F NMR of **1x** (376 MHz, DCM-d_2)

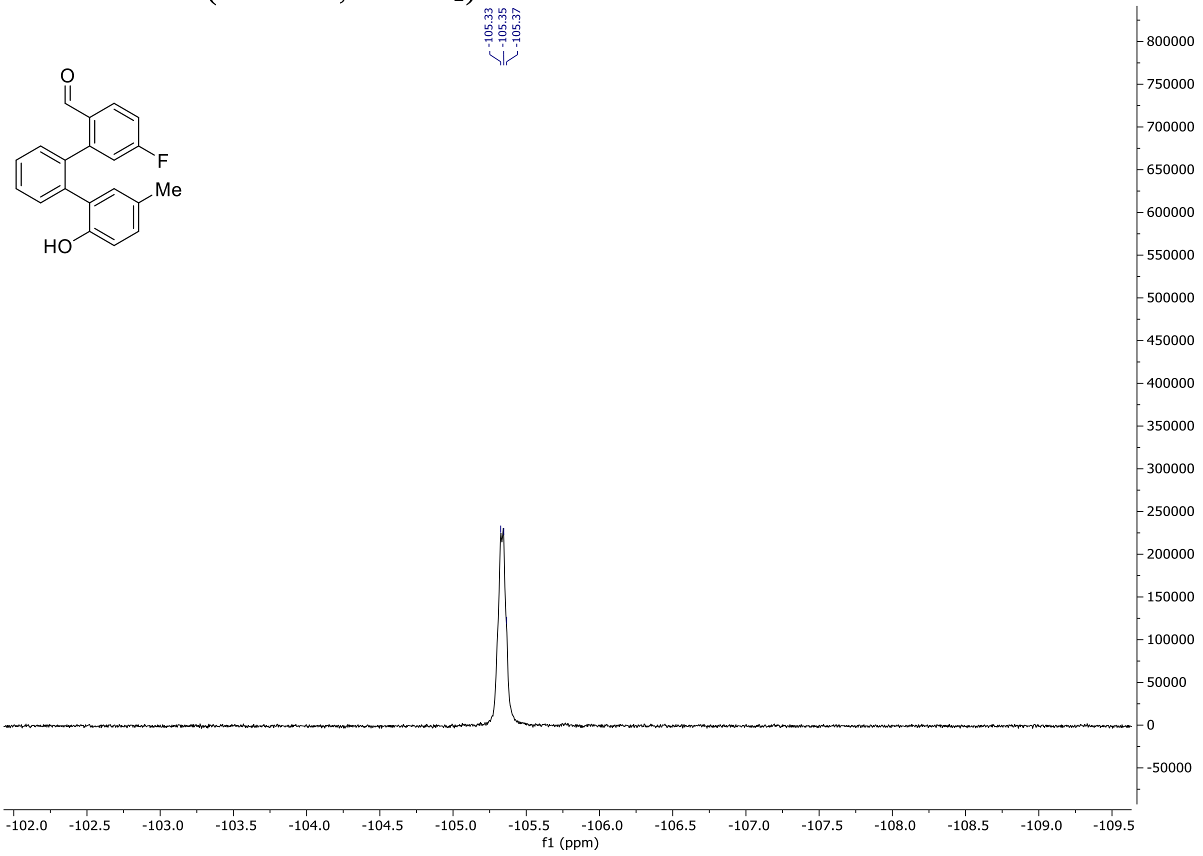

10*H*-Tribenzo[*b*,*d*,*f*]oxocin-10-one (2a)

^{1}H NMR of **2a** (400 MHz, DCM-*d*$_2$)

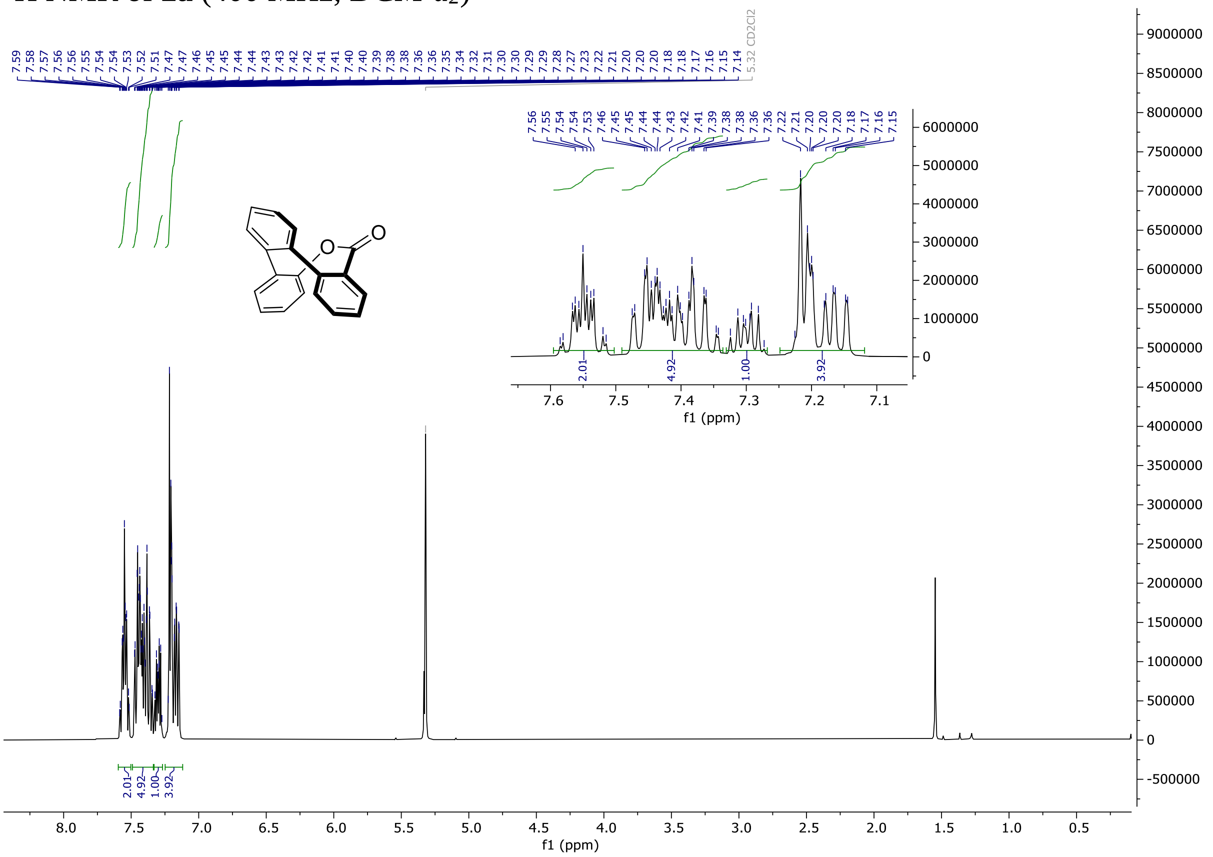

^{13}C{^{1}H} NMR of **2a** (101 MHz, DCM-*d*$_2$)

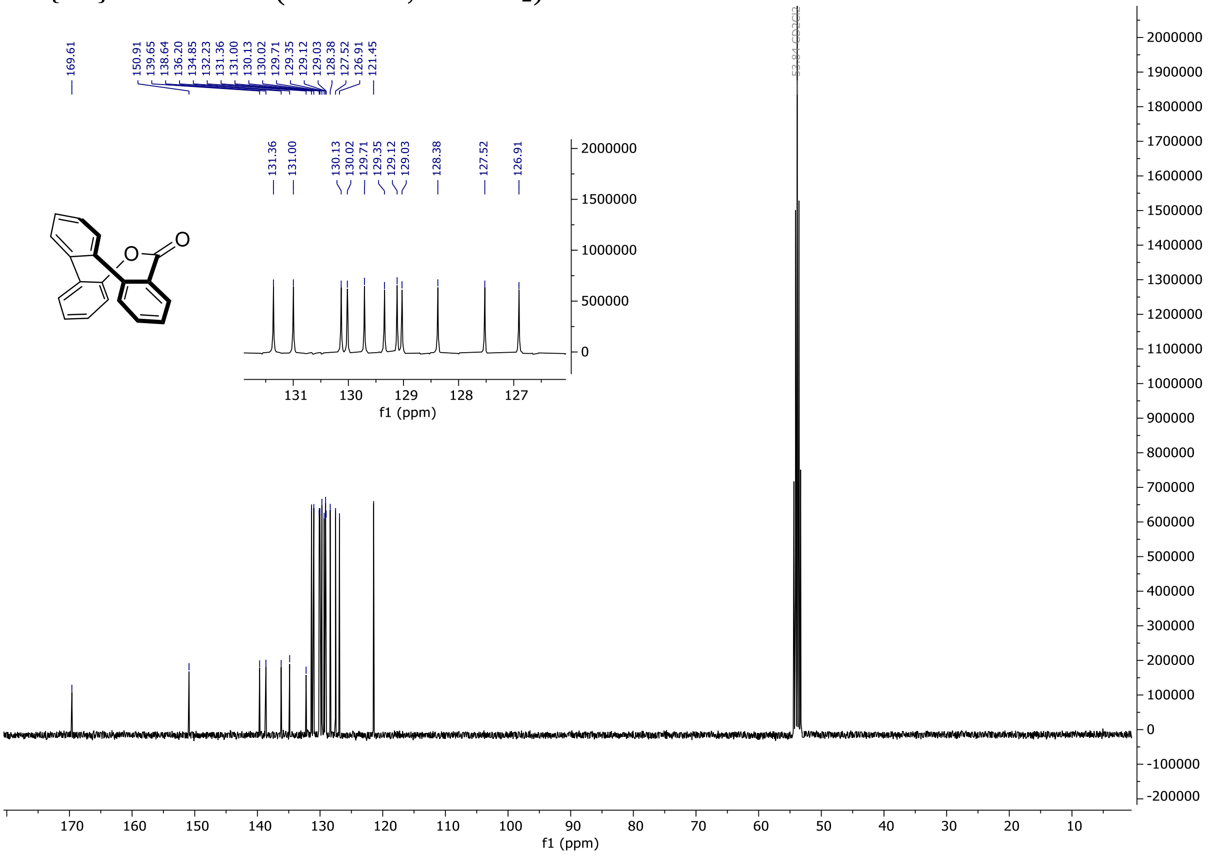

13-Methyl-10*H*-tribenzo[*b*,*d*,*f*]oxocin-10-one (2b)

^{1}H NMR of **2b** (400 MHz, DCM-d_2)

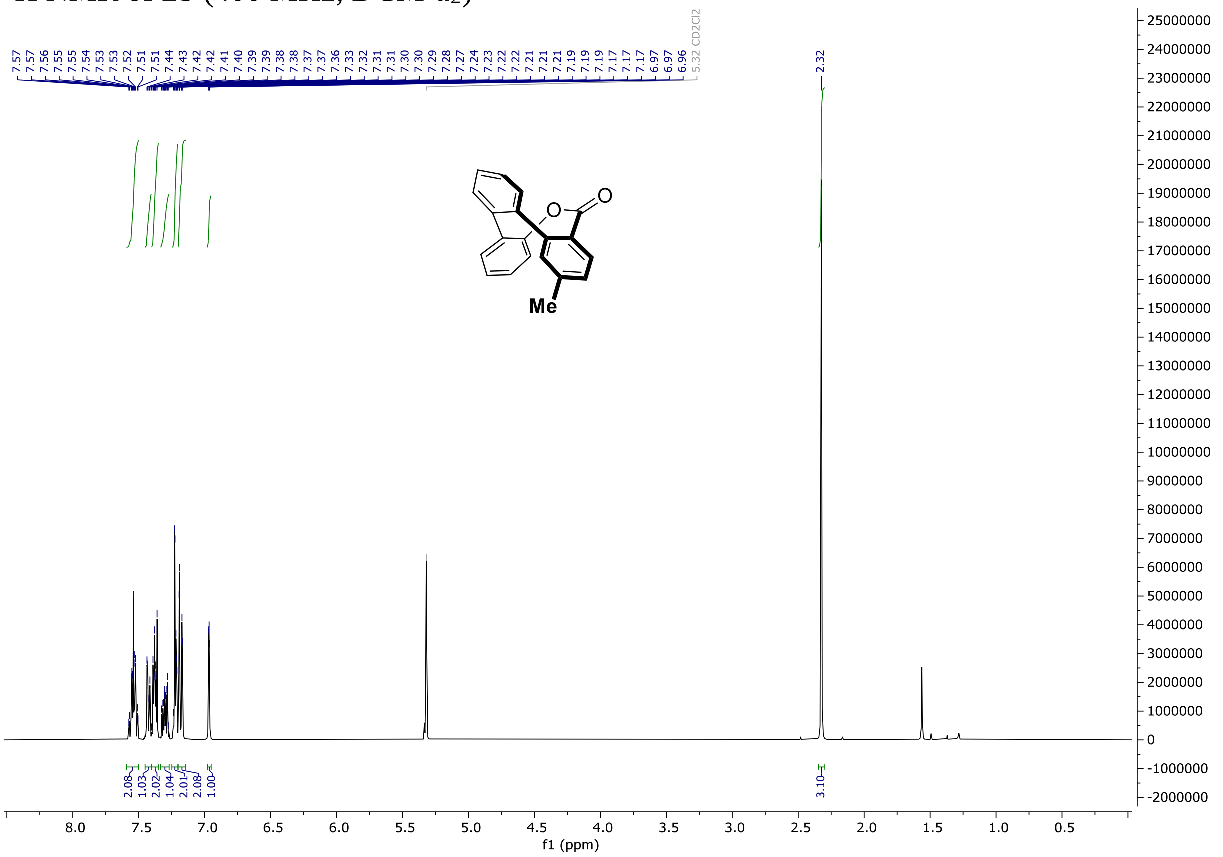

^{13}C{^{1}H} NMR of **2b** (101 MHz, DCM-d_2)

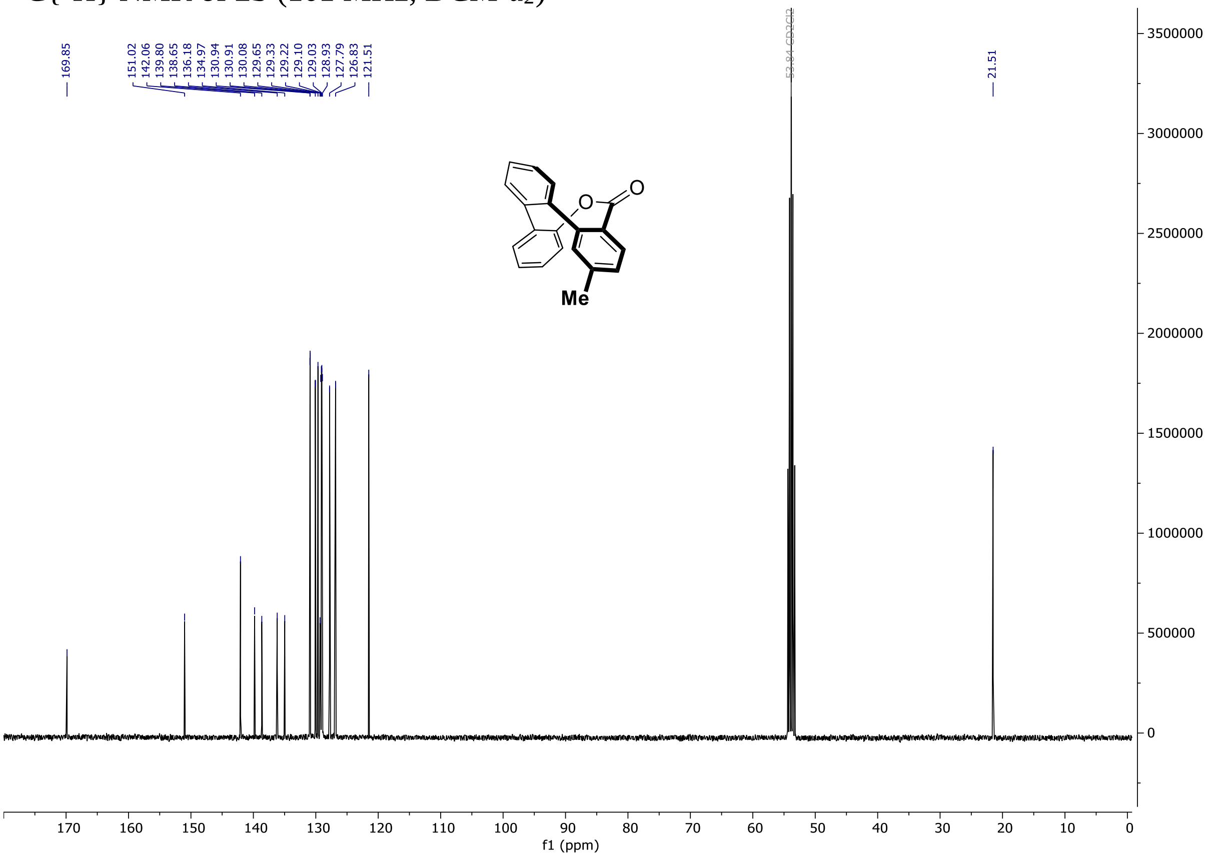

11-Methyl-10*H*-tribenzo[*b*,*d*,*f*]oxocin-10-one (2c)

^{1}H NMR of **2c** (400 MHz, $CDCl_3$)

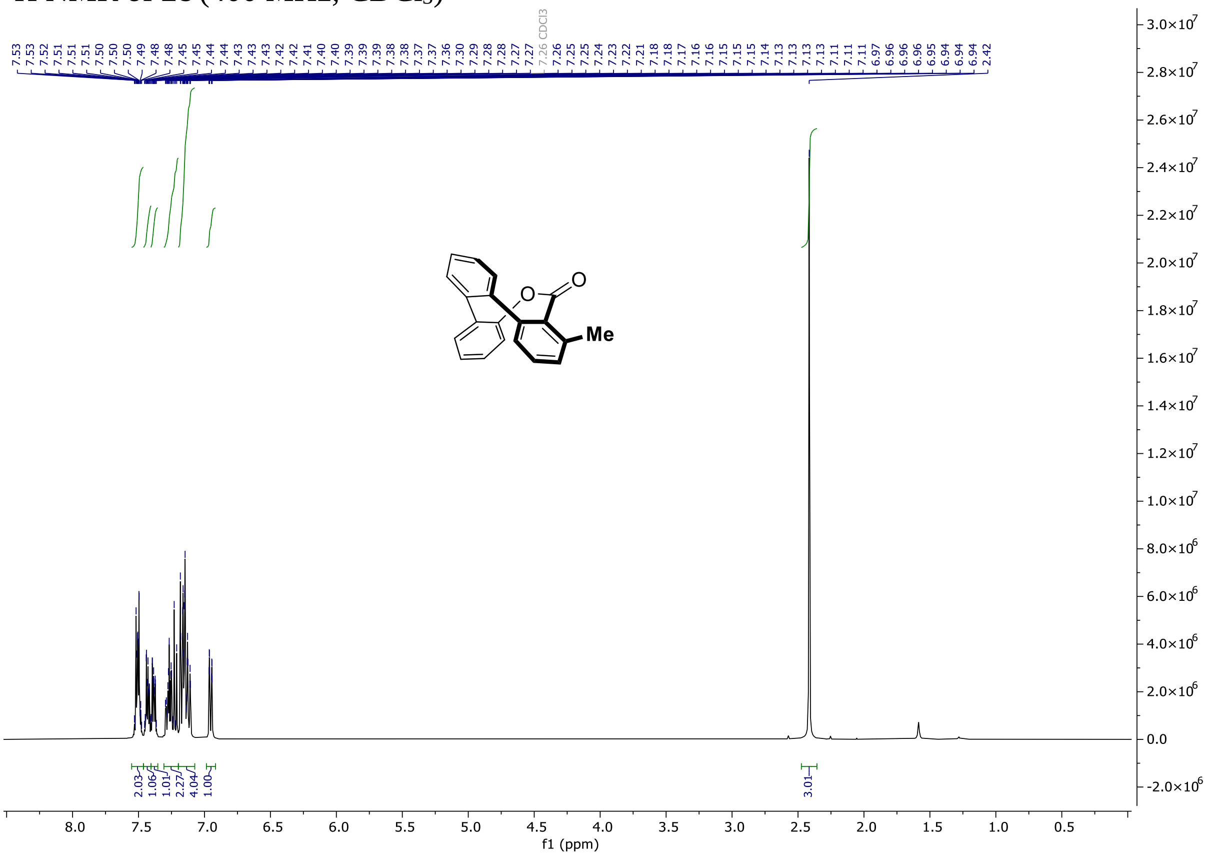

^{13}C{^{1}H} NMR of **2c** (101 MHz, chloroform-*d*)

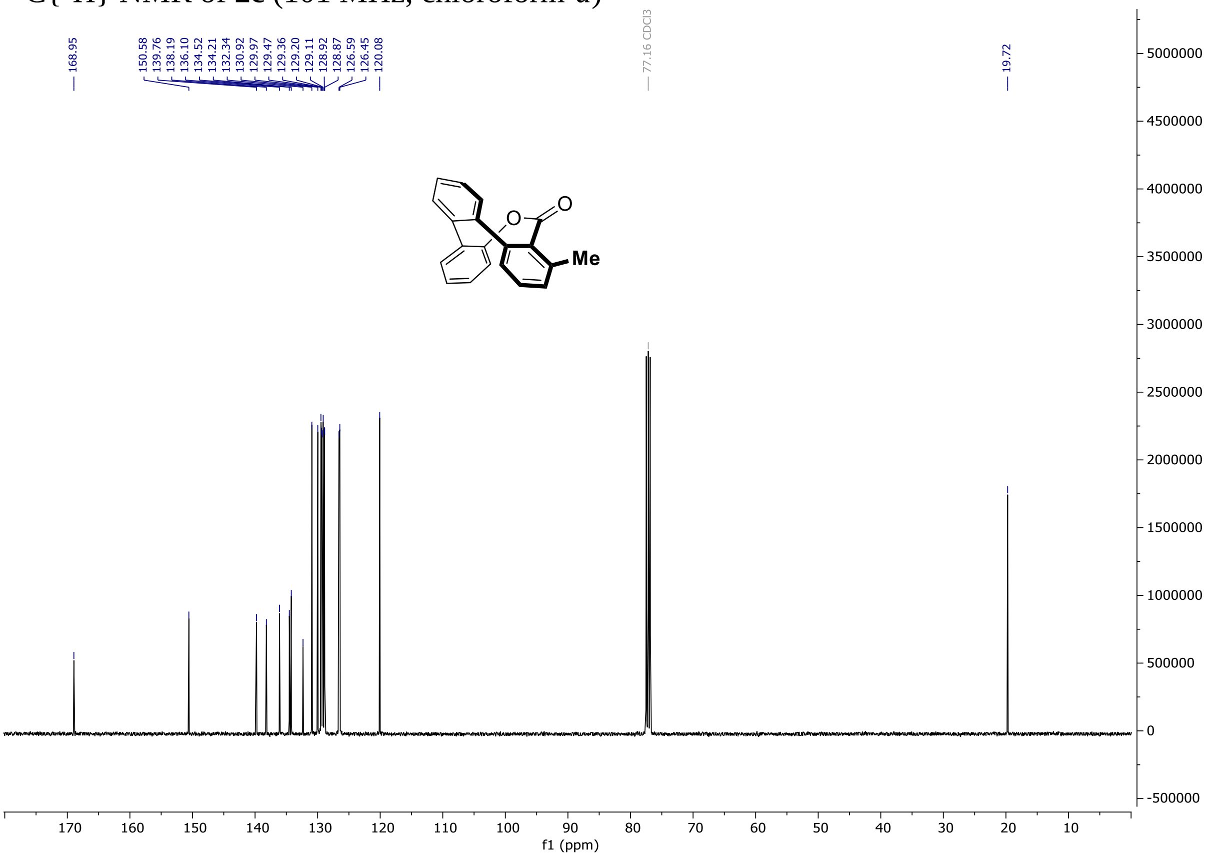

12-Methyl-10*H*-tribenzo[*b*,*d*,*f*]oxocin-10-one (2d)

^{1}H NMR of **2d** (400 MHz, $CDCl_3$)

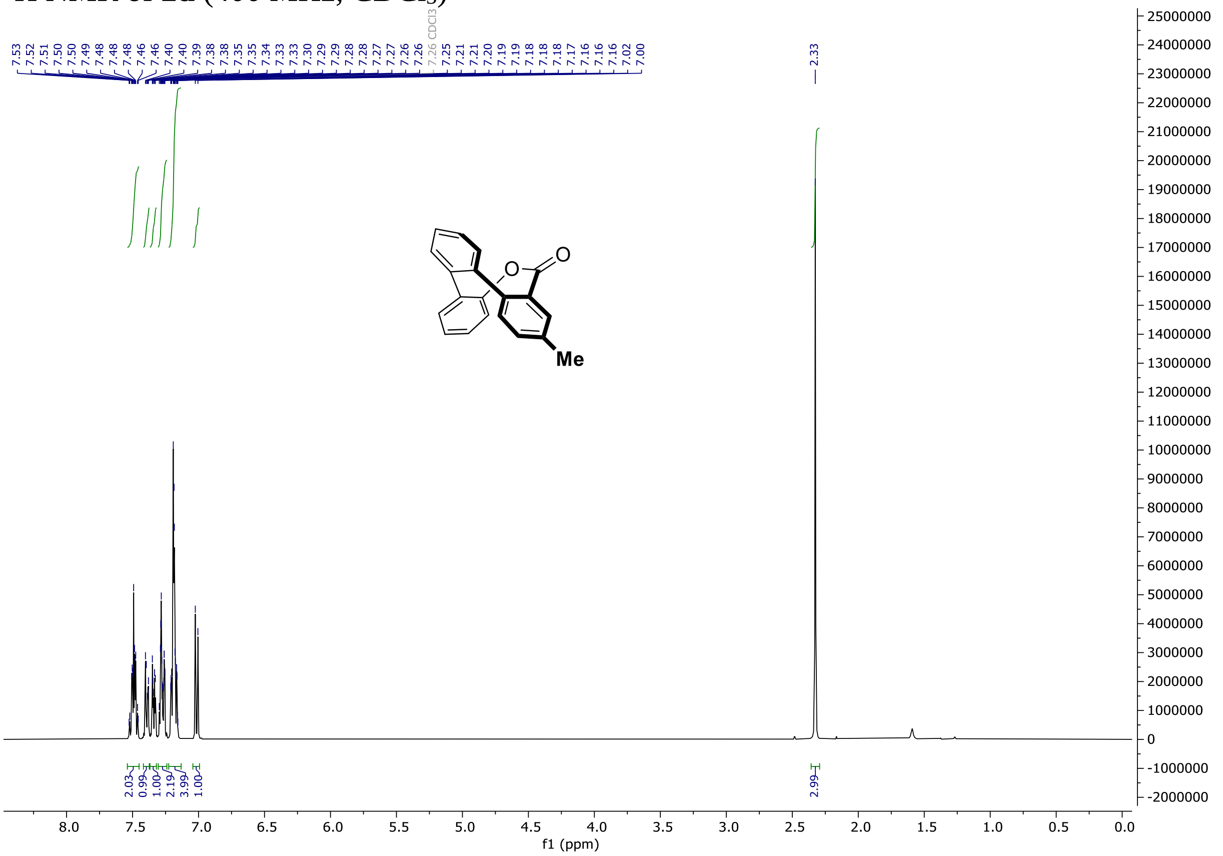

^{13}C{^{1}H} NMR of **2d** (101 MHz, chloroform-*d*)

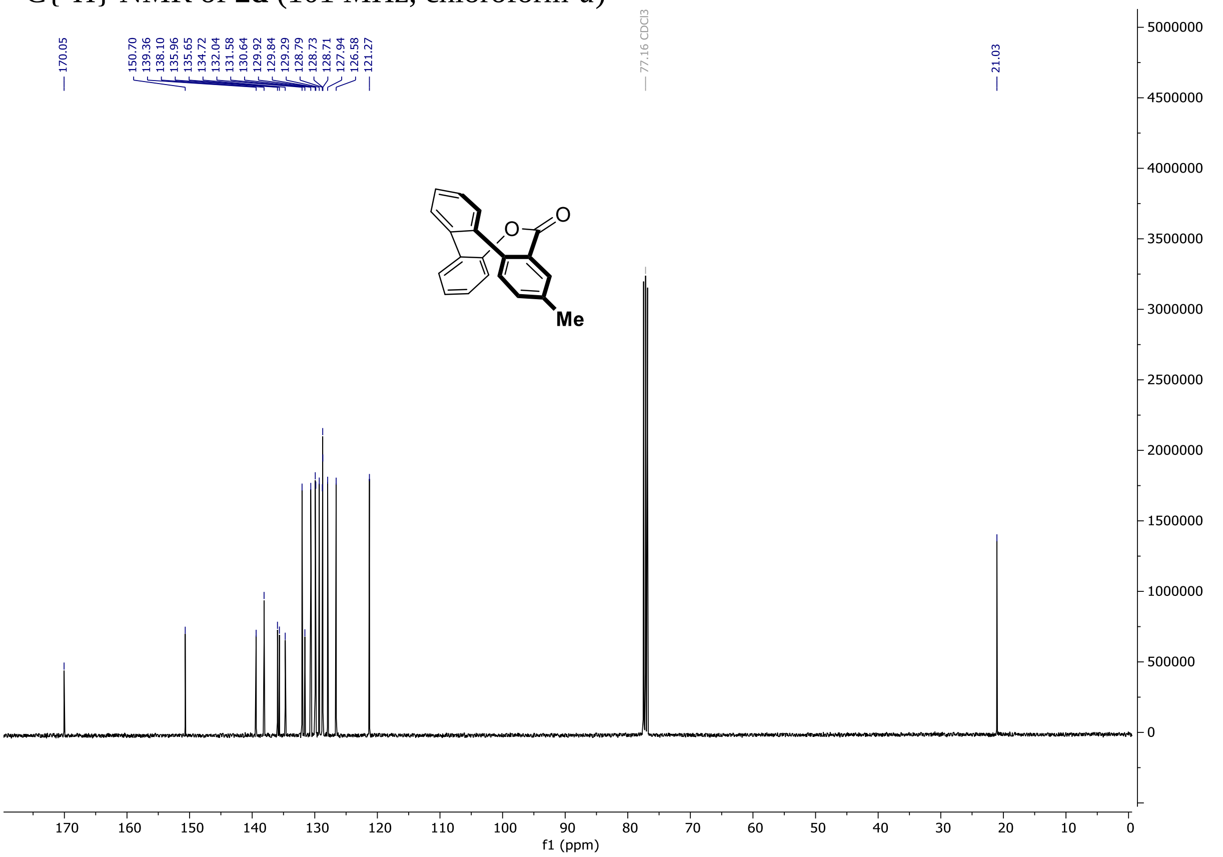

11-Methyl-10*H*-tribenzo[*b*,*d*,*f*]oxocin-10-one (2e)

^{1}H NMR of **2e** (400 MHz, DCM-d_2)

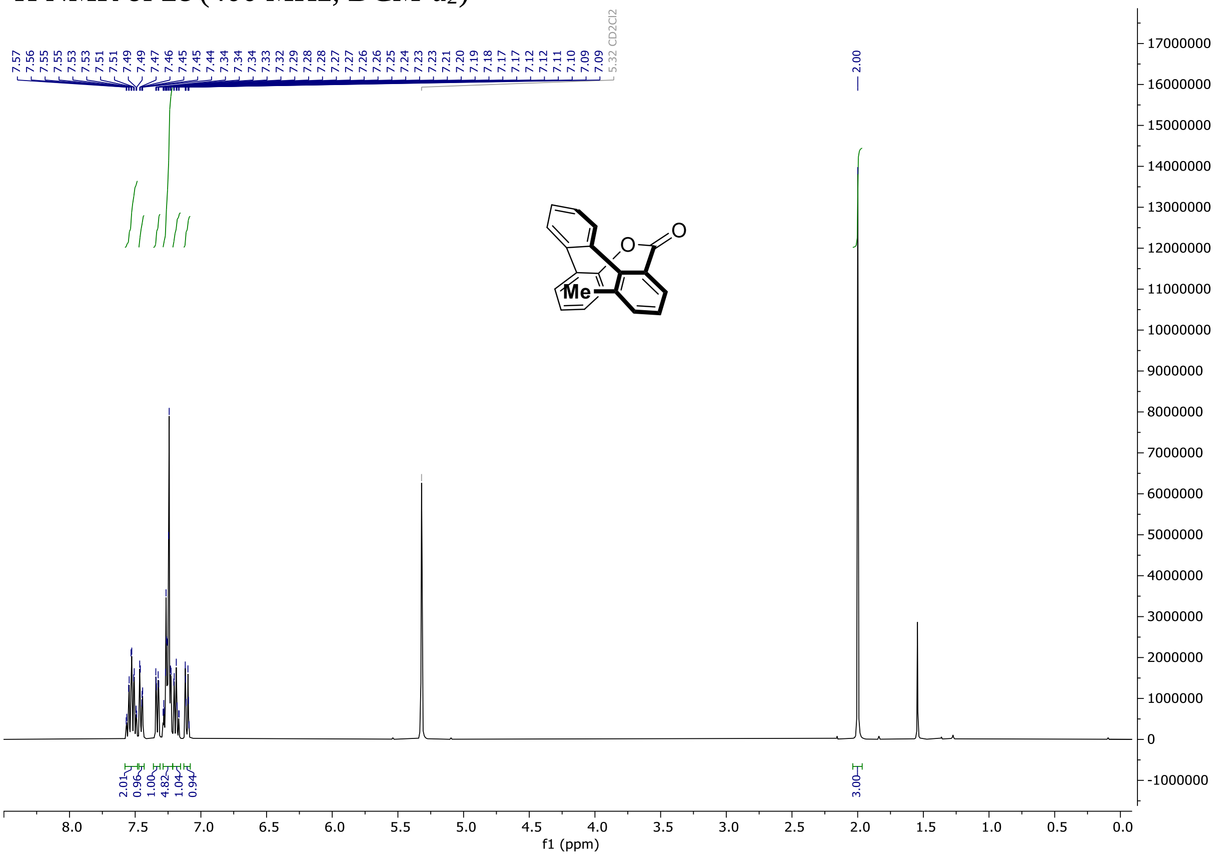

^{13}C{^{1}H} NMR of **2e** (101 MHz, DCM-d_2)

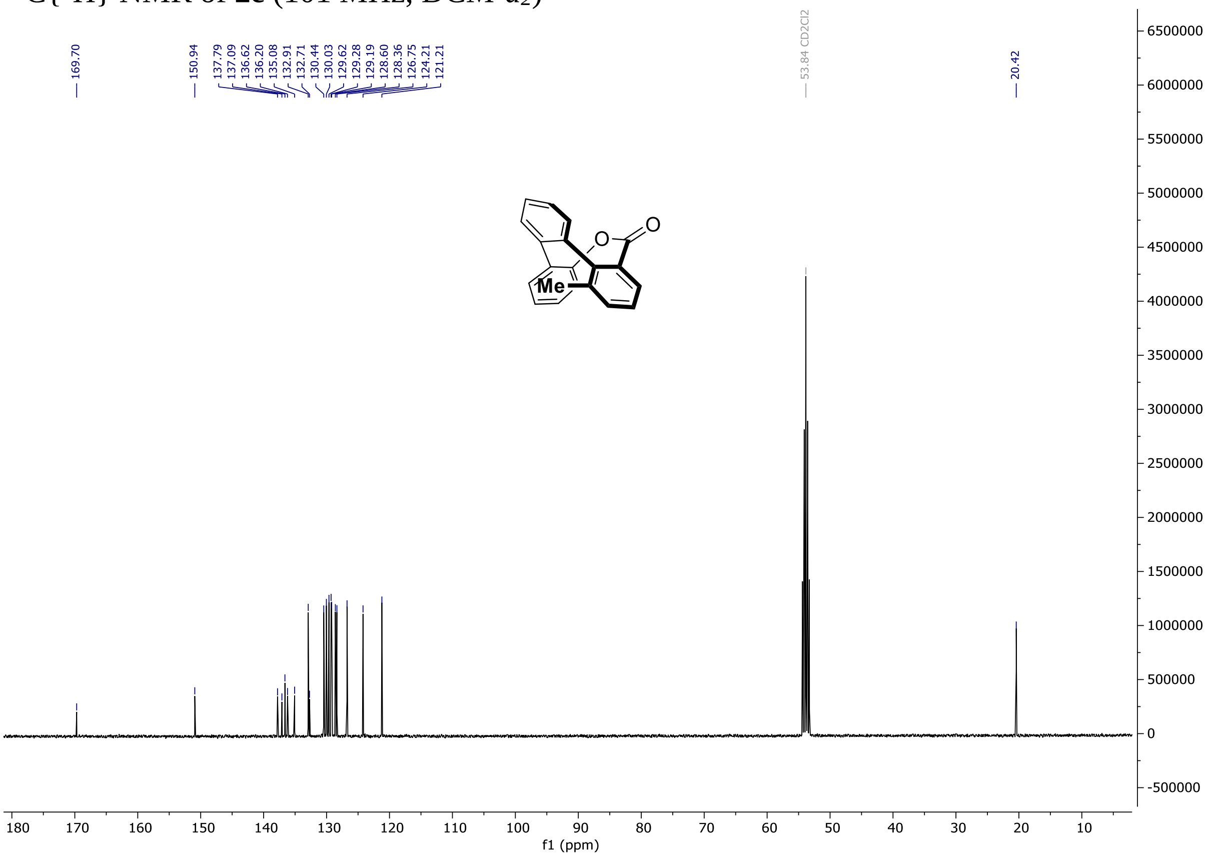

13-Methoxy-10*H*-tribenzo[*b*,*d*,*f*]oxocin-10-one (2f)

^{1}H NMR of **2c** (400 MHz, DCM-d_2)

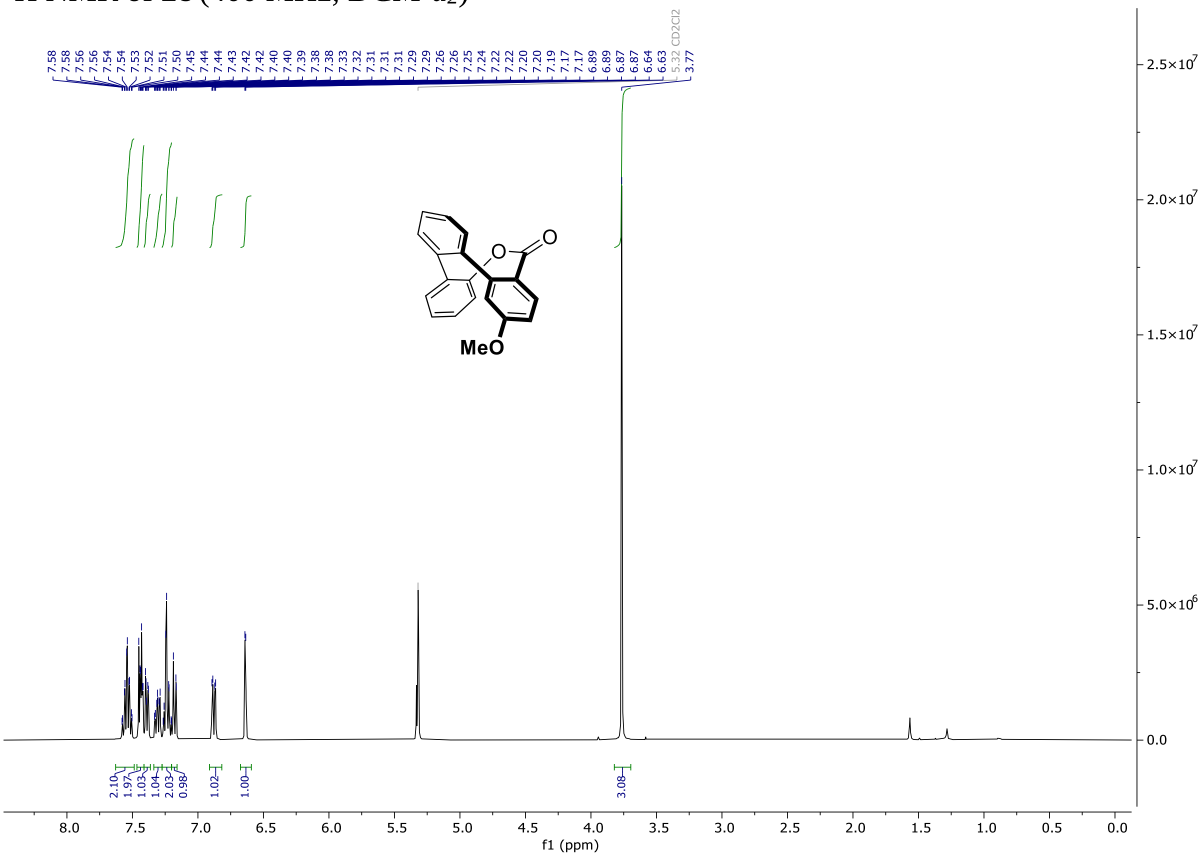

^{13}C{^{1}H} NMR of **2f** (101 MHz, DCM-d_2)

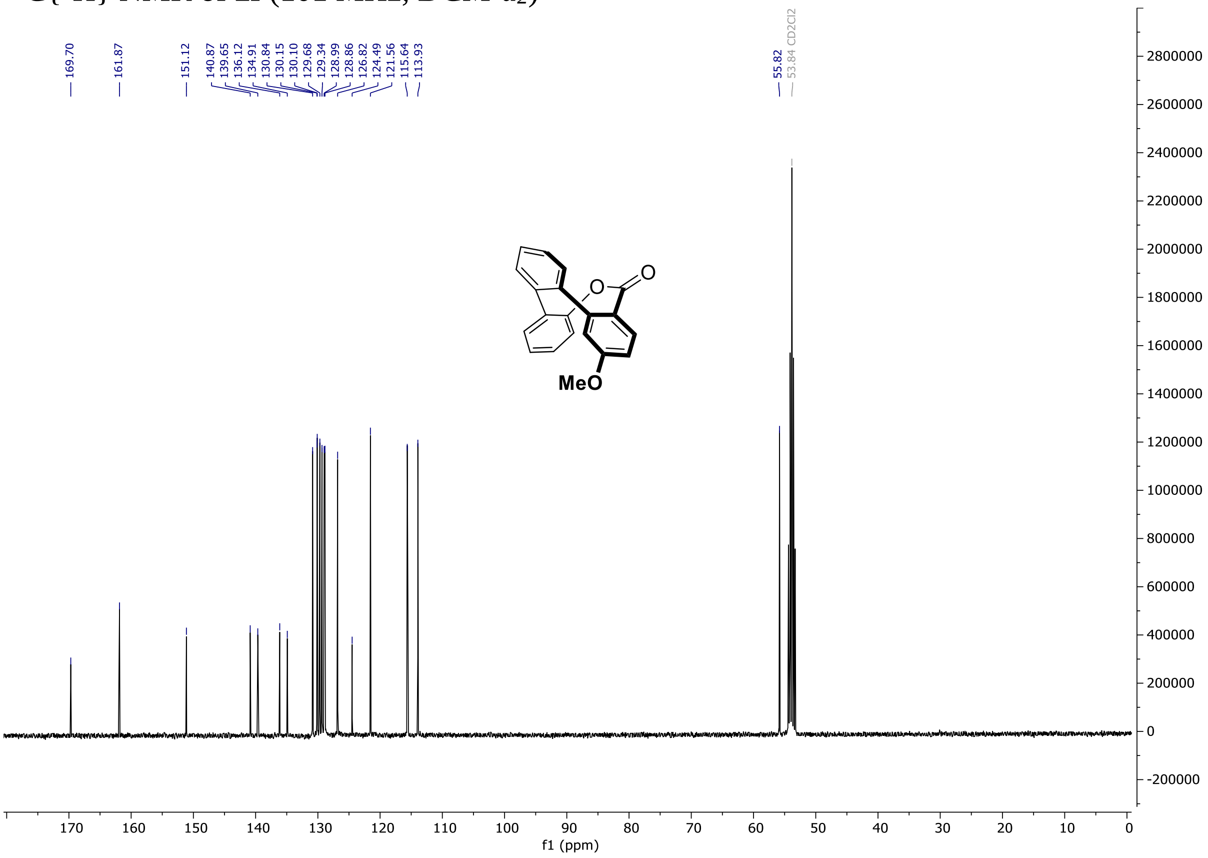

13-Fluoro-10*H*-tribenzo[*b,d,f*]oxocin-10-one (2g)

^{1}H NMR of **2g** (400 MHz, DCM-d_2)

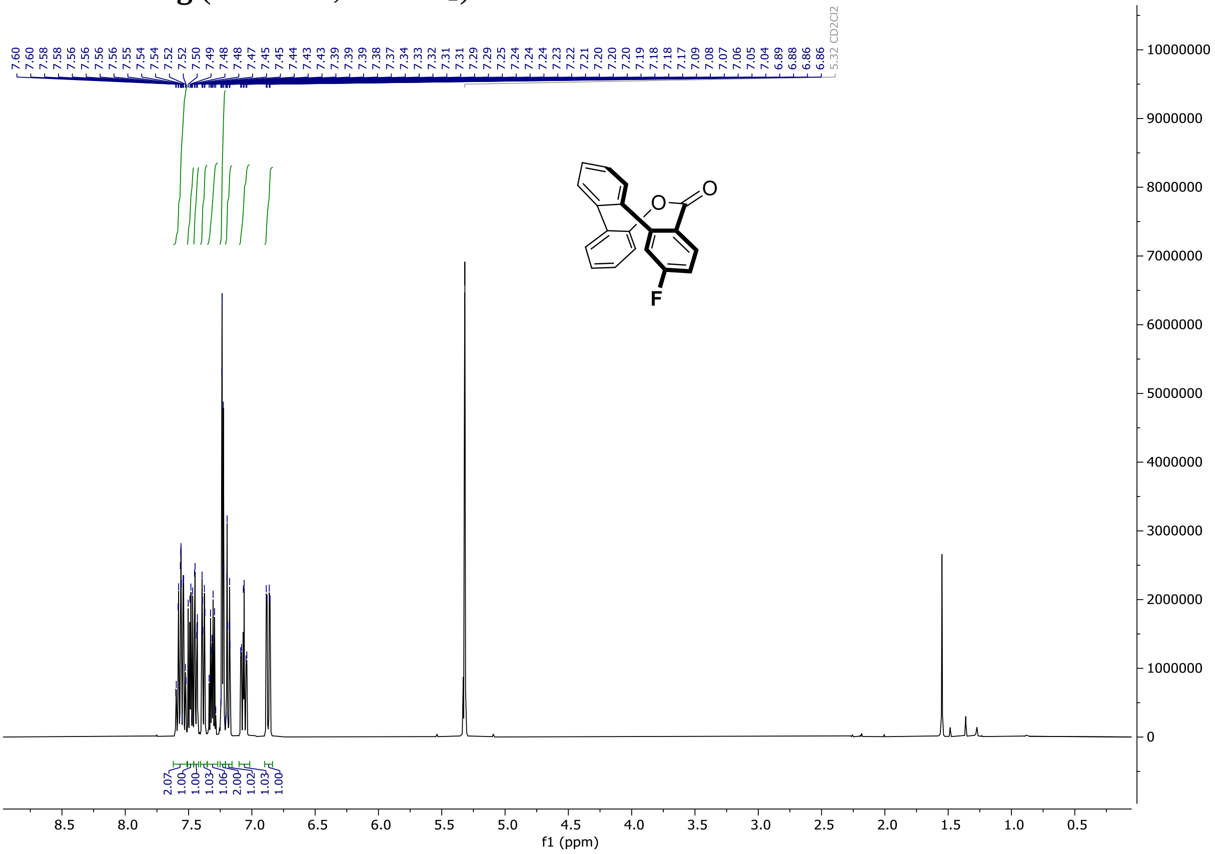

^{13}C{^{1}H} NMR of **2g** (101 MHz, DCM-d_2)

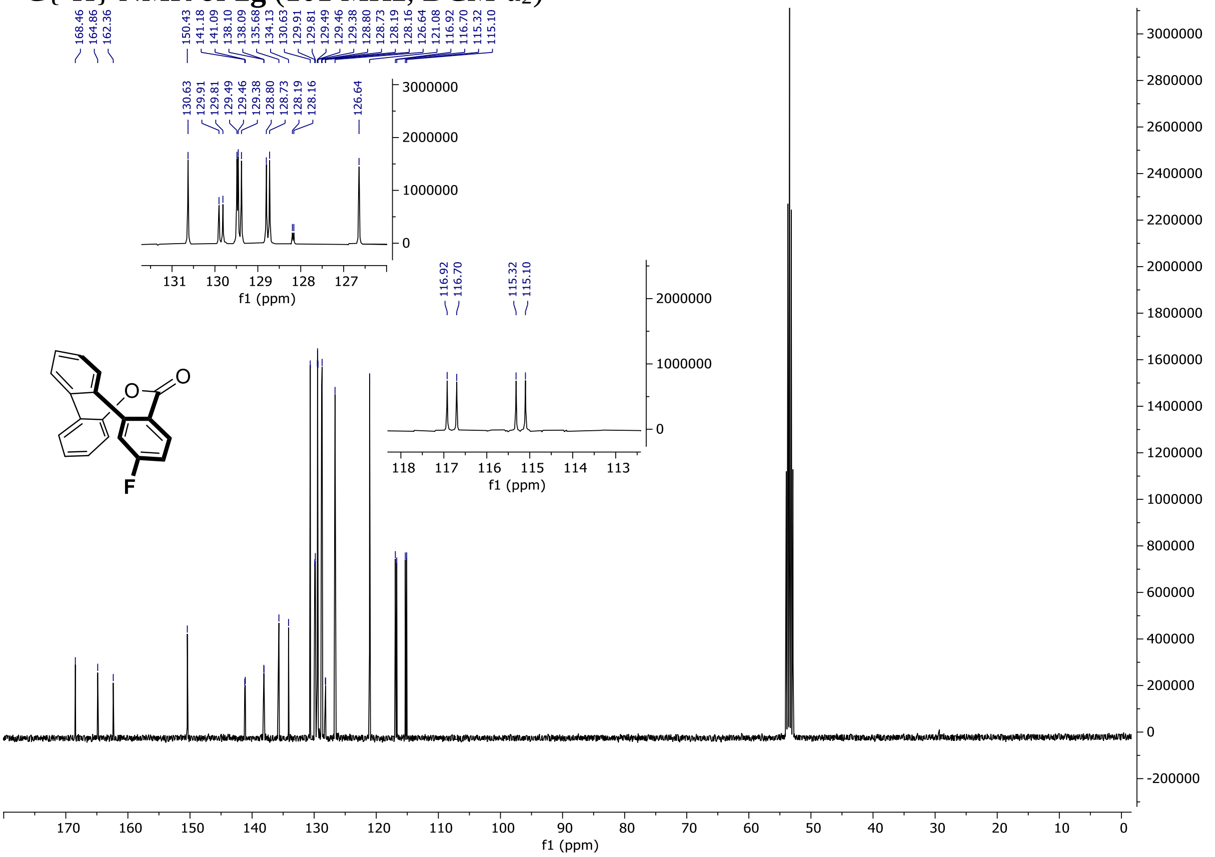

^{19}F NMR of **2g** (376 MHz, DCM-d_2)

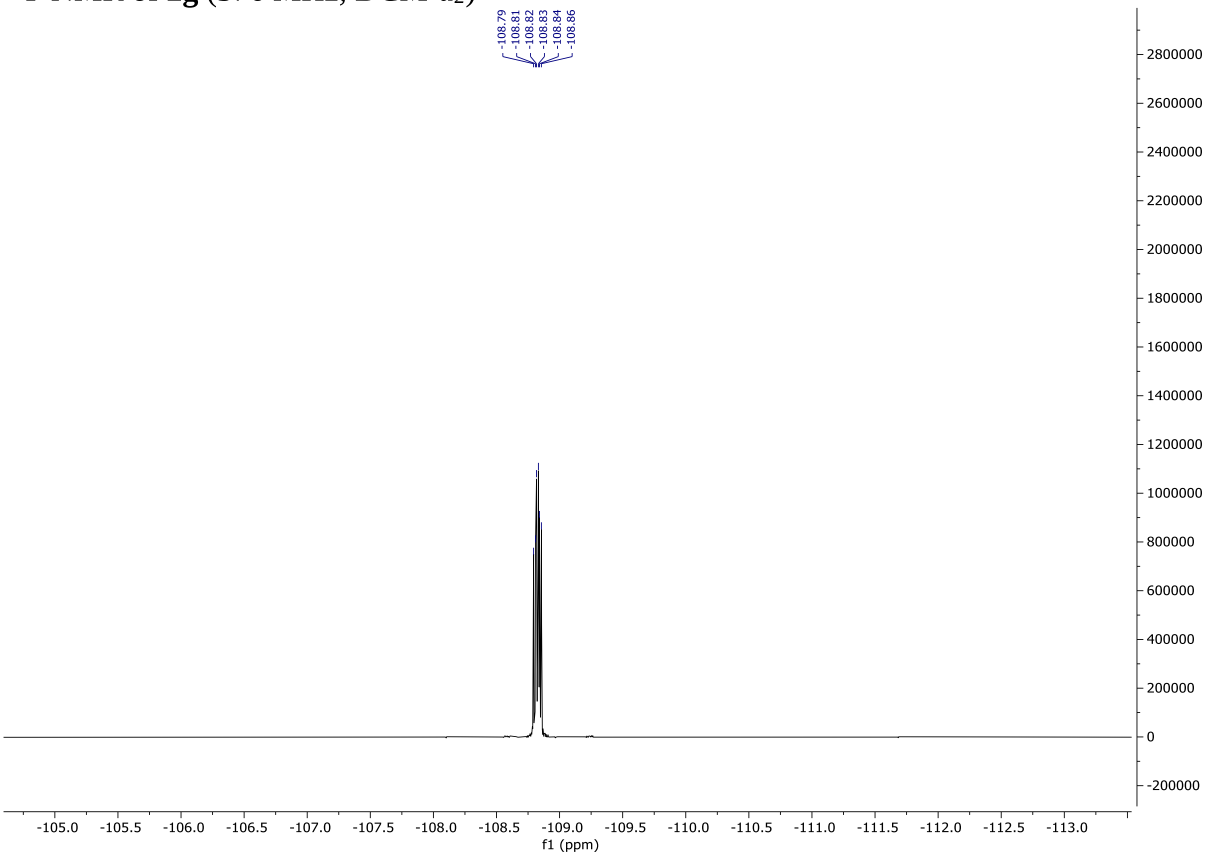

13-Chloro-10*H*-tribenzo[*b*,*d*,*f*]oxocin-10-one (2h)

^{1}H NMR of **2h** (400 MHz, DCM-d_2)

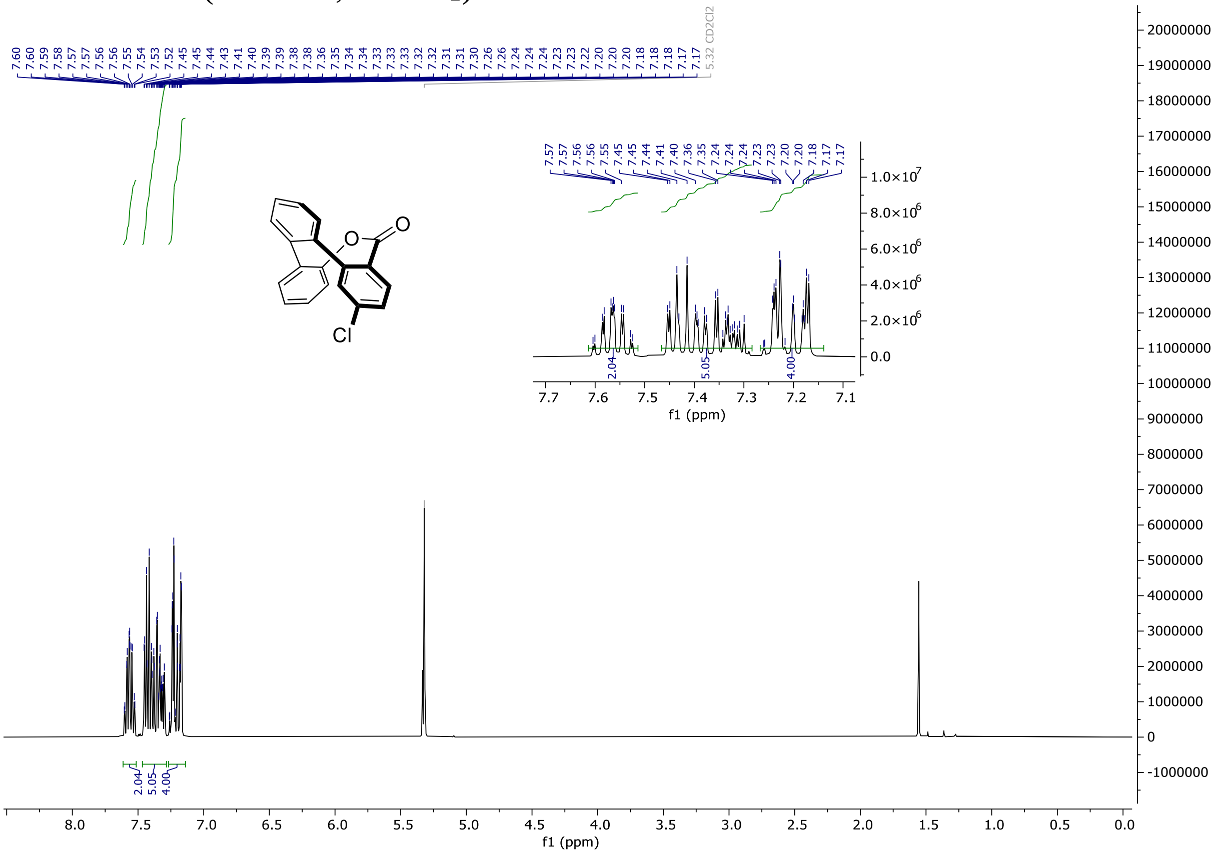

^{13}C{^{1}H} NMR of **2h** (101 MHz, DCM-d_2)

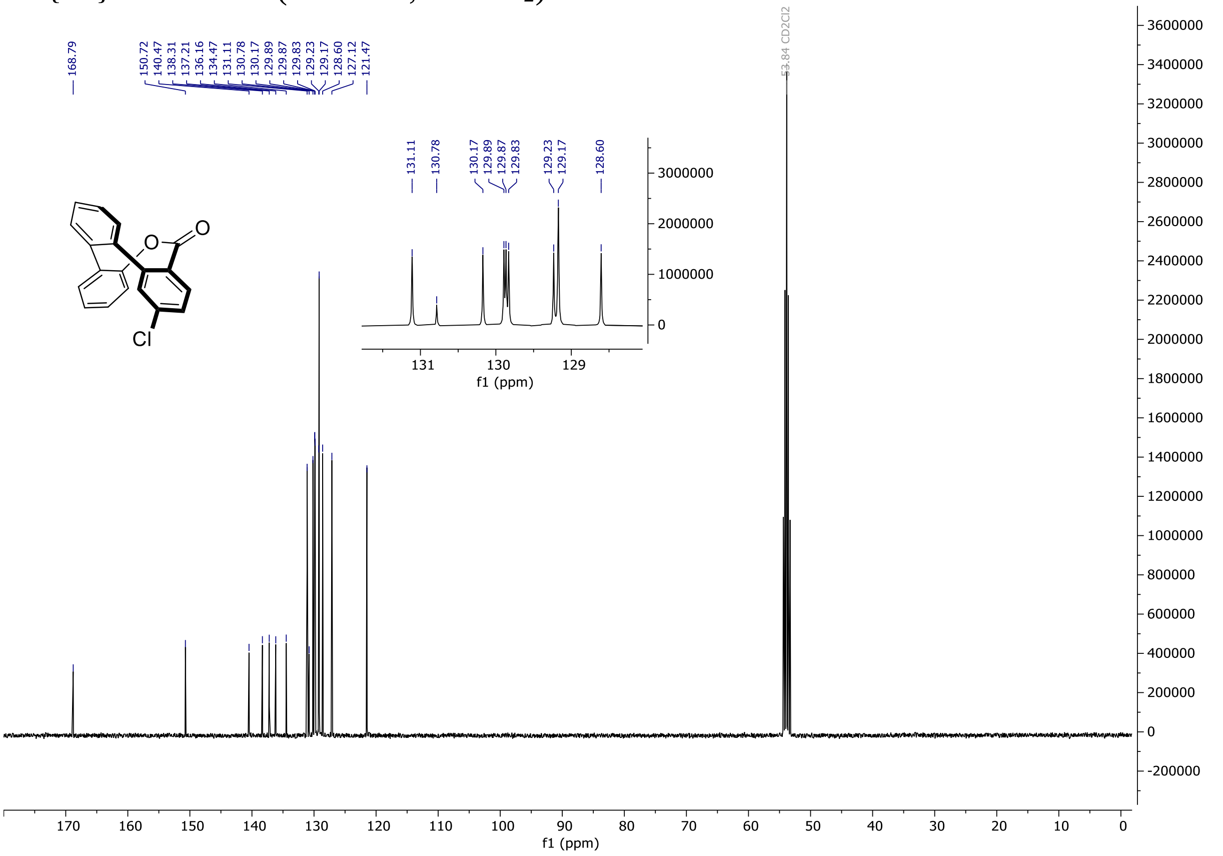

13-Bromo-10*H*-tribenzo[*b*,*d*,*f*]oxocin-10-one (2i)

^{1}H NMR of **2f** (400 MHz, DCM-d_2)

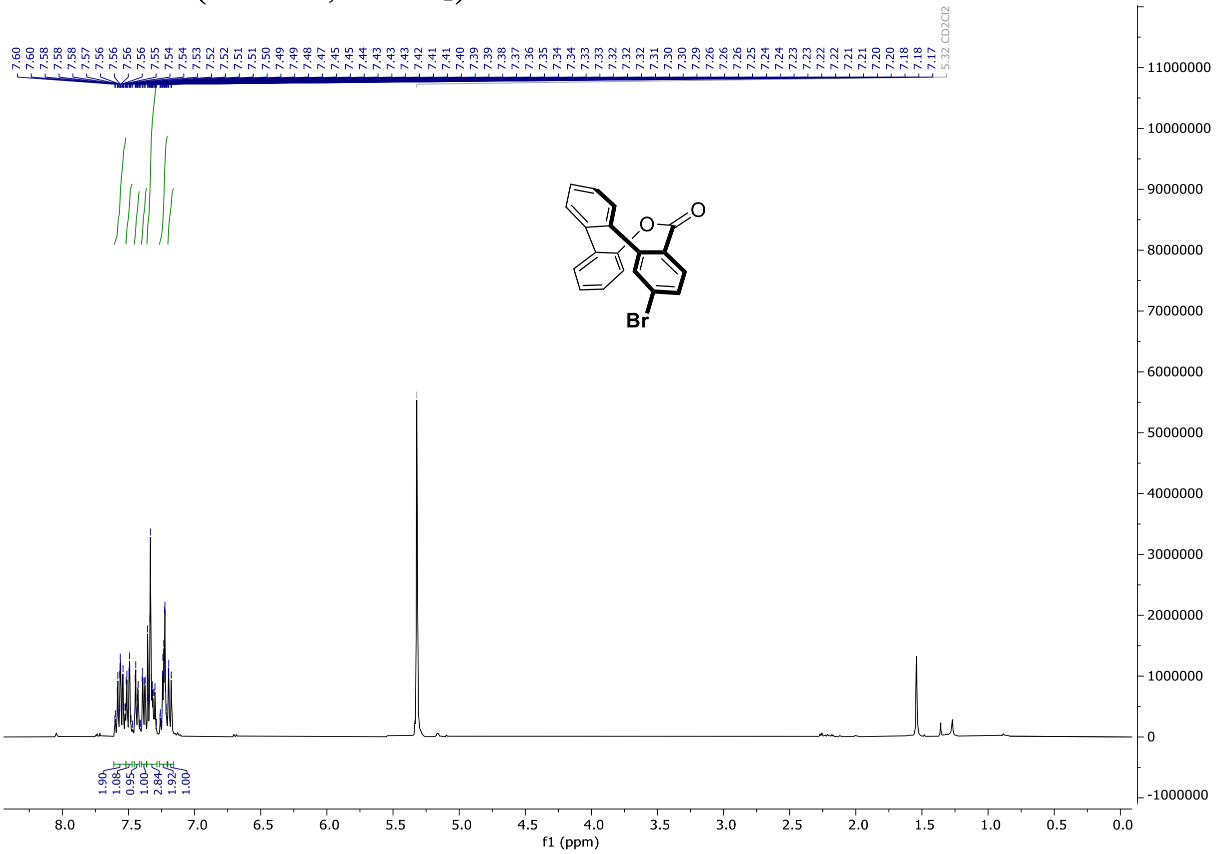

^{13}C{^{1}H} NMR of **2i** (101 MHz, DCM-d_2)

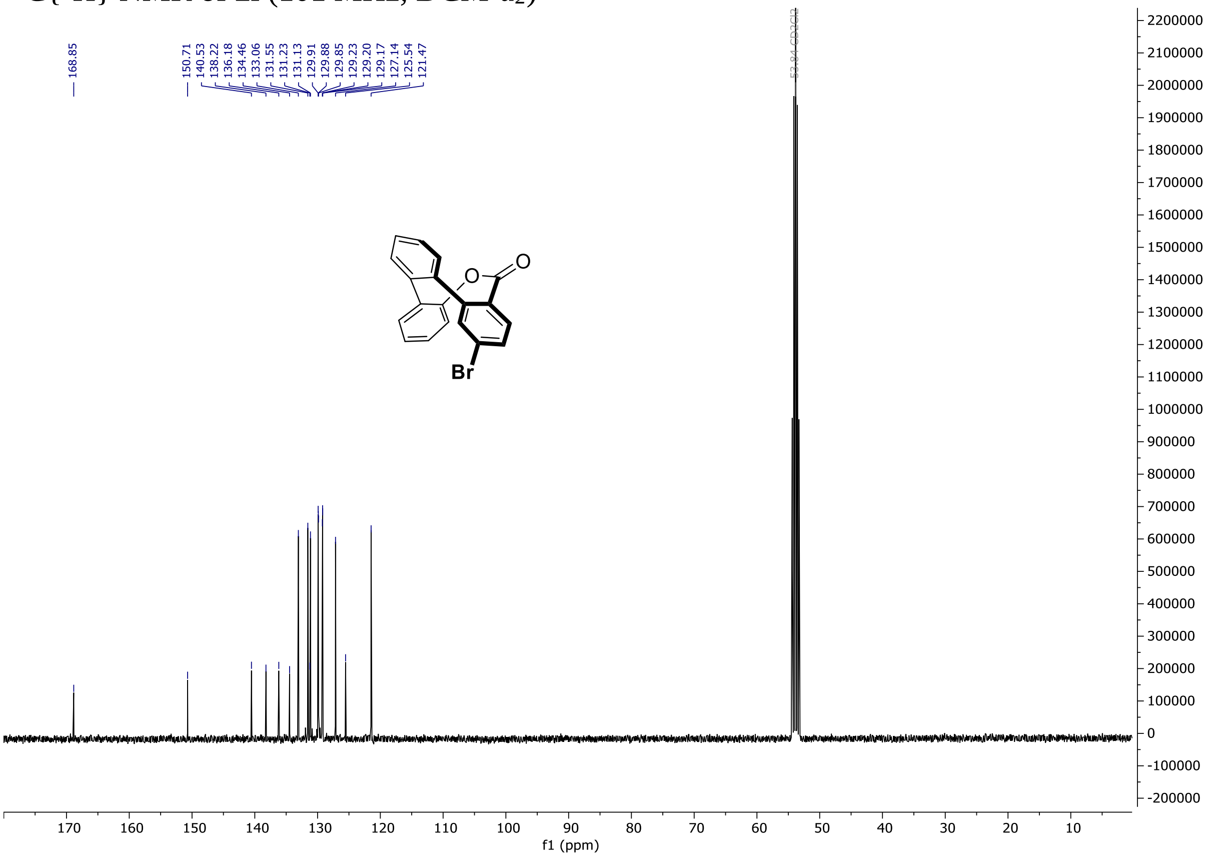

10-Oxo-10*H*-tribenzo[*b*,*d*,*f*]oxocine-13-carbonitrile (2j)

^{1}H NMR of **2j** (400 MHz, DCM-d_2)

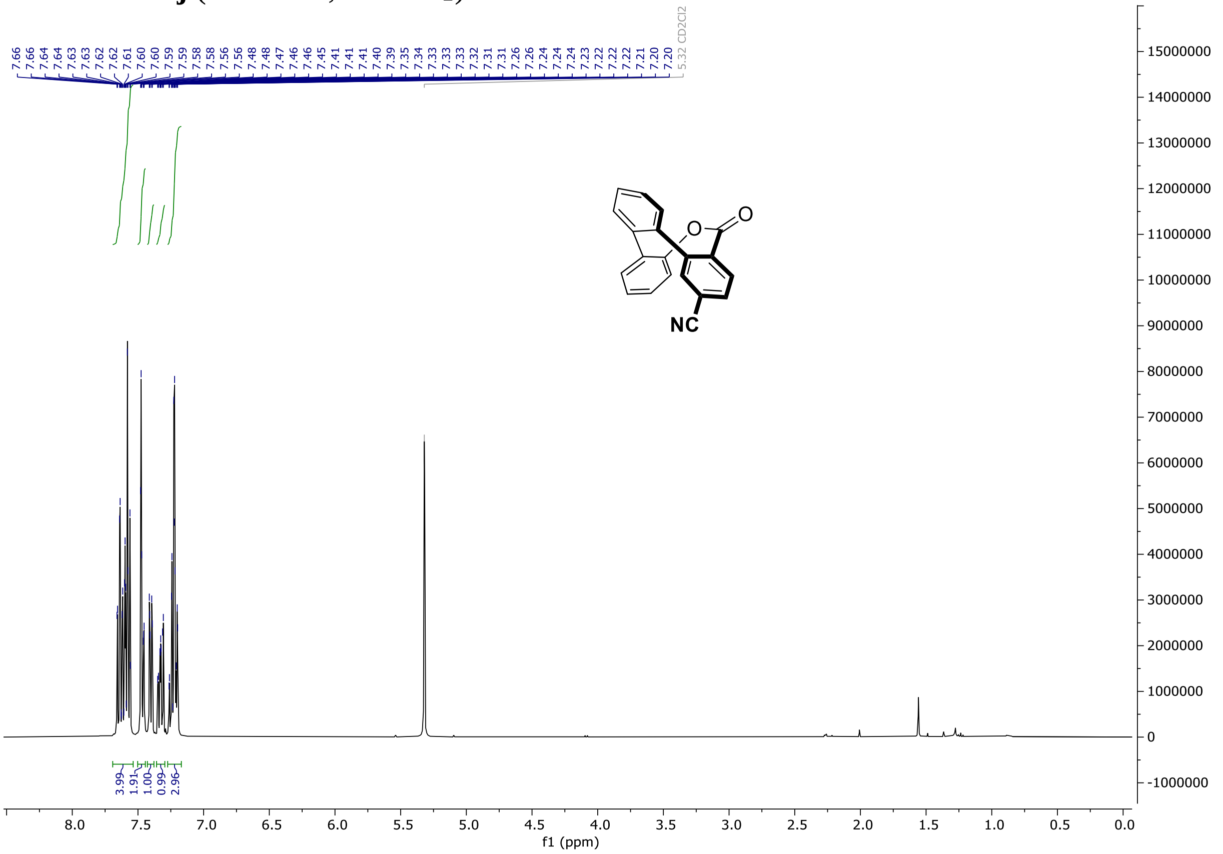

^{13}C{^{1}H} NMR of **2j** (101 MHz, DCM-d_2)

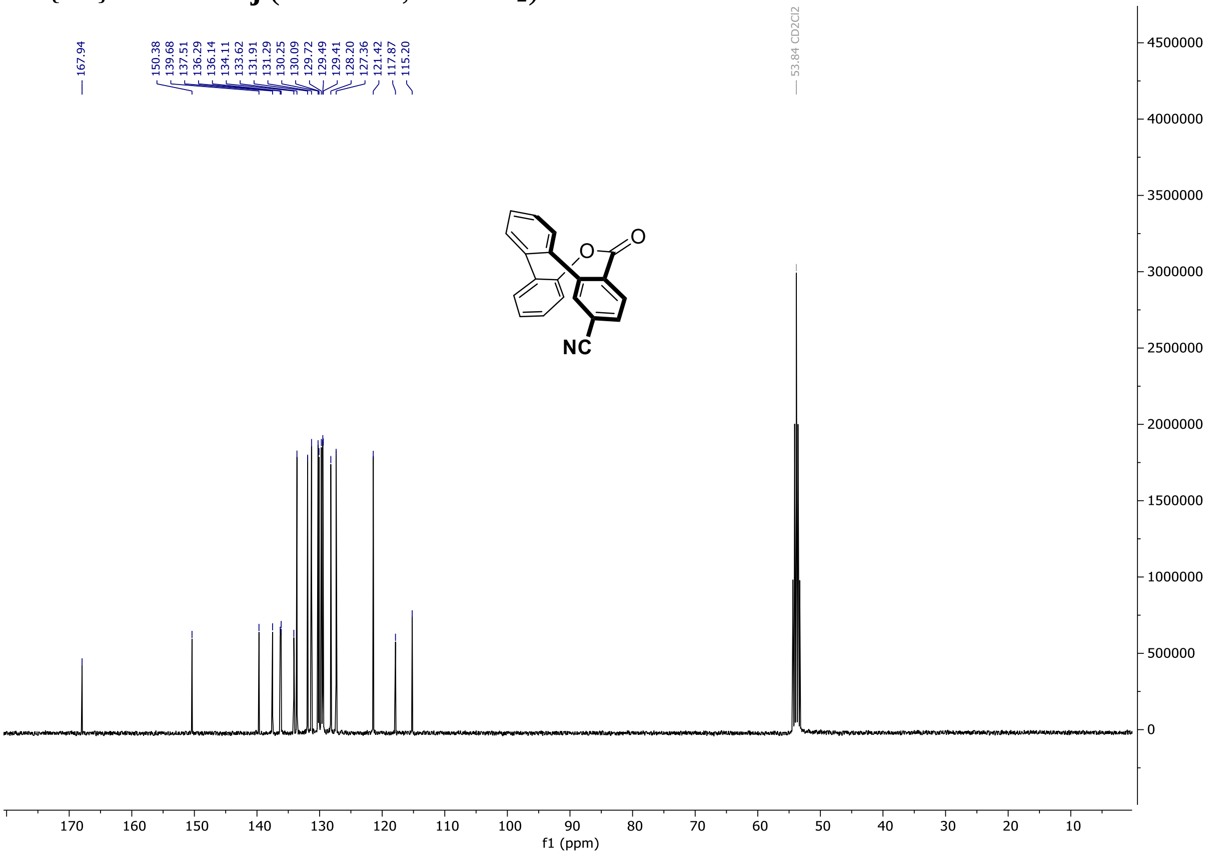

13-(Trifluoromethyl)-10*H*-tribenzo[*b*,*d*,*f*]oxocin-10-one (2k)

^{1}H NMR of **2k** (400 MHz, DCM-d_2)

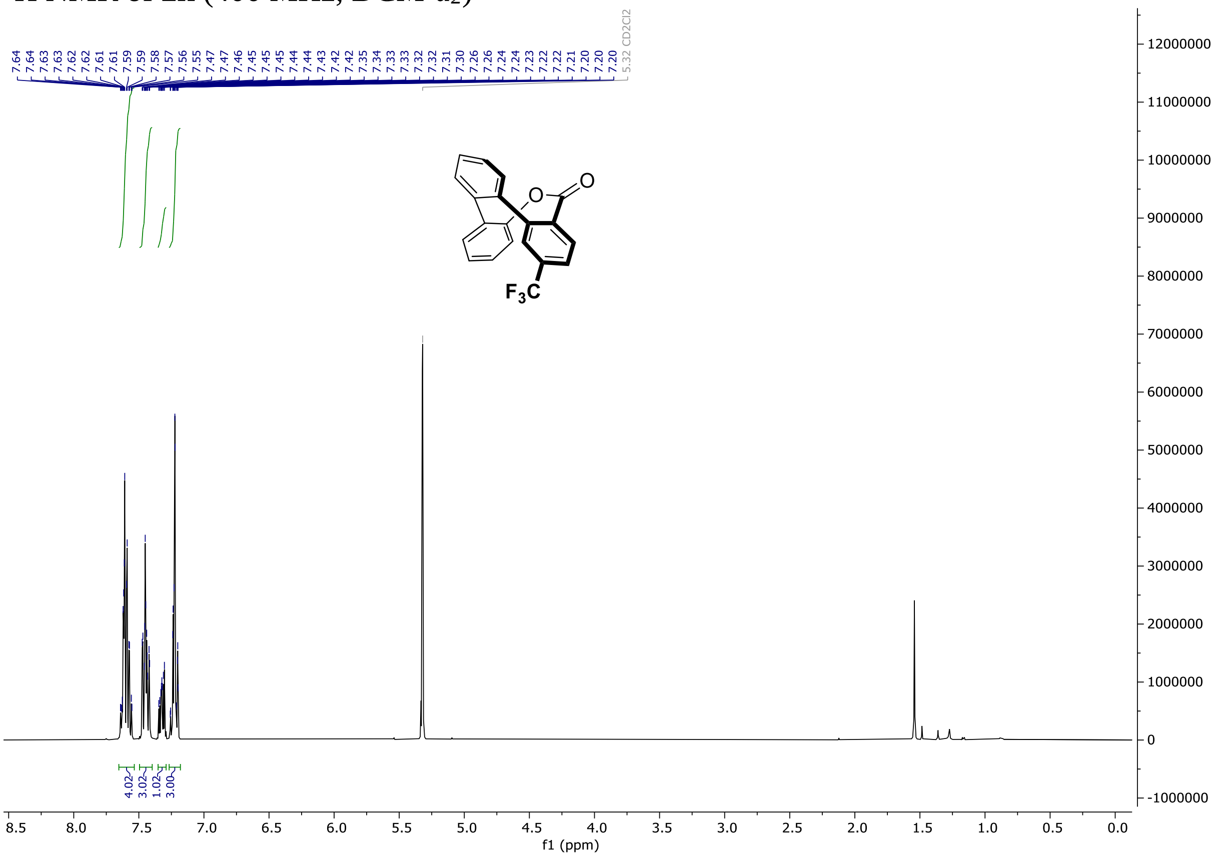

^{13}C{^{1}H} NMR of **2k** (101 MHz, DCM-d_2)

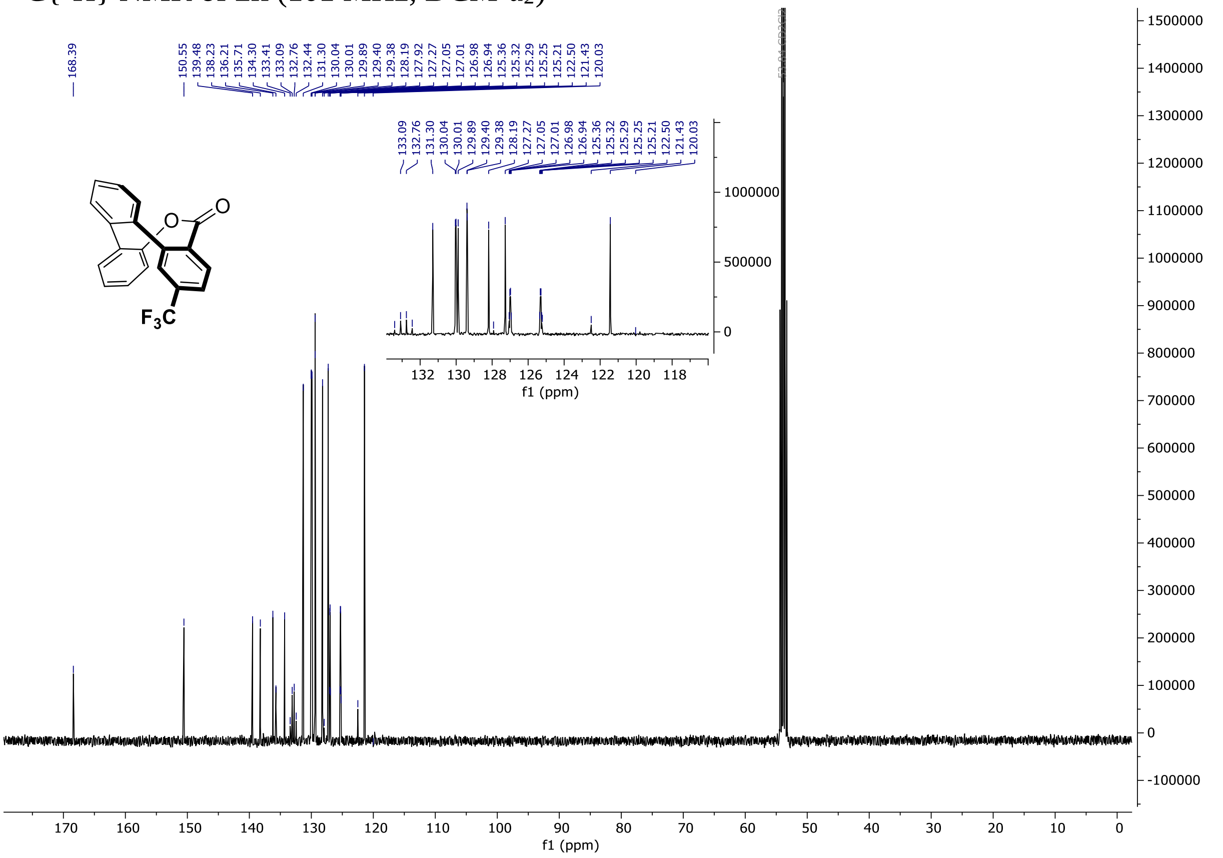

^{19}F NMR of **2k** (376 MHz, DCM-d_2)

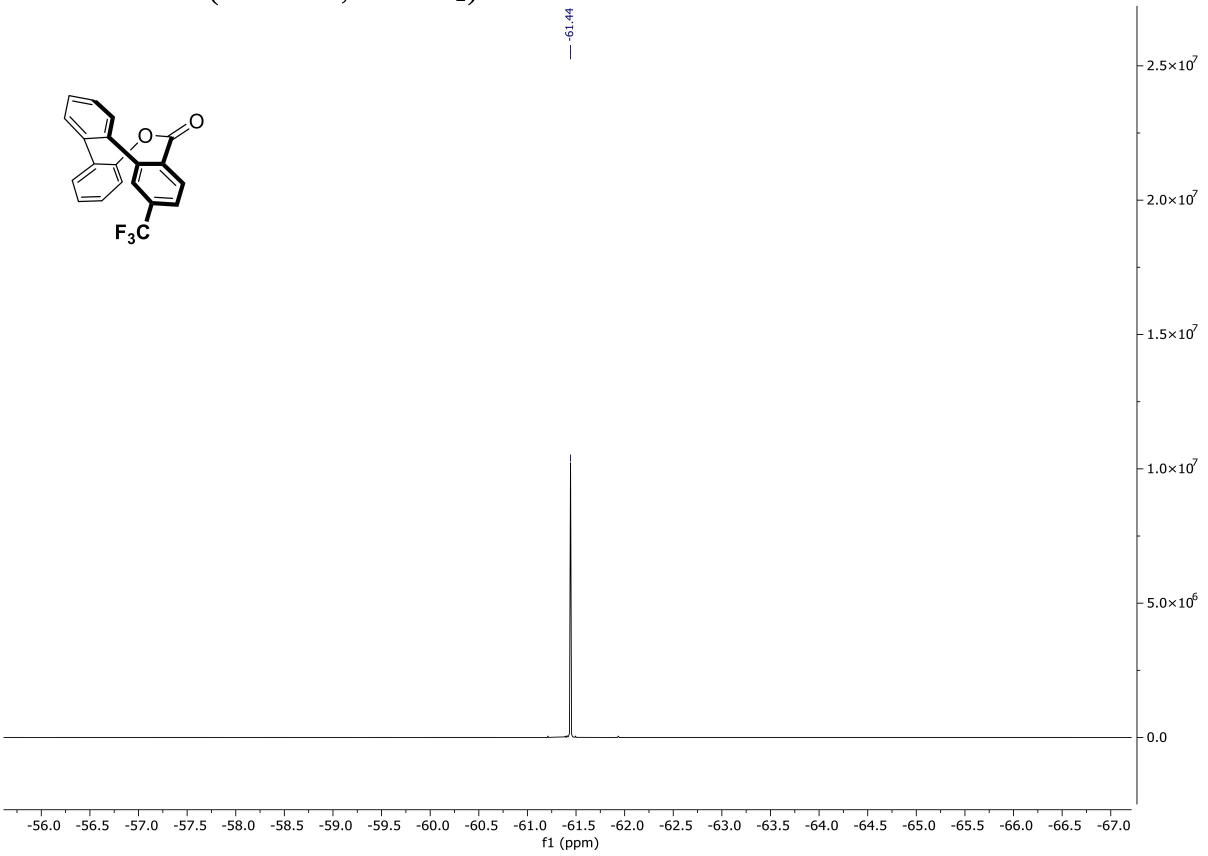

13-Nitro-10*H*-tribenzo[*b*,*d*,*f*]oxocin-10-one (2l)

^{1}H NMR of **2l** (400 MHz, DCM-d_2)

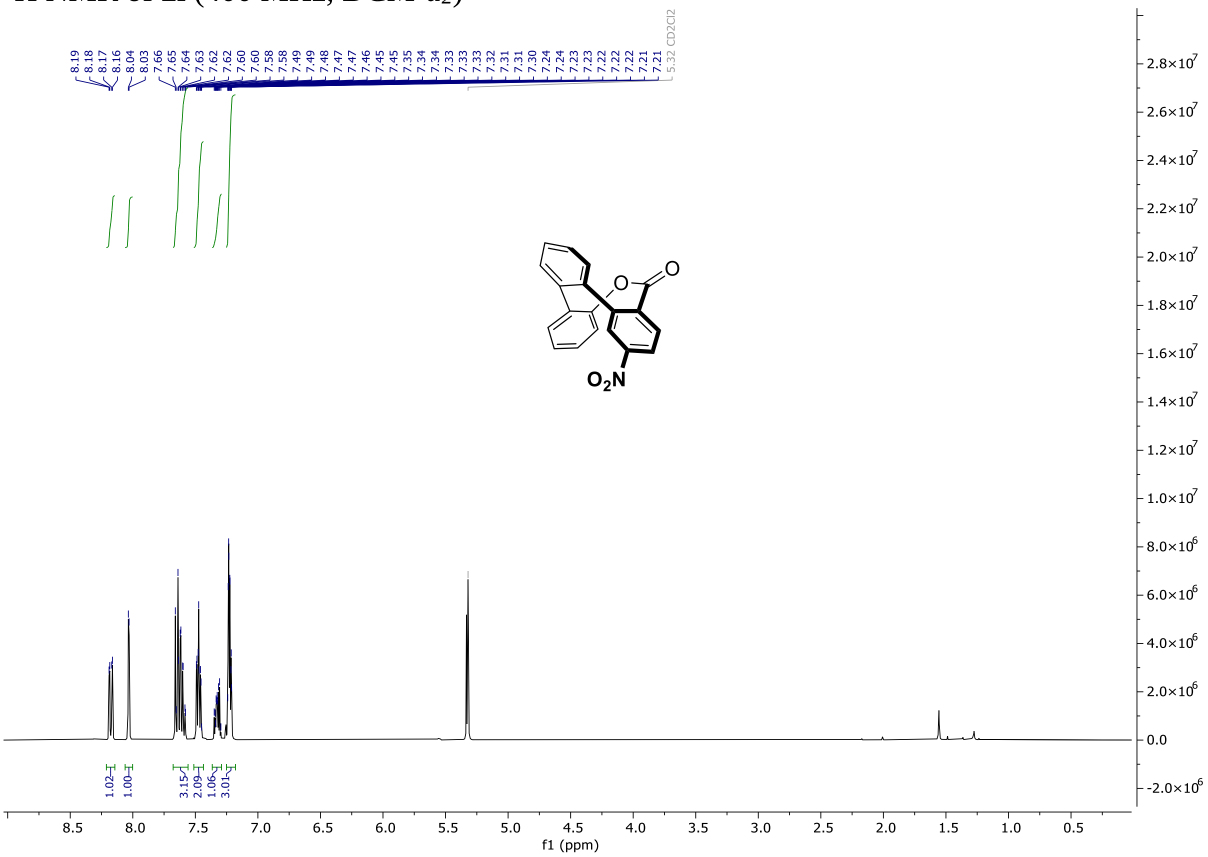

$^{13}C\{^{1}H\}$ NMR of **2l** (101 MHz, DCM-d_2)

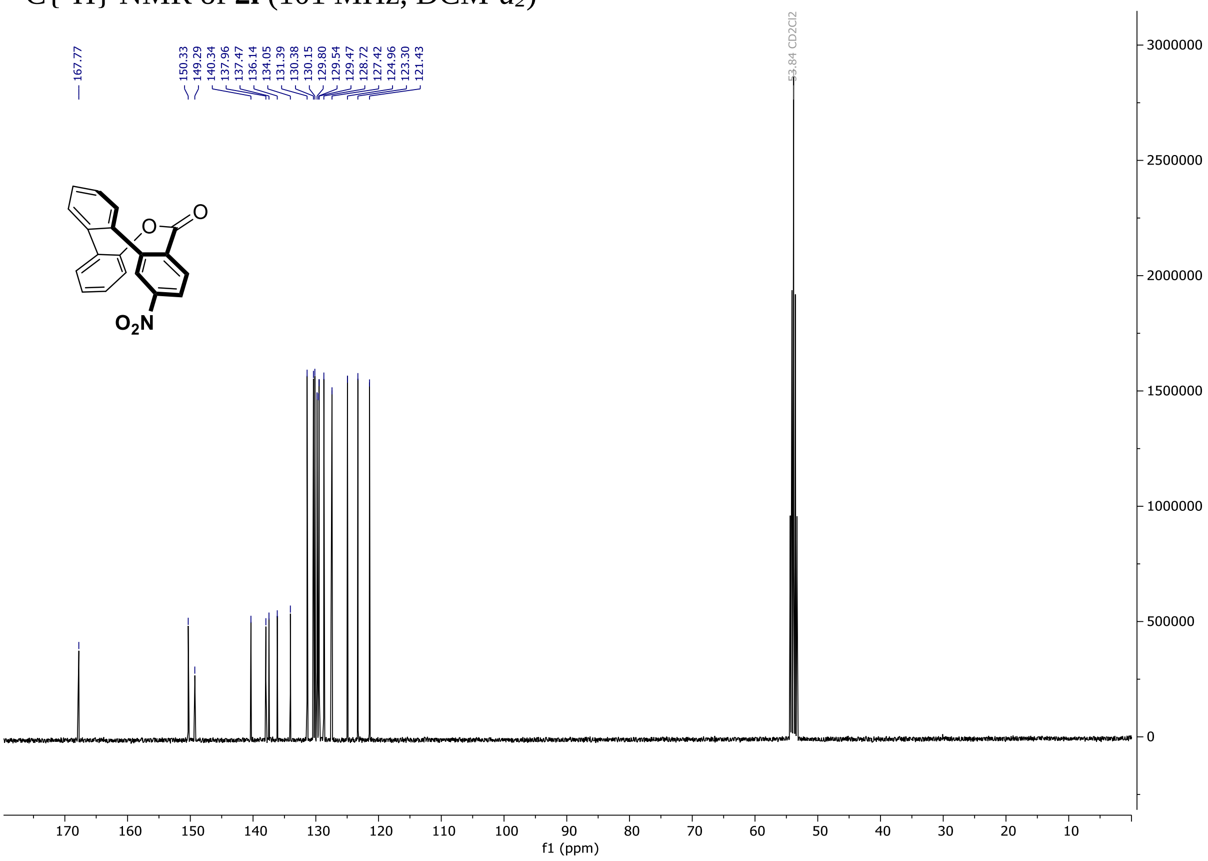

6-Methyl-10*H*-tribenzo[*b*,*d*,*f*]oxocin-10-one (2m)

^{1}H NMR of **2m** (400 MHz, DCM-d_2)

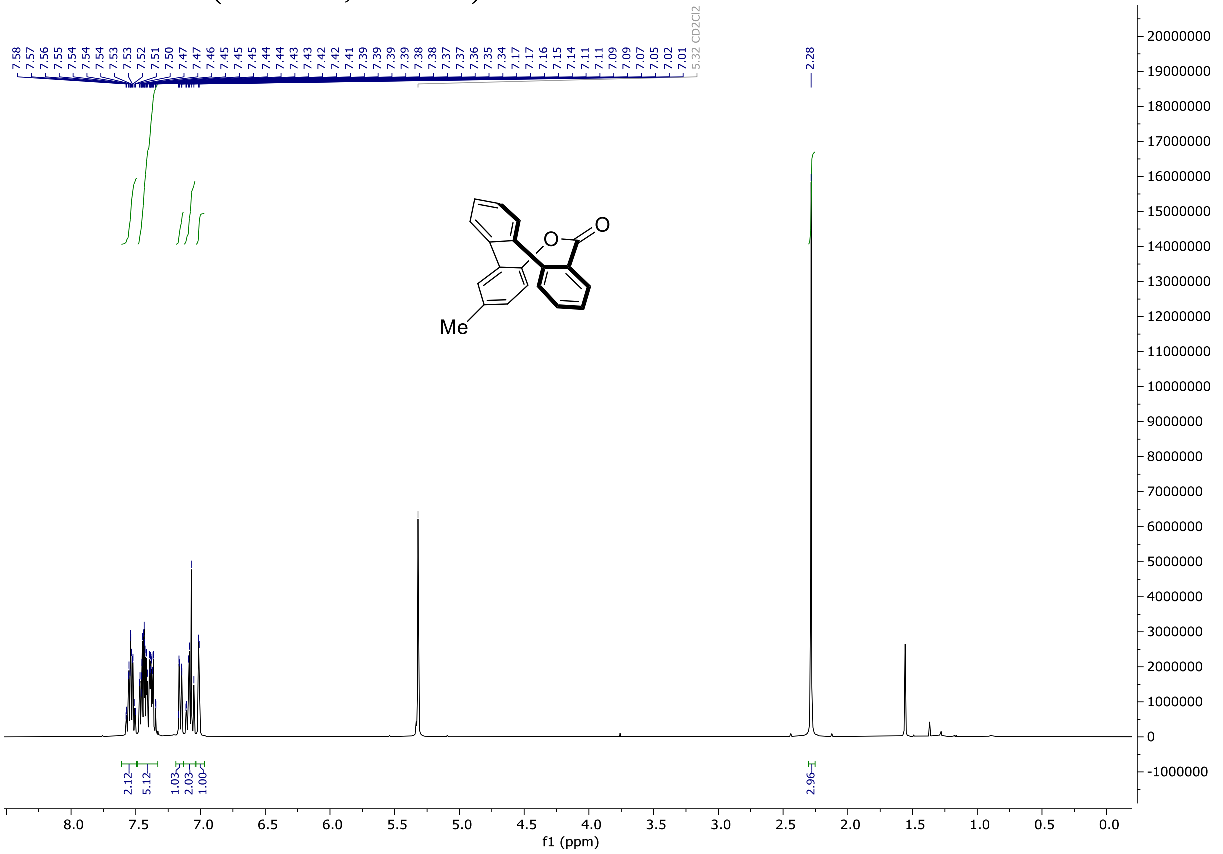

^{13}C{^{1}H} NMR of **2m** (101 MHz, DCM-d_2)

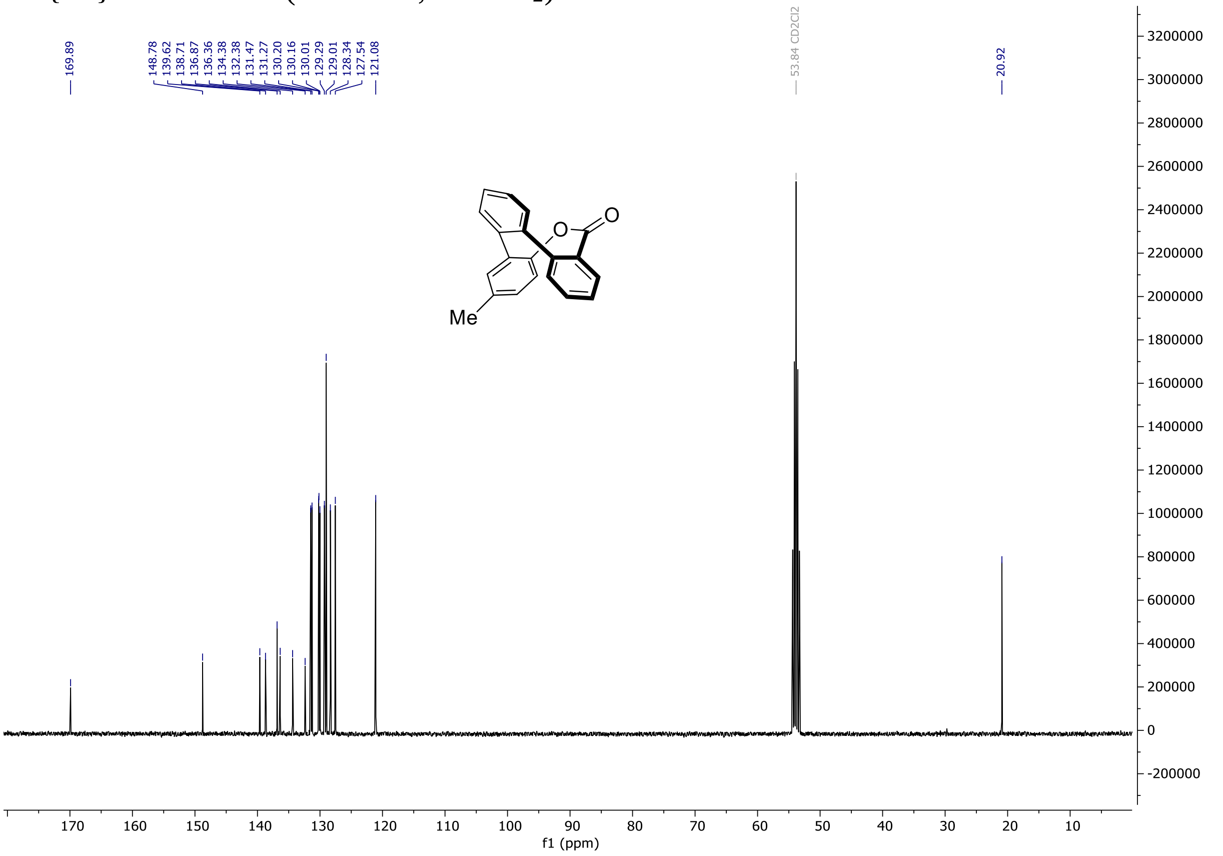

5-Methyl-10*H*-tribenzo[*b*,*d*,*f*]oxocin-10-one (2n)

^{1}H NMR of **2n** (400 MHz, chloroform-*d*)

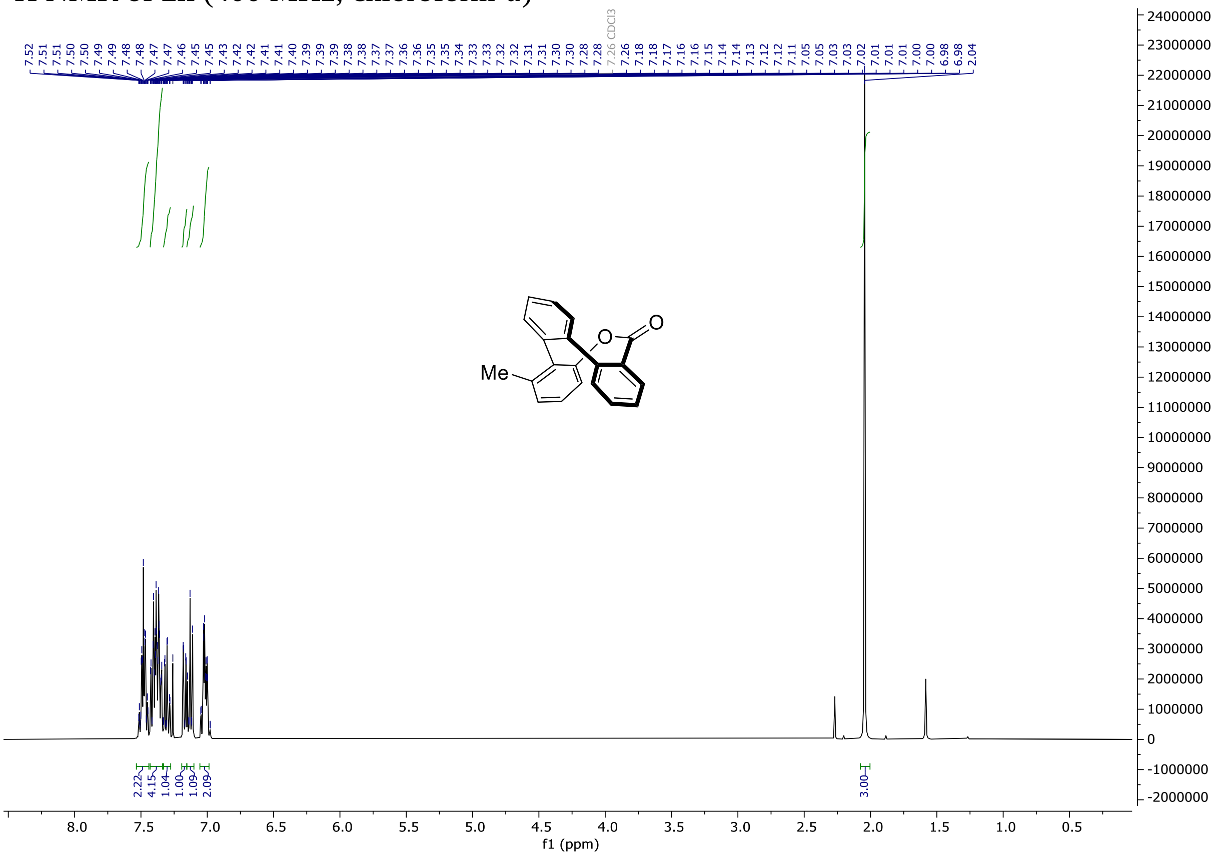

^{13}C{^{1}H} NMR of **2n** (101 MHz, chloroform-*d*)

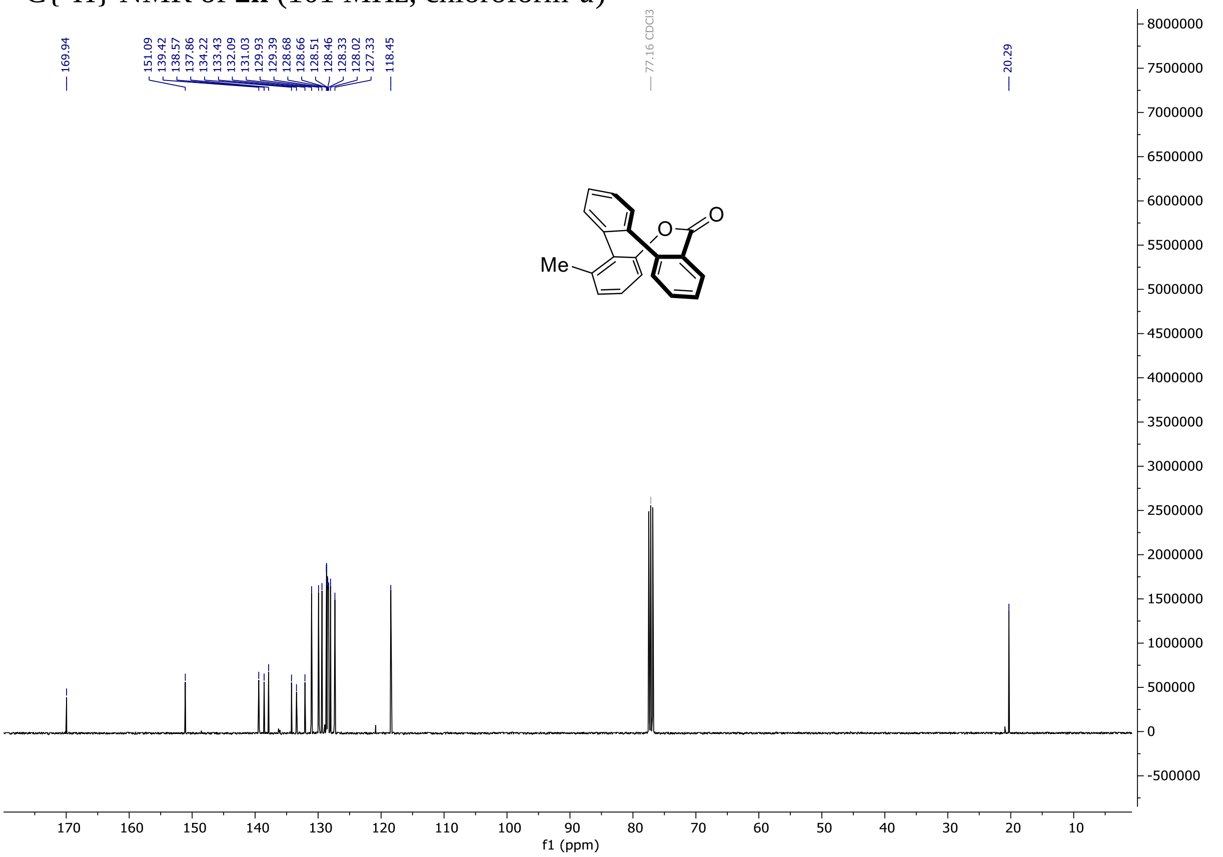

7-Methyl-10*H*-tribenzo[*b*,*d*,*f*]oxocin-10-one (2o)

^{1}H NMR of **2o** (400 MHz, chloroform-*d*)

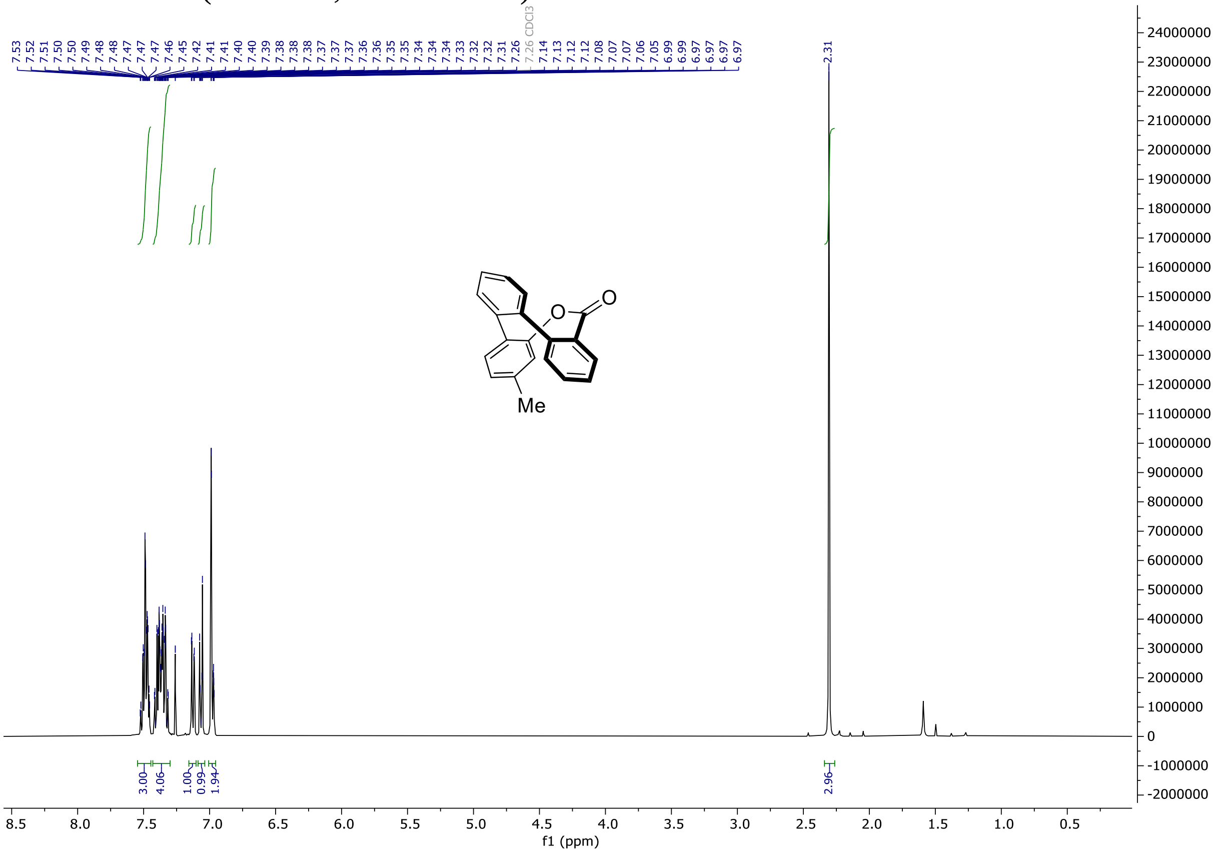

^{13}C{^{1}H} NMR of **2o** (101 MHz, chloroform-*d*)

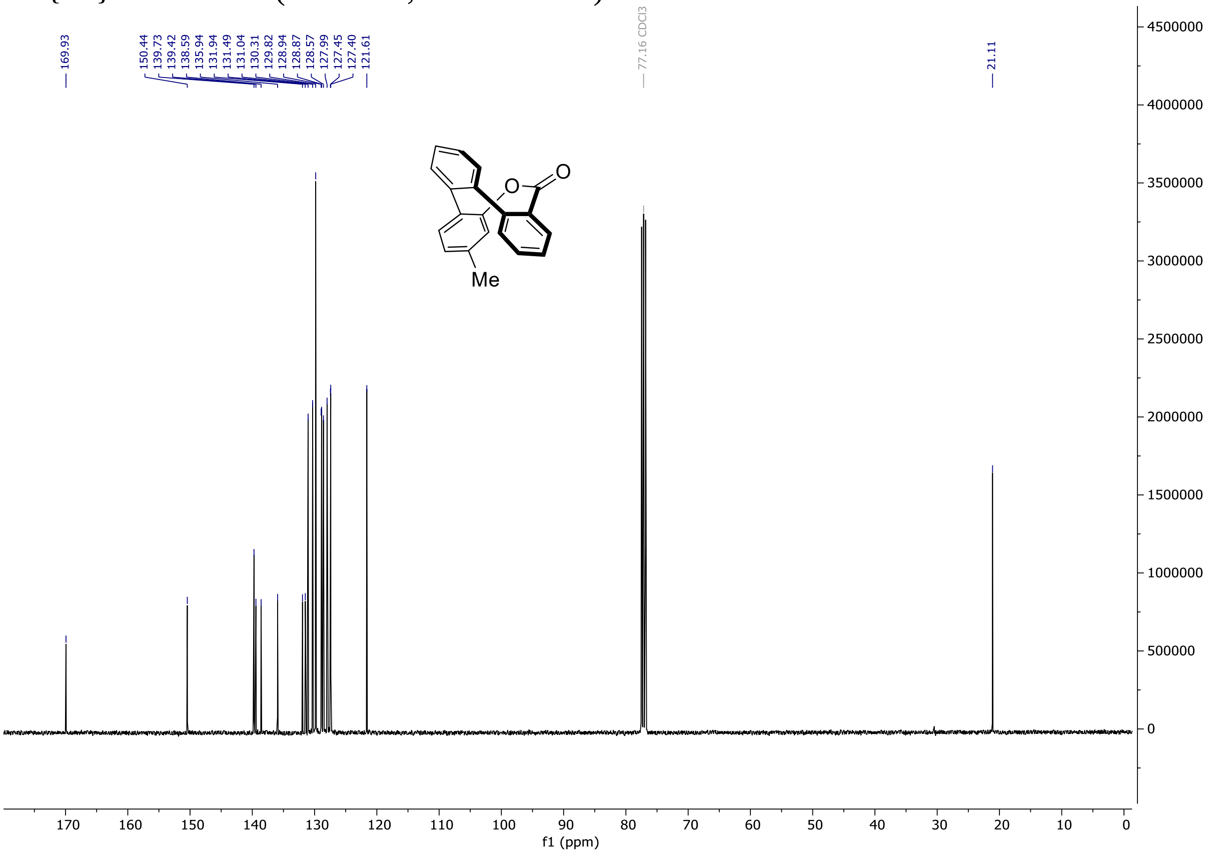

6-Methyl-10*H*-tribenzo[*b*,*d*,*f*]oxocin-10-one (2p)

^{1}H NMR of **2p** (400 MHz, chloroform-*d*)

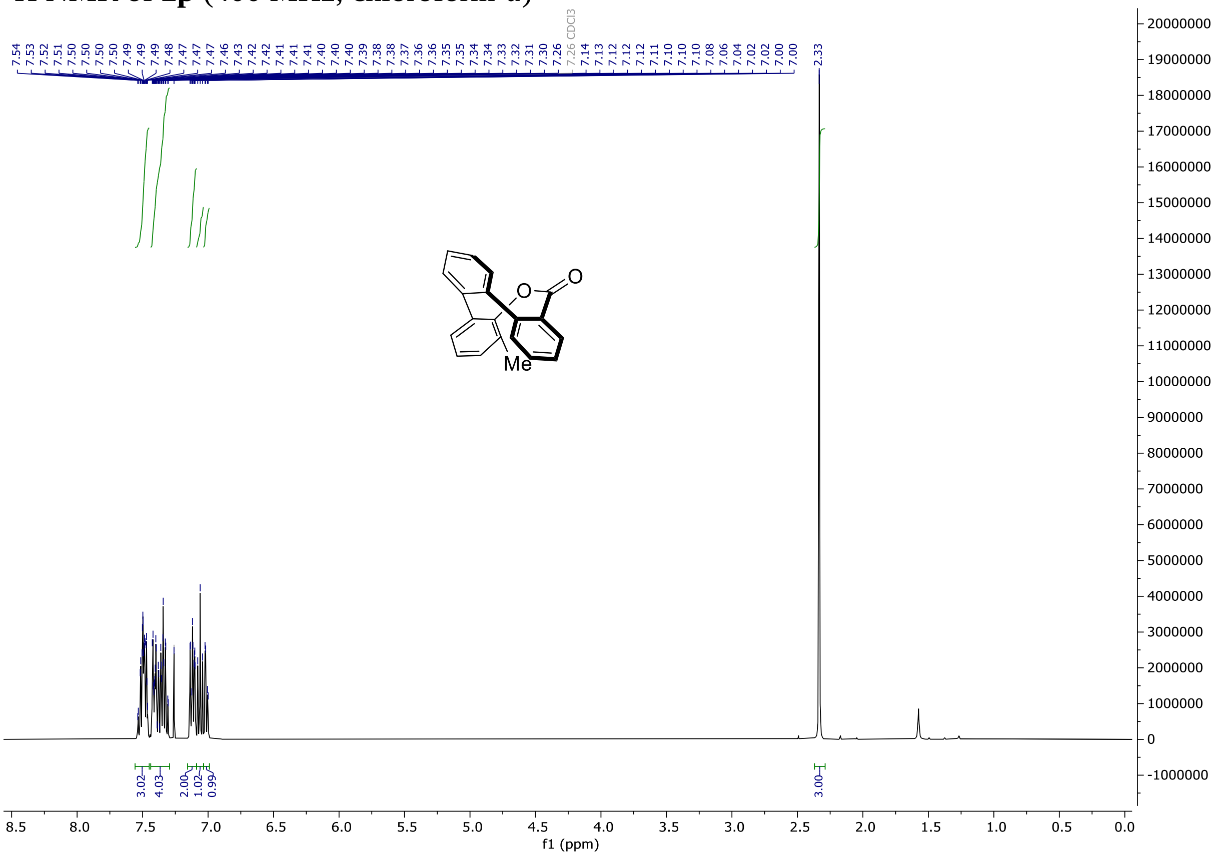

^{13}C{^{1}H} NMR of **2p** (101 MHz, chloroform-*d*)

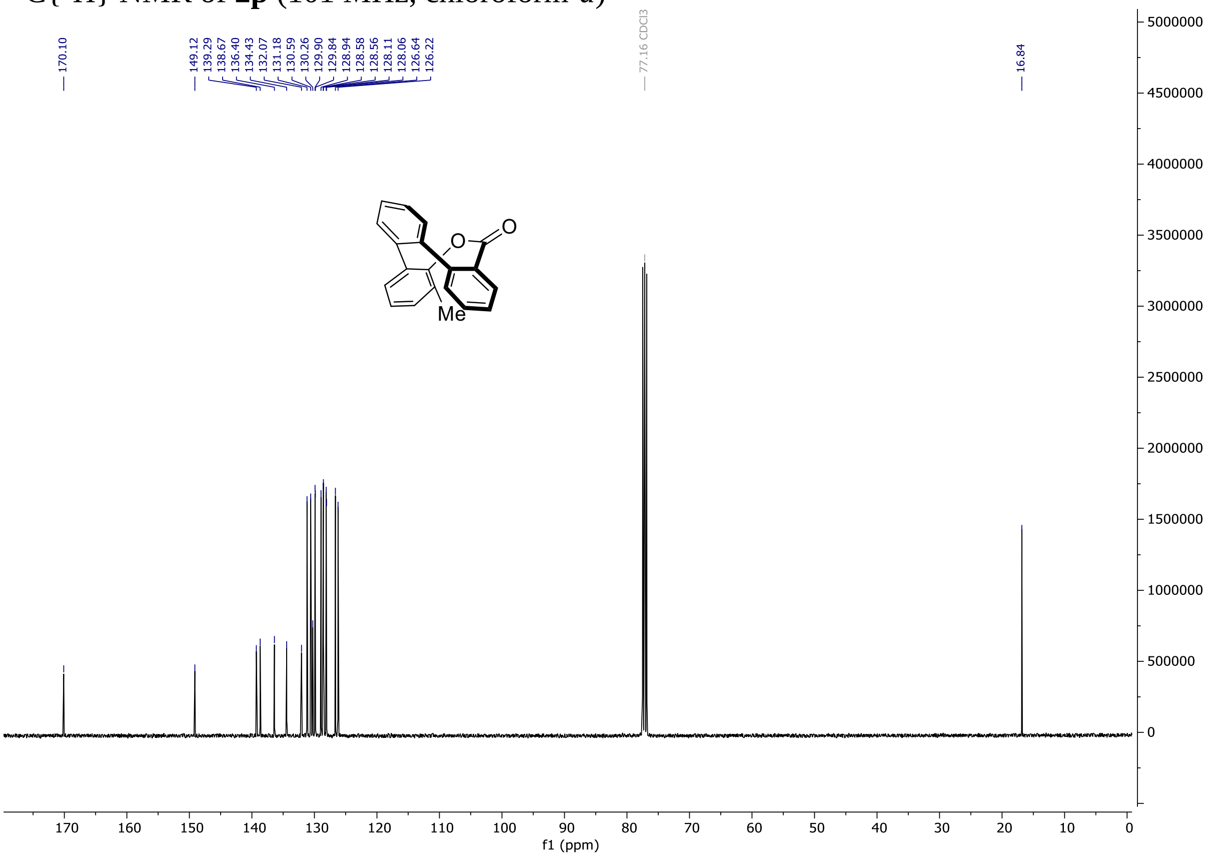

6-Fluoro-10*H*-tribenzo[*b*,*d*,*f*]oxocin-10-one (2q)

^{1}H NMR of **2q** (400 MHz, DCM-d_2)

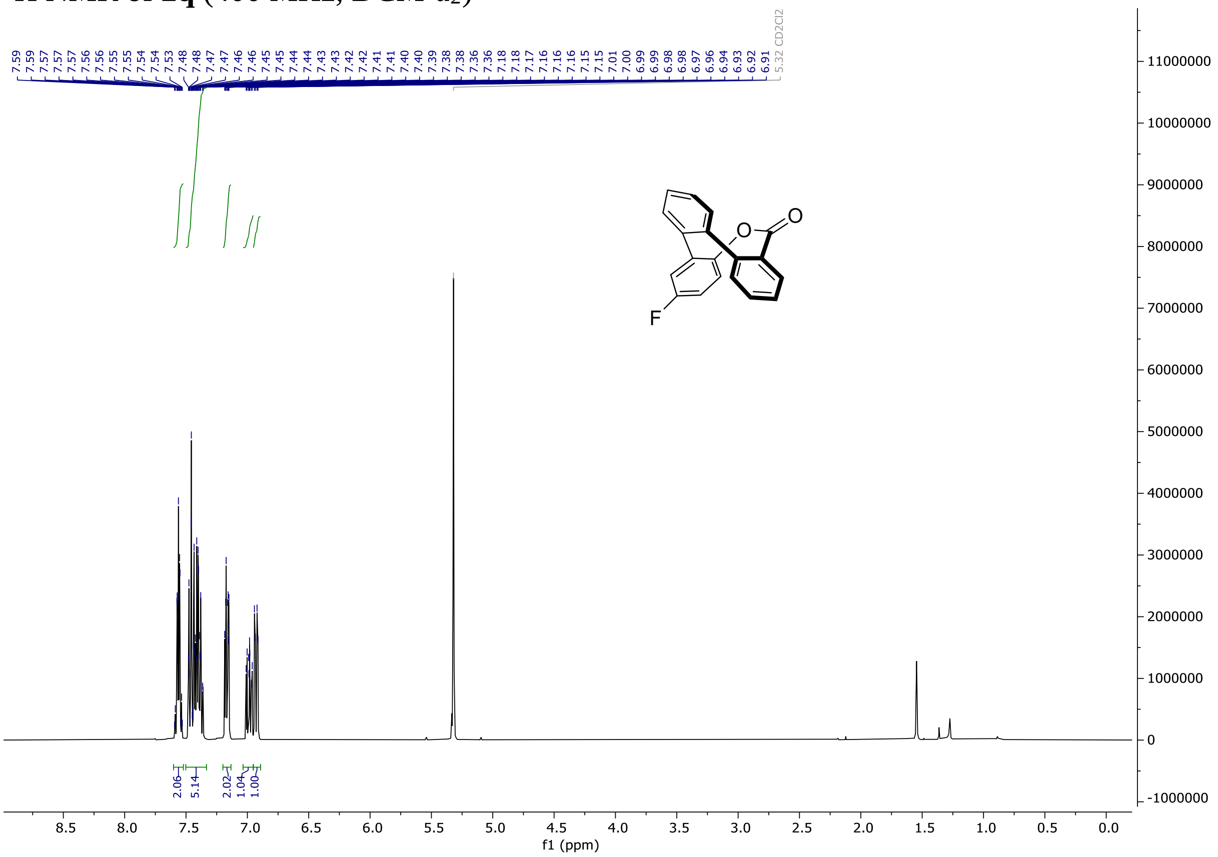

^{13}C{^{1}H} NMR of **2q** (101 MHz, DCM-d_2)

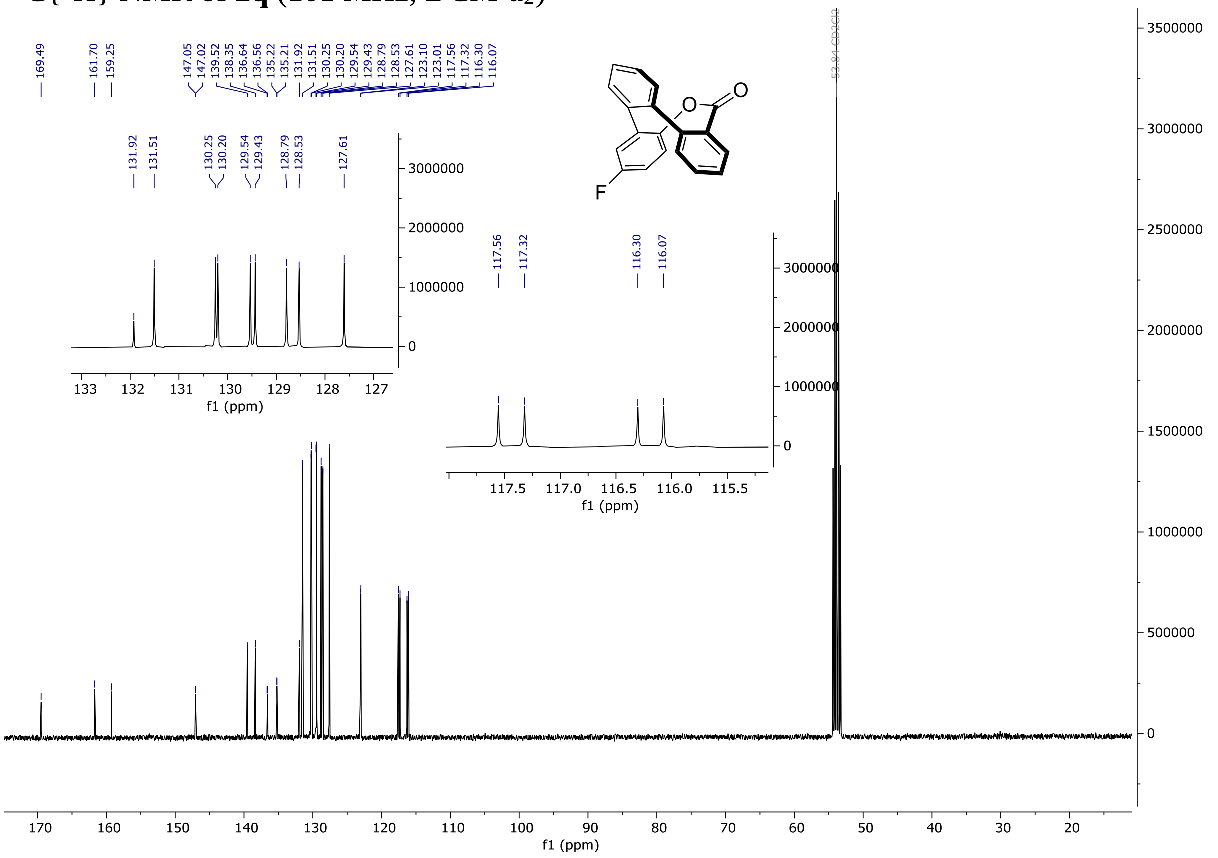

^{19}F NMR of **2q** (376 MHz, DCM-d_2)

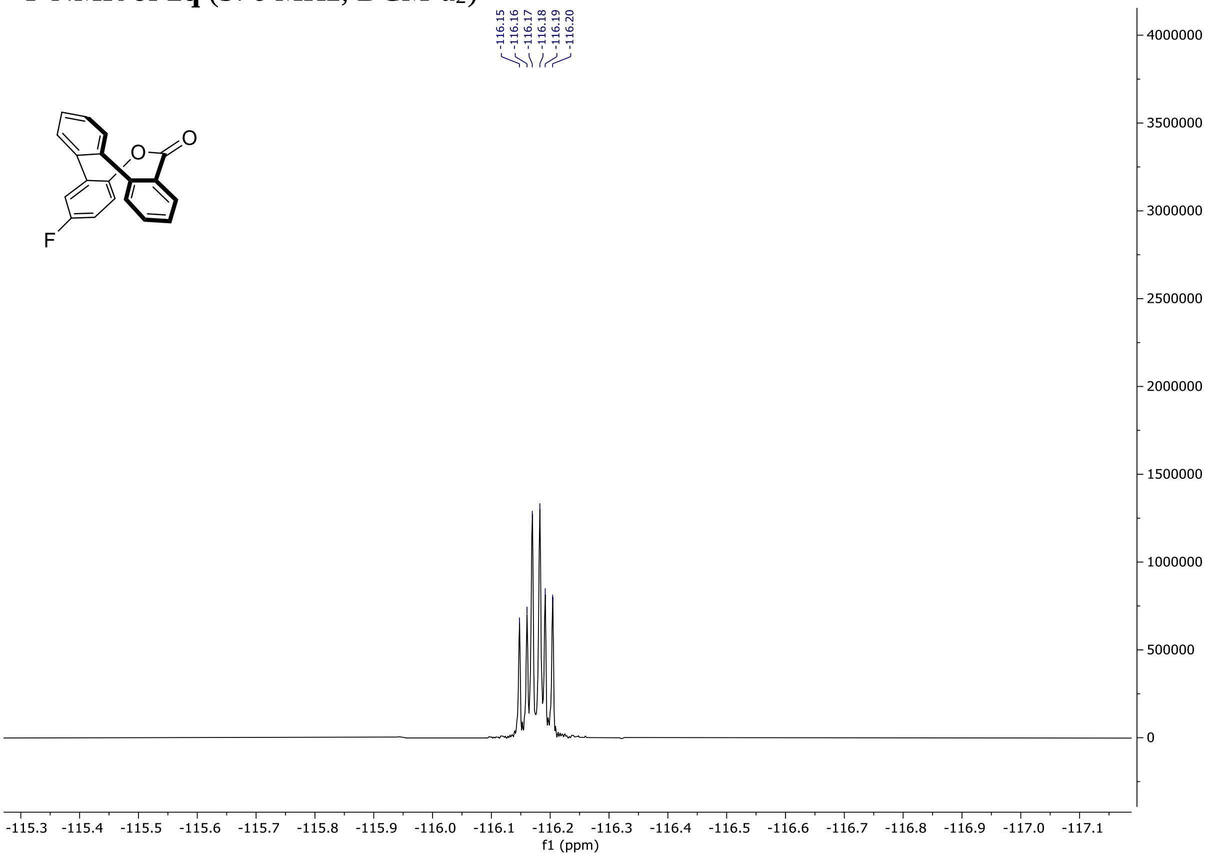

6-Chloro-10*H*-tribenzo[*b*,*d*,*f*]oxocin-10-one (2r)

^{1}H NMR of **2r** (400 MHz, DCM-d_2)

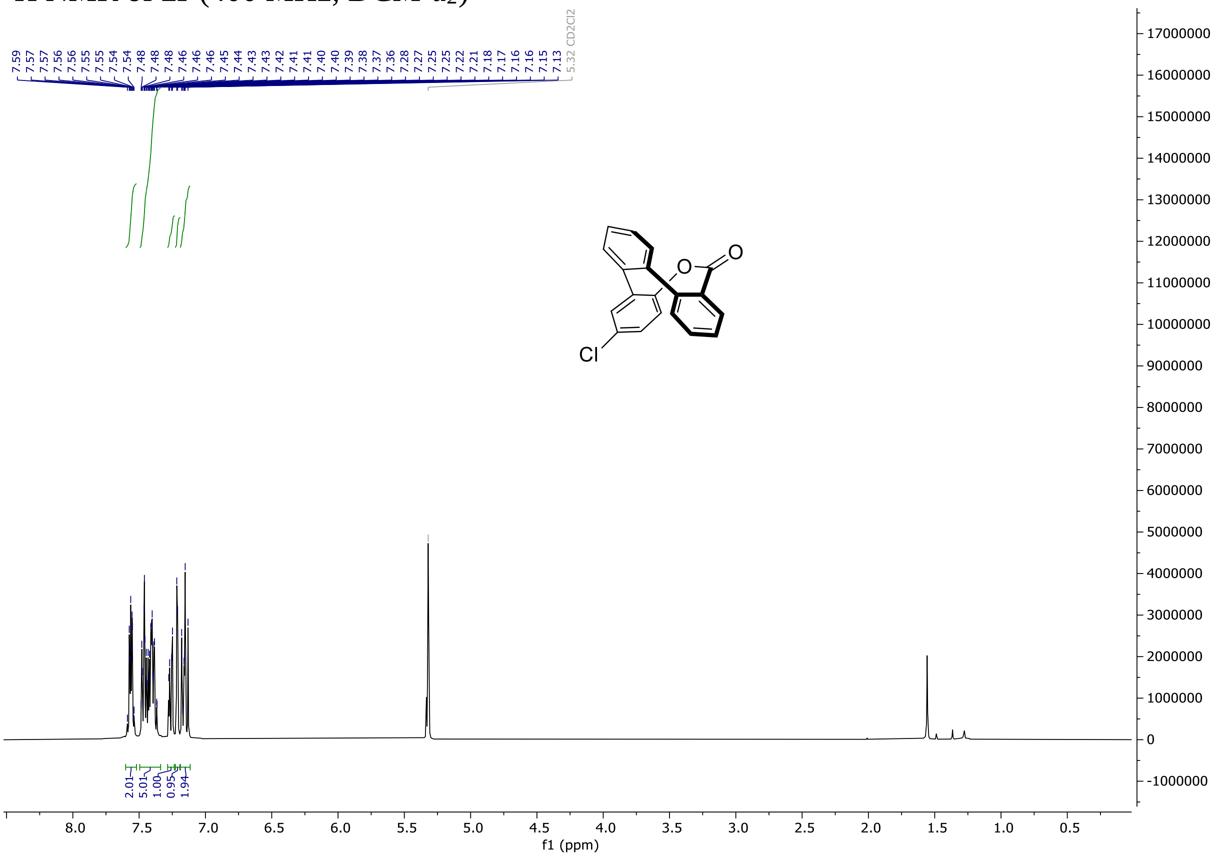

^{13}C{^{1}H} NMR of **2r** (101 MHz, DCM-d_2)

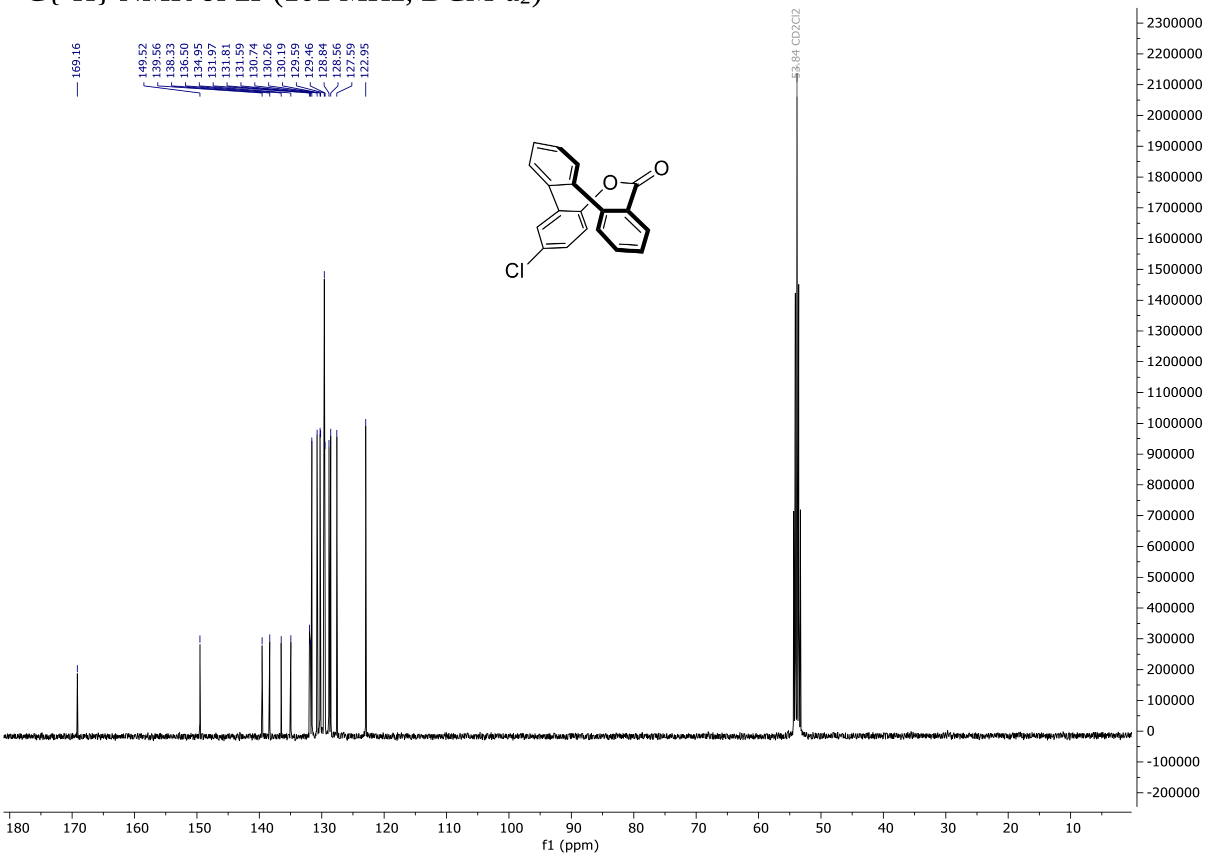

6-Chloro-10*H*-tribenzo[*b*,*d*,*f*]oxocin-10-one (2s)

^{1}H NMR of **2s** (400 MHz, DCM-d_2)

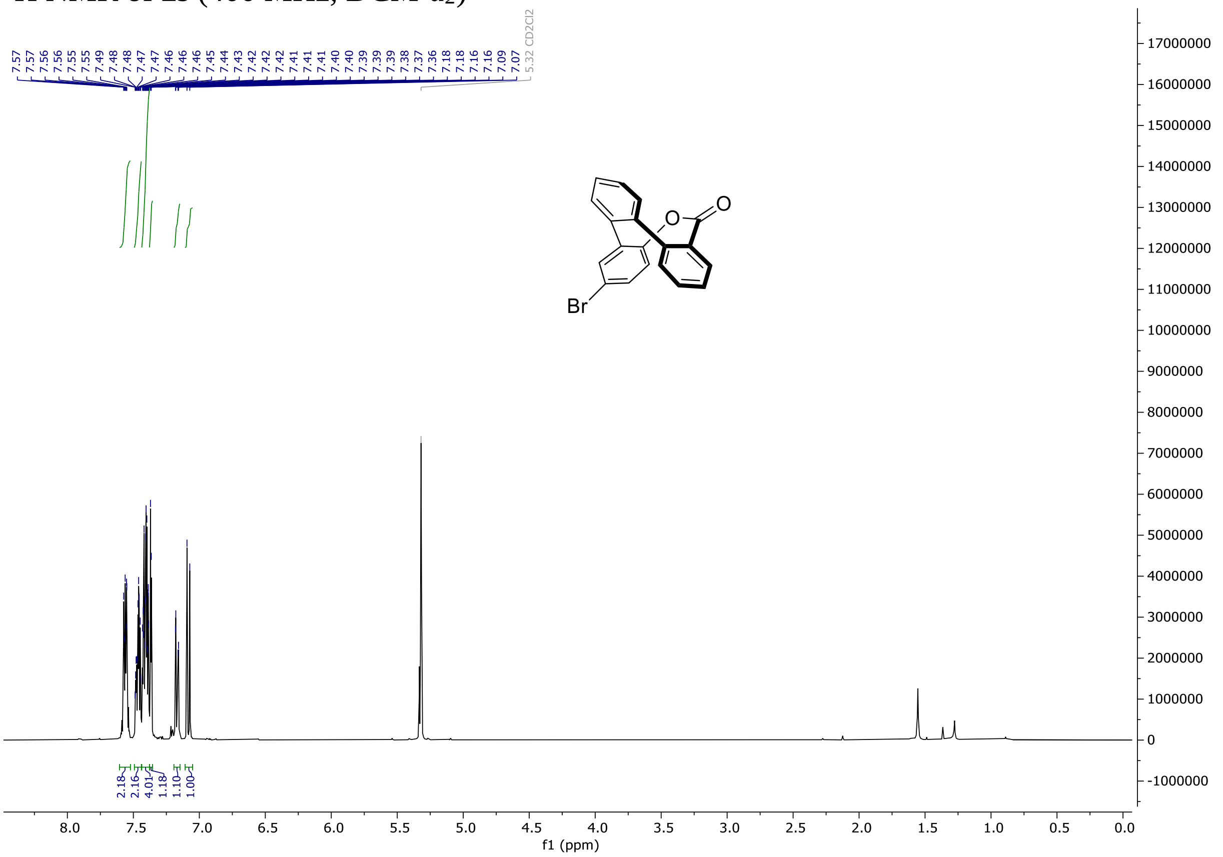

^{13}C{^{1}H} NMR of **2s** (101 MHz, DCM-d_2)

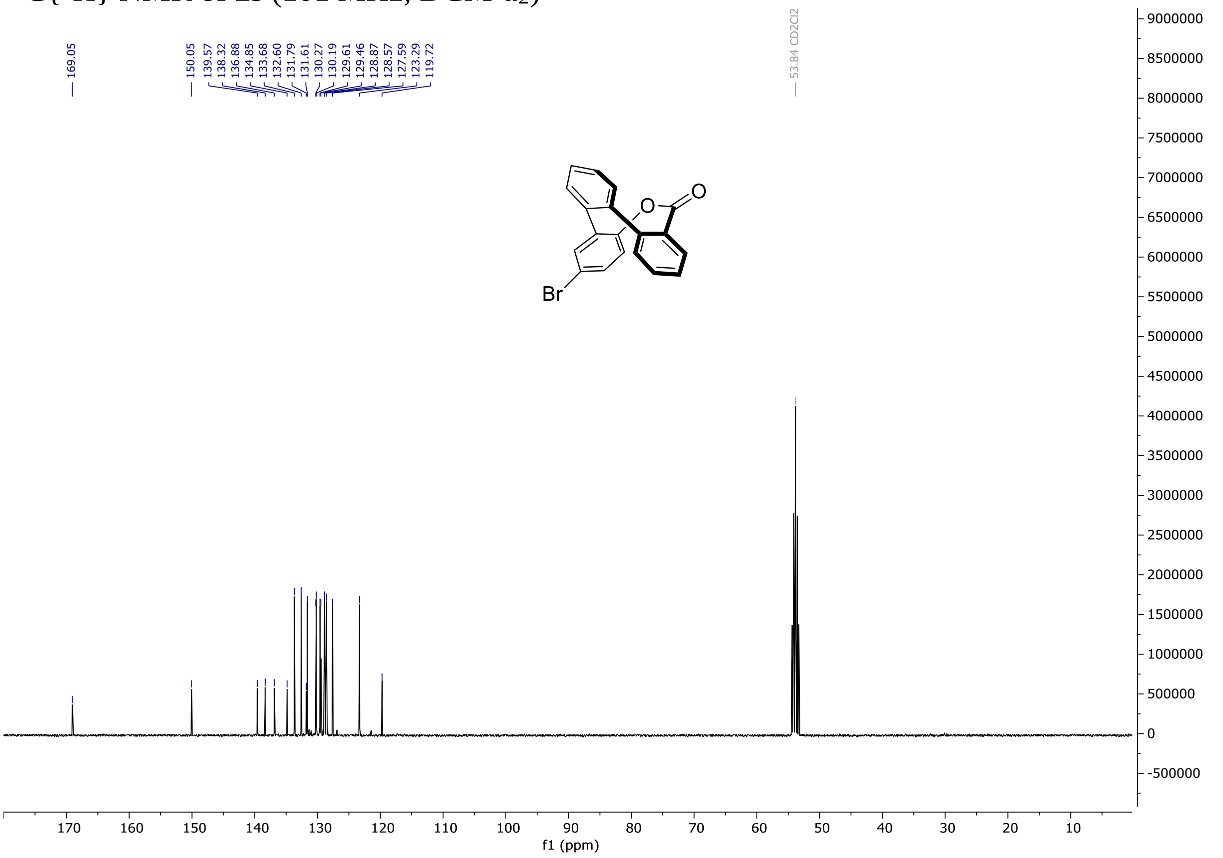

2-Methyl-10*H*-tribenzo[*b*,*d*,*f*]oxocin-10-one (2t)

^{1}H NMR of **2t** (400 MHz, DCM-d_2)

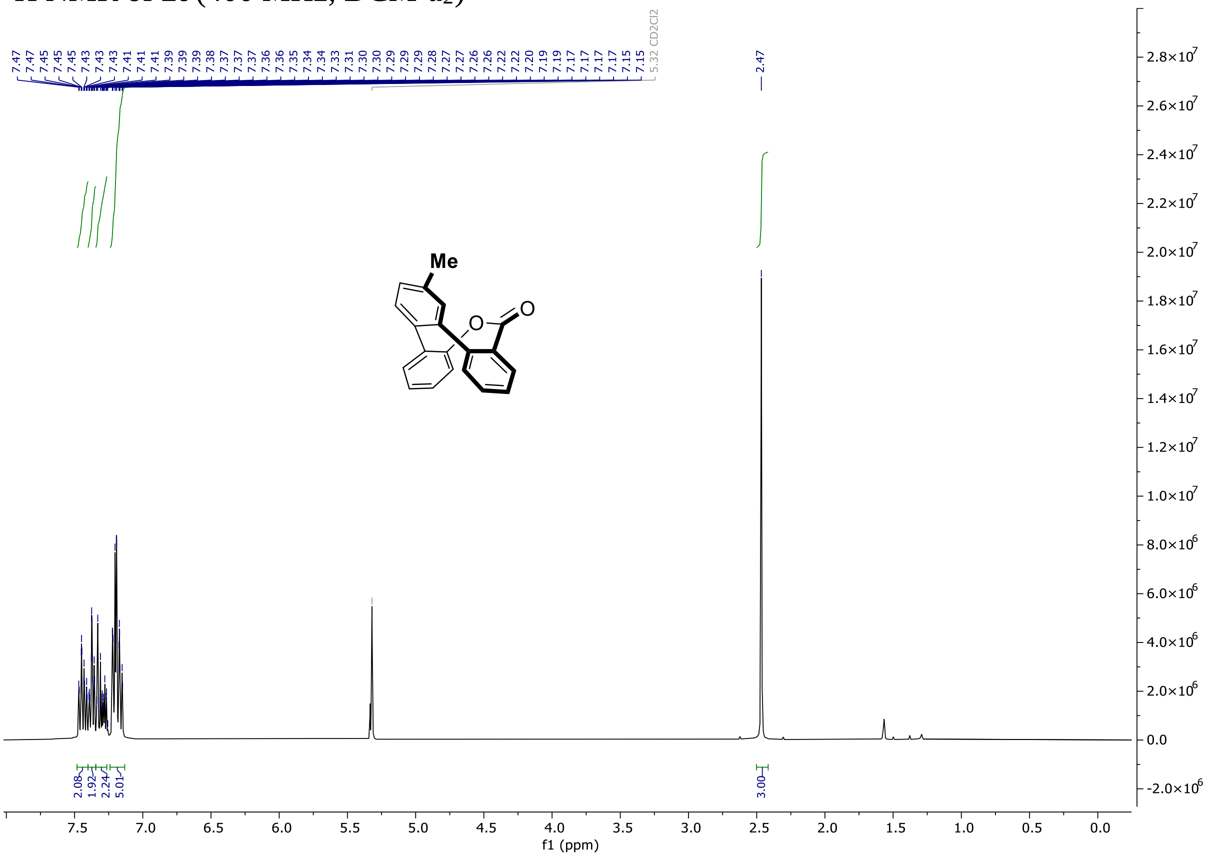

^{13}C{^{1}H} NMR of **2t** (101 MHz, DCM-d_2)

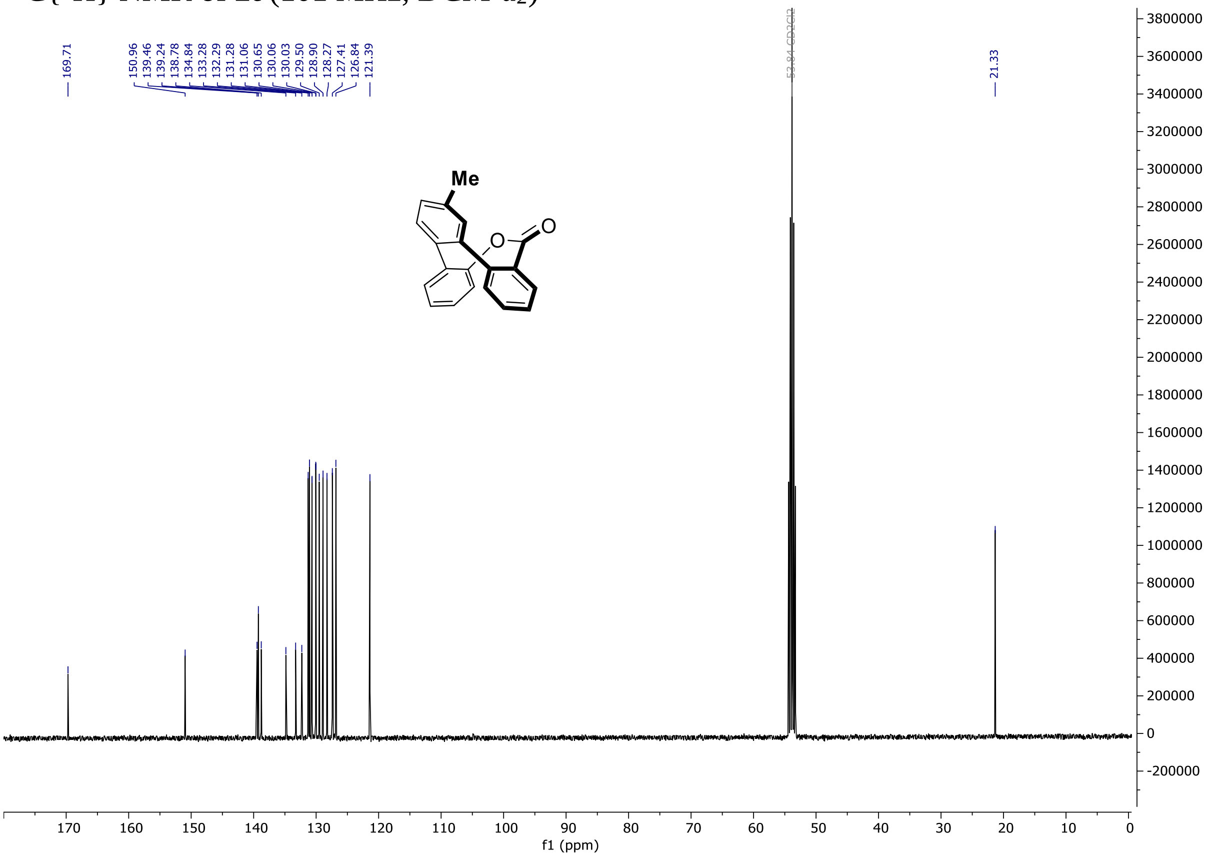

3-Methyl-10*H*-tribenzo[*b*,*d*,*f*]oxocin-10-one (2u)

^{1}H NMR of **2u** (400 MHz, DCM-d_2)

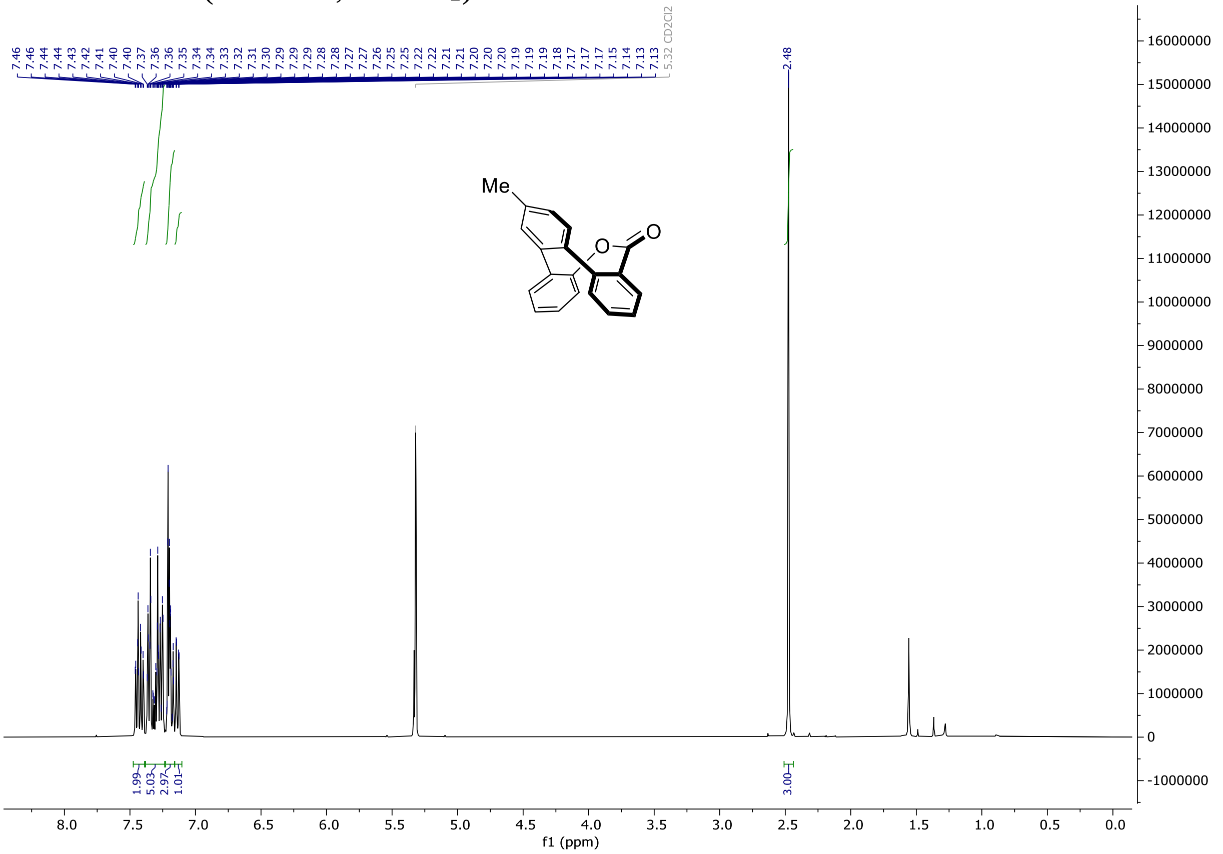

^{13}C{^{1}H} NMR of **2u** (101 MHz, DCM-d_2)

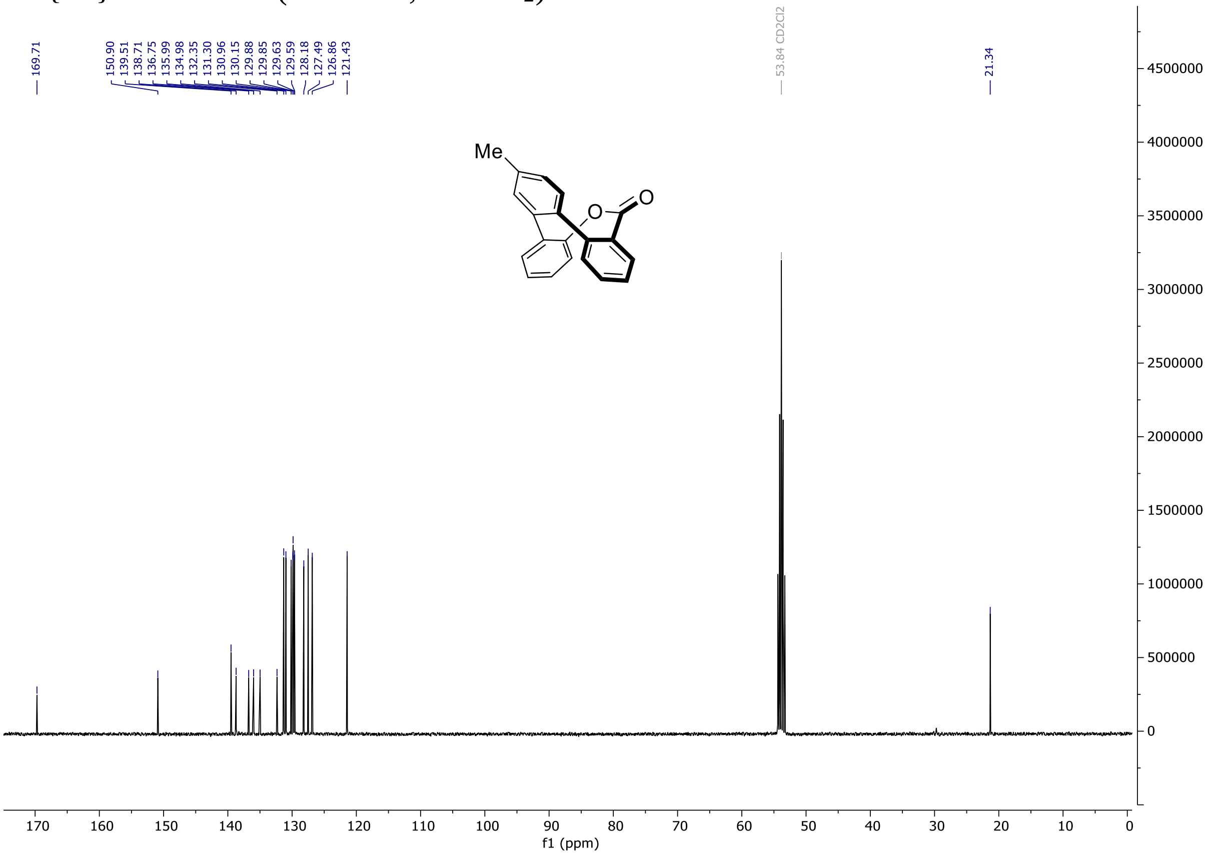

6,13-Dimethyl-10*H*-tribenzo[*b*,*d*,*f*]oxocin-10-one (2v)

^{1}H NMR of **2v** (400 MHz, DCM-d_2)

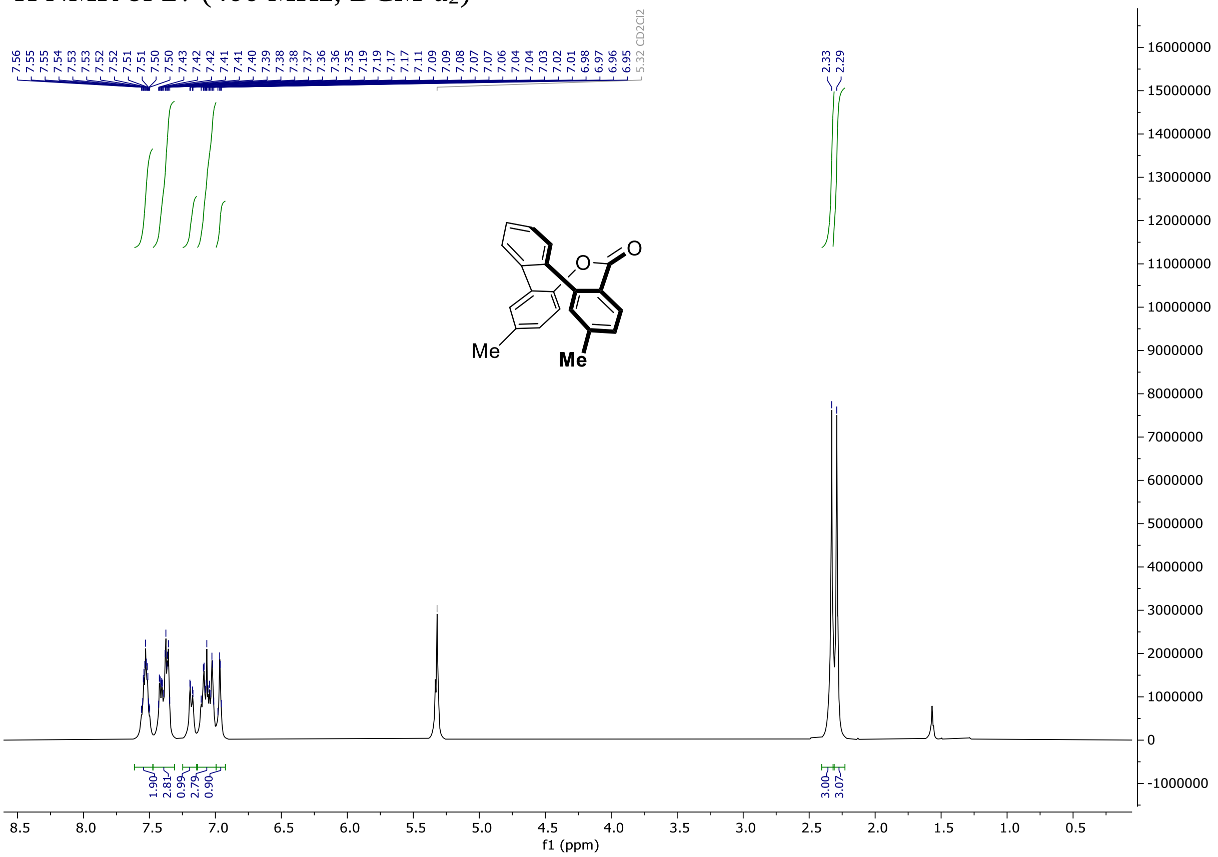

^{13}C{^{1}H} NMR of **2v** (101 MHz, DCM-d_2)

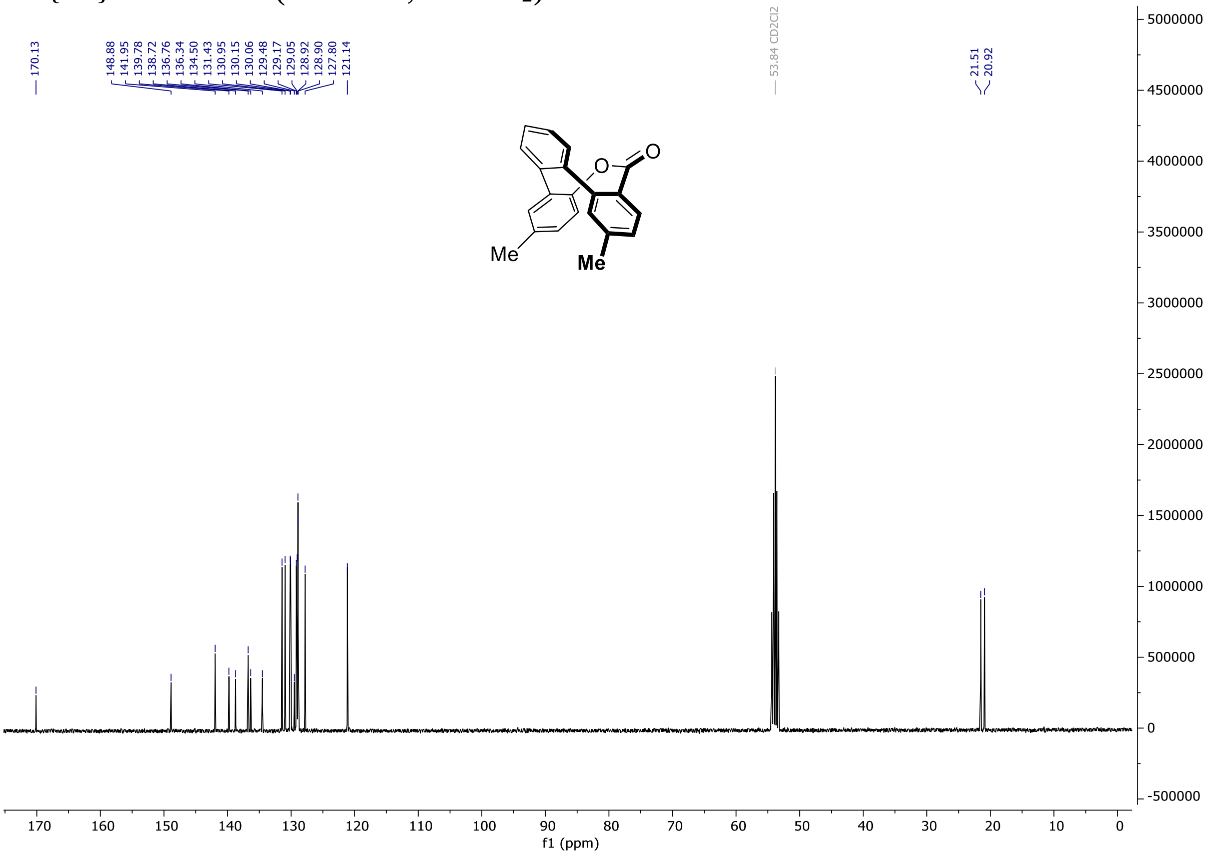

6,13-Difluoro-10*H*-tribenzo[*b*,*d*,*f*]oxocin-10-one (2w)

^{1}H NMR of **2w** (400 MHz, DCM-d_2)

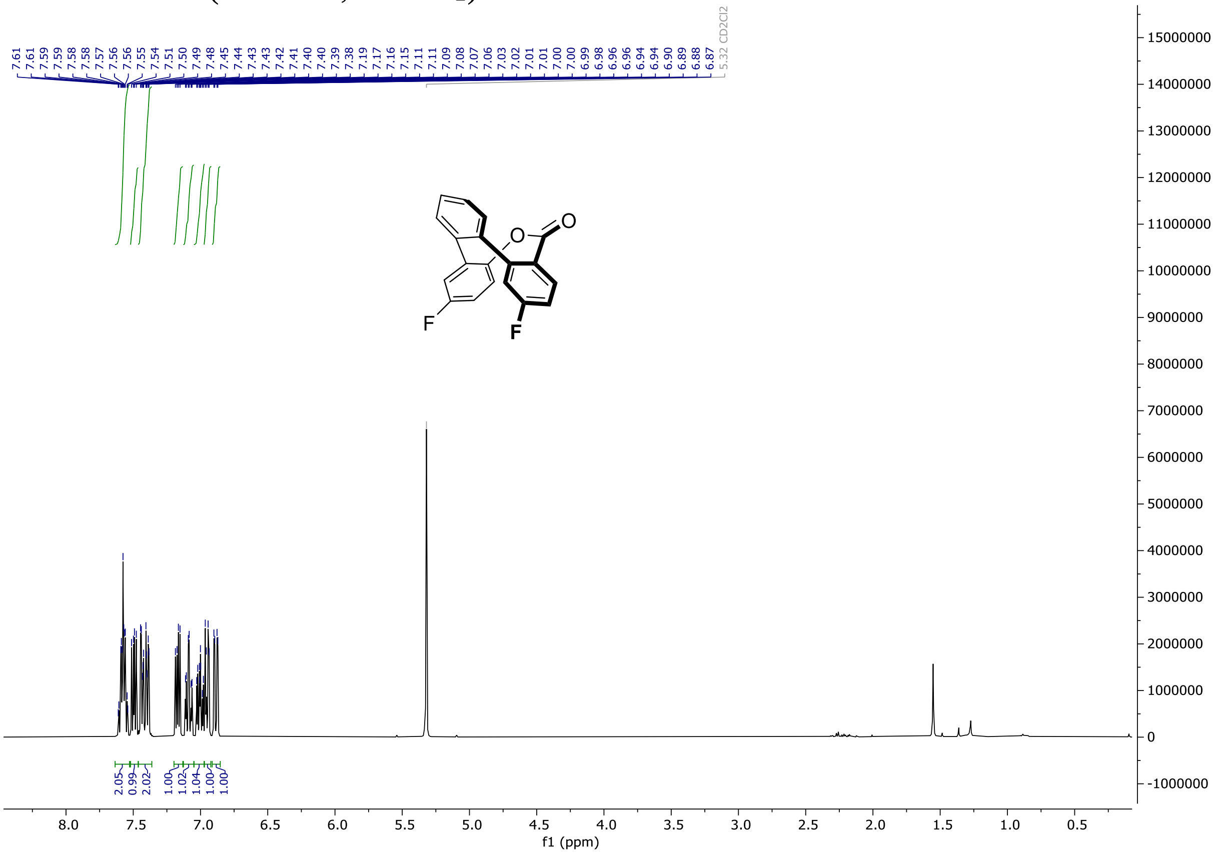

^{13}C{^{1}H} NMR of **2w** (101 MHz, DCM-d_2)

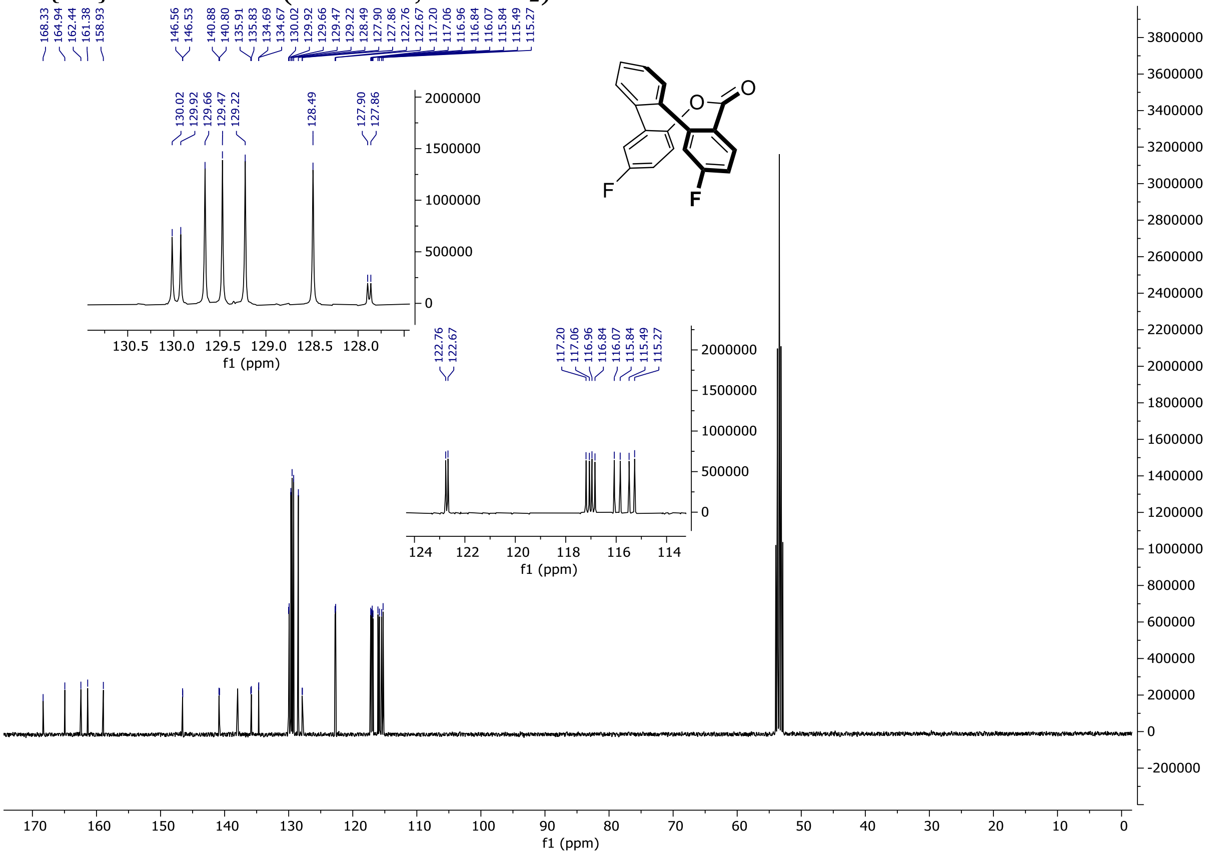

^{19}F NMR of **2w** (376 MHz, DCM-d_2)

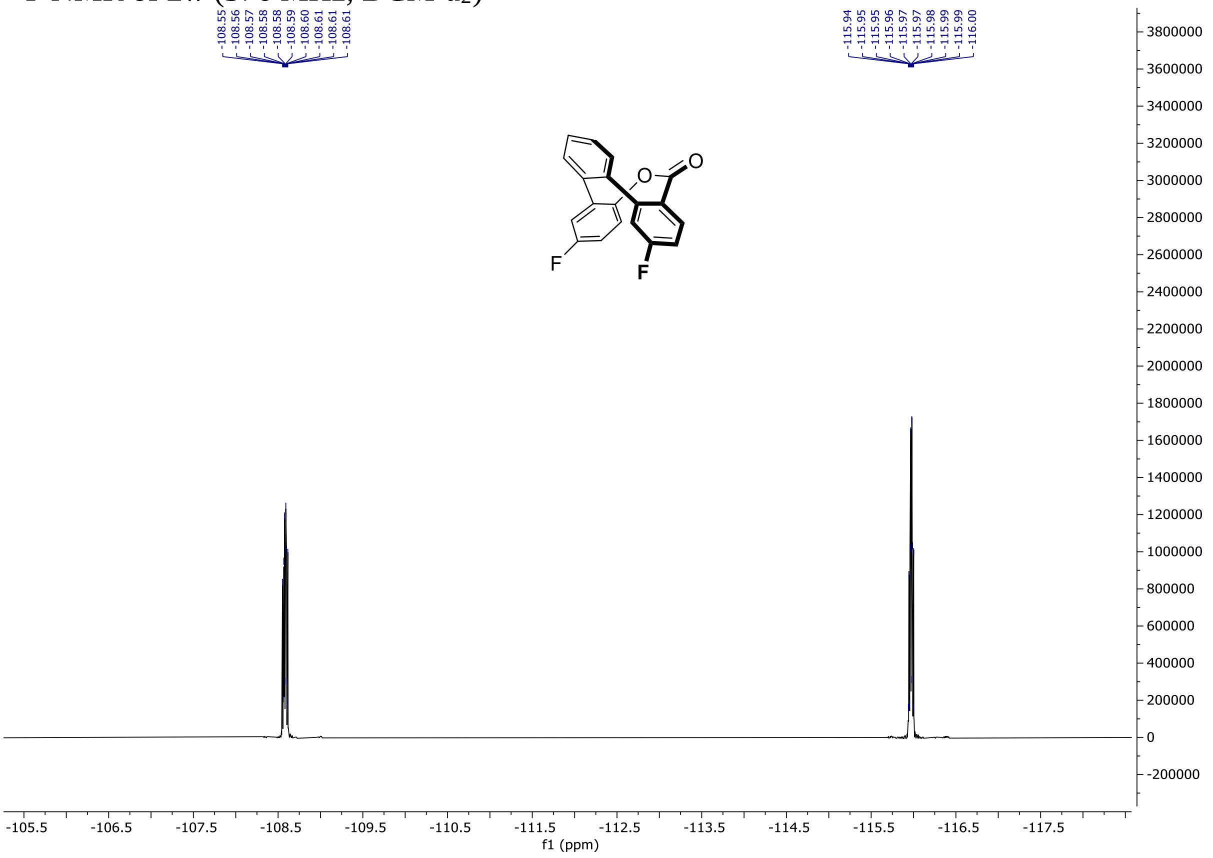

13-Fluoro-6-methyl-10*H*-tribenzo[*b*,*d*,*f*]oxocin-10-one (2x)

^{1}H NMR of **2x** (400 MHz, DCM-d_2)

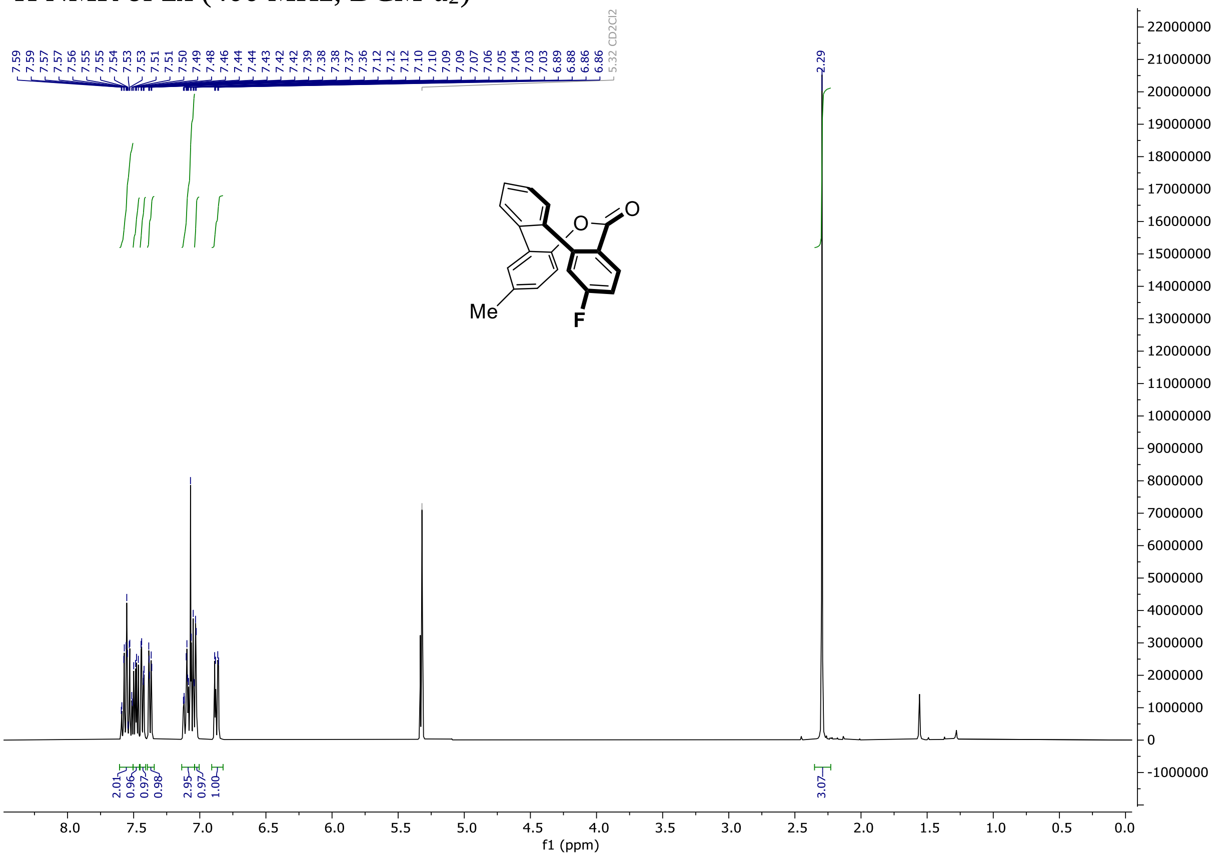

^{13}C{^{1}H} NMR of **2x** (101 MHz, DCM-d_2)

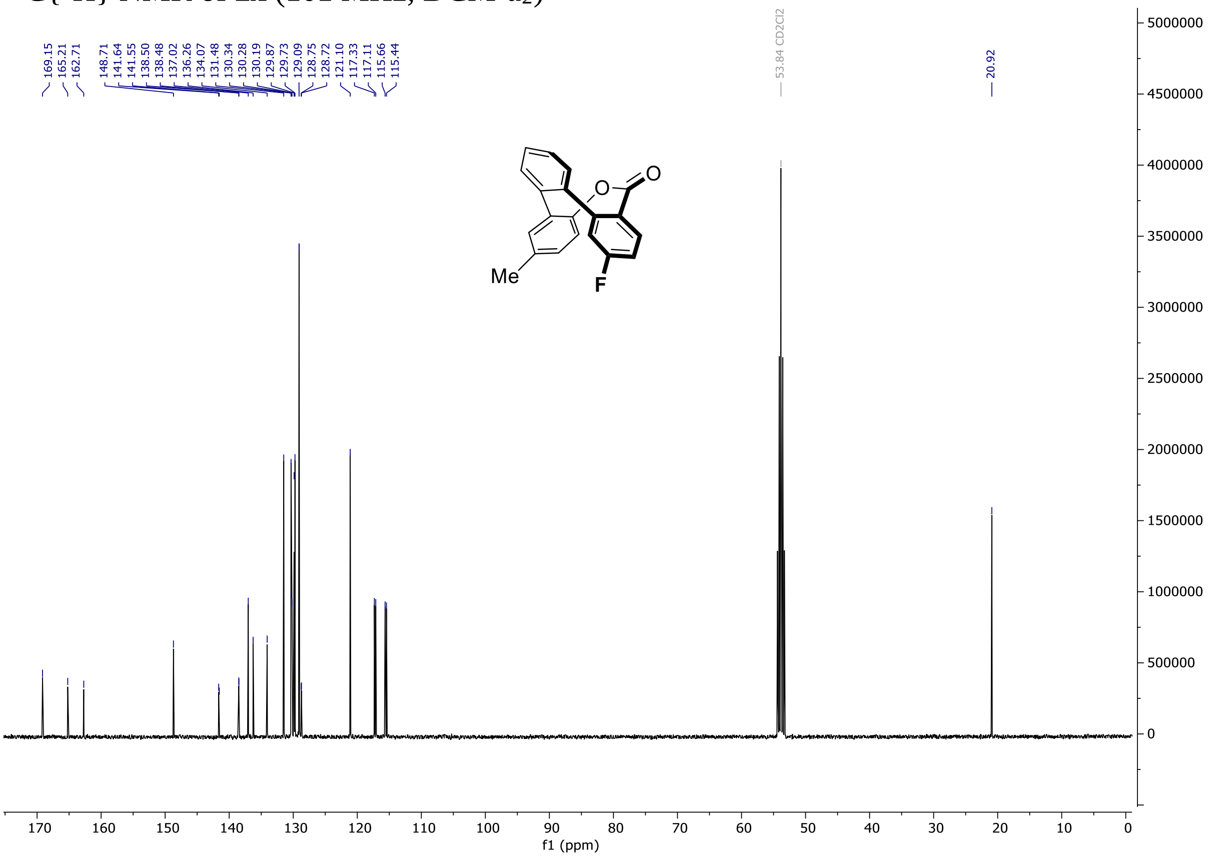

^{19}F NMR of **2x** (376 MHz, DCM-d_2)

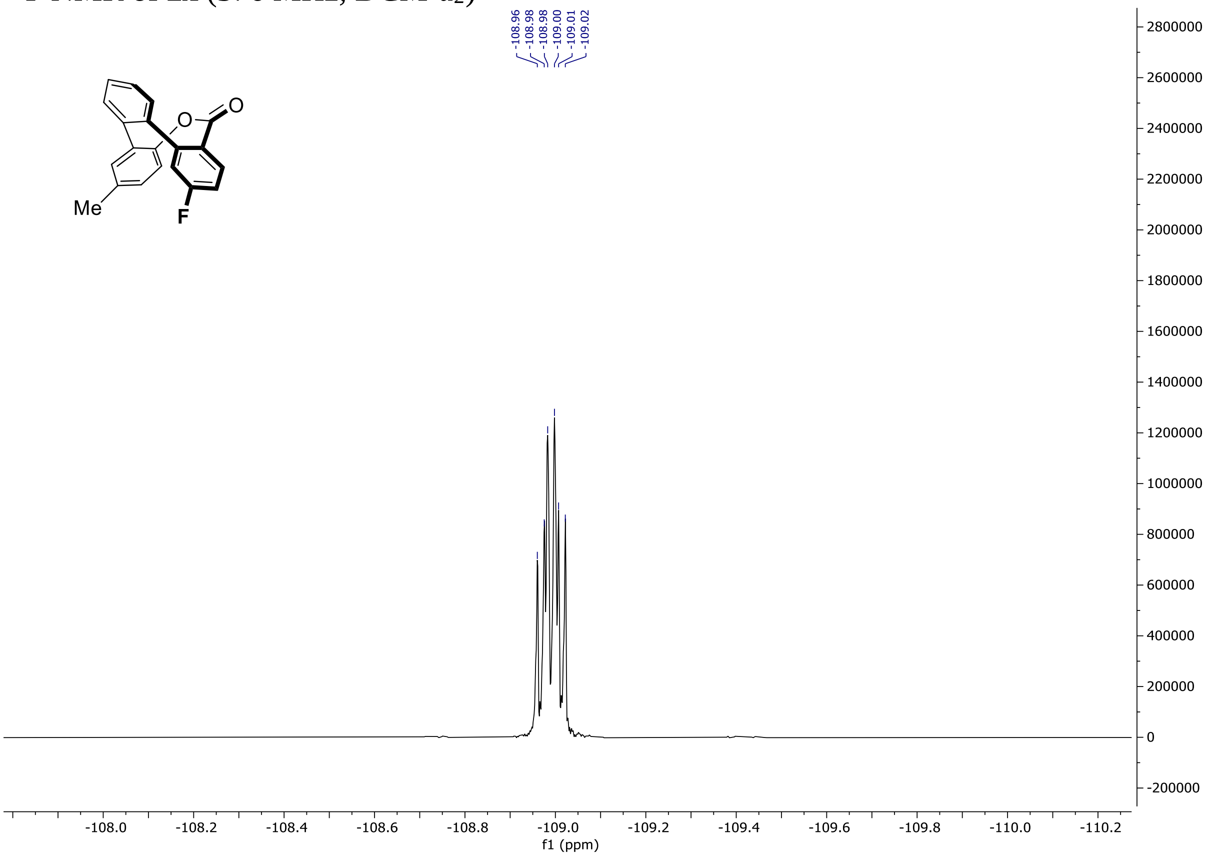

Chiral HPLC

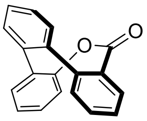

Conditions: Chiralpak IG column
mobile phase: *n*-heptane/propan-2-ol – 90:10
λ = 190 nm, V = 1.0 ml/min, t = 25 °C
for **2a**: t_R = 11.3 min (minor), t_R = 12.8 min (major)

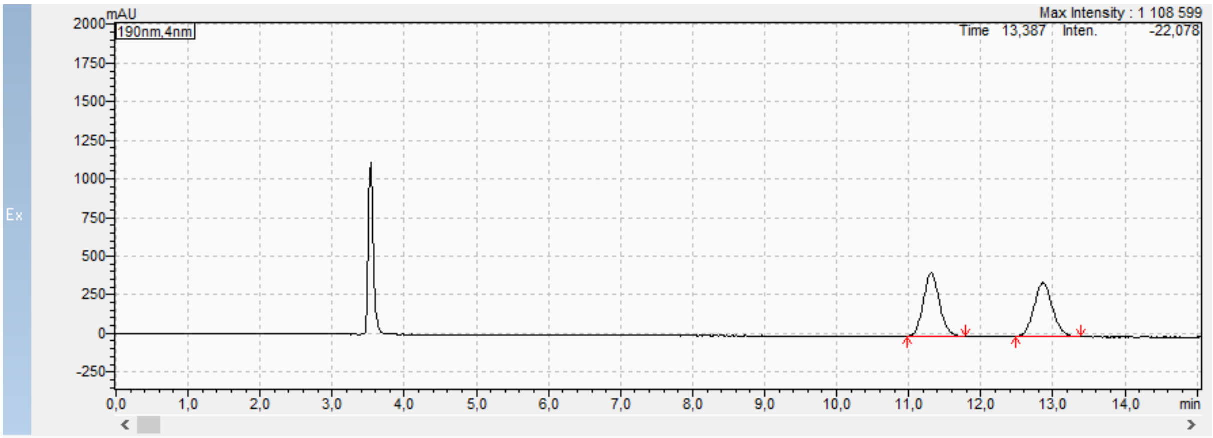

Results View - Peak Table

Peak Table | Compound | Group | Calibration Curve

Peak#	Ret. Time	Area	Height	Peak Start	Peak End	Mark	Conc.	Unit	Area%
1	11,309	6351706	412601	10,976	11,787	M	50,312		50,312
2	12,862	6272953	350128	12,480	13,387	M	49,688		49,688
Total		12624660	762729				100,000		100,000

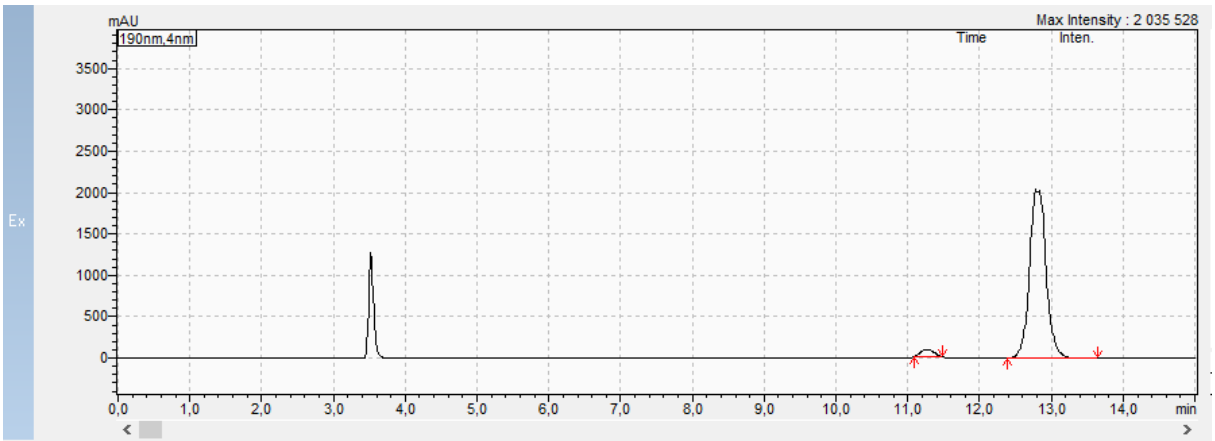

Results View - Peak Table

Peak Table | Compound | Group | Calibration Curve

Peak#	Ret. Time	Area	Height	Peak Start	Peak End	Mark	Conc.	Unit	Area%
1	11,267	1066191	84678	11,093	11,477	M	3,162		3,162
2	12,790	32648689	2036223	12,384	13,643	M	96,838		96,838
Total		33714880	2120901				100,000		100,000

for **2a**: *er* = 97:3 *er* (*ee* = 94%)

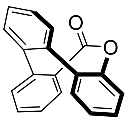

Conditions: Chiralpak IG column
mobile phase: *n*-heptane/propan-2-ol – 90:10
λ = 190 nm, V = 1.0 ml/min, t = 25 °C
for *ent*-**2a**: t_R = 11.3 min (major), t_R = 12.9 min (minor)

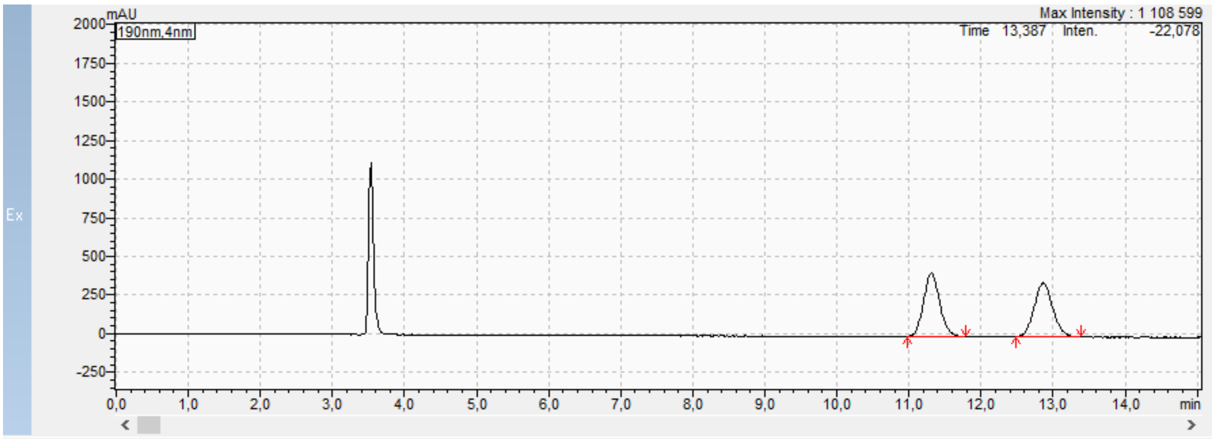

Results View - Peak Table

Peak Table | Compound | Group | Calibration Curve

Peak#	Ret. Time	Area	Height	Peak Start	Peak End	Mark	Conc.	Unit	Area%
1	11,309	6351706	412601	10,976	11,787	M	50,312		50,312
2	12,862	6272953	350128	12,480	13,387	M	49,688		49,688
Total		12624660	762729				100,000		100,000

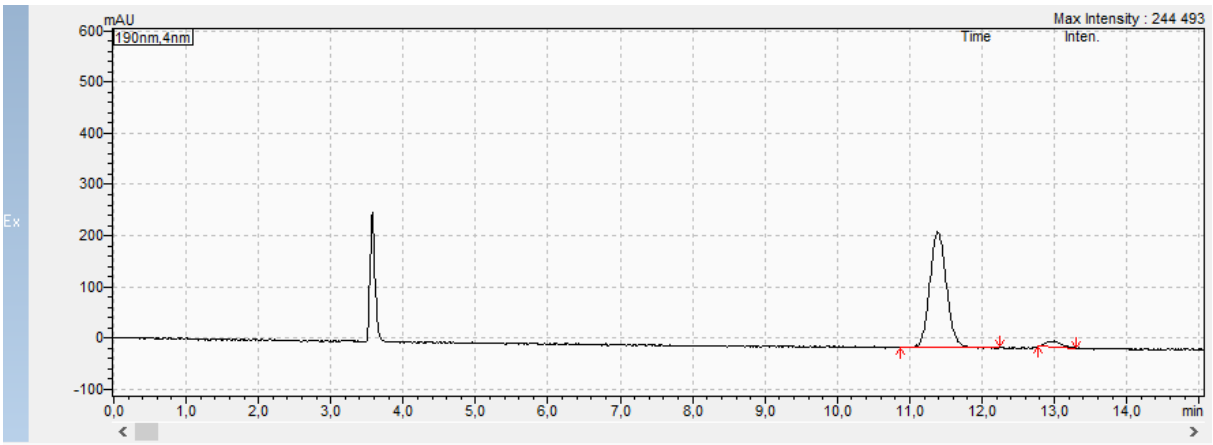

Results View - Peak Table

Peak Table | Compound | Group | Calibration Curve

Peak#	Ret. Time	Area	Height	Peak Start	Peak End	Mark	Conc.	Unit	Area%
1	11,385	3605978	226539	10,869	12,245	M	95,565		95,565
2	12,991	167336	11733	12,768	13,301	M	4,435		4,435
Total		3773314	238272				100,000		100,000

for *ent*-**2a**: *er* = 96:4 *er* (*ee* = 91%)

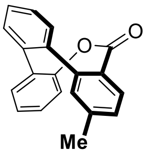

Conditions: Chiralpak IB column
mobile phase: *n*-heptane/propan-2-ol – 80:20
λ = 190 nm, V = 1.0 ml/min, t = 25 °C
for **2b**: t_R = 5.9 min (major), t_R = 6.9 min (minor)

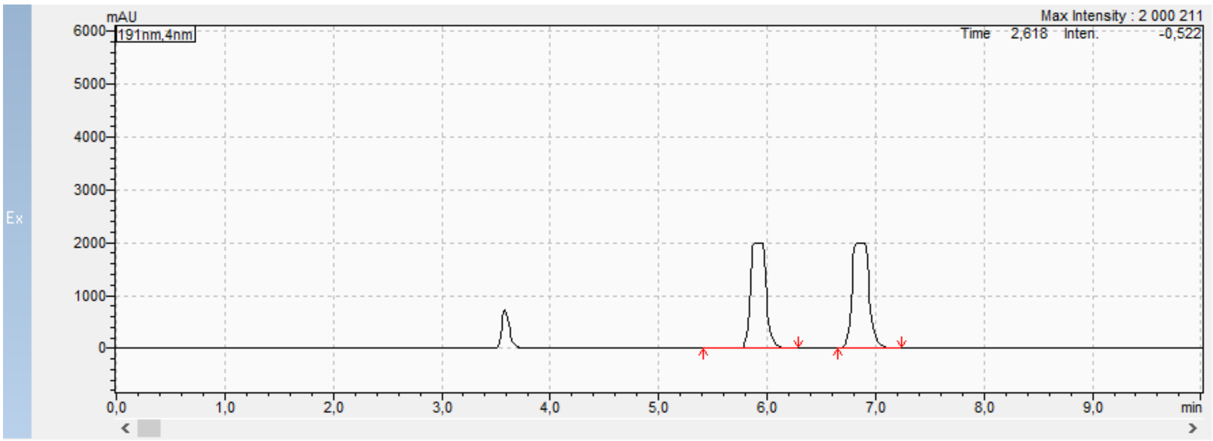

Results View - Peak Table

Peak Table | Compound | Group | Calibration Curve

Peak#	Ret. Time	Area	Height	Peak Start	Peak End	Mark	Conc.	Unit	Area%
1	5,947	19951863	1999035	5,408	6,283	M	47,909		47,909
2	6,885	21693616	1997173	6,656	7,243	M	52,091		52,091
Total		41645479	3996209				100,000		100,000

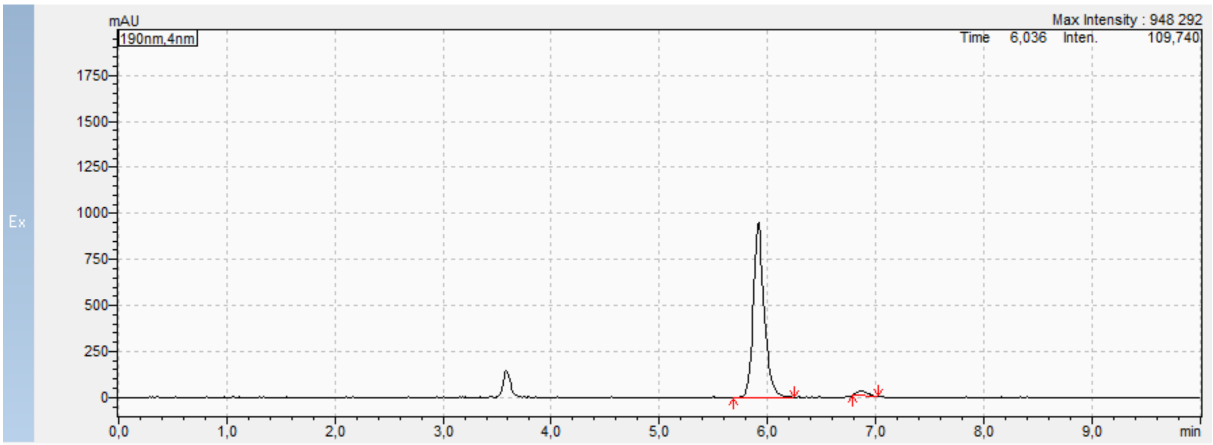

Results View - Peak Table

Peak Table | Compound | Group | Calibration Curve

Peak#	Ret. Time	Area	Height	Peak Start	Peak End	Mark	Conc.	Unit	Area%
1	5,916	6585617	947073	5,685	6,251	M	97,283		97,283
2	6,863	183917	25002	6,784	7,029	M	2,717		2,717
Total		6769533	972075				100,000		100,000

for **2b**: *er* = 97:3 *er* (*ee* = 95%)

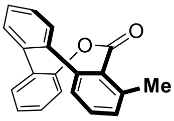

Conditions: Chiralpak IG column
mobile phase: *n*-heptane/propan-2-ol – 90:10
λ = 190 nm, V = 1.0 ml/min, t = 25 °C
for **2c**: t_R = 9.0 min (minor), t_R = 9.4 min (major)

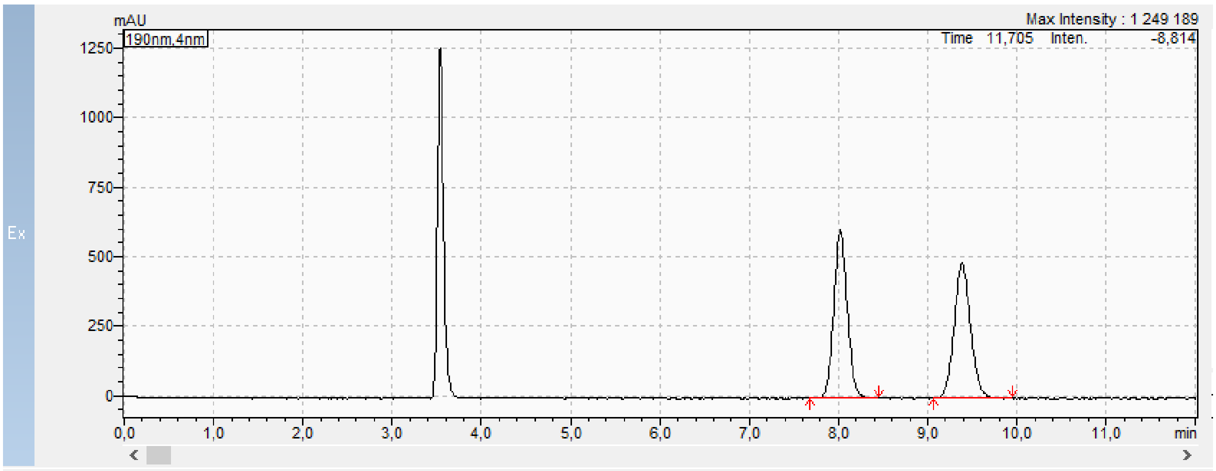

Results View - Peak Table

Peak Table | Compound | Group | Calibration Curve

Peak#	Ret. Time	Area	Height	Peak Start	Peak End	Mark	Conc.	Unit	Area%
1	8,020	6327061	606986	7,680	8,448	M	50,819		50,819
2	9,385	6123186	485857	9,067	9,952	M	49,181		49,181
Total		12450246	1092842				100,000		100,000

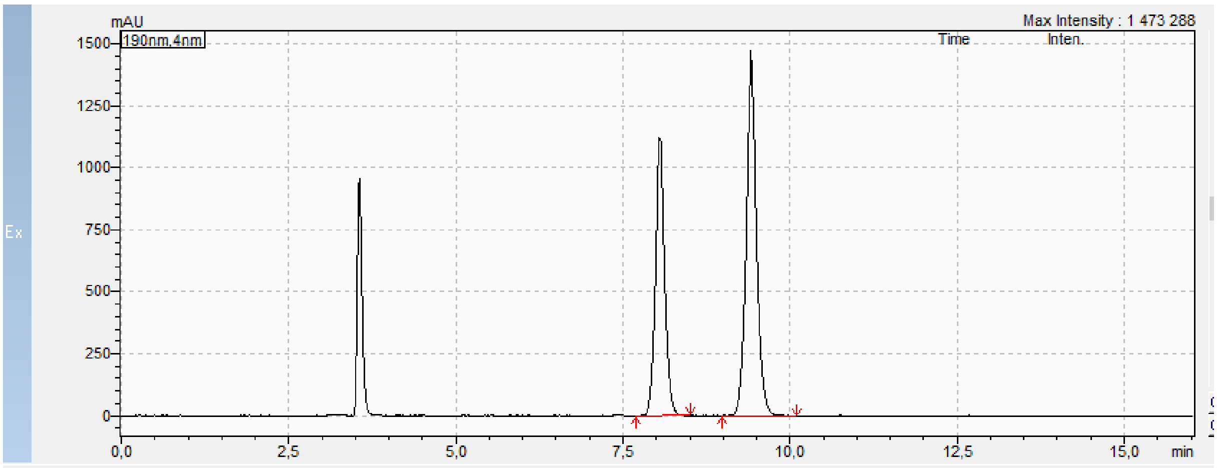

Results View - Peak Table

Peak Table | Compound | Group | Calibration Curve

Peak#	Ret. Time	Area	Height	Peak Start	Peak End	Mark	Conc.	Unit	Area%
1	8,047	10683319	1121084	7,691	8,512	M	41,139		41,139
2	9,420	15285626	1473592	8,981	10,101	M	58,861		58,861
Total		25968944	2594677				100,000		100,000

for **2c**: *er* = 59:41 *er* (*ee* = 18%)

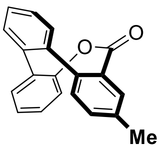

Conditions: Chiralpak IB column
mobile phase: *n*-heptane/propan-2-ol – 80:20
λ = 215 nm, V = 1.0 ml/min, t = 25 °C
for **2d**: t_R = 5.7 min (major), t_R = 7.0 min (minor)

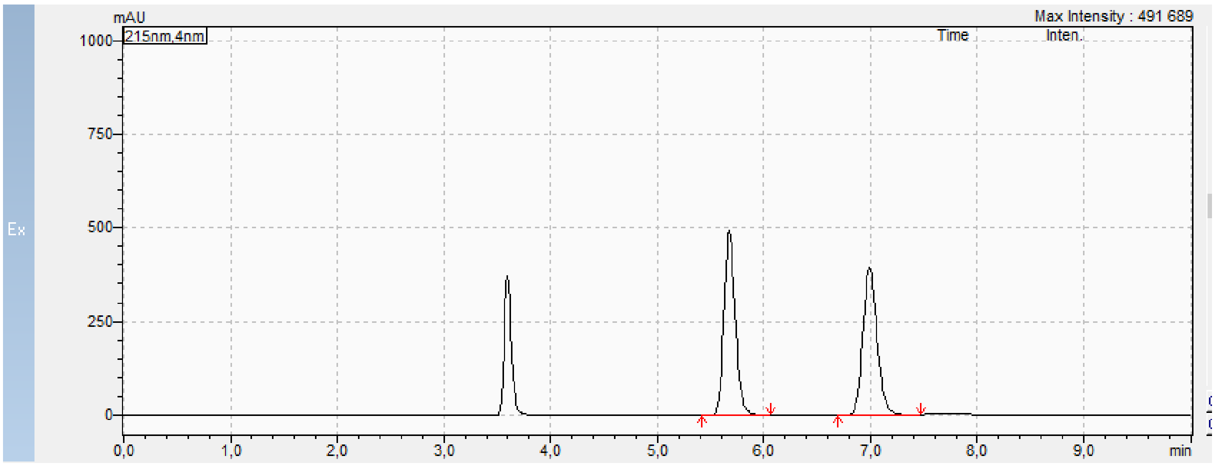

Results View - Peak Table

Peak Table | Compound | Group | Calibration Curve

Peak#	Ret. Time	Area	Height	Peak Start	Peak End	Mark	Conc.	Unit	Area%
1	5,675	3675251	491771	5,419	6,059	M	50,119		50,119
2	6,990	3657837	393243	6,688	7,467	M	49,881		49,881
Total		7333088	885014				100,000		100,000

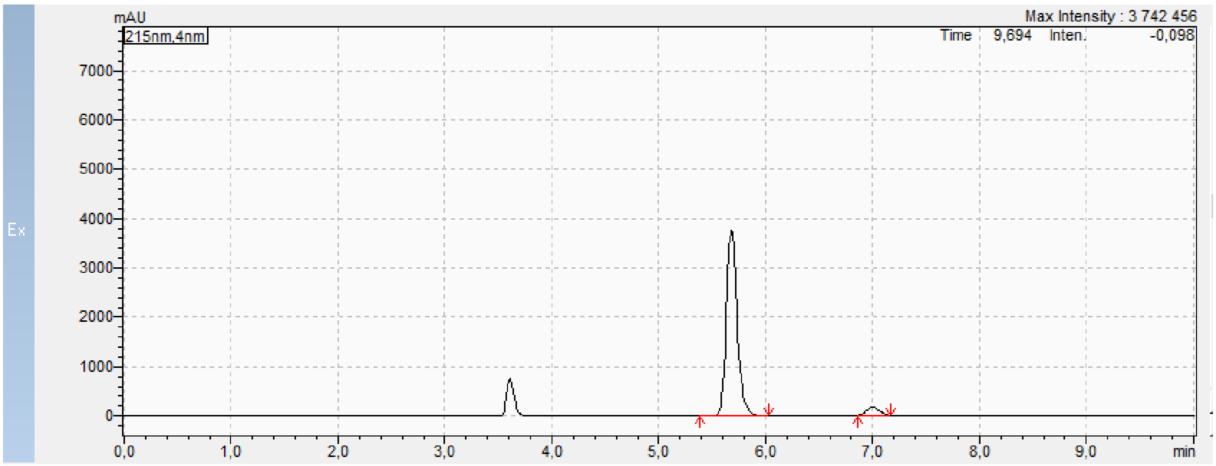

Results View - Peak Table

Peak Table | Compound | Group | Calibration Curve

Peak#	Ret. Time	Area	Height	Peak Start	Peak End	Mark	Conc.	Unit	Area%
1	5,688	26607326	3740607	5,387	6,027	M	94,855		94,855
2	7,000	1443221	168547	6,859	7,168	M	5,145		5,145
Total		28050547	3909154				100,000		100,000

for **2d**: *er* = 95:5 *er* (*ee* = 90%)

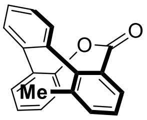

Conditions: Chiralpak IB column
mobile phase: *n*-heptane/propan-2-ol – 80:20
λ = 194 nm, V = 1.0 ml/min, t = 25 °C
for **2e**: t_R = 6.2 min (major), t_R = 7.2 min (minor)

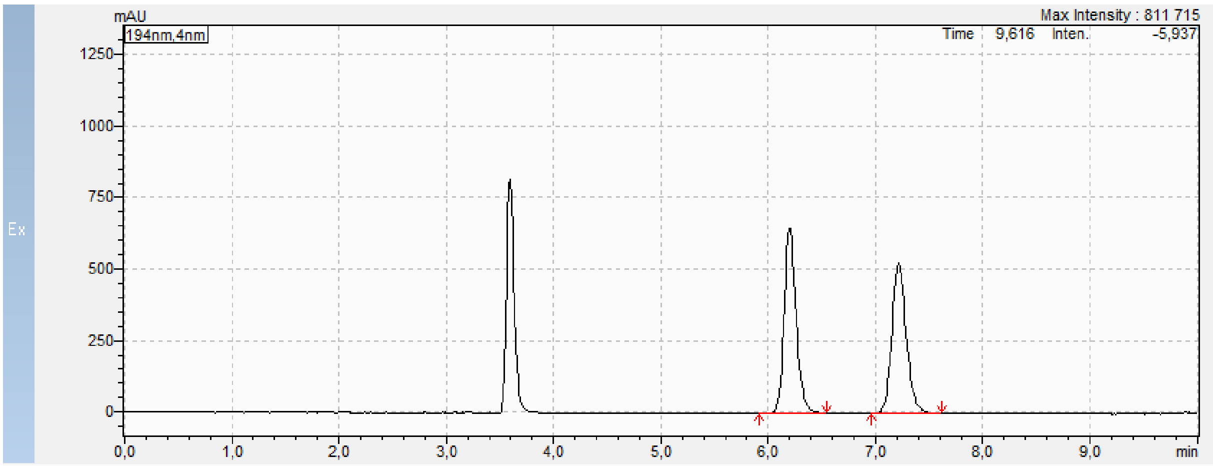

Results View - Peak Table

Peak Table | Compound | Group | Calibration Curve

Peak#	Ret. Time	Area	Height	Peak Start	Peak End	Mark	Conc.	Unit	Area%
1	6,202	4995802	644796	5,920	6,549	M	50,703		50,703
2	7,216	4857240	526386	6,965	7,616	M	49,297		49,297
Total		9853042	1171182				100,000		100,000

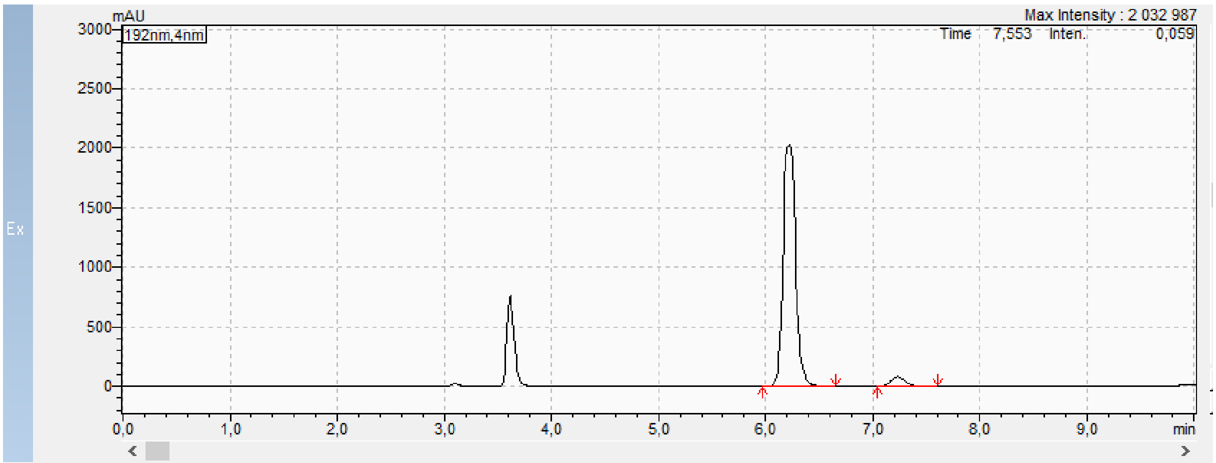

Results View - Peak Table

Peak Table | Compound | Group | Calibration Curve

Peak#	Ret. Time	Area	Height	Peak Start	Peak End	Mark	Conc.	Unit	Area%
1	6,225	16479873	2033354	5,973	6,656	M	95,558		95,558
2	7,232	765999	82240	7,040	7,605	M	4,442		4,442
Total		17245871	2115594				100,000		100,000

for **2e**: *er* = 96:4 *er* (*ee* = 91%)

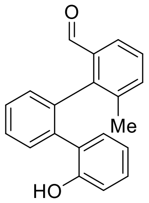

Conditions: Chiralpak IB column
mobile phase: *n*-heptane/propan-2-ol – 80:20
λ = 286 nm, V = 1.0 ml/min, t = 25 °C
for (-)-**1e**: t_R = 4.6 min (minor), t_R = 6.7 min (major)

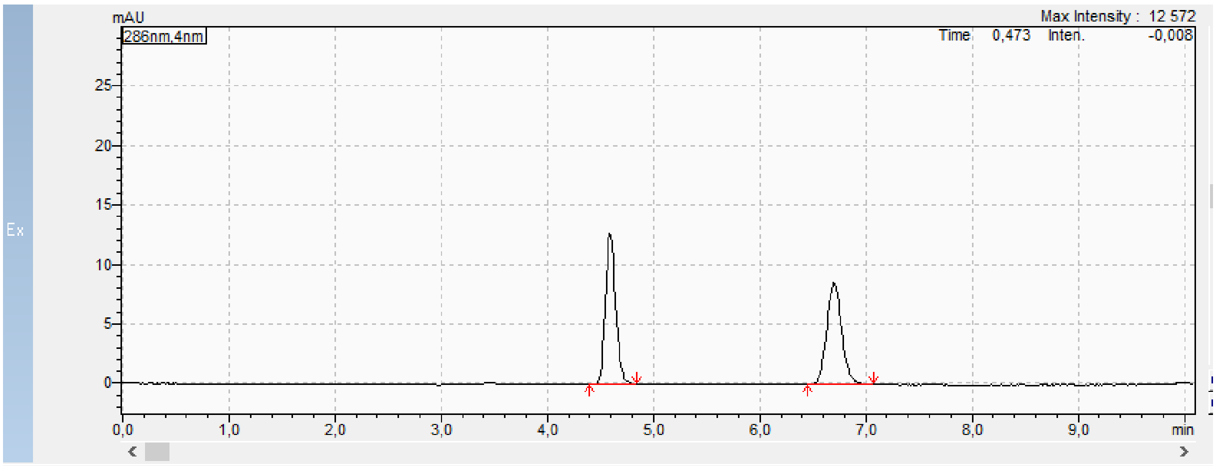

Results View - Peak Table

Peak Table | Compound | Group | Calibration Curve

Peak#	Ret. Time	Area	Height	Peak Start	Peak End	Mark	Conc.	Unit	Area%
1	4,591	84043	12659	4,395	4,843		49,925		49,925
2	6,699	84294	8539	6,453	7,072		50,075		50,075
Total		168338	21198				100,000		100,000

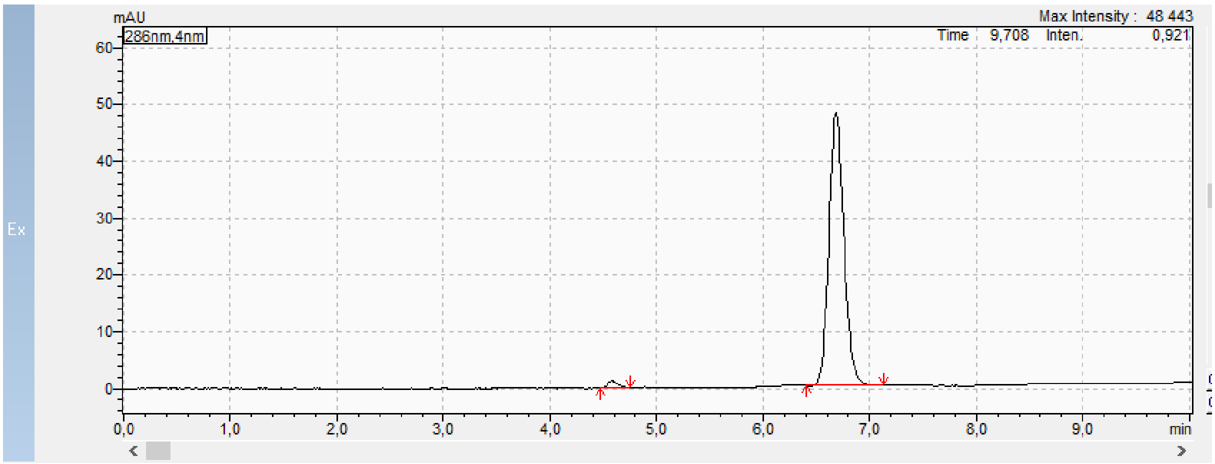

Results View - Peak Table

Peak Table | Compound | Group | Calibration Curve

Peak#	Ret. Time	Area	Height	Peak Start	Peak End	Mark	Conc.	Unit	Area%
1	4,582	8675	1206	4,469	4,757	M	1,801		1,801
2	6,684	473034	47905	6,400	7,136	M	98,199		98,199
Total		481709	49110				100,000		100,000

for (-)-**1e**: *er* = 98:2 *er* (*ee* = 96%)

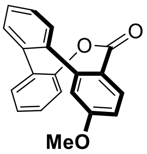

Conditions: Chiralpak IB column
mobile phase: *n*-heptane/propan-2-ol – 80:20
λ = 190 nm, V = 1.0 ml/min, t = 25 °C
for **2f**: t_R = 7.3 min (major), t_R = 9.1 min (minor)

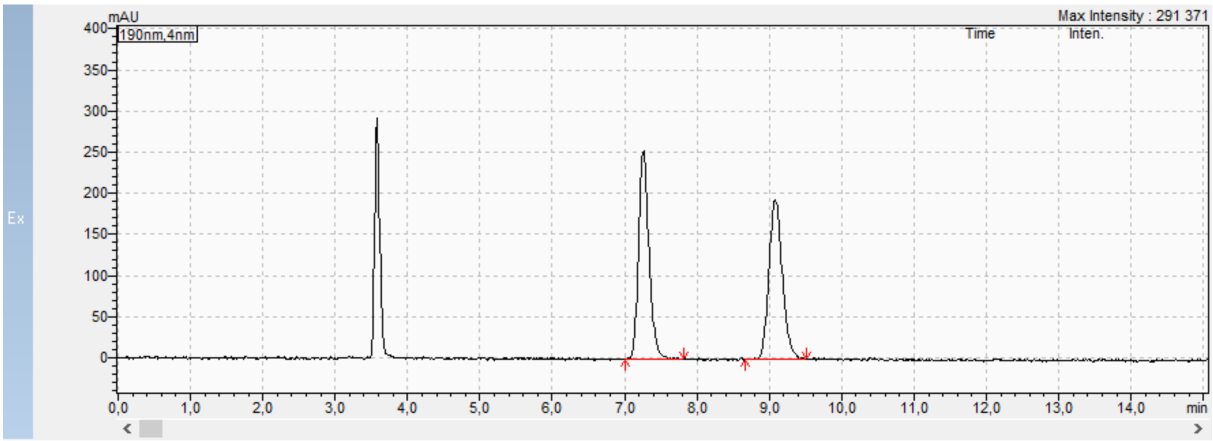

Results View - Peak Table

Peak Table | Compound | Group | Calibration Curve

Peak#	Ret. Time	Area	Height	Peak Start	Peak End	Mark	Conc.	Unit	Area%
1	7,260	2536146	252275	7,008	7,819	M	50,692		50,692
2	9,080	2466858	193711	8,661	9,515	M	49,308		49,308
Total		5003004	445985				100,000		100,000

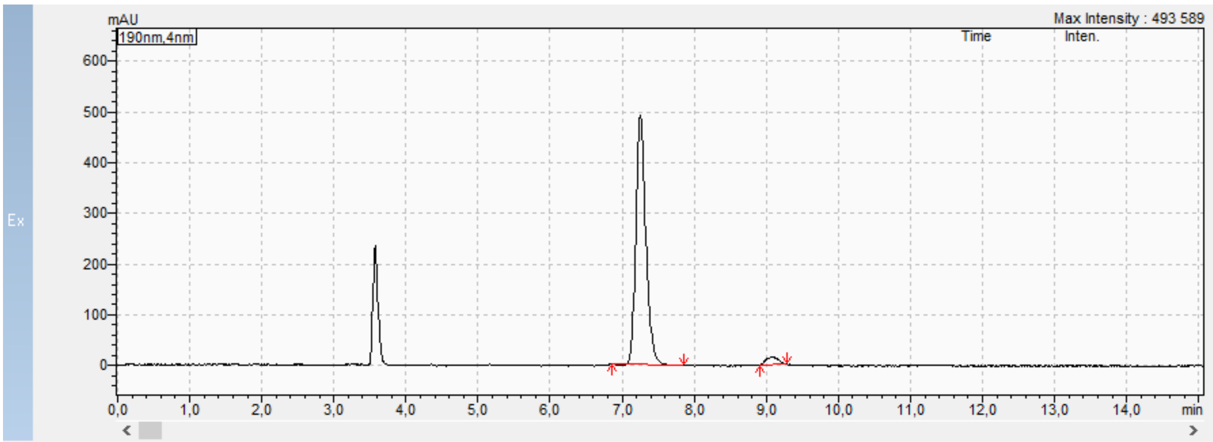

Results View - Peak Table

Peak Table | Compound | Group | Calibration Curve

Peak#	Ret. Time	Area	Height	Peak Start	Peak End	Mark	Conc.	Unit	Area%
1	7,253	4707166	491627	6,859	7,861	M	96,336		96,336
2	9,069	179054	15634	8,917	9,291	M	3,664		3,664
Total		4886220	507261				100,000		100,000

for **2f**: *er* = 96:4 *er* (*ee* = 93%)

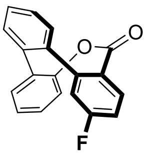

Conditions: Chiralpak IB column
mobile phase: *n*-heptane/propan-2-ol – 80:20
λ = 190 nm, V = 1.0 ml/min, t = 25 °C
for **2g**: t_R = 5.9 min (major), t_R = 7.1 min (minor)

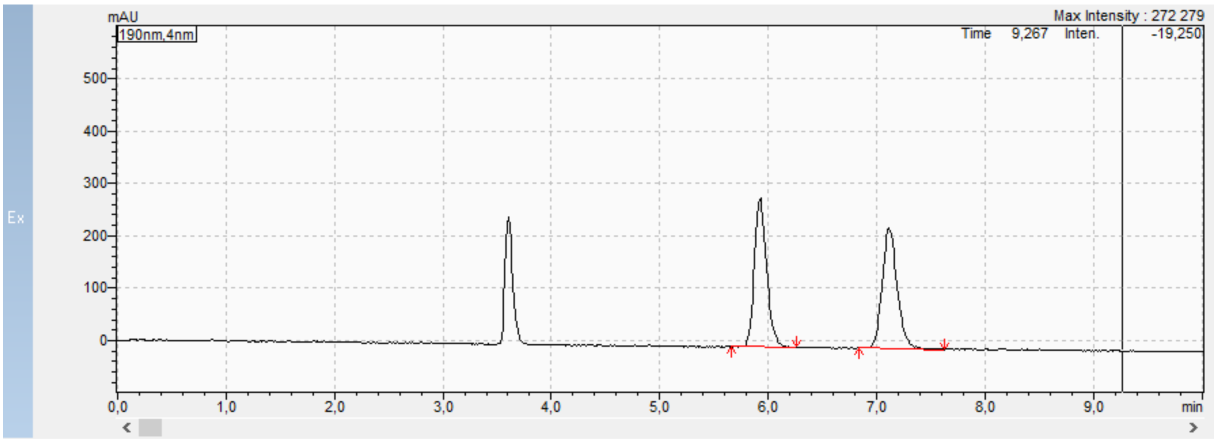

Results View - Peak Table

Peak Table | Compound | Group | Calibration Curve

Peak#	Ret. Time	Area	Height	Peak Start	Peak End	Mark	Conc.	Unit	Area%
1	5,926	2238224	284638	5,664	6,261	M	50,517		50,517
2	7,115	2192381	230057	6,837	7,627	M	49,483		49,483
Total		4430605	514694				100,000		100,000

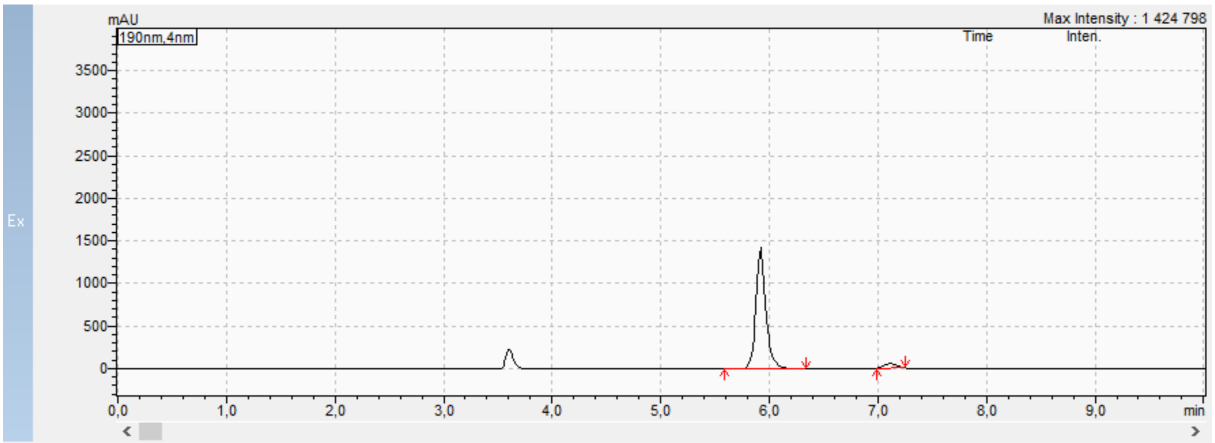

Results View - Peak Table

Peak Table | Compound | Group | Calibration Curve

Peak#	Ret. Time	Area	Height	Peak Start	Peak End	Mark	Conc.	Unit	Area%
1	5,920	9092707	1426518	5,589	6,336	M	95,680		95,680
2	7,112	410531	51277	6,987	7,253	M	4,320		4,320
Total		9503238	1477794				100,000		100,000

for **2g**: *er* = 96:4 *er* (*ee* = 91%)

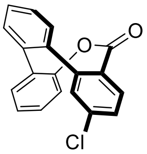

Conditions: Chiralpak IB column
mobile phase: *n*-heptane/propan-2-ol – 80:20
λ = 190 nm, V = 1.0 ml/min, t = 25 °C
for **2h**: t_R = 5.7 min (major), t_R = 7.1 min (minor)

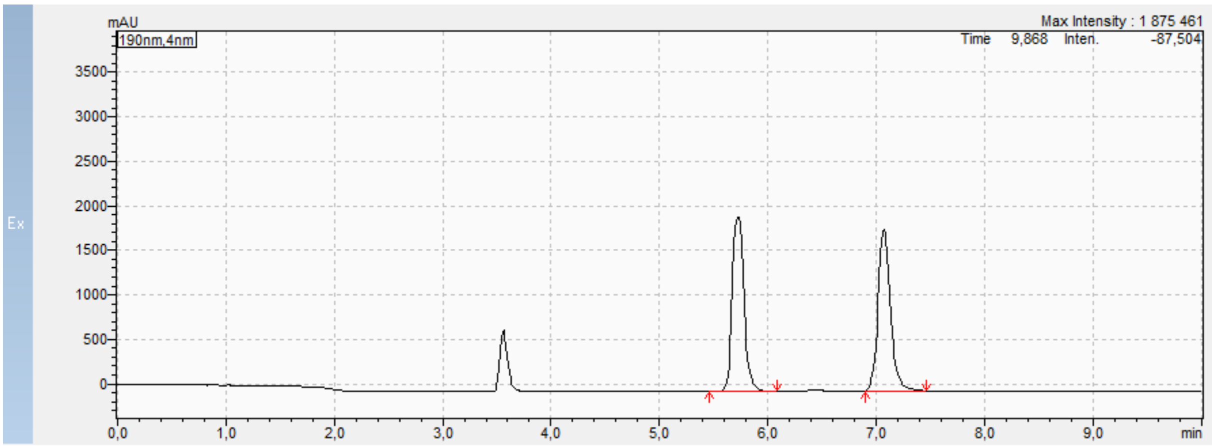

Results View - Peak Table

Peak Table | Compound | Group | Calibration Curve

Peak#	Ret. Time	Area	Height	Peak Start	Peak End	Mark	Conc.	Unit	Area%
1	5,734	14811572	1960761	5,461	6,091	M	50,486		50,486
2	7,071	14526411	1813612	6,901	7,467	M	49,514		49,514
Total		29337982	3774374				100,000		100,000

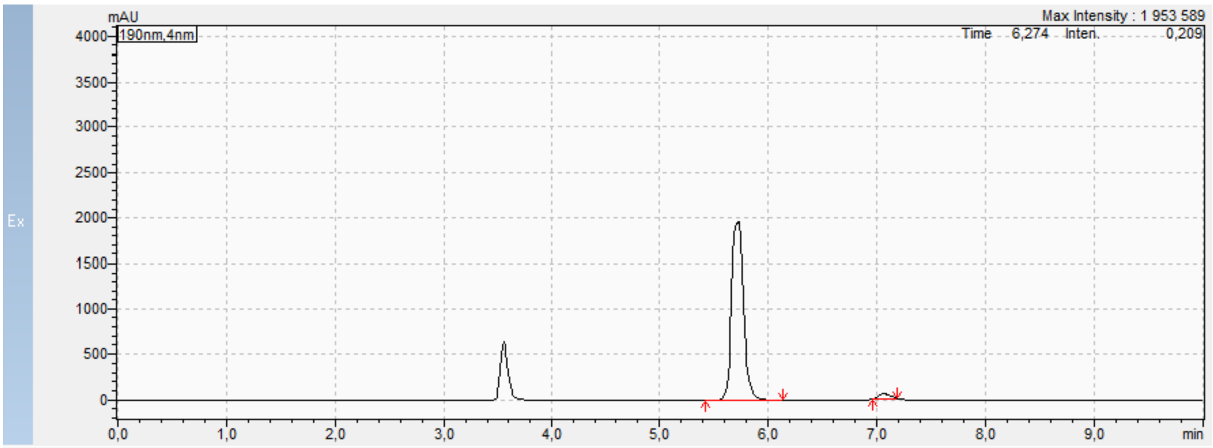

Results View - Peak Table

Peak Table | Compound | Group | Calibration Curve

Peak#	Ret. Time	Area	Height	Peak Start	Peak End	Mark	Conc.	Unit	Area%
1	5,733	14931217	1954592	5,419	6,133	M	97,316		97,316
2	7,078	411817	54955	6,965	7,189	M	2,684		2,684
Total		15343034	2009547				100,000		100,000

for **2h**: *er* = 97:3 *er* (*ee* = 95%)

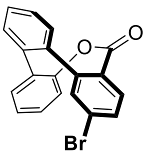

Conditions: Chiralpak IB column
mobile phase: *n*-heptane/propan-2-ol – 80:20
λ = 190 nm, V = 1.0 ml/min, t = 25 °C
for **2i**: t_R = 5.9 min (minor), t_R = 7.4 min (major)

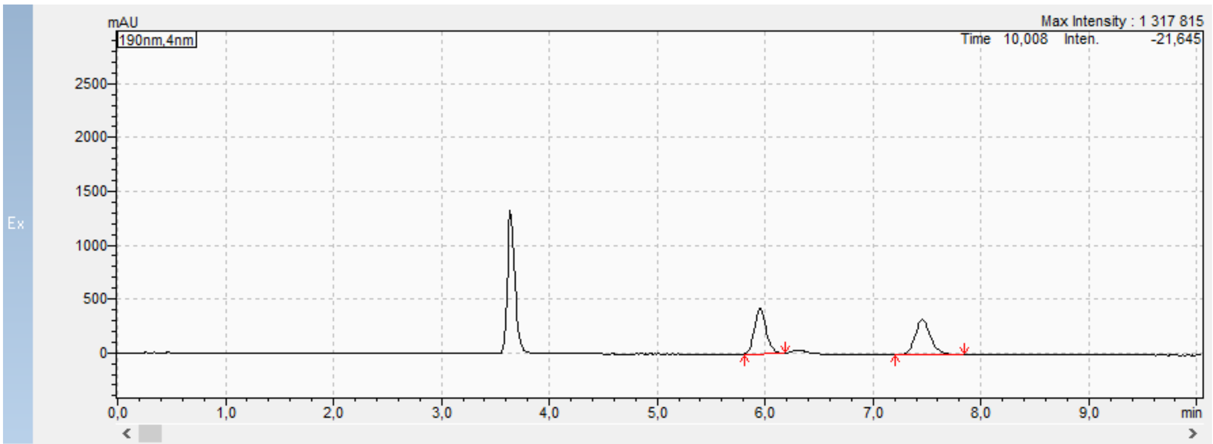

Results View - Peak Table

Peak Table | Compound | Group | Calibration Curve

Peak#	Ret. Time	Area	Height	Peak Start	Peak End	Mark	Conc.	Unit	Area%
1	5,952	3187422	426183	5,803	6,187	M	49,763		49,763
2	7,455	3217841	325475	7,200	7,840	M	50,237		50,237
Total		6405263	751657				100,000		100,000

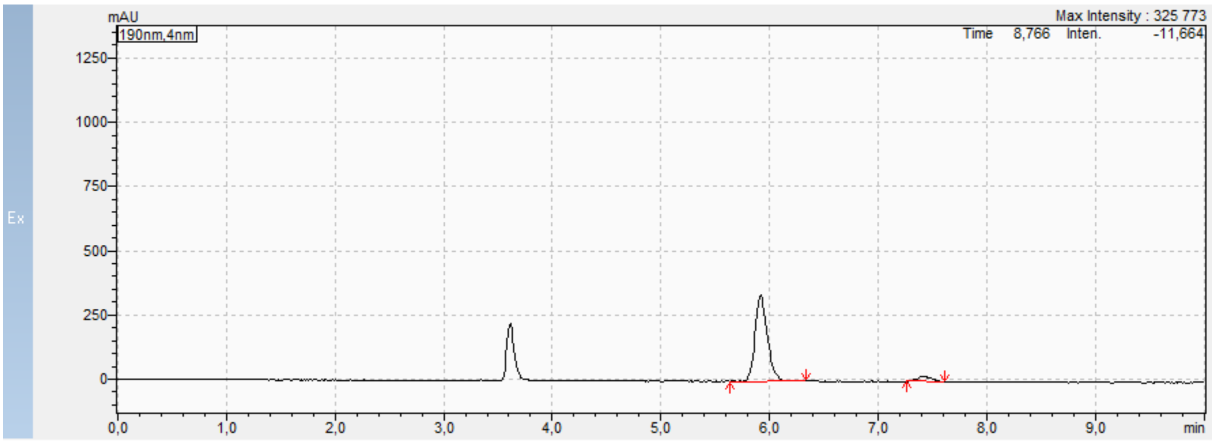

Results View - Peak Table

Peak Table | Compound | Group | Calibration Curve

Peak#	Ret. Time	Area	Height	Peak Start	Peak End	Mark	Conc.	Unit	Area%
1	5,921	2617232	333322	5,632	6,336	M	93,620		93,620
2	7,431	178361	19112	7,264	7,616	M	6,380		6,380
Total		2795593	352434				100,000		100,000

for **2i**: *er* = 94:6 *er* (*ee* = 87%)

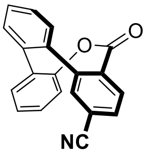

Conditions: Chiralpak IB column
mobile phase: *n*-heptane/propan-2-ol – 80:20
λ = 190 nm, V = 1.0 ml/min, t = 25 °C
for **2j**: t_R = 10.2 min (minor), t_R = 13.1 min (major)

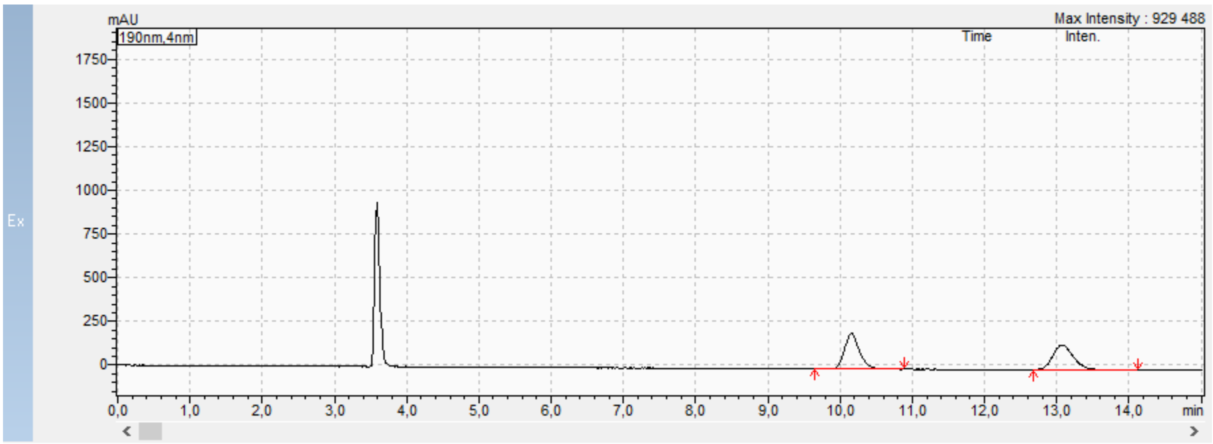

Results View - Peak Table

Peak Table | Compound | Group | Calibration Curve

Peak#	Ret. Time	Area	Height	Peak Start	Peak End	Mark	Conc.	Unit	Area%
1	10,163	2957787	202466	9,643	10,891	M	51,157		51,157
2	13,079	2823942	141741	12,672	14,123	M	48,843		48,843
Total		5781730	344207				100,000		100,000

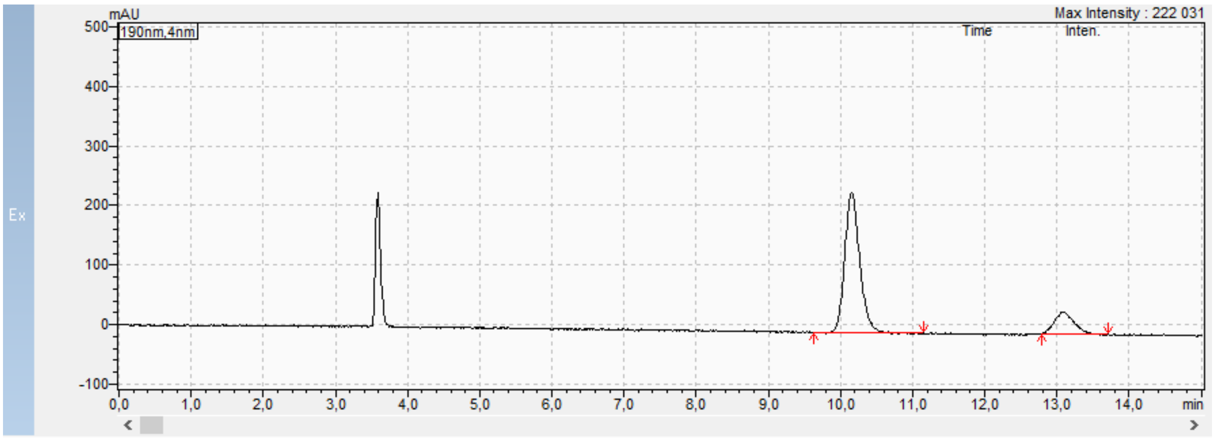

Results View - Peak Table

Peak Table | Compound | Group | Calibration Curve

Peak#	Ret. Time	Area	Height	Peak Start	Peak End	Mark	Conc.	Unit	Area%
1	10,153	3391604	234613	9,621	11,157	M	83,405		83,405
2	13,105	674817	36463	12,789	13,707	M	16,595		16,595
Total		4066421	271076				100,000		100,000

for **2j**: *er* = 83:17 *er* (*ee* = 67%)

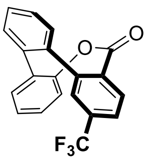

Conditions: Chiralpak IB column
mobile phase: *n*-heptane/propan-2-ol – 80:20
λ = 190 nm, V = 1.0 ml/min, t = 25 °C
for **2k**: t_R = 5.2 min (major), t_R = 6.9 min (minor)

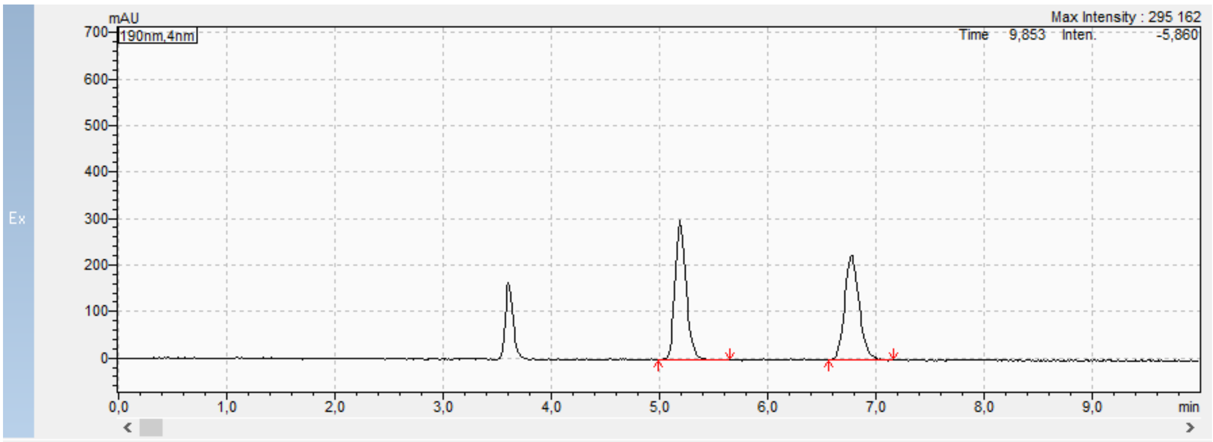

Results View - Peak Table

Peak Table | Compound | Group | Calibration Curve

Peak#	Ret. Time	Area	Height	Peak Start	Peak End	Mark	Conc.	Unit	Area%
1	5,191	2124501	298824	4,992	5,653	M	50,429		50,429
2	6,776	2088346	225064	6,571	7,168	M	49,571		49,571
Total		4212847	523888				100,000		100,000

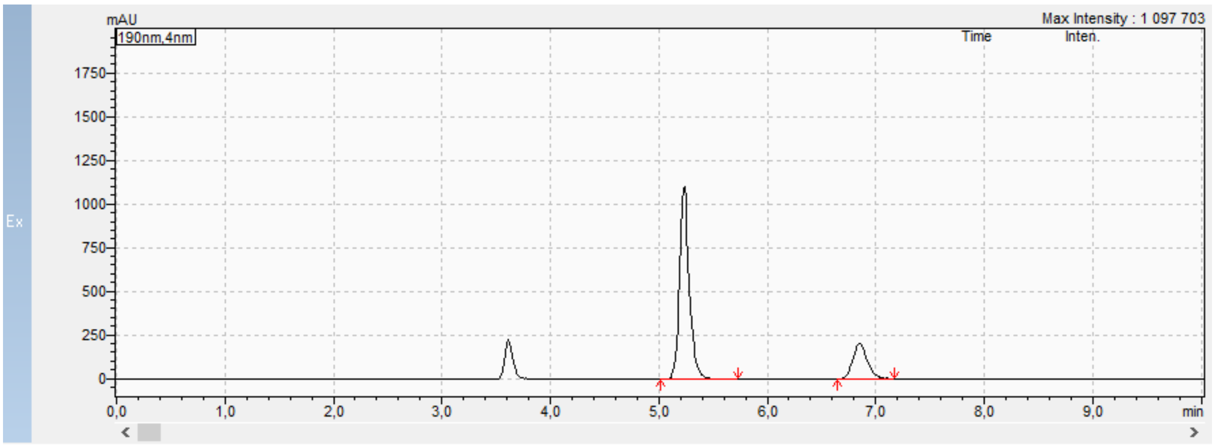

Results View - Peak Table

Peak Table | Compound | Group | Calibration Curve

Peak#	Ret. Time	Area	Height	Peak Start	Peak End	Mark	Conc.	Unit	Area%
1	5,233	6821961	1100436	5,013	5,728	M	78,362		78,362
2	6,851	1883743	201448	6,645	7,168	M	21,638		21,638
Total		8705705	1301884				100,000		100,000

for **2k**: *er* = 78:22 *er* (*ee* = 57%)

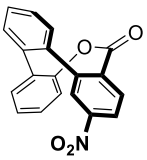

Conditions: Chiralpak IB column
mobile phase: *n*-heptane/propan-2-ol – 80:20
λ = 190 nm, V = 1.0 ml/min, t = 25 °C
for **2l**: t_R = 10.1 min (major), t_R = 12.4 min (minor)

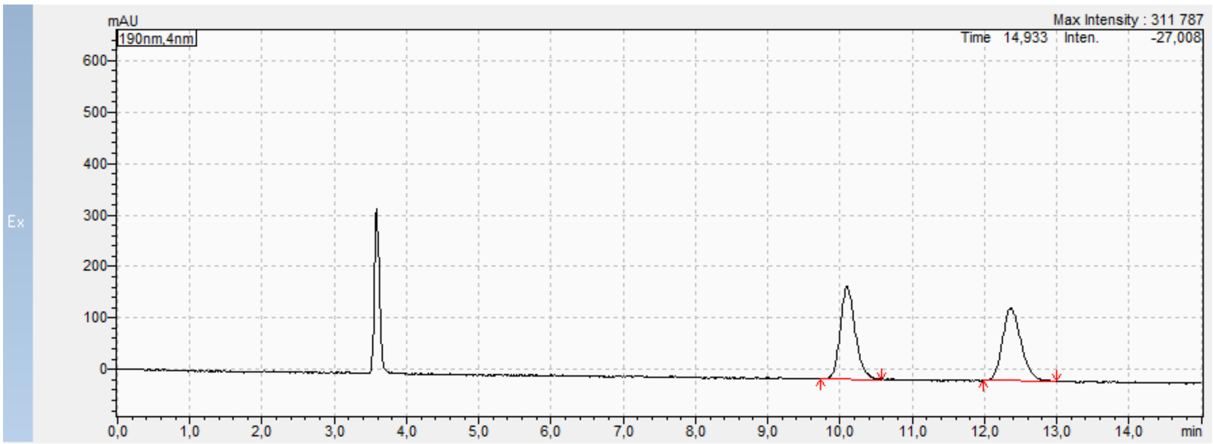

Results View - Peak Table

Peak Table | Compound | Group | Calibration Curve

Peak#	Ret. Time	Area	Height	Peak Start	Peak End	Mark	Conc.	Unit	Area%
1	10,098	2580138	179337	9,728	10,571	M	50,465		50,465
2	12,364	2532615	141069	11,989	13,003	M	49,535		49,535
Total		5112753	320405				100,000		100,000

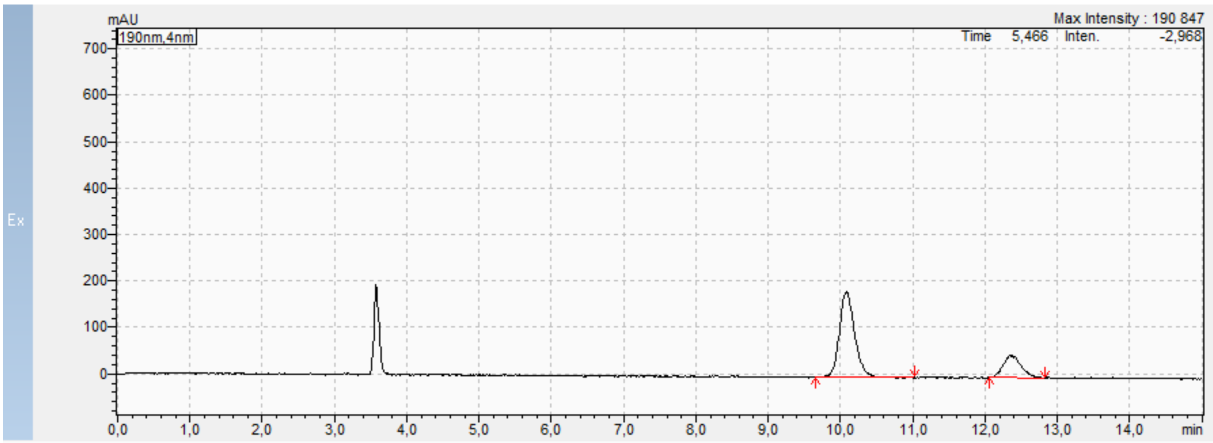

Results View - Peak Table

Peak Table | Compound | Group | Calibration Curve

Peak#	Ret. Time	Area	Height	Peak Start	Peak End	Mark	Conc.	Unit	Area%
1	10,084	2652382	182848	9,653	11,029	M	76,508		76,508
2	12,363	814423	46687	12,053	12,821	M	23,492		23,492
Total		3466806	229535				100,000		100,000

for **2l**: *er* = 77:23 *er* (*ee* = 53%)

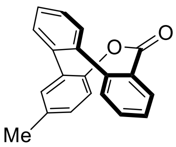

Conditions: Chiralpak IB column
mobile phase: *n*-heptane/propan-2-ol – 80:20
λ = 190 nm, V = 1.0 ml/min, t = 25 °C
for **2m**: t_R = 6.1 min (major), t_R = 6.7 min (minor)

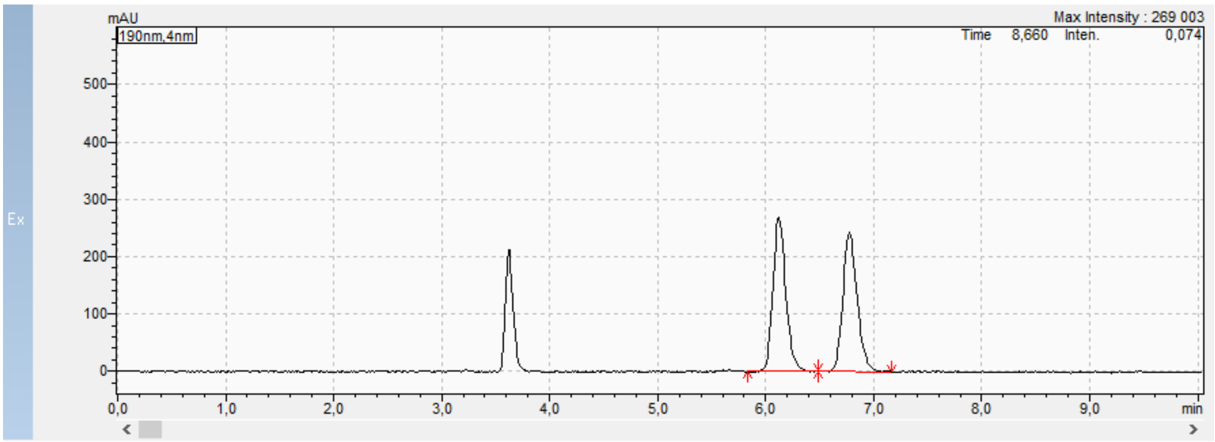

Results View - Peak Table

Peak Table | Compound | Group | Calibration Curve

Peak#	Ret. Time	Area	Height	Peak Start	Peak End	Mark	Conc.	Unit	Area%
1	6,120	2223791	269314	5,835	6,485	M	49,876		49,876
2	6,775	2234847	242875	6,485	7,168	M	50,124		50,124
Total		4458638	512189				100,000		100,000

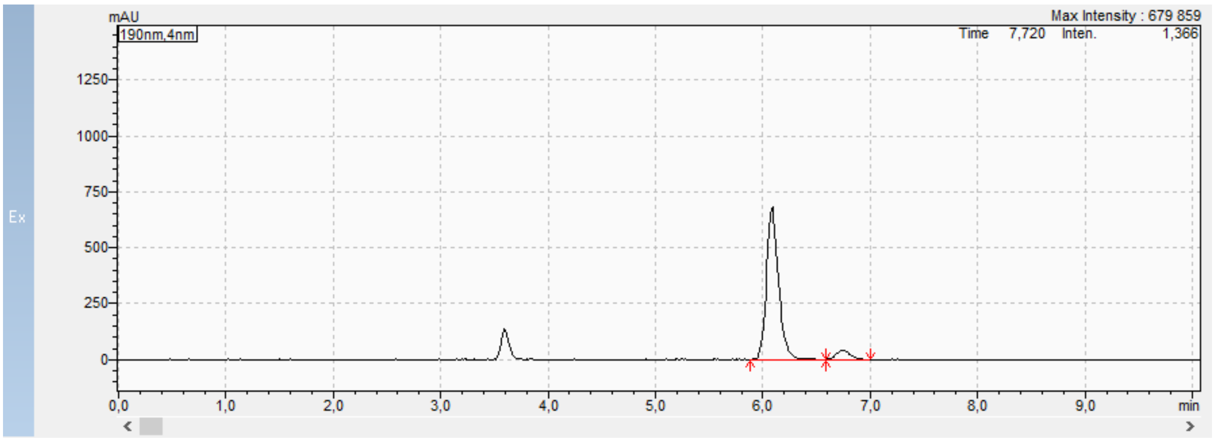

Results View - Peak Table

Peak Table | Compound | Group | Calibration Curve

Peak#	Ret. Time	Area	Height	Peak Start	Peak End	Mark	Conc.	Unit	Area%
1	6,087	5303554	678887	5,877	6,592	M	93,488		93,488
2	6,736	369432	39638	6,592	7,008	M	6,512		6,512
Total		5672986	718526				100,000		100,000

for **2m**: *er* = 93:7 *er* (*ee* = 87%)

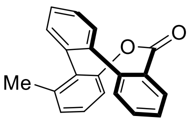

Conditions: Chiralpak IB column
mobile phase: *n*-heptane/propan-2-ol – 80:20
λ = 204 nm, V = 1.0 ml/min, t = 25 °C
for **2n**: t_R = 6.1 min (major), t_R = 7.1 min (minor)

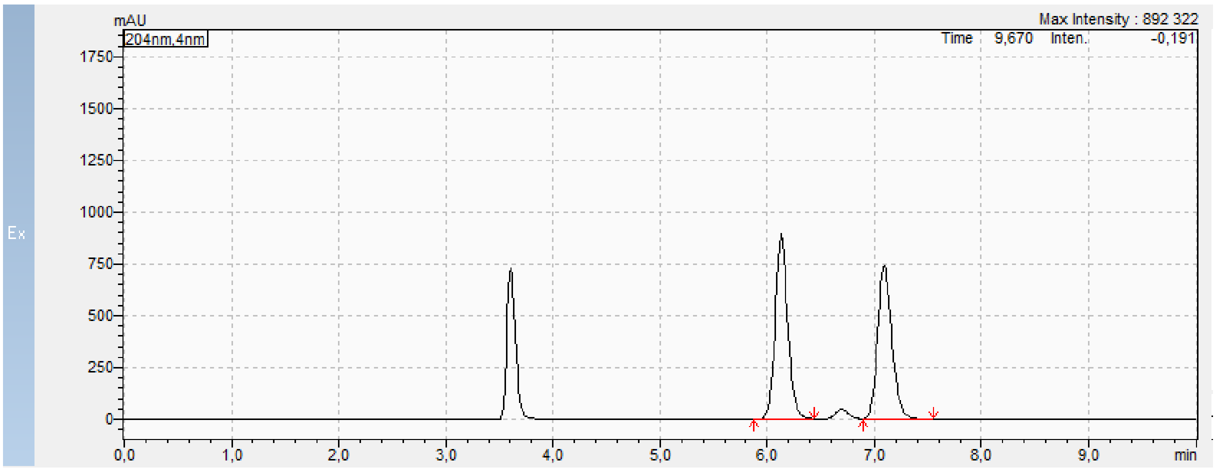

Results View - Peak Table

Peak Table | Compound | Group | Calibration Curve

Peak#	Ret. Time	Area	Height	Peak Start	Peak End	Mark	Conc.	Unit	Area%
1	6,129	7324271	890985	5,877	6,443	M	51,669		51,669
2	7,092	6850985	737259	6,891	7,552	M	48,331		48,331
Total		14175257	1628244				100,000		100,000

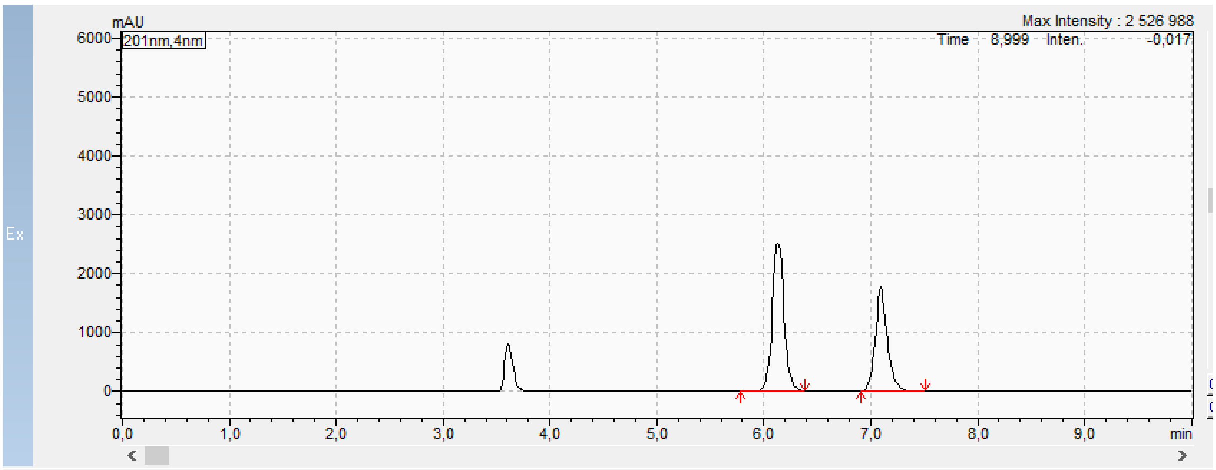

Results View - Peak Table

Peak Table | Compound | Group | Calibration Curve

Peak#	Ret. Time	Area	Height	Peak Start	Peak End	Mark	Conc.	Unit	Area%
1	6,132	19418521	2520276	5,781	6,389	M	58,011		58,011
2	7,094	14055481	1785729	6,901	7,509	M	41,989		41,989
Total		33474002	4306006				100,000		100,000

for **2n**: *er* = 58:42 *er* (*ee* = 16%)

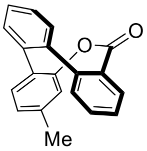

Conditions: Chiralpak IB column
mobile phase: *n*-heptane/propan-2-ol – 80:20
λ = 210 nm, V = 1.0 ml/min, t = 25 °C
for **2o**: t_R = 6.2 min (major), t_R = 7.4 min (minor)

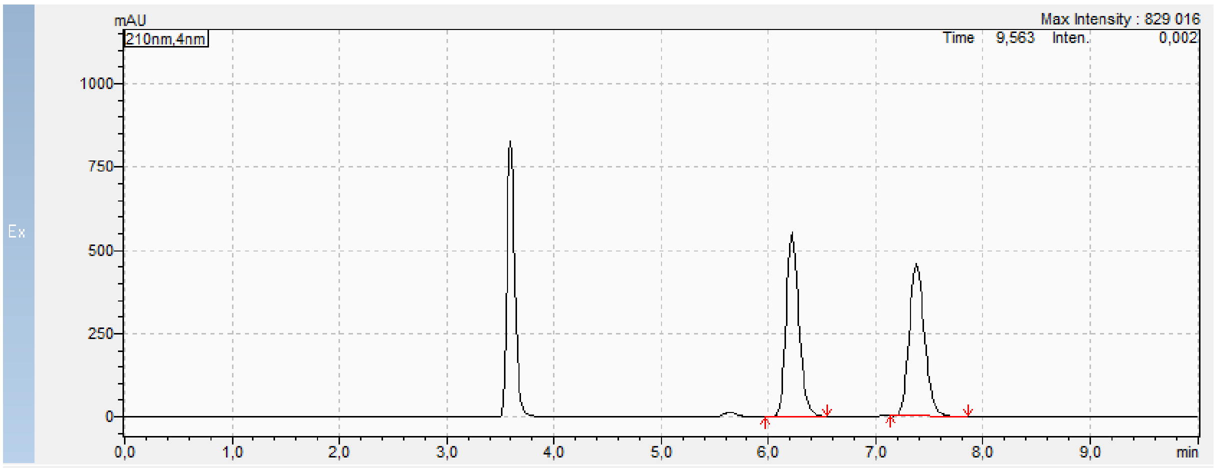

Results View - Peak Table

Peak Table | Compound | Group | Calibration Curve

Peak#	Ret. Time	Area	Height	Peak Start	Peak End	Mark	Conc.	Unit	Area%
1	6,220	4606891	551195	5,973	6,549	M	50,323		50,323
2	7,377	4547826	455579	7,136	7,861	M	49,677		49,677
Total		9154717	1006774				100,000		100,000

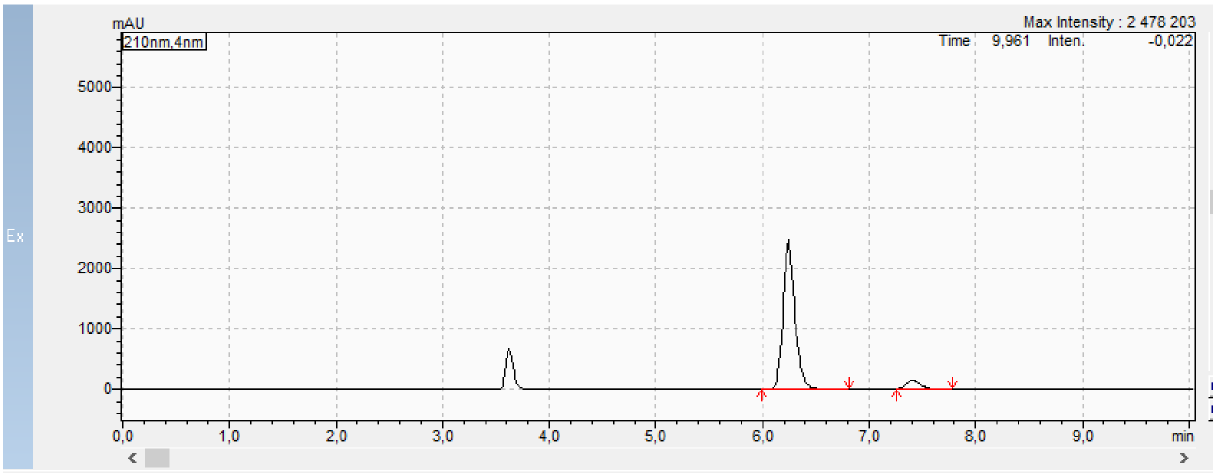

Results View - Peak Table

Peak Table | Compound | Group | Calibration Curve

Peak#	Ret. Time	Area	Height	Peak Start	Peak End	Mark	Conc.	Unit	Area%
1	6,242	18703644	2477153	5,995	6,816	M	93,405		93,405
2	7,410	1320542	137501	7,253	7,776	M	6,595		6,595
Total		20024186	2614654				100,000		100,000

for **2o**: *er* = 93:7 *er* (*ee* = 87%)

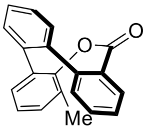

Conditions: Chiralpak IB column
mobile phase: *n*-heptane/propan-2-ol – 80:20
λ = 190 nm, V = 1.0 ml/min, t = 25 °C
for **2p**: t_R = 5.6 min (major), t_R = 6.0 min (minor)

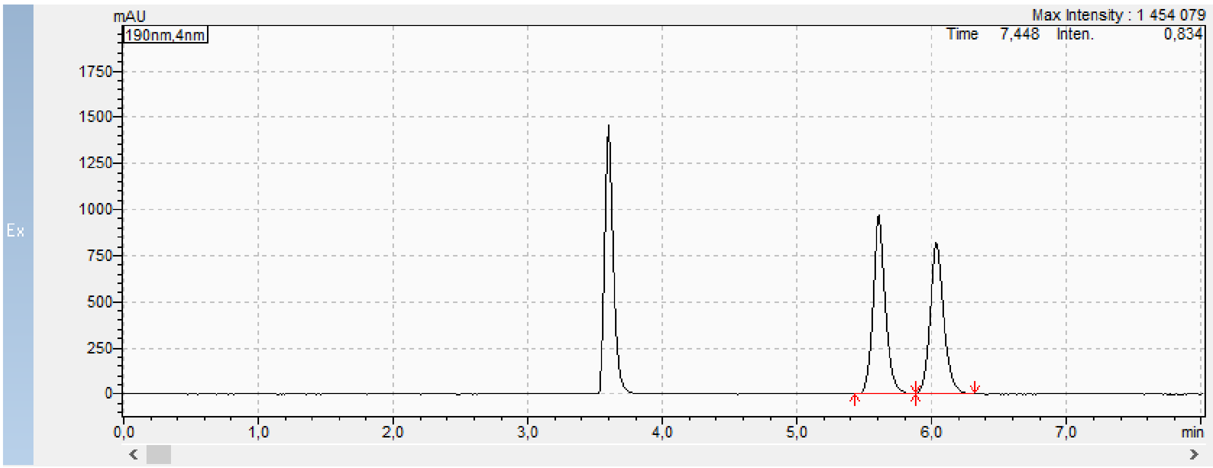

Results View - Peak Table

Peak Table | Compound | Group | Calibration Curve

Peak#	Ret. Time	Area	Height	Peak Start	Peak End	Mark	Conc.	Unit	Area%
1	5,606	5968459	965988	5,429	5,877	M	51,289		51,289
2	6,031	5668484	814062	5,877	6,315	M	48,711		48,711
Total		11636943	1780049				100,000		100,000

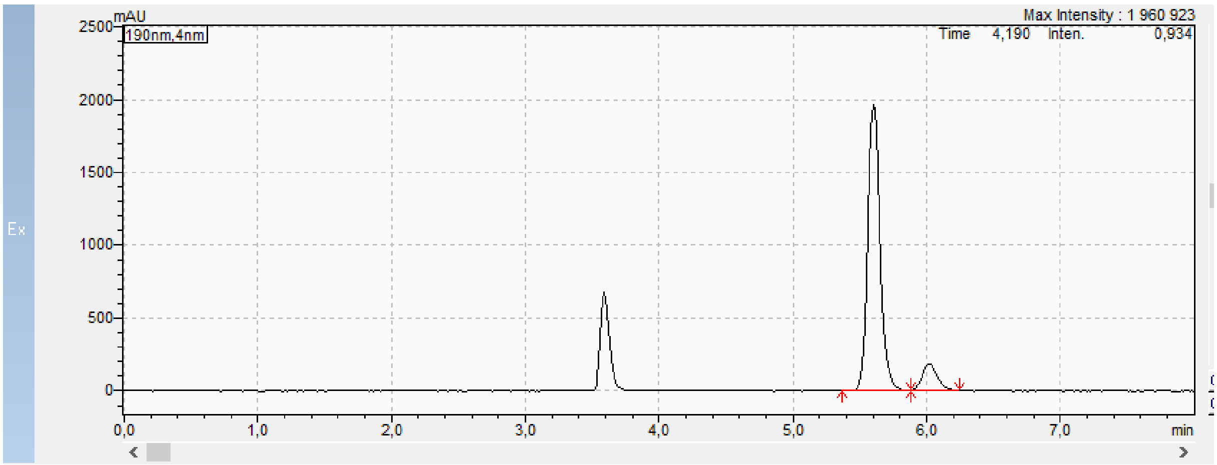

Results View - Peak Table

Peak Table | Compound | Group | Calibration Curve

Peak#	Ret. Time	Area	Height	Peak Start	Peak End	Mark	Conc.	Unit	Area%
1	5,602	12589825	1958156	5,365	5,888	M	89,894		89,894
2	6,026	1415298	182787	5,888	6,251	M	10,106		10,106
Total		14005124	2140943				100,000		100,000

for **2p**: *er* = 90:10 *er* (*ee* = 80%)

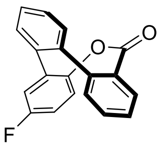

Conditions: Chiralpak IG column
mobile phase: *n*-heptane/propan-2-ol – 90:10
λ = 190 nm, V = 1.0 ml/min, t = 25 °C
for **2q**: t_R = 9.1 min (minor), t_R = 10.6 min (major)

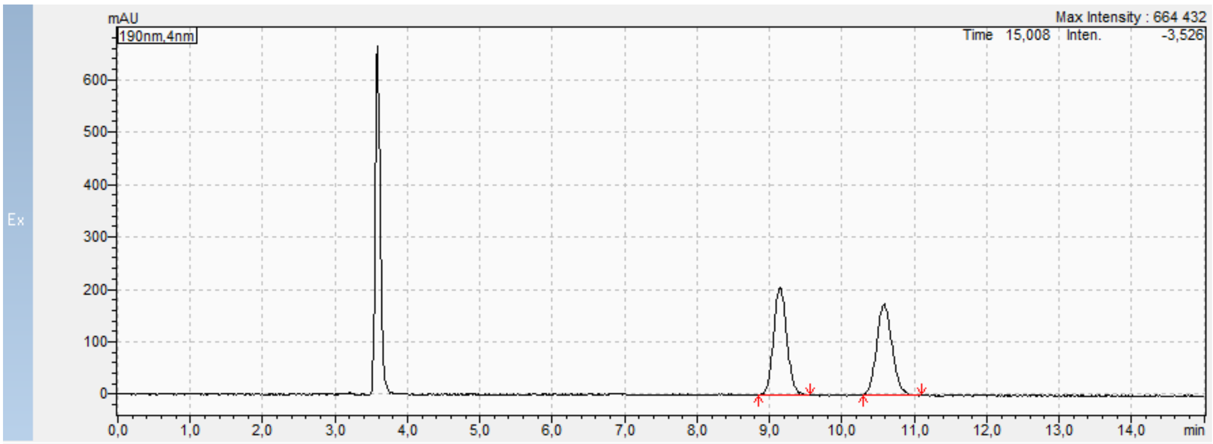

Results View - Peak Table

Peak Table | Compound | Group | Calibration Curve

Peak#	Ret. Time	Area	Height	Peak Start	Peak End	Mark	Conc.	Unit	Area%
1	9,142	2631177	206608	8,843	9,557	M	50,877		50,877
2	10,588	2540443	172885	10,293	11,104	M	49,123		49,123
Total		5171620	379493				100,000		100,000

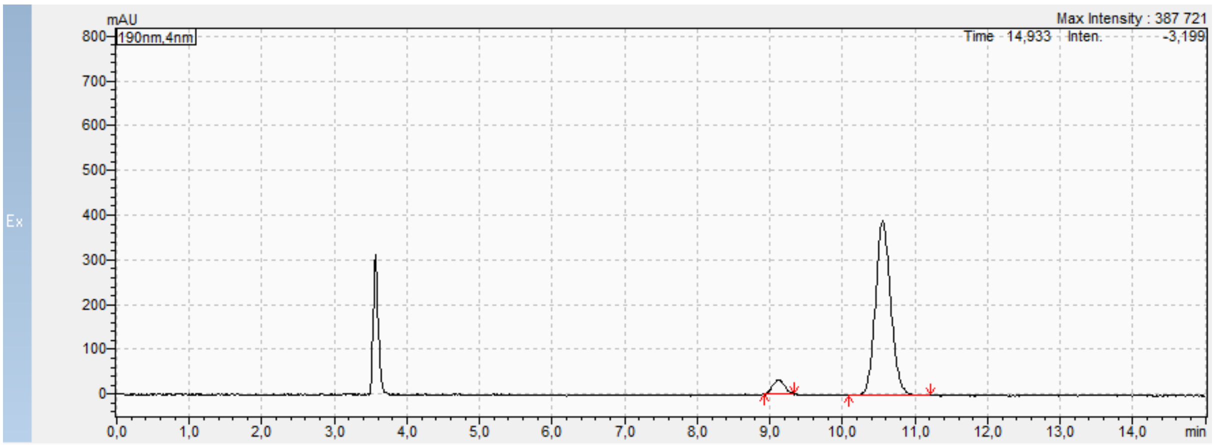

Results View - Peak Table

Peak Table | Compound | Group | Calibration Curve

Peak#	Ret. Time	Area	Height	Peak Start	Peak End	Mark	Conc.	Unit	Area%
1	9,125	366226	31530	8,917	9,333	M	5,976		5,976
2	10,559	5762211	390941	10,091	11,221	M	94,024		94,024
Total		6128437	422471				100,000		100,000

for **2q**: *er* = 94:6 *er* (*ee* = 88%)

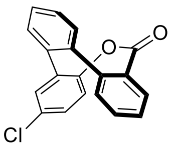

Conditions: Chiralpak IG column
mobile phase: *n*-heptane/propan-2-ol – 90:10
λ = 190 nm, V = 1.0 ml/min, t = 25 °C
for **2r**: t_R = 8.2 min (minor), t_R = 9.7 min (major)

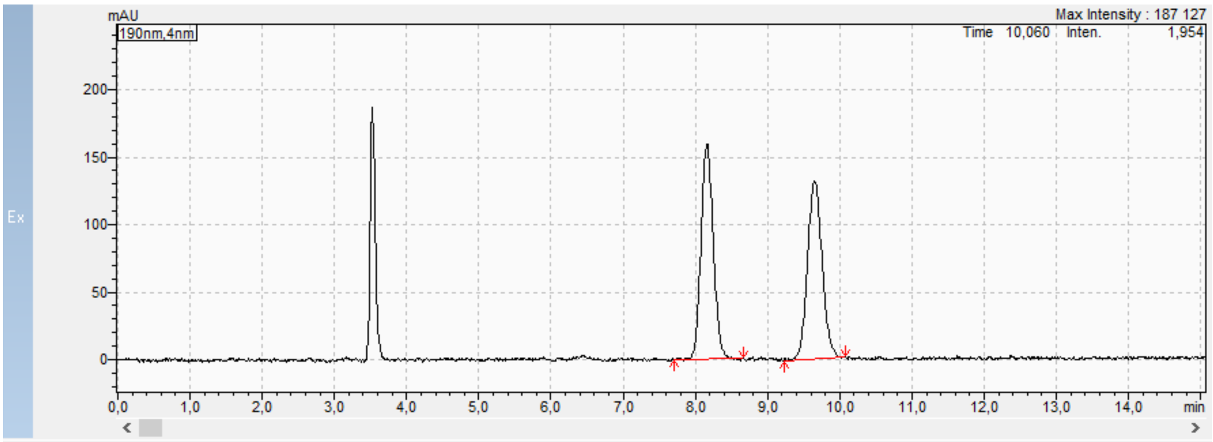

Results View - Peak Table

Peak Table | Compound | Group | Calibration Curve

Peak#	Ret. Time	Area	Height	Peak Start	Peak End	Mark	Conc.	Unit	Area%
1	8,163	1869061	159484	7,701	8,661	M	50,098		50,098
2	9,650	1861759	131340	9,227	10,080	M	49,902		49,902
Total		3730820	290824				100,000		100,000

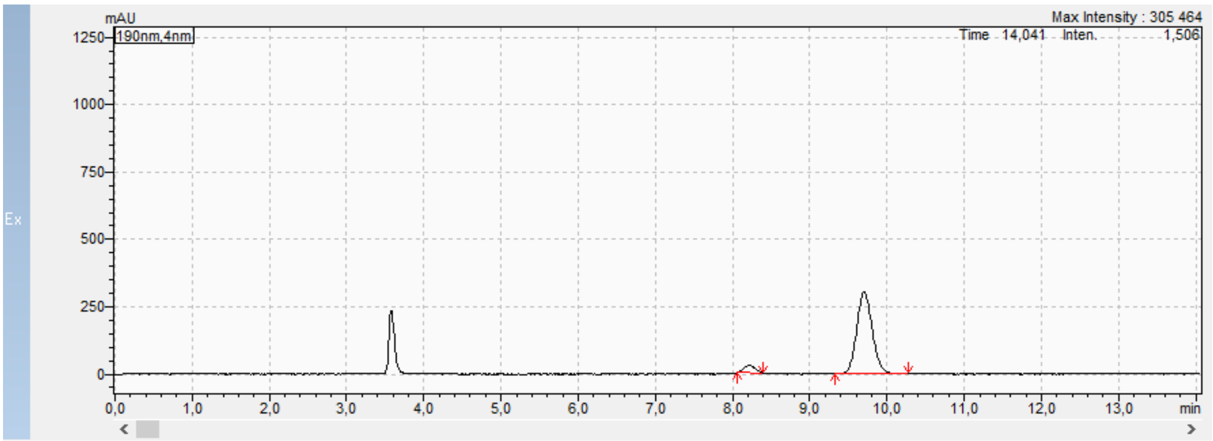

Results View - Peak Table

Peak Table | Compound | Group | Calibration Curve

Peak#	Ret. Time	Area	Height	Peak Start	Peak End	Mark	Conc.	Unit	Area%
1	8,214	280519	28700	8,064	8,395	M	6,171		6,171
2	9,704	4265552	304718	9,323	10,272	M	93,829		93,829
Total		4546071	333418				100,000		100,000

for **2r**: *er* = 94:6 *er* (*ee* = 88%)

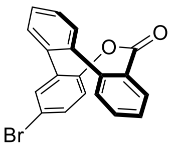

Conditions: Chiralpak IG column
mobile phase: *n*-heptane/propan-2-ol – 90:10
λ = 190 nm, V = 1.0 ml/min, t = 25 °C
for **2s**: t_R = 8.4 min (minor), t_R = 10.0 min (major)

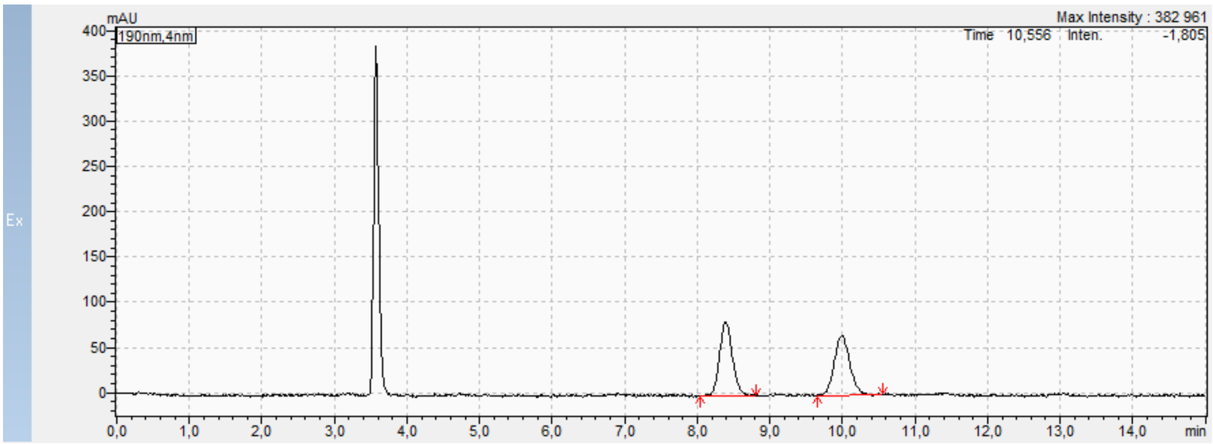

Results View - Peak Table

Peak Table | Compound | Group | Calibration Curve

Peak#	Ret. Time	Area	Height	Peak Start	Peak End	Mark	Conc.	Unit	Area%
1	8,391	1015579	81898	8,043	8,811	M	50,251		50,251
2	9,988	1005451	66174	9,653	10,560	M	49,749		49,749
Total		2021030	148072				100,000		100,000

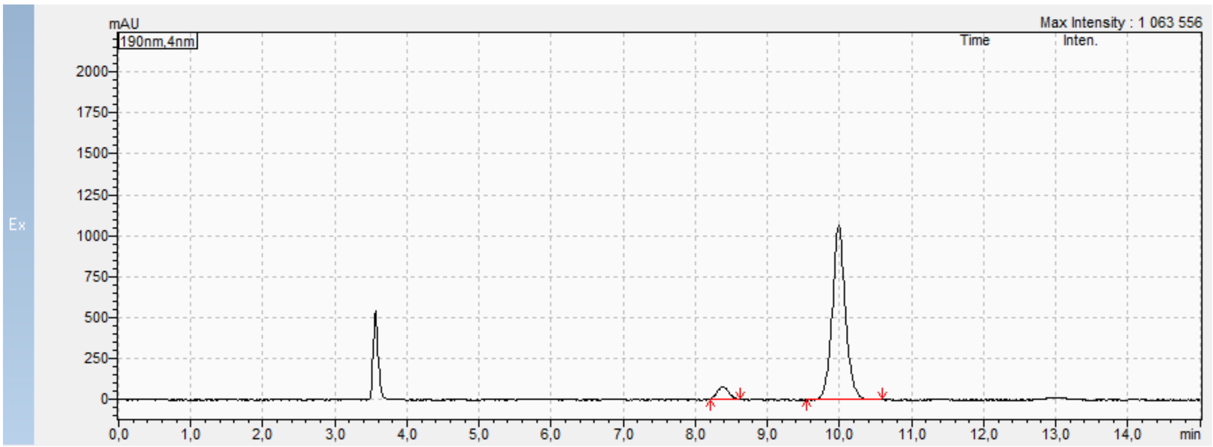

Results View - Peak Table

Peak Table | Compound | Group | Calibration Curve

Peak#	Ret. Time	Area	Height	Peak Start	Peak End	Mark	Conc.	Unit	Area%
1	8,382	829779	74865	8,213	8,619	M	5,822		5,822
2	9,990	13421768	1064557	9,536	10,603	M	94,178		94,178
Total		14251547	1139421				100,000		100,000

for **2s**: *er* = 94:6 *er* (*ee* = 88%)

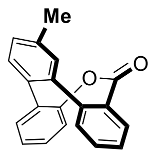

Conditions: Chiralpak IB column
mobile phase: *n*-heptane/propan-2-ol – 80:20
λ = 190 nm, V = 1.0 ml/min, t = 25 °C
for **2t**: t_R = 5.5 min (major), t_R = 7.6 min (minor)

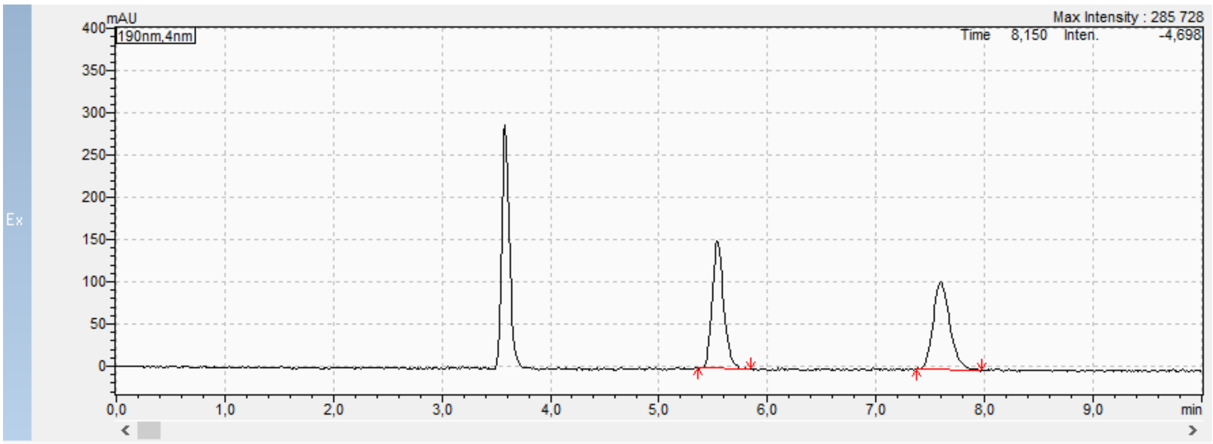

Results View - Peak Table

Peak Table | Compound | Group | Calibration Curve

Peak#	Ret. Time	Area	Height	Peak Start	Peak End	Mark	Conc.	Unit	Area%
1	5,539	1111463	150760	5,355	5,845	M	50,105		50,105
2	7,596	1106808	103752	7,371	7,979	M	49,895		49,895
Total		2218271	254512				100,000		100,000

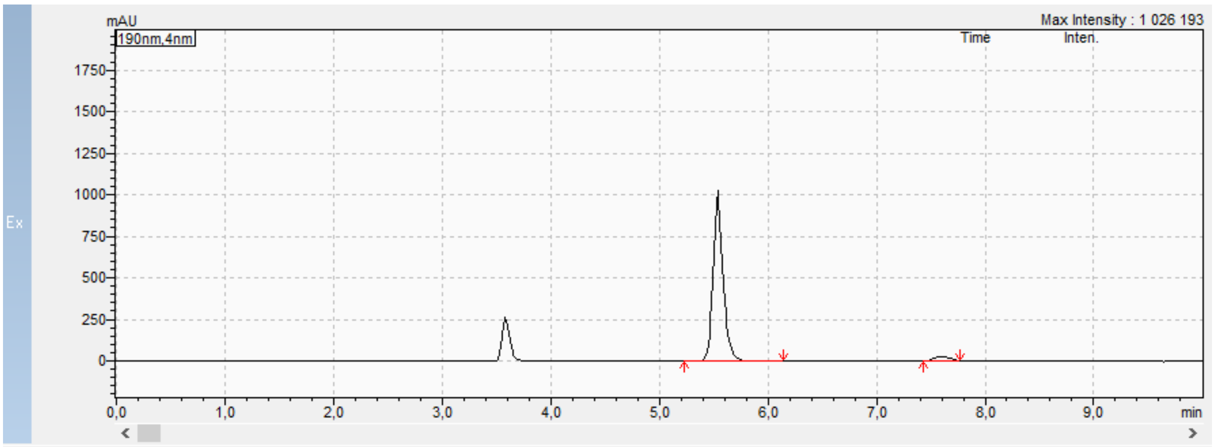

Results View - Peak Table

Peak Table | Compound | Group | Calibration Curve

Peak#	Ret. Time	Area	Height	Peak Start	Peak End	Mark	Conc.	Unit	Area%
1	5,537	6448888	1027552	5,227	6,144	M	96,071		96,071
2	7,599	263771	27646	7,435	7,765	M	3,929		3,929
Total		6712658	1055197				100,000		100,000

for **2t**: *er* = 96:4 *er* (*ee* = 92%)

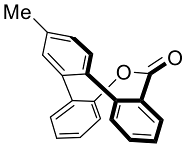

Conditions: Chiralpak IB column
mobile phase: *n*-heptane/propan-2-ol – 80:20
λ = 190 nm, V = 1.0 ml/min, t = 25 °C
for **2u**: t_R = 6.0 min (minor), t_R = 6.3 min (major)

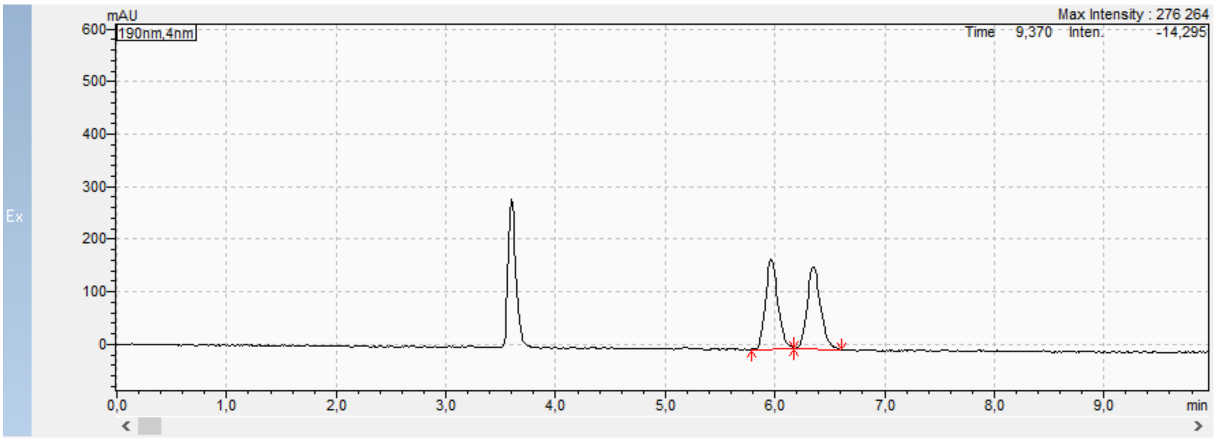

Results View - Peak Table

Peak Table | Compound | Group | Calibration Curve

Peak#	Ret. Time	Area	Height	Peak Start	Peak End	Mark	Conc.	Unit	Area%
1	5,966	1327401	170363	5,781	6,176	M	50,170		50,170
2	6,351	1318396	155569	6,176	6,613	M	49,830		49,830
Total		2645797	325932				100,000		100,000

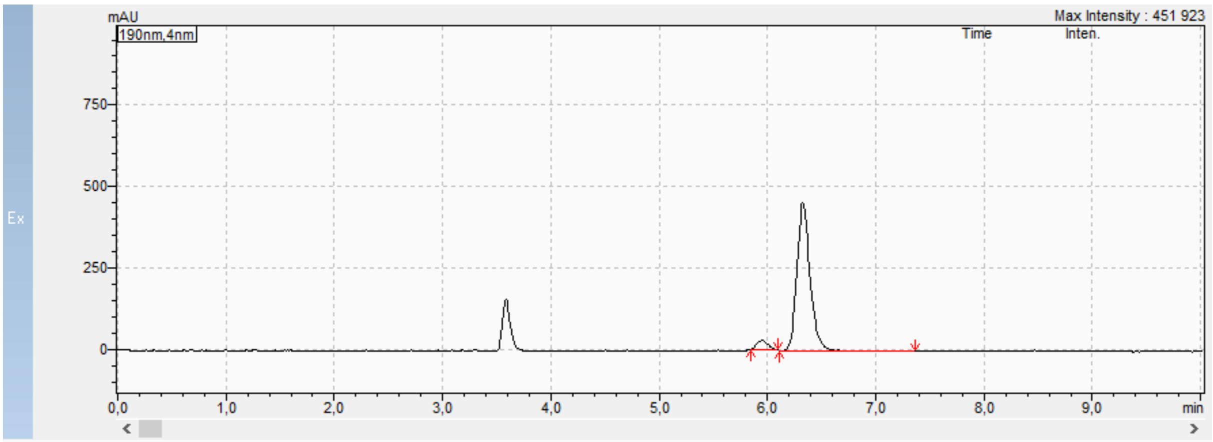

Results View - Peak Table

Peak Table | Compound | Group | Calibration Curve

Peak#	Ret. Time	Area	Height	Peak Start	Peak End	Mark	Conc.	Unit	Area%
1	5,950	214278	29394	5,845	6,101	M	5,346		5,346
2	6,327	3794215	453550	6,112	7,360	M	94,654		94,654
Total		4008493	482943				100,000		100,000

for **2u**: *er* = 95:5 *er* (*ee* = 89%)

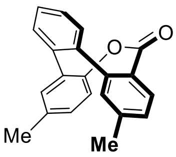

Conditions: Chiralpak IG column
mobile phase: *n*-heptane/propan-2-ol – 90:10
λ = 190 nm, V = 1.0 ml/min, t = 25 °C
for **2v**: t_R = 11.5 min (minor), t_R = 14.3 min (major)

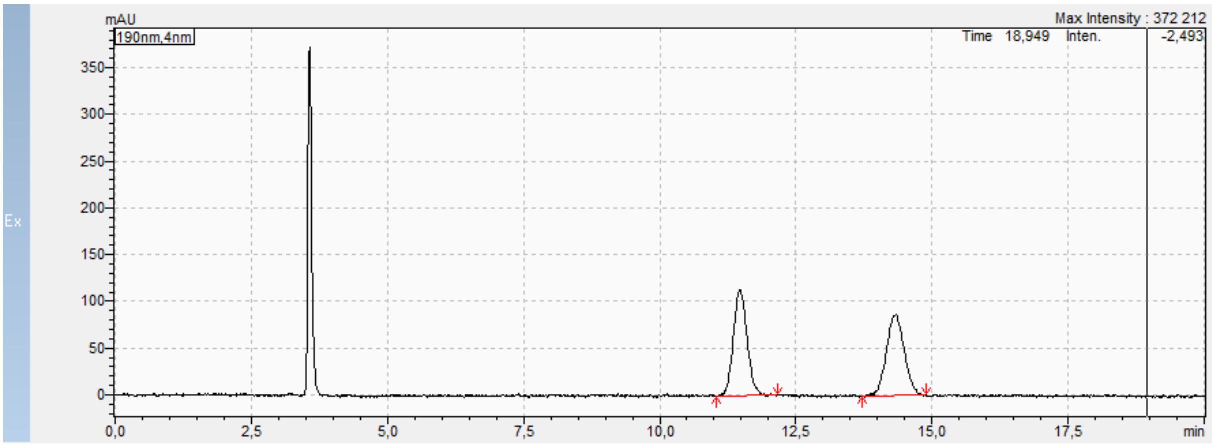

Results View - Peak Table

Peak Table | Compound | Group | Calibration Curve

Peak#	Ret. Time	Area	Height	Peak Start	Peak End	Mark	Conc.	Unit	Area%
1	11,476	2004727	113790	11,040	12,160	M	50,104		50,104
2	14,342	1996405	87026	13,717	14,891	M	49,896		49,896
Total		4001131	200815				100,000		100,000

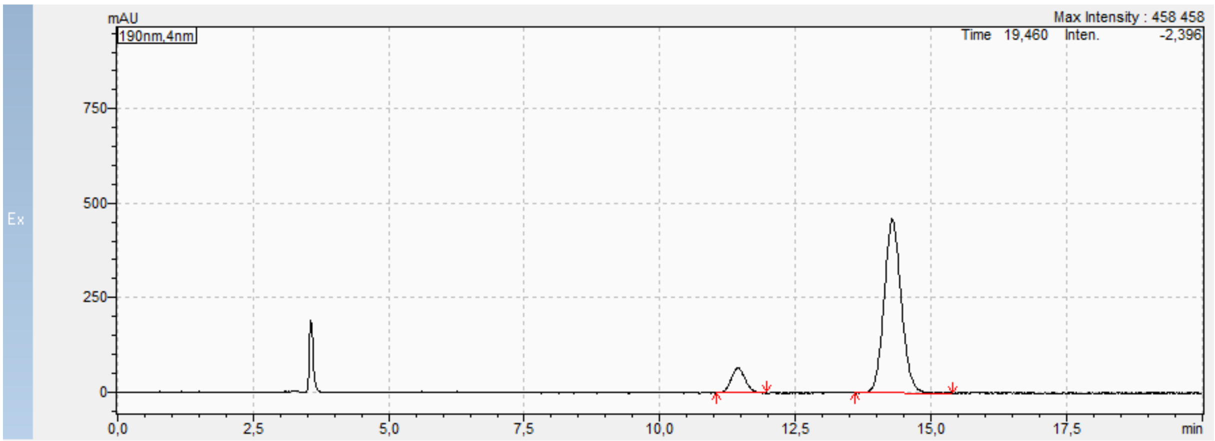

Results View - Peak Table

Peak Table | Compound | Group | Calibration Curve

Peak#	Ret. Time	Area	Height	Peak Start	Peak End	Mark	Conc.	Unit	Area%
1	11,442	1172870	66443	11,051	11,979	M	10,415		10,415
2	14,286	10088268	460681	13,611	15,413	M	89,585		89,585
Total		11261138	527124				100,000		100,000

for **2v**: *er* = 90:10 *er* (*ee* = 79%)

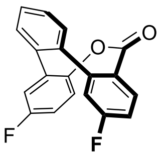

Conditions: Chiralpak IG column
mobile phase: *n*-heptane/propan-2-ol – 90:10
λ = 190 nm, V = 1.0 ml/min, t = 25 °C
for **2w**: t_R = 7.9 min (minor), t_R = 8.6 min (major)

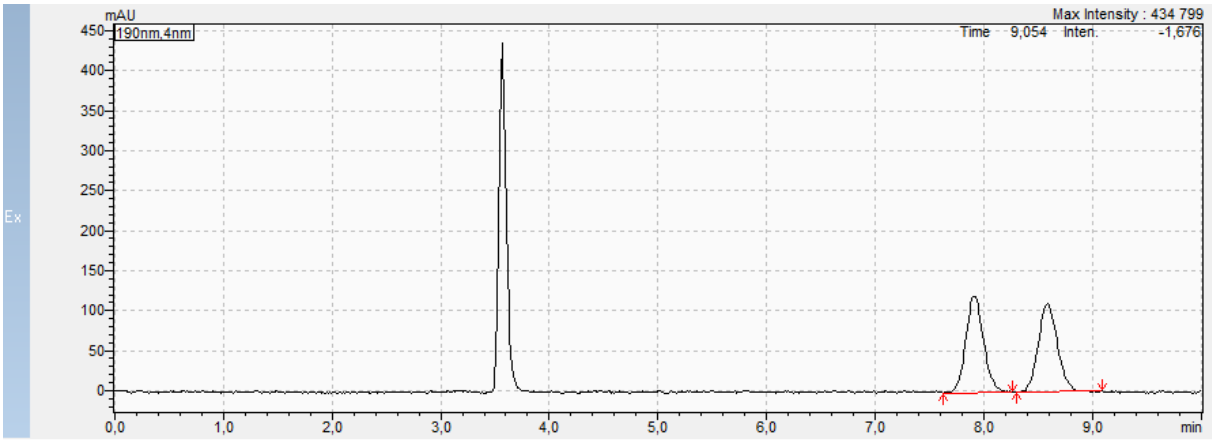

Results View - Peak Table

Peak Table | Compound | Group | Calibration Curve

Peak#	Ret. Time	Area	Height	Peak Start	Peak End	Mark	Conc.	Unit	Area%
1	7,918	1357757	119731	7,627	8,267	M	50,016		50,016
2	8,587	1356868	110803	8,299	9,088	M	49,984		49,984
Total		2714625	230534				100,000		100,000

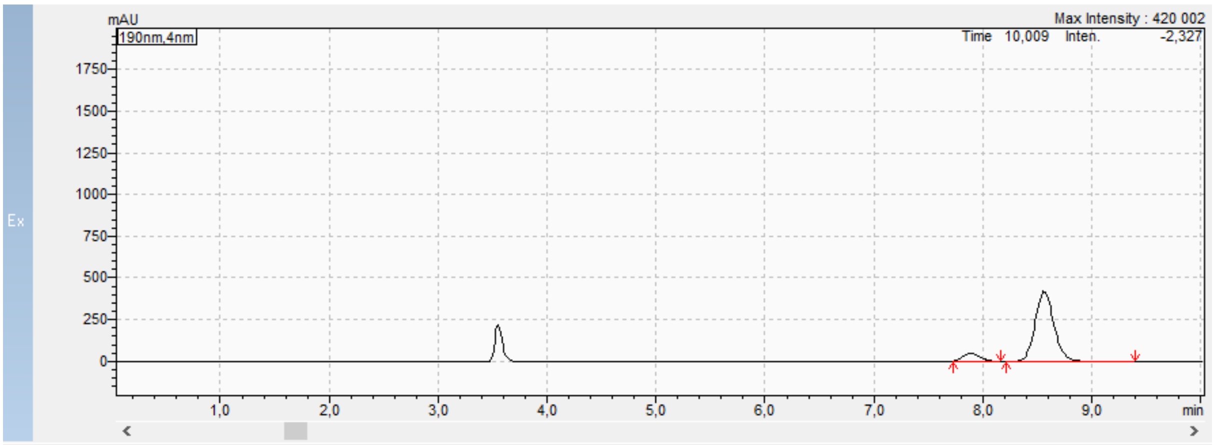

Results View - Peak Table

Peak Table | Compound | Group | Calibration Curve

Peak#	Ret. Time	Area	Height	Peak Start	Peak End	Mark	Conc.	Unit	Area%
1	7,891	511683	47674	7,723	8,160	M	9,108		9,108
2	8,558	5106383	420947	8,213	9,397	M	90,892		90,892
Total		5618066	468621				100,000		100,000

for **2w**: *er* = 91:9 *er* (*ee* = 82%)

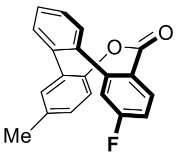

Conditions: Chiralpak IG column
mobile phase: *n*-heptane/propan-2-ol – 90:10
λ = 190 nm, V = 1.0 ml/min, t = 25 °C
for **2x**: t_R = 8.6 min (minor), t_R = 10.0 min (major)

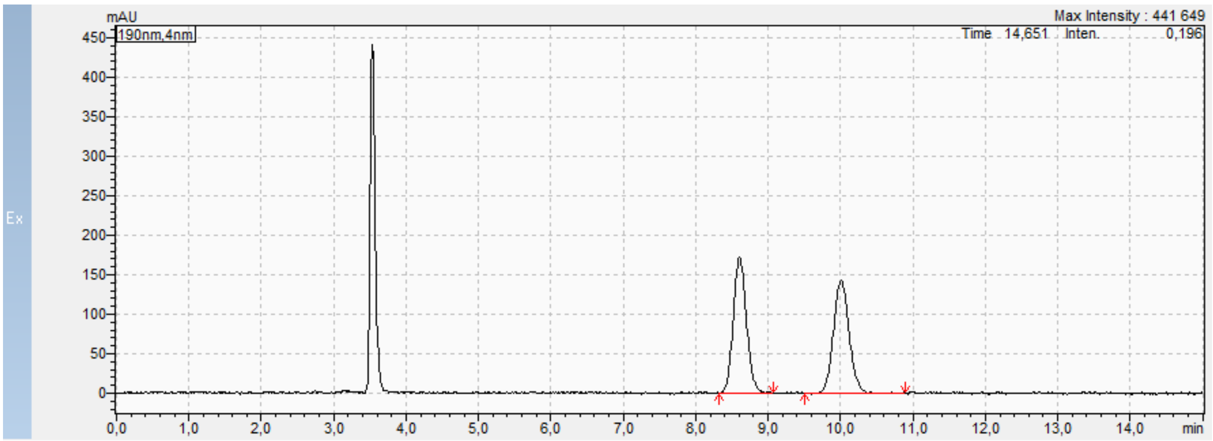

Results View - Peak Table

Peak Table | Compound | Group | Calibration Curve

Peak#	Ret. Time	Area	Height	Peak Start	Peak End	Mark	Conc.	Unit	Area%
1	8,605	2150705	171013	8,320	9,067	M	50,061		50,061
2	10,007	2145448	143458	9,504	10,891	M	49,939		49,939
Total		4296152	314471				100,000		100,000

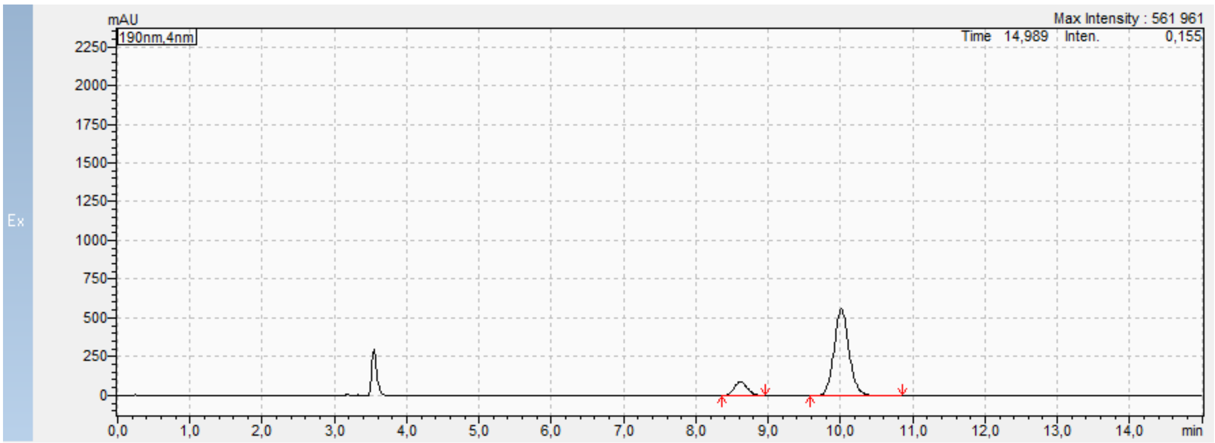

Results View - Peak Table

Peak Table | Compound | Group | Calibration Curve

Peak#	Ret. Time	Area	Height	Peak Start	Peak End	Mark	Conc.	Unit	Area%
1	8,612	1086541	87642	8,363	8,960	M	12,011		12,011
2	10,008	7959938	562461	9,579	10,848	M	87,989		87,989
Total		9046479	650103				100,000		100,000

for **2x**: *er* = 88:12 *er* (*ee* = 76%)

References

1 Q. J. Zhou, K. Worm and R. E. Dolle, *J. Org. Chem.*, 2004, **69**, 5147.

2 M. N. Kumar, S. Mahesh, J. B. Nanubolu and M. S. Reddy, *J. Org. Chem.*, 2025, **90**, 5444.

3 Y. Sumida, R. Harada, T. Sumida, K. Johmoto, H. Uekusa and T. Hosoya, *Org. Lett.*, 2020, **22**, 6687.

4 G. M. Sheldrick, *Acta Crystallogr. Sect. A Found. Crystallogr.*, 2015, **A71**, 3.

5 G. M. Sheldrick, *Acta Crystallogr. Sect. C Struct. Chem.*, 2015, **C71**, 3.

6 S. Parsons, H. D. Flack and T. Wagner, *Acta Crystallogr. Sect. B Struct. Sci. Cryst. Eng. Mater.*, 2013, **B69**, 249.

7 a) F. Neese, *WIREs Comput. Mol. Sci.*, 2025, **15**, 15, e70019; b) F. Neese, *WIREs Comput. Mol. Sci.*, 2012, **2**, 73.

8 a) S. G. Balasubramani, G. P. Chen, S. Coriani, M. Diedenhofen, M. S. Frank, Y. J. Franzke, F. Furche, R. Grotjahn, M. E. Harding, C. Hättig, A. Hellweg, B. Helmich-Paris, C. Holzer, U. Huniar, M. Kaupp, A. M. Khah, S. K. Khani, T. Müller, F. Mack, B. D. Nguyen, S. M. Parker, E. Perlt, D. Rappoport, K. Reiter, S. Roy, M. Rückert, G. Schmitz, M. Sierka, E. Tapavicza, D. P. Tew, C. Wüllen, V. K. Voora, F. Weigend, A. Wodyński and J.M. You, *J. Chem. Phys.*, 2020, **152**, 184107; b) R. Ahlrichs, M. Bär, M. Häser, H. Horn and C. Kölmel, *Chem. Phys. Lett.*, 1989, **162**, 165.

9 C. Bannwarth, S. Ehlert and S. Grimme, *J. Chem. Theory Comput.*, 2019, **15**, 1652.

10 P. Pracht, F. Bohle and S. Grimme, *Phys. Chem. Chem. Phys.*, 2020, **22**, 7169.

11 V. Ásgeirsson, B. O. Birgisson, R. Bjornsson, U. Becker, F. Neese, C. Riplinger and H. Jónsson, *J. Chem. Theory Comput.*, 2021, **17**, 4929.

12 A. Klamt and M. Diedenhofen, *J. Comput. Chem.*, 2018, **39**, 1648.

13 a) B. P. Pritchard, D. Altarawy, B. Didier, T. D. Gibson and T. L. Windus, *J. Chem. Inf. Model.*, 2019, **59**, 4814; b) N. Godbout, D. R. Salahub, J. Andzelm, and E. Wimmer, *Can. J. Chem.*, 1992, **70**, 2.

14 J. Hostaš and J. Řezáč, *J. Chem. Theory Comput.*, 2017, **13**, 3575.
